# Discovery of (3-Phenylcarbamoyl-3,4-dihydro-2*H*-pyrrol-2-yl)phosphonates as Imidazoline *I*_2_ Receptor Ligands with Anti-Alzheimer and Analgesic Properties

**DOI:** 10.1021/acs.jmedchem.4c01644

**Published:** 2025-01-17

**Authors:** Andrea Bagán, Alba López-Ruiz, Sònia Abás, M. Carmen Ruiz-Cantero, Foteini Vasilopoulou, Teresa Taboada-Jara, Christian Griñán-Ferré, Mercè Pallàs, Carolina Muguruza, Rebeca Diez-Alarcia, Luis F. Callado, José M. Entrena, Enrique J. Cobos, Belén Pérez, José A. Morales-García, Elies Molins, Steven De Jonghe, Dirk Daelemans, José Brea, Cristina Val, M. Isabel Loza, Elena Hernández-Hernández, Jesús A. García-Sevilla, M. Julia García-Fuster, Caridad Díaz, Rosario Fernández-Godino, Olga Genilloud, Milan Beljkaš, Slavica Oljačić, Katarina Nikolic, Carmen Escolano

**Affiliations:** 1 Laboratory of Medicinal Chemistry (Associated Unit to CSIC), Department of Pharmacology, Toxicology and Medicinal Chemistry, Faculty of Pharmacy and Food Sciences, University of Barcelona, Av. Joan XXIII, 27-31, Barcelona 08028, Spain; 2 Institute of Biomedicine of the University of Barcelona (IBUB), University of Barcelona, Barcelona 08028, Spain; 3 Pharmacology Section, Toxicology and Medicinal Chemistry, Faculty of Pharmacy and Food Sciences, and Institut de Neurociències, University of Barcelona, Av. Joan XXIII, 27-31, Barcelona 08028, Spain; 4 Centro de Investigación Biomédica en Red Enfermedades Neurodegenerativas (CiberNed), National Institute of Health Carlos III, Madrid 28029, Spain; 5 Department of Pharmacology, University of the Basque Country, UPV/EHU48940 Leioa, Bizkaia;; 18 Centro de Investigación Biomédica en Red de Salud Mental, CIBERSAM, Spain; 6 BioBizkaia Health Research Institute, Barakaldo, Bizkaia 48903,Spain; 7 Animal Behavior Research Unit, Scientific Instrumentation Center, Parque Tecnológico de la Salud, University of Granada, Armilla, Granada 18100, Spain; 8 Department of Pharmacology, Faculty of Medicine and Biomedical Research Center (Neurosciences Institute), Biosanitary Research Institute ibs.GRANADA, University of Granada, Granada 18016, Spain; 9 Department of Pharmacology, Therapeutic and Toxicology. Autonomous, University of Barcelona, Cerdanyola 08193, Spain; 10 Department of Cell Biology. Faculty of Medicine, Computense University of Madrid. (UCM), Madrid 28040, Spain; 11 Institut de Ciencia de Materials de Barcelona (CSIC), Campus UAB, Cerdanyola 08193, Spain; 12 Molecular, Structural and Translational Virology Research Group, Rega Institute for Medical Research, Department of Microbiology, Immunology and Transplantation, Katholieke Universiteit Leuven, Leuven 3000, Belgium; 13 Molecular Genetics and Therapeutics in Virology and Oncology Research Group, Rega Institute for Medical Research, Department of Microbiology, Immunology and Transplantation, Katholieke Universiteit Leuven, Leuven 3000, Belgium; 14 Drug Screening Platform/Biofarma Research Group, CIMUS Research Center, University of Santiago de Compostela (USC), Santiago de Compostela 15782, Spain; 15 IUNICS, University of the Balearic Islands (UIB) and IdISBa, Cra. Valldemossa km 7.5, Palma de Mallorca 07122, Spain; 16 Fundación MEDINA Centro de Excelencia en Investigación de Medicamentos Innovadores de Andalucía, Avda. Del Conocimiento 34, Ganada 10016, Spain; 17 Department of Pharmaceutical Chemistry, Faculty of Pharmacy, University of Belgrade, Vojvode Stepe 450, Belgrade 11000, Serbia

## Abstract

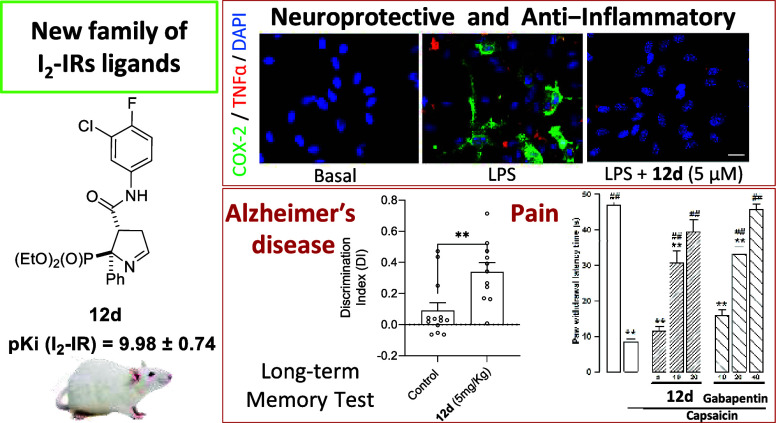

Imidazoline *I*_2_ receptors (I_2_-IRs) are altered in Alzheimer’s disease (AD) patients and are associated with analgesia. I_2_-IRs are not structurally described, and their pharmacological characterization relies on their modulation by highly affine ligands. Herein, we describe the synthesis of (3-phenylcarbamoyl-3,4-dihydro-2*H*-pyrrol-2-yl)phosphonates endowed with relevant affinities for I_2_-IRs in human brain tissues. The optimal ADME and pharmacokinetic profile of a selected compound, 12d, secured its *in vivo* exploration in a senescence accelerated prone 8 mice revealing improvement in the cognitive impairment and unveiling the mechanism of action by analyzing specific AD biomarkers. The treatment of a capsaicin-induced mechanical hypersensitivity murine model with 12d revealed analgesic properties devoid of motor coordination issues. The target engagement of 12d was demonstrated by suppression of the analgesic effect by pretreatment with idazoxan. Overall, 12d is a putative candidate for advancing preclinical phases and supports the modulation of I_2_-IRs as an innovative approach for therapeutics.

## Introduction

**Figure 1:**
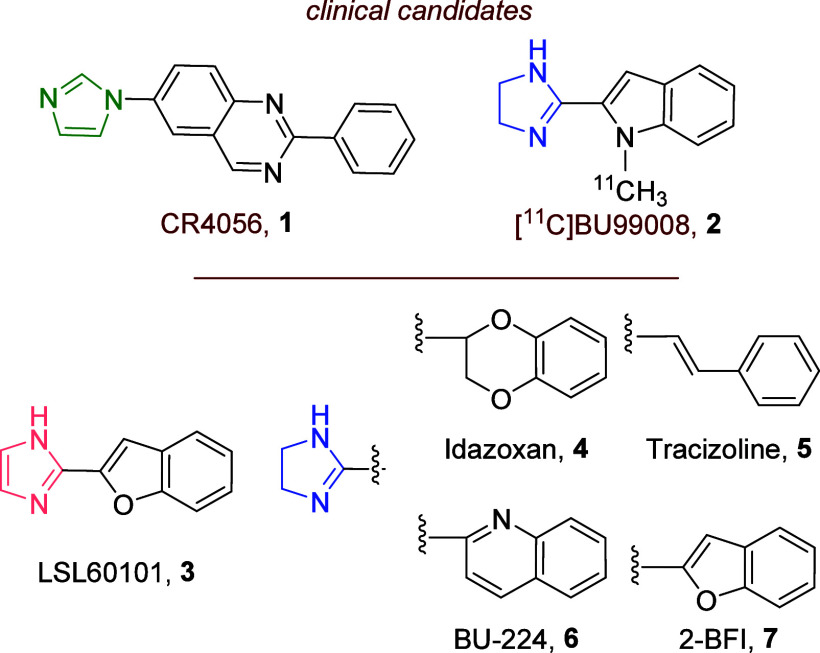
Structure of the I_2_–IRs ligands: CR4056 (**1**), [^11^C]BU99008 (**2**), LSL60101 (**3**), idazoxan (**4**), tracizoline (**5**), BU224 (**6**), and 2-BFI (**7**).

At the end of the 20th century, scientists identified imidazoline I_2_ receptors (I_2_–IRs), nonadrenergic binding sites for imidazolines, as relevant biological targets. I_2_–IRs are widely distributed in the brain and are found in organs such as the heart and the liver.^1−3^ The dysregulation of the levels of I_2_–IRs is a hallmark in human brain disorders encompassing glial tumors,^4,5^ depression,^6,7^ Alzheimer’s disease (AD),^8^ Huntington’s disease,^9^ and Parkinson’s disease (PD).^10,11^ More recently, I_2_–IRs have been validated as promising new targets in analgesia^12^ and AD diagnostics, after the progression in clinical trials of ligands CR4056 (**1,**Figure 1) and [^11^C]BU99008 (**2**, Figure 1). CR4056 (**1**),^13^ the first-in-class I_2_–IRs ligand, is in a phase II multisite randomized placebo-controlled clinical trial for knee osteoarthritis patients, and has also been reported to have a promising role in AD therapeutics.^14^ We have described sex differences in the antidepressant-like response and molecular events induced by CR4056 (**1**) in rats.^15^ [^11^C]BU99008 (**2**) is a clinical candidate in phase I for Positron Emission Tomography diagnostics in patients suffering from PD and AD.^11,16^

From the molecular point of view, I_2_–IRs are nondescribed heterogeneous proteins that are identified by their high affinity for the radiolabeled ligand [^3^H]idazoxan (**4**), and with less affinity for [^3^H]clonidine and [^3^H]*p*-aminoclonidine.^3^

**Figure 2:**
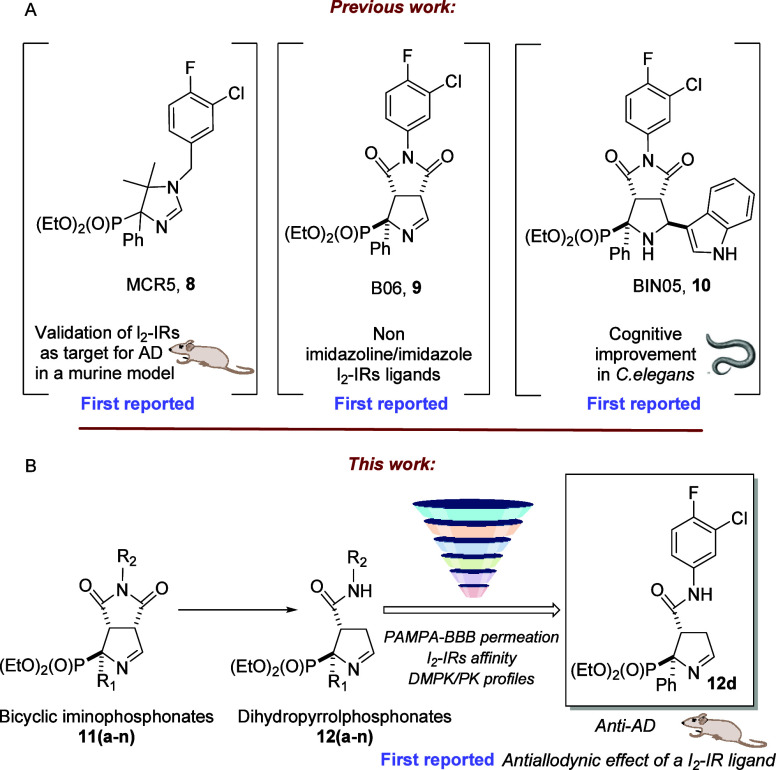
(A) Outline of the previous work and structure of compounds MCR5 (**8**), B06 (**9**), and BIN05 (**10**), and milestones achieved. (B) Outline of the present work.

Due to the absence of structural data on I_2_–IRs, their pharmacological characterization relies on the discovery of high affine and selective I_2_–IRs ligands. Unfortunately, the known I_2_–IRs ligands are scarce,^17^ and their structures are largely restricted to imidazole heterocycles, such as CR4056 (**1**) and LSL60101 (**3,**Figure 1), 2-heterocyclic-2-imidazolines, such as [^11^C]BU99008 (**2**), idazoxan (**4**, Figure 1), tracizoline (**5**, Figure 1), BU224 (**6**, Figure 1) and 2-BFI (**7**, Figure 1) and other imidazoline derivatives such as phenyzoline and diphenyzoline.^18^

Being aware of the importance of discovering I_2_–IRs ligands to build a comprehensive understanding of the pharmacological implications of I_2_–IRs, we took the challenge of developing structurally original ligands. Thus, we published that (2-imidazolin-4-yl)phosphonates derivatives were endowed with outstanding binding affinity and selectivity for I_2_–IRs.^19^ The improvement in the condition and amelioration of AD hallmarks in a murine model of age-related cognitive decline model, the senescence-accelerate mouse prone 8 (SAMP8) treated with a representative compound, the diethyl [(1-(3-chloro-4-fluorobenzyl)-5,5-dimethyl-4-phenyl-4,5-dihydro-1*H*-imidazol-4-yl]phosphonate named MCR5 (**8**, Figure 2), was the first *in vivo* experimental evidence that brought forward I_2_–IRs as a new therapeutic target for neurodegeneration,^20^ depression,^21^ and vascular disease.^22^ To support the potential of I_2_–IRs ligands in AD, we reported a disease-modifying single therapy of a murine familial AD model (5xFAD) treated with LSL60101 (**3**).^23^ We also synthesized and pharmacologically characterized, heterocyclic N1 or N2-linked imidazole skeleton compounds as new I_2_–IRs ligands, and reported the amelioration of the cognitive impairment and synaptic plasticity after the treatment of 5xFAD model with 2-(benzo[*b*]thiophene-2-yl)-1*H*-imidazole hydrochloride.^24,25^

More recently, we took on the challenge of moving beyond the imidazoline/imidazole ring to bicyclic iminophosphonates as a source of new high affine I_2_–IRs ligands. A selected compound, (1*RS*,3*aSR*,6*aSR*)-5-(3-chloro-4-fluorophenyl)-4,6-dioxo-1-phenyl-1,3a,4,5,6,6a-hexahydropyrrolo[3,4-*c*]pyrrole-1-phosphonate, named B06 (**9**, Figure 2), ameliorated the cognitive decline and improved the behavior of two murine models, SAMP8 and 5xFAD.^26,27^ Furthermore, B06 (**9**) showed promising *in vitro* ADME-Tox properties, anti-inflammatory properties, and neuroprotective properties in an *in vitro* model of PD.^28^ These bicyclic iminophosphonates are highly functionalized and we approximate their structural modification by adding nucleophiles to the imine functional group. Selected compounds with an additional indole substituent of the newly accessed bicyclic phosphoprolines family, displayed an interesting affinity for I_2_–IRs and rescued the neurodegenerative condition of a transgenic AD *Caenorhabditis elegans* model (BIN05, **10**, Figure 2).^29^

This success prompted us to continue exploring the synthetic transformation opportunities of bicyclic iminophosphonates and the pharmacological characteristics of the new compounds.

In particular, the opening of the ring embodying the imide functional group attracted our attention and disclosed a new transformation leading to unprecedented (3-phenylcarbamoyl-3,4-dihydro-2*H*-pyrrol-2-yl)phosphonates. The new compounds were completely characterized from the stereochemical point of view after performing X-ray crystallography of selected compounds. We evaluated the pharmacological profile of new compounds in human tissues by competitive studies using the selective radioligand [^3^H]2-BFI ([^3^H]**7**) and, its selectivity versus the related α_2_-adrenergic receptor (α_2_-ARs) through competition studies with the selective α_2_-ARs radioligand [^3^H]RX821002 (2-methoxyidazoxan). Analogously, the selectivity versus the related target, imidazoline I_1_ receptors (I_1_–IRs), was assessed for the most promising compounds through competition studies using [^3^H]clonidine. After a screening cascade, the representative compound, diethyl [(2*RS*,3*RS*)-3-((3-chloro-4-fluorophenyl)carbamoyl)-2-phenyl-3,4-dihydro-2*H*-pyrrol-2-yl)phosphonate (**12d**, Figure 2), was selected for further *in vitro* and *in vivo* studies.

## Results and Discussion

### Chemistry. Synthesis and Structural Characterization

**Scheme 1:**
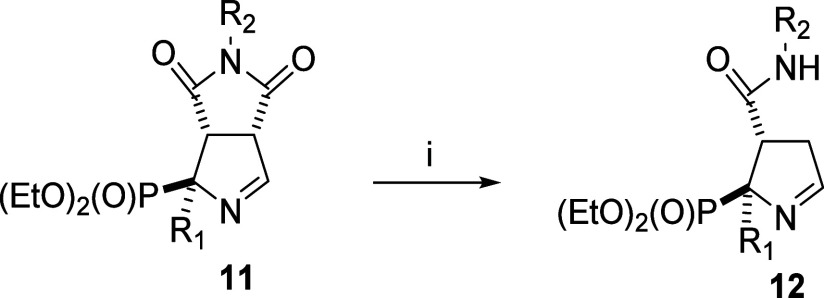
General Structures of the Starting Materials (**11**) and Final Products (**12**) *Reagents and conditions*: (i) NaOH 0.05M, 2:1 THF/H_2_O, r. t., 2.5 h.

The starting bicyclic iminophosphonates (Scheme 1, compound **9** and compounds with the general structure **11**) were prepared by diastereoselective [3 + 2]cycloaddition reaction of a diethyl isocyanomethylphosphonate derivative and an *N*-substituted maleimide following procedures described by us.^26^ The opening of the imide was performed by treatment of the bicyclic derivatives **9** and **11** with a solution of NaOH 0.05 M in a mixture 2:1 THF/H_2_O (Scheme 1). To define the scope of the reaction, the absence, or the presence of a substituent in the α-phosphonate position (R_1_ = aryl, substituted aryl, alkyl), and different substituents in the nitrogen atom of the imide (R_2_= aryl, substituted aryl or cycloalkyl) were considered. To this end, the starting bicyclic derivatives with the α-phosphonate position (R_1_) occupied with a hydrogen atom, **11a** and **11b** (Table 1, entries 1 and 2, respectively), with a phenyl group **11c**, **9**, **11e**-**11h** (Table 1, entries 3 to 8, respectively), with a 4-substituted phenyl group **11i**, **11j** and **11k, 11l** (*p*-fluoro, Table 1, entries 9 and 10, and *p*-methoxy, Table 1, entries 11 and 12, respectively), with a methyl group **11m** (Table 1, entry 13) and with a benzyl group, **11n** and **11o** (Table 1, entries 14 and 15), were subjected to the general reaction conditions to yield the 3-substitutedcarbamoyl-3,4-dihydro-2*H*-pyrrol-2-yl)phosphonates (**12a**-**12o**), in 19–75% yields (Table 1).

**Table 1:** Description of the Different Substituents, R_1_ and R_2_, Number of the Starting Materials and Final Products, and Yield of the Reaction.^a^

**Entry**	**SM**	**R**_**1**_	**R**_**2**_	**Comp**	**Yield (%)**
1	**11a**	H	Ph	**12a**	51
2	**11b**	H	3-Cl,4-FPh	**12b**	44
3	**11c**	Ph	Ph	**12c**	70
4	**9**	Ph	3-Cl,4-FPh	**12d**	76
5	**11e**	Ph	4-ClPh	**12e**	50
6	**11f**	Ph	4-PhPh	**12f**	50
7	**11g**	Ph	4-CF_3_Ph	**12g**	50
8	**11h**	Ph	cyclohexyl	**12h**	73
9	**11i**	4-FPh	Ph	**12i**	50
10	**11j**	4-FPh	3-Cl,4-FPh	**12j**	50
11	**11k**	4-MeOPh	Ph	**12k**	50
12	**11l**	4-MeOPh	3-Cl,4-FPh	**12l**	50
13	**11m**	Me	Ph	**12m**	31
14	**11n**	Bn	Ph	**12n**	19
15	**11o**	Bn	cyclohexyl	**12o**	57

a SM = starting material.

**Figure 3:**
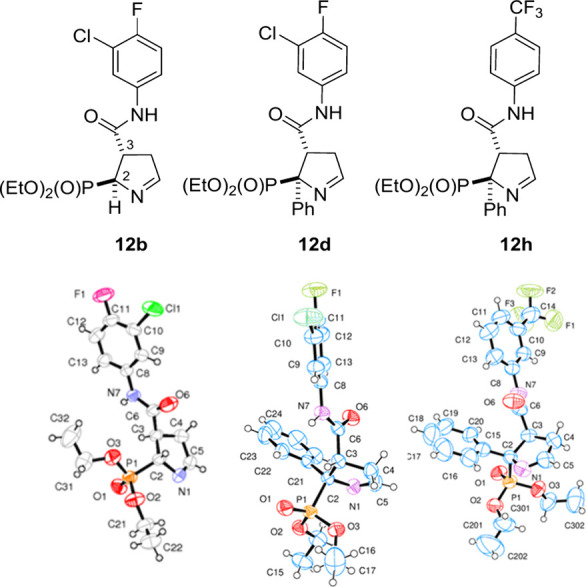
X-ray structures of **12b**, **12d** and **12h**.

To confirm that the relative configuration of the stereocenters in the bicyclic iminophosphonates was retained, the X-ray crystallography of monocrystals of compounds **12b**, **12d** and **12h** was performed. The *cis* relationship between the hydrogen atom in position 2 in **12b**, and the phenyl group in **12d** and **12h**, and the phenylcarbamoyl substituent in position 3 was unambiguously confirmed (Figure 3). The stereochemistry of the other compounds (**12**, Scheme 1) was assigned by comparison of their ^1^H and ^13^C NMR spectra (pages S3–S17, Tables S1 and S2, discussion on the spectra data S20).

**Scheme 2:**
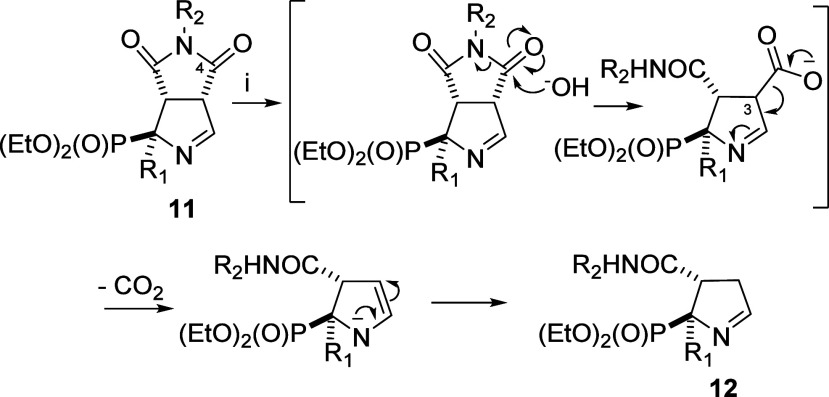
Putative Mechanism for the Formation of Compounds with the General Structure **12** from **11**. *Reagents and conditions:* (i) NaOH 0.05 M, 2:1 THF/H_2_O, r. t., 2.5 h

The reaction mechanism occurred irrespective of the presence or absence of a substituent in the α-phosphonate position or the type of substituent (phenyl, alkyl, or benzyl). To understand the molecular transformations to yield final compounds with the general structure **12**, a putative mechanism is depicted in Scheme 2. The regioselective nucleophilic attack of the anion hydroxide to the carbonyl (4-CO) produced the opening of the imide, leading to an intermediate having a carboxylic acid at the position 3 of an imine functional group that, in the reaction conditions, underwent decarboxylation leading to the monocyclic final structures. These final products (general structure **12** in Scheme 1) are unprecedented and offer a wide range of synthetic transformations due to the high degree of functionalization that will be explored in the future.

### I_2_–IR Binding Activity and Structure–Activity Relationships

The pharmacological activity of the compounds **12a**-**12o** depicted in Scheme 1 was evaluated through competition binding studies against the selective I_2_–IRs radioligand [^3^H]-2-BFI, ([^3^H]**7,**) and the selective α_2_-ARs radioligand [^3^H]RX821002. The studies were performed in membranes from the *post-mortem* human frontal cortex, a brain area that shows an important density of I_2_–IRs and α_2_-ARs.^30^ The inhibition constant (*K*_i_) for each compound was obtained and it is expressed as the corresponding p*K*_i_ (Table 2). The selectivity for these two receptors was determined by the I_2_/α_2_ index, calculated as the antilogarithm of the ratio between p*K*_i_ values for I_2_–IRs and p*K*_i_ values for α_2_-ARs (Table 2). Competition experiments against [^3^H]2-BFI, ([^3^H]**7,**) were monophasic for most of the compounds (for exceptions **12a**, **12b**, **12f, 12h, 12l** and **12n,**Table 2).

**Table 2:**
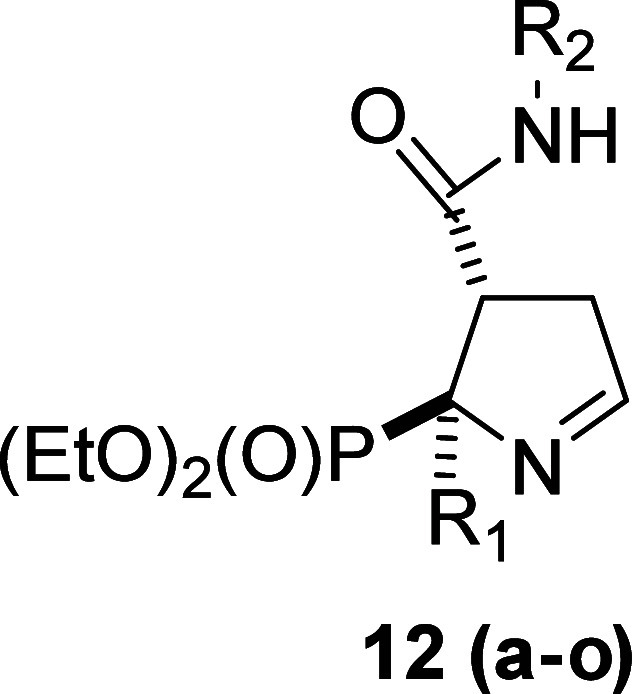

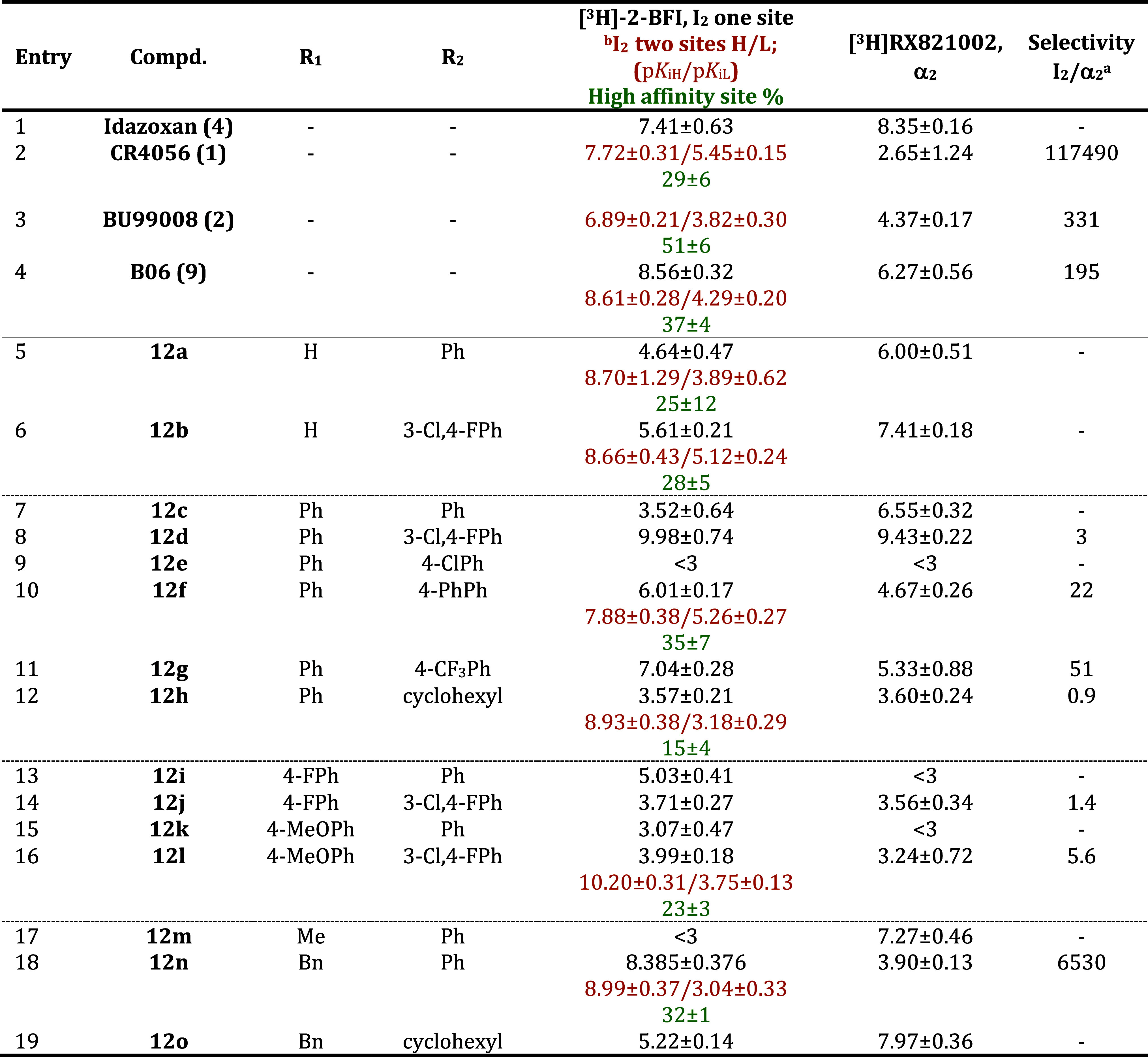
I_2_–IRs and α_2_-ARs Binding Affinities (p*K*_i_) of Four Previously Reported Compounds^25^ and New Compounds.

a Selectivity I_2_–IRs/α_2_-ARs expressed as the antilog (p*K*_i_ I_2_–IRs- p*K*_i_ α_2_-ARs).

b The best fit of the data for CR4056 **1**, BU99008 **2**, B06 **9**, **12a**, **12b**, **12f, 12h** and **12l** was to a two-site binding model of binding with high p*K*_i_ (p*K*_iH_) and low p*K*_i_ (p*K*_iL_) affinities for both binding sites respectively.

As reference compounds, we used idazoxan (**4**), a nonselective compound, with well-established affinity for I_2_–IRs (monophasic curve, p*K*_i_ = 7.41 ± 0.63) and α_2_-ARs (p*K*_i_ = 8.35 ± 0.16) (Table 2, entry 1), as well as the clinical candidates CR4056 (**1**)^31^ and BU99008 (**2**)^32^ that were synthesized following described procedures. We reported the affinity of both clinical candidates for human I_2_–IRs (Table 2, entries 2 and 3, respectively).^26^ Additionally, as the structurally closest compound, we selected our representative compound B06 (**9**) (Table 2, entry 4). The affinity of these compounds for I_2_–IRs is better described with a biphasic curve, CR4056 (**1**), p*K*_iH_ I_2_ = 7.72 ± 0.31 (29% occupancy) and p*K*_iL_ I_2_ = 5.45 ± 0.15 (Table 2, entry 2), BU99008 (**2**), p*K*_iH_ I_2_ = 6.89 ± 0.21 (51% occupancy) and p*K*_iL_ I_2_ = 3.82 ± 0.30 (Table 2, entry 3), and B06 (**9**), p*K*_iH_ I_2_ = 8.61 ± 0.28 (37% occupancy) and p*K*_iL_ I_2_ = 4.29 ± 0.20 (Table 2, entry 4).

The compounds with the general structure **12** where R_1_ is a hydrogen atom, and R_2_ is an aryl derivative, **12a** and **12b**, were not selective (I_2_/α_2_) and fitted best into a two-site model of binding with p*K*_iH_ I_2_ = 8.70 ± 1.29 (*K*_i_ = 1.99 nM, 25% occupancy) and p*K*_iL_ I_2_ = 3.89 ± 0.62 and p*K*_iH_ I_2_ = 8.66 ± 0.43 (*K*_i_ = 2.18 nM, 28% occupancy) and p*K*_iL_ I_2_ = 5.12 ± 0.24, respectively. The two compounds depicted better affinity for the I_2_–IRs than the three standard compounds (Table 2, entries 1–3), but no I_2_/α_2_ selectivity. As a first structural approximation, we turned our attention to compounds bearing a quaternary center in the α-position by including a phenyl group. This modification was highly deleterious for the affinity in compound **12c** (R_1_, R_2_ = Ph, p*K*_i_ 3.52 ± 0.64, Table 2, entry 7). To maintain the homology with **12b** (R_1_= H, R_2_= 3-Cl,4-FPh) compound **12d** (R_1_= Ph, R_2_= 3-Cl,4-FPh) was evaluated. A remarkable benefit was observed by an increase in the affinity to a p*K*_i_ = 9.98 ± 0.74 (*K*_i_ = 1.05 nM) with no I_2_/α_2_ selectivity (Table 2, entry 8). The introduction of halogen atoms in the R_2_ = Ph, as in **12e** (R_2_ = 4-ClPh), was highly deleterious for the affinity (Table 2, entry 9). Compound **12f** (R_2_ = 4-PhPh) showed an affinity property that fits best with a two-site model of binding, p*K*_iH_ I_2_ = 7.88 ± 0.38 (35% occupancy) and p*K*_iL_ I_2_ = 5.26 ± 0.27 (Table 2, entry 10), without overpassing the standard I_2_–IRs ligands (Table 2, entries 1–3). The presence of a 4-CF_3_ substituent in the phenyl R_2_ substituent in the compound **12g** led to an affinity (p*K*_i_ 7.04 ± 0.28) in the range of the idazoxan (**4**) but with an improved selectivity ratio of 51 (I_2_/α_2_).

After exploring compounds **12c**, **12d**, **12e**, **12f,** and **12g** with *N*-aryl substituents, we synthesized compound **12h** with a *N*-cyclohexyl substituent showing a biphasic curve with a p*K*_iH_ I_2_ = 8.93 ± 0.38 but with only a 15% occupancy and p*K*_iL_ I_2_ = 3.18 ± 0.29 (Table 2, entry 12). Next, we considered modifications in the α-phenyl substituent. For maintaining the homology with the first considered compounds (**12a** and **12c**, R_2_ = Ph, and **12b** and **12d**, R_2_ = 3-Cl,4-FPh), we synthesized compounds **12i** and **12j** (R_2_ = Ph, and 3-Cl,4-FPh, respectively) with a 4-fluorine atom in the α-phenyl substituent and compounds **12k** and **12l** (R_2_ = Ph, and 3-Cl,4-FPh, respectively) with an electron donating group, 4-methoxy, in the α-phenyl substituent. The presence of an additional fluorine atom did not restore the affinity with a p*K*_i_ I_2_ = 5.03 ± 0.41 for **12i** (Table 2, entry 13) and p*K*_i_ I_2_ = 3.71 ± 0.27 for **2j** (Table 2, entry 14). The 4-methoxyphenyl group was not the right choice in the case of compound **12k** (p*K*_i_ I_2_ = 3.07 ± 0.47, Table 2, entry 15) but showed better activity in compound **12l**, considering the biphasic fitted curve p*K*_iH_ I_2_ = 10.20 ± 0.31 (23% occupancy) and p*K*_iH_ I_2_ = 3.75 ± 0.13.

Finally, the substitution of an α-phenyl group for an alkyl substituent, R_1_ = Me in compound **12m** displays negligible p*K*_i_ I_2_ (Table 2, entry 17). Compound **12n** where the alkyl substituent is a benzyl group and R_2_ is phenyl exhibit a biphasic fitted curve, p*K*_iH_ I_2_ = 8.99 ± 0.37, with 33% occupancy and p*K*_iL_ I_2_ = 3.04 ± 0.33 and a remarkable selectivity I_2_/α_2_ 6530 (Table 2, entry 18)_._ The affinity drops in compound **12o** bearing the α-benzyl subtituent and with an R_2_ cyclohexyl substituent, p*K*_i_ I_2_ = 5.22 ± 0.14 (Table 2, entry 19).

### Selectivity for I_2_–IR Versus I_1_–IR

To further evaluate the selectivity of new compounds, apart from considering α_2_-ARs (Table 2), we assessed the affinity of selected compounds for the related I_1_–IRs. The competition binding site assays for I_1_–IRs, using [^3^H]clonidine as radioligand, were conducted in membranes obtained from the rat kidney using as reference moxonidine, a known I_1_–IRs selective compound. Compounds **12a**, **12b**, **12d**, **12h**, **12l** and **12n**, with an affinity value (p*K*_i_ or p*K*_iH_) for the I_2_–IRs above 8 were selected (Table 2). The results are summarized in Table S18, and only deserved a mention, the values of p*K*_i_ 7.84 ± 0.24 and 7.58 ± 0.47 for compounds **12a** and **12c**, respectively. Therefore, there was not significant interaction with I_1_–IRs highlighting the I_2_–IRs selective behavior of the herein reported family. This fact allows us to exclude that the new compounds may have secondary effects on blood pressure through the activation of I_1_–IRs.^33^

### Three-Dimensional Quantitative Structure–Activity Relationship Study

A comprehensive three-dimensional quantitative structure–activity relationship (3D-QSAR) study was performed to gain insight into the structural features that are critical for the activity and selectivity for I_2_–IRs of new compounds with the general structure **12** (Scheme 1). Also, a comparison of these results was carried out with already established 3D-QSAR models of starting materials (bicyclic iminophosphonates, Scheme 1).^26^

The results of the external validation confirmed that both 3D-QSAR models reliably predict I_2_–IRs and α_2_-ARs activity (Tables S19 and S20). The key variables that correlate with I_2_–IRs activity and selectivity are further discussed and evaluated.

**Figure 4:**
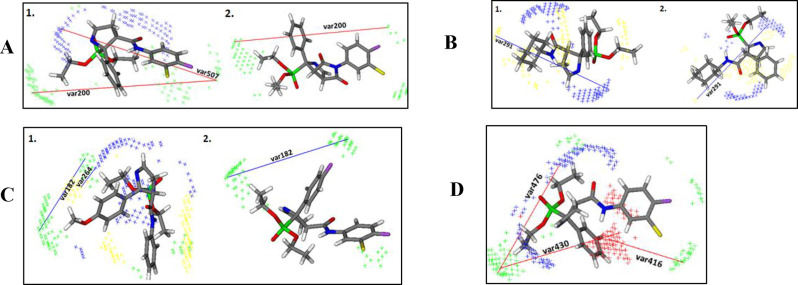
(A1). Favorable var200: TIP-TP and favorable var507: N1-TIP of compound **12d** (3D-QSAR I_2_–IRs model); (A2) Favorable var200: TIP-TIP of compound B06 **9**; the steric hot spots (TIP) are presented in green and H-bond acceptor regions in blue. (B1) Unfavorable var291: DRY-N1 of compound **12h** (3D-QSAR I_2_–IRs model); (B2) Unfavorable var291: DRY-N1 of compound **12n** (3D-QSAR I_2_–IRs model); the hydrophobic regions (DRY) are labeled in yellow and H-bond acceptor regions in blue; (C1) Favorable var182: TIP-TIP and favorable var264: DRY-N1 of compound **12k** (3D-QSAR I_2_–IRs model); (C2) Favorable var182: TIP-TIP of compound **12j** (3D-QSAR I_2_–IRs model); the hydrophobic regions (DRY) are labeled in yellow, the steric hot spots (TIP) are presented in green and H-bond acceptor regions in blue. (D) Favorable var416: O-TIP, favorable var430: O-TIP and favorable var476: N1-TIP of compound **12d** (3D-QSAR α_2_-ARs model); the steric hot spots (TIP) are presented in green, H-bond acceptor regions in blue and H-bond donor regions in red.

Var200 (TIP-TIP: 17.6 Å – 18.0 Å) is observed between the steric hot spots around the *para*-substituents of the phenyl ring (such as fluorine, trifluoromethyl and phenyl) and the steric hot spots around the ethoxy group. This variable appears in almost all the most active compounds (**12d**, **B06**, etc) (Figure 4A), suggesting that the introduction of substituents at the *para*-position of the phenyl ring can positively correlate with the overall I_2_–IRs activity. Furthermore, this variable is absent in the least active compound, **12k**, which has no substitutions on the phenyl ring, supporting this conclusion. Likewise, the contribution of *para*-substitution to I_2_–IRs activity can be explained by var507 (N1-TIP:15.6 Å – 16.0 Å), which emphasizes the importance of the optimal distance between the steric hot spots around the *para*-substituents of the phenyl ring and the hydrogen-accepting group (the nitrogen atom of 1-pyrroline or carbonyl group) in the most active compound **12d**. Notably, this variable is also not present in the least active compound, **12k**, nor is var200.

The significance of the phenyl ring attached to the amide group for I_2_–IRs activity is evidenced by var350 (DRY-TIP: 15.2 Å – 15.6 Å), which reflects the optimal distance between the steric hot spots around the phenyl ring and the hydrophobic regions around the ethoxy group. In contrast, this variable is absent in compounds that have cyclohexane substituents instead of the phenyl ring, such as **12h** and **12n**, which exhibit low I_2_–IRs activity (p*K*_i_ I_2_ = 3.57 and p*K*_i_ I_2_ = 5.22, respectively). In addition, the negative impact of cyclohexane on I_2_–IRs activity is underlined by unfavorable var291 (DRY-N1:12.4 Å −12.8 Å) that is observed in **12h** and **12n** (Figure 4B) between the hydrogen accepting group (nitrogen atom of 1-pyrroline) and the hydrophobic regions around the cyclohexane ring. This led us to conclude that the phenyl ring is important for I_2_–IRs activity.

Finally, the introduction of *para*-substituents on the phenyl ring (C-5 of 1-pyrroline) correlates negatively with I_2_–IRs activity, as shown by the unfavorable var264 (DRY-N1:1.6 Å – 2.0 Å) and the unfavorable var182 (TIP-TIP: 10.4 Å −10.8 Å) in the less active compounds of the data set (**12j** and **12k**, Figure 4C). Var264 indicates the negative influence of the distance between the hydrogen-accepting group of 1-pyrroline (the nitrogen atom) and the hydrophobic regions around the *para*-substituents of the phenyl ring, such as fluorine and methoxy group, on the I_2_–IRs activity. In parallel, var182 points out the negative effects of the distance between steric hot spots of these substituents and ethoxy groups on the overall I_2_–IRs activity.

The 3D-QSAR (α_2_-ARs) model identifies var476 (N1-TIP: 5.6 Å −6.0 Å) as one of the key factors contributing to α_2_-ARs activity (Figure 4D). This variable represents the optimal distance between a hydrogen-accepting group, such as a carbonyl group, and a hot spot region around the ethoxy group. In contrast, the 3D-QSAR (I_2_–IRs) model does not highlight the carbonyl group as critical for I_2_–IRs activity. This suggests that changes to this part of the molecule could potentially decrease α_2_-ARs activity while increasing selectivity for I_2_–IRs.

Two variables, var430 (O-TIP:12.0 Å – 12.4 Å) and var416 (O-TIP:16.4 Å −16.8 Å), indicate the positive correlation between the presence of a hydrogen-donating group –the nitrogen atom of the amide - and α_2_-ARs activity, suggesting that the substitution of this atom is likely to decrease α_2_-ARs activity while being important for selectivity toward I_2_–IRs, bearing in mind that the 3D-QSAR (I_2_–IRs) model does not imply the importance of this structural feature for I_2_–IRs activity (Figure 4D).

In summary, we find that the presence of phenyl rings (positions C-4 and C-5 of 1-pyrroline) and ethoxy groups positively correlates with I_2_–IRs activity, as shown by var351 and var291. The introduction of *para*-substituents into the phenyl ring at position C-4 can have a favorable effect on I_2_–IRs activity, as indicated by the favorable var200 and var507, whereas substitution of the phenyl ring at C-5 leads to a decrease in I_2_–IRs activity, as shown by the unfavorable var264 and var182. Finally, the modification of the amide group could be important to increase I_2_–IRs activity and selectivity, which is consistent with the influence of var476, var430 and var416 on α_2_-ARs activity.

Similar to the previously described 3D-QSAR model,^26^ the phenyl ring was identified as a critical structural feature for I_2_–IRs activity, especially with respect to the *para*-substituents on the phenyl ring (C-4 position of the 1-pyrroline moiety). In contrast to the previous 3D-QSAR model, which suggested that *meta*- and *para*-substituents on the phenyl ring enhance both I_2_–IRs activity and selectivity, the current model shows that these substituents are indeed essential for I_2_–IRs activity but have no effect on selectivity. This result is consistent with experimental data, as the most active molecule for α_2_-ARs, **12d**, has these substituents. Furthermore, the new model shows that *para*-substitution of the phenyl ring at the C-5 position can have negative effects on the overall I_2_–IRs activity and should not be considered in the further development of inhibitors.

In conclusion, the 3D-QSAR models show that strategic modification of the phenyl rings and selective substitution patterns as well as modification of the amide group can increase I_2_–IRs activity while improving selectivity toward α_2_-ARs, providing valuable guidance for the development of more selective I_2_–IR ligands.

### *In Silico* ADMET Analysis of Physicochemical and Pharmacokinetic Parameters

*In silico* prediction of absorption, distribution, metabolism, excretion and toxicity (ADMET) aims to evaluate the individual pharmacokinetic properties of the investigated drugs, which is an important step in lead optimization. The pharmacokinetic profiles of the compounds were determined using the ADMET Predictor software,^34^ while the physicochemical parameters were determined using the online program SwissADME.^35^ The predicted parameters are listed in Tables S21 and S22. From the results of the calculations performed, we can conclude that all the compounds studied have good water solubility and lipophilicity. The compounds presented here fulfill the Lipinski’s Rule of 5, which speaks for their drug-like properties and potential chance of oral bioavailability. In addition, the polarity of the compounds was evaluated by TPSA (topological polar surface area) and the results showed that the studied compounds had higher polarity than CR4056 (**1**), BU99008 (**2**), and idazoxan (**4**). In terms of pharmacokinetic properties, all molecules were found to exhibit high permeation into the BBB. Compared to standard molecules such as BU99008 (**2**) and idazoxan (**4**), all studied compounds showed lower levels of unbound drug in plasma. In addition, this family showed a lower metabolic CYP risk than idazoxan (**4**) and a lower TOX risk compared to CR4056 (**1**), BU99008 (**2**), and idazoxan (**4**). P-gp is thought to play an important role in drug distribution and resistance to CNS drugs. The investigated compounds were not identified as potential substrates for P-gp transporters. Finally, the compounds studied did not show a significantly different probability of inhibiting hERG channels compared to idazoxan (**4**). As there were no caveats in this theoretical study, we are confident to perform further *in vitro* and eventually *in vivo* experiments to evaluate the new compounds as neuroprotective agents.

### PAMPA-BBB Permeation Assay

Considering the location of I_2_–IRs in the CNS, a good ability to cross the BBB is an essential requirement for developing effective I_2_–IRs ligands with potential therapeutic applications in the neuroprotective field. For this reason, the *in vitro* permeability (Pe) of all the novel compounds was determined by using the PAMPA-BBB permeability assay (Table S24). Most of the new compounds prepared were well above the threshold established by Di et at.^36^ for high BBB permeation (Pe > 5.198 × 10^–6^ cm s^–1^), while **12b**, **12k** and **12m** had values considered of uncertain BBB permeation (CNS ± : 5.198 > *P*e (10^–6^ cm s^–1^) > 2.054).

### Cytotoxicity

Since compounds **12a** and **12d** displayed high binding affinity values for I_2_–IRs (Table 2, entries 5 and 8), they were selected for a broad cytotoxicity screening against different mammalian cell lines, such as HeLa (human cervix carcinoma), Vero (African green monkey kidney), MDCK (Mandin-Darby canine kidney), and MT4 (human T-lymphocyte). None of the two compounds showed any cytotoxicity at 100 μM, the highest concentration tested. In addition, compounds **12a**, **12c**, **12d**, **12f**, **12g**, **12h**, **12i**, **12j**, **12k** and **12l** lacked cytotoxicity (CC_50_ > 100 μM) when evaluated in a panel of cancer cell lines including LN-229, Caspan-1, Hap-1, HCT-116, NCI-H460, DND-41, HL-60, K-562 and Z-138 cell lines. Moreover, compound **12d** was also tested in MRC-5 (human embryonic lung fibroblast) cells and no cytotoxicity was observed (CC_50_ > 100).

Considering its affinity for I_2_–IRs, noncytotoxicity and predicted BBB-penetrance, we selected compound **12d** for further characterization as I_2_–IRs ligand.

### ADME-DMPK Profiling of 12d

The determination of the following parameters is recommended for predicting drug absorption and avoiding misleading *in vivo* results. The solubility of **12d** was excellent (>100 μM) in a mixture of 1% DMSO and a 99% PBS buffer. The chemical stability of **12d** in buffers at different pHs was undertaken to assess its degradation by no-enzymatic processes (e.g., hydrolysis and oxidation). The pH value of the gastrointestinal tract varies from acidic in the stomach to basic in the small and large intestine, and alterations of the representative compound **12d** need to be assessed. To this end, a test solution of **12d** (1 μM, 0.1% final DMSO concentration) was incubated with a buffer at pH 2, 5, and 7.4 at 37 °C. The analysis of the samples taken at 0, 5, 30, 60, and 120 min by LC-MS/MS reveals that **12d** was stable at the pHs and times studied (Tables S25–S27). The permeability across a human colon carcinoma cell line Caco-2 monolayer indicated an optimal prediction for the intestinal absorption of **12d**. The parameters of microsomal stability, plasma stability, and protein binding were evaluated in different species (human, mouse, and rat), ensuring confidence in **12d** to progress. The concentration for the inhibition of cytochromes [CYP1A2, CYP2C9, CYP2C19, CYP2A4 (BFC and DBF), and CYP2D6] was 10 μM, far away from the nanomolar *K*_i_ of the affinity of **12d** for the I_2_–IRs. The inhibition of the hERG potassium channel was negligible considering the nanomolar range of actuation of **12d**. The results of this study warrant the confidence in the progression of **12d** in further studies (see the parameters and conditions of the assays in the Supporting Information).

### *In Vitro* Effects of 12b in a Preclinical Model of Neurodegeneration

**Figure 5:**
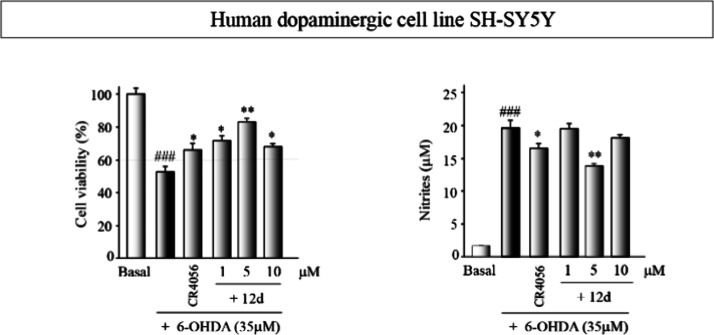
Neuroprotective effect of compound **12d**. SH-SY5Y cultures were exposed to 6-OHDA (35 μM) for at least 18 h. Previously, some cultures were pretreated 1h before with different concentrations of the compound **12d**. CR4056 (**1**) (1 μM) was used as a reference. Basal cultures were treated with vehicle. Cell viability was evaluated using the 3-(4,5-dimethylthiazol-2-yl)-5-(3-carboxymethoxyphenyl)-2-(4-sulfophenyl)-2*H*-tetrazolium (MTT) assay, and nitrite production in the cell supernatant was quantified using the Griess reaction. The reported values represent the mean ± SD obtained from triplicate determinations repeated at least three times. Statistical analysis was performed: *p ≤ 0.05, **p ≤ 0.01 (versus 6-OHDA-treated cultures); ^###^*p* ≤ 0.001 (compared to basal cultures).

Neurodegenerative diseases are primarily characterized by neuronal death accompanied by a strong neuroinflammatory process that contributes to disease progression. Therefore, we utilized two well-known *in vitro* models of neurodegeneration and neuroinflammation. One model is based on the use of the neurotoxin 6-hydroxydopamine (6-OHDA), which induces cytotoxic stress in SH-SY5Y neuronal cultures. The other model uses lipopolysaccharide (LPS) as an inducer of glial hyperactivation in primary astrocyte and microglial cultures, mimicking the inflammatory process characteristic of neurodegenerative disorders. Initially, we assessed the potential cytotoxic effect of the compound **12d** itself. For this, we treated neuronal and glial cultures with various concentrations of the compound. The results of cell survival assays and nitrite measurement tests, as an indicator of inflammation, demonstrate that the compound is nontoxic at the assessed concentrations (Figure S6). Subsequently, we evaluated the neuroprotective capacity of the compound **12d** in a neuronal damage model. The results shown in Figure 5 demonstrate that compound exerts a significant neuroprotective effect against 6-OHDA-induced damage at all tested concentrations. This analysis also included the clinical candidate CR4056 (**1**) at 1 μM concentration as a reference.

**Figure 6:**
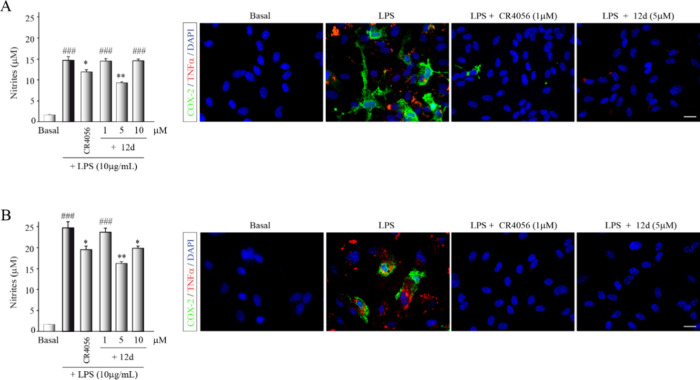
Role of compound **12d** in an *in vitro* neuroinflammation model. Glial cells, astrocytes (A) and microglial cells (B) were isolated from murine cortex and stimulated with bacterial LPS (10 μg/mL) in the presence of the compound **12d** (at different doses). CR4056 **1** (1 μM) was used as a reference and basal cultures were treated with vehicle. After 24h in culture, nitrite production was determined in the supernatant of glial cells using the Griess reaction. Values presented represent the mean ± SD from triplicate determinations, repeated at least three times. Statistical analysis revealed significant differences: *p ≤ 0.05, **p ≤ 0.01 compared to LPS-treated cultures; ^###^*p* ≤ 0.001 compared to basal cultures. Immunocytochemical analysis of the expression of pro-inflammatory factors in primary glial cultures is shown, cyclooxygenase 2 (COX-2, green) and tumor necrosis factor alpha (TNFα, red). Cell nuclei were stained with DAPI. Scale bar, 20 μM.

When we assessed the neuroprotective effect (Figure 5), we observed that compound **12d** also exhibited anti-inflammatory properties in this neuronal damage model, significantly reducing nitrite levels produced after injury. In neurodegenerative pathologies, glial cells are the main inducers of inflammatory processes; upon activation, they produce pro-inflammatory factors that contribute to neuronal death and disease progression. In a subsequent analysis, we used primary cultures of astrocytes (Figure 6A) and microglia (Figure 6B) isolated from the murine cerebral cortex. The results presented in Figure 6 demonstrate that compound **12d**, at a concentration of 5 μM, exerts a considerable anti-inflammatory effect. This is evidenced by the reduced nitrite levels in culture and the marked decrease in the production of pro-inflammatory agents such as cyclooxygenase-2 (COX-2) and tumor necrosis factor-α (TNFα) compared to LPS-stimulated glial cultures.

### Pharmacokinetics of 12d

The resulting plasma PK parameters of **12d** are shown in Table 3.

**Table 3:** Basic PK Parameters were Calculated.^a^

Pharmacokinetic parameters of **12d** in plasma	
*t*_1/2β_ (min)	176 ± 36.2
*T*_max_ (min)	15 ± 0
*C*_max_ (μg/mL)	2.61 ± 0.64
AUC_0-_^1440^ (μg/mL*min)	197.43 ± 16.58
AUC_0-_^∞^ (μg/mL*min)	199.23 ± 16.25
Cl (mg/kg)/(μg/mL)/min	0.15 ± 0.01
Vd (mg/kg)/(μg/mL)	38.24 ± 8.25

Pharmacokinetic parameters of **12d** in brain	
*t*_1/2β_ (min)	252 ± 74.7
*T*_max_ (min)	15 ± 0
*C*_max_ (μg/mL)	3.19 ± 1.01
AUC_0-_^1440^ (μg/mL*min)	117.62 ± 34.51
AUC_0-_^∞^ (μg/mL*min)	118.83 ± 35.02

a *C*_max_: maximum observed concentration, *T*_max_: time of maximum observed concentration, *t*_1/2β_: terminal elimination half-life, AUC_0-_^1440^: area under the curve from zero to the last sampling time, AUC_0-_^∞^: area under the curve from zero extrapolated to infinity, CI: plasma clearance, Vd: volume of distribution.

The maximum mean concentration found in plasma (2.61 ± 0.64 μg/mL) was reached at 15 min after the drug administration in a single oral dose of 30 mg/kg. Compound **12d** is found in plasma after 5 min of administration and rapidly disappears from the plasma, due to its high distribution throughout the organism. The AUC levels between the last sampling and infinite were very similar to each other. Notwithstanding plasma levels do not remain very high during the sampling period, it should be noted that the administration of **12d** in drinking water at a dose of 5 mg/kg to the murine model (SAMP8) continued at effective levels for 4 weeks. These efficacy results could also be explained by the elimination half-life (176 ± 36.2 min) which will allow reaching effective levels in the brain during treatment.

In the PK study, the quantitation in mouse brain samples was assessed, and after a single oral administration of 30 mg/kg, a maximum concentration of 3.19 ± 1.01 μg/mL was detected at 15 min (*C*_max_). From this moment on, concentrations decreased significantly but remained detectable throughout the sampling interval. These values support the presumed high distribution of **12d** throughout the body and its rapid elimination at a single dose as well as the levels sustained during administration through water.

For details on the method validation for quantification of **12d** in mouse plasma and in mouse brain (Supporting Information pages S87–S93).

### Selectivity

In an Alzheimer’s MoA panel (Eurofins)^37^ with 41 assays, including neurotransmitters, glutamate excitotoxicity, bioprocess of β-amyloid, tau hyperphosphorylation, and neuroinflammation, compound **12d** did not give significant results on the interaction with the targets included (Table S40).

### Hypothermic Effects of 12d in Mice

**Figure 7:**
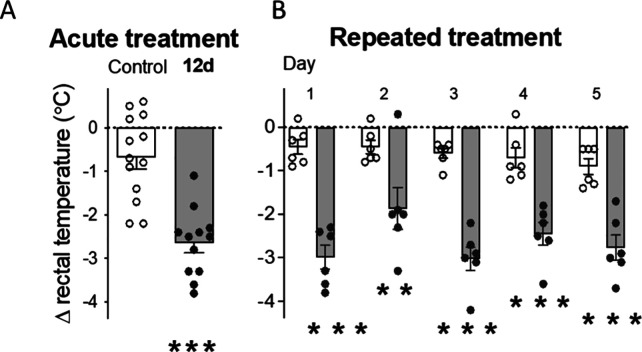
Acute and repeated effects of compound **12d** at core body temperature in mice. (A) The hypothermic effect was induced by acute treatment with **12d** (20 mg/kg, i.p., 1 h). Columns are means ± SEM of the difference in body temperature (°C) (Δ, 1 h minus basal value) for **12d**-treated mice (*n* = 12) and vehicle-treated control mice (*n* = 13). Symbols represent individual values for each mouse. ***p < 0.001 vs control group (Student’s *t* test, *t* = 5.588, *df* = 23). (B) Hypothermic effects observed during the repeated treatment with **12d** (20 mg/kg, i.p., 5 days). Columns are means ± SEM of the daily difference in body temperature (°C) (Δ, 1 h minus basal value) for **12d**-treated mice (*n* = 6) and vehicle-treated control mice (*n* = 6). **p < 0.01 or ***p < 0.001 vs control group (two-way repeated measures ANOVA followed by Sidak’s multiple comparisons test).

It is well precedented that several I_2_–IRs ligands induce hypothermia in rodents.^3,19,24,26,38^ Similar to what was recently reported for B06 (**9**),^26^ acute **12d** (20 mg/kg) induced hypothermia in adult CD1 mice (ranging from −1.1 to −3.8 °C in temperature drop) as measured 1 h postinjection (Figure 7A). Repeated administration of **12d** (20 mg/kg, 5 days) showed persistent hypothermic effects in mice, observed consistently from days 1 to 5 of treatment (Figure 7B), and contrarily to what was previously observed for other I_2_–IRs ligands, that developed tolerance to the acute hypothermic effects.^19,20,26^ As mentioned in our prior publications,^19,20,24,26,39^ the hypothermic effects induced by **12d** might be mediated and/or related to the neuroprotective potential of I_2_–IRs ligands. In this context, prior data suggested a putative role of hypothermia in mediating neuroprotection, such as in cerebral ischemia,^40^ or following stroke or brain injury, proposing a way to improve the neurological outcome under various pathological conditions in the clinic,^41,42^ and presenting these novel I_2_–IRs ligands as good therapeutic candidates.

### Neurochemical Effects of 12d in Mice Hippocampus

**Figure 8:**
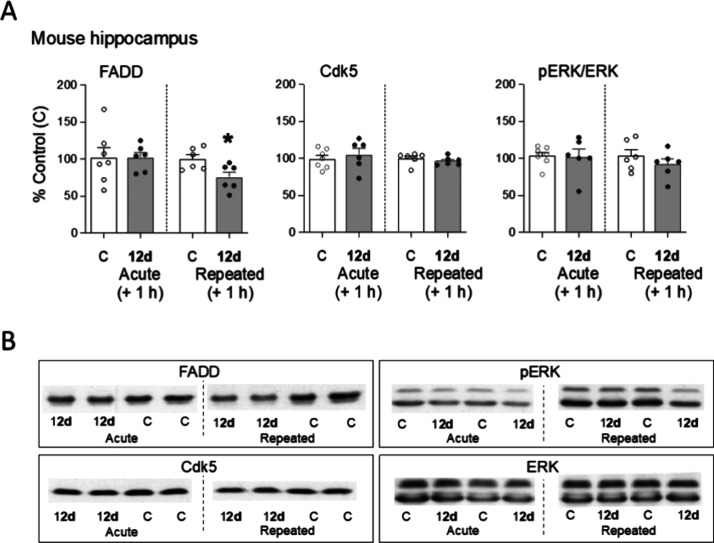
Acute and repeated effects of compound **12d** on the content of hippocampal neurochemical markers (FADD, Cdk5, pERK/ERK) in mice. (A) Acute (20 mg/kg, i.p., 1 h, *n* = 6) and repeated (20 mg/kg, i.p., 5 days, *n* = 6) effects induced by **12d**. Columns are means ± SEM of the target protein (% Control (C), *n* = 7–6). Symbols represent individual values for each mouse. *p = 0.0025 vs respective C group (Student’s *t*-test, *t* = 2.644, *df* = 10). (B) Representative immunoblots depicting the labeling of FADD, Cdk5 and pERK/ERK for each treatment group.

We have recently shown that the acute administration of some I_2_–IRs ligands to rodents, besides inducing hypothermia, decreased the brain content of FADD multifunctional protein.^19,20,26^ As previously described, FADD is an adaptor of cell death receptors with a dual role in the brain, since it can mediate both pro- and antiapoptotic/neuroprotective actions in rodents.^43^ The present results failed to show an acute regulation of FADD by treatment with compound **12d**, but revealed a 25% decrease in hippocampal FADD content following the repeated drug paradigm (Figure 8). This significant decrease, together with the still present hypothermic effect observed at this time point (Figure 7B), suggests that a more prolonged treatment might be needed for this compound to mediate some of its neuroplastic or neuroprotective actions through the regulation of this key brain marker, in contrast to the acute effects observed for other I_2_–IRs compounds.^19,20,26^ In this line of thought, we evaluated other putative neurochemical markers that could parallel the effects observed for **12d** and that have been previously shown to be regulated in conjunction with FADD, such as Cdk5 and pERK/ERK.^19,44^ However, the results, as reported in Figure 11, showed no changes in the protein content of Cdk5 or pERK/ERK by **12d**, reinforcing the particular role of FADD in the effects mediated by I_2_–IRs ligands and in parallel to their induction of hypothermia.

### Cognitive Effects in the *In Vivo* Senescence Accelerated Prone 8 (SAMP8) Mice Treated with 12d

**Figure 9:**
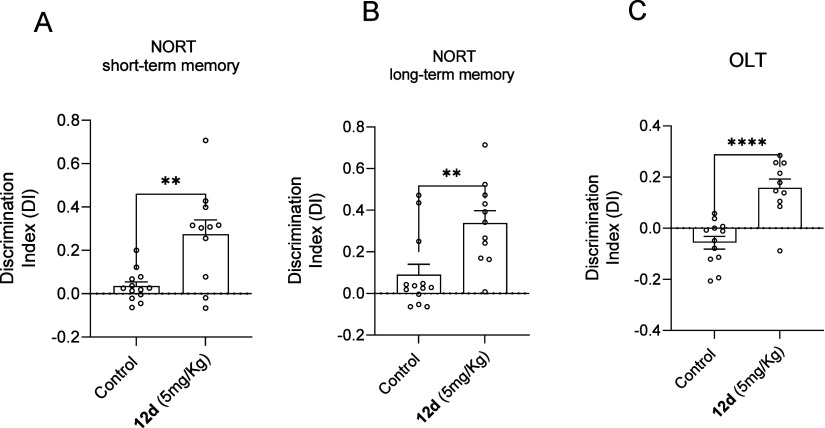
Results obtained from NORT, both short- (A) and long-term memory (B), and OLT (C) evaluations in SAMP8. Control mice group and SAMP8 treated with the compound **12d** mice group represented as the summary of DI. Values represented are mean ± Standard error of the mean (SEM); (*n* = 8 per group); *p < 0.05; **p < 0.01; ***p < 0.001; ****p < 0.0001.

The SAMP8 mouse model is a well-characterized inbred selected strain to show AD pathological characteristics fully present at 5–6 months of age, including cognitive and behavioral deficits such as memory impairment.^45−47^ Thus, at the selected age of 10 months, SAMP8 mice provide a severe AD pathological landscape suitable for evaluating the drug effects. The treatment with compound **12d** (5 mg/kg for 4 weeks, p. o.) improved cognitive parameters measured through Novel Object Recognition and Novel Object Location Tests (NORT and OLT, respectively). NORT short- and long-term memory showed a significant Discrimination Index (DI) increase indicating an amelioration of working memory. Moreover, spatial memory determined by OLT was also significantly improved in SAMP8 (Figure 9). These results agreed with those previously published with other I_2_–IRs ligands in SAMP8 and in 5XFAD, a mice model of familial AD.^20,21,23,27^

### Molecular Mechanisms Driving 12d Treatment Neuroprotection in SAMP8Mice

Ca2+/calmodulin-dependent cyclic nucleotide phosphodiesterase (PDE1) maintains the homeostasis of 3′,5′-cyclic adenosine monophosphate (cAMP) and 3′,5′-cyclic guanosine monophosphate (cGMP) in the brain, two key second messengers that regulate a broad range of intracellular processes and neurocognitive functions, specifically memory and cognition, associated with AD.^48^ Ca2+/calmodulin-dependent protein kinase II (CaM kinase II or CaMKII) is also regulated by the Ca2+/calmodulin complex, also involved in many signaling cascades, and is thought to be an important mediator of learning and memory.^49^

**Figure 10:**
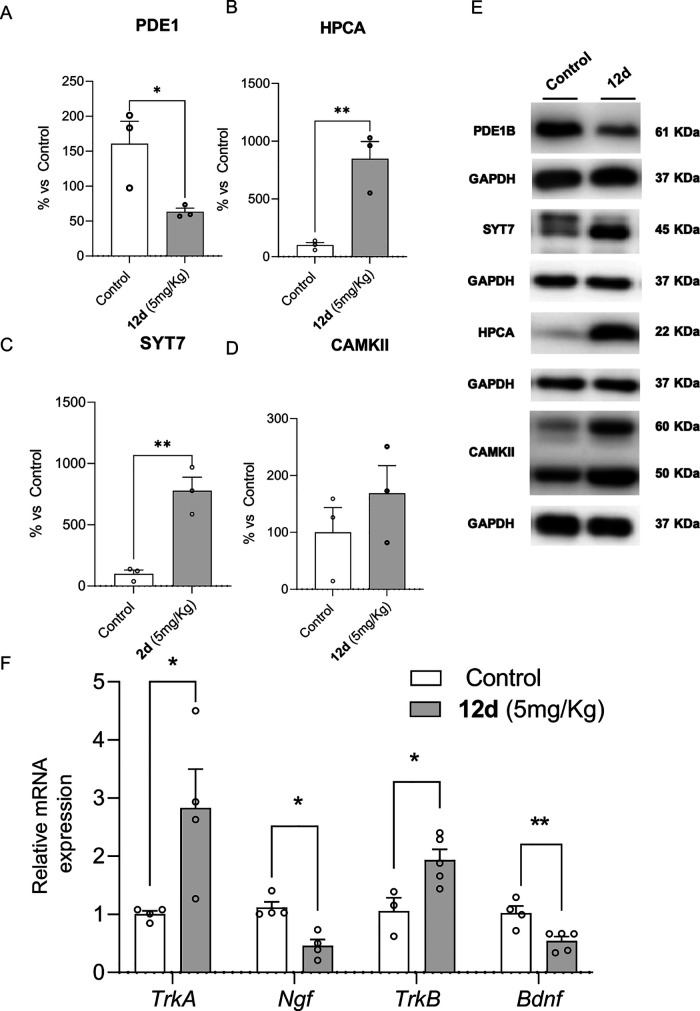
Representative Western blots and quantifications for PDE1B, HPCA, SYT7, and CAMKII (A – E). Values in bar graphs are adjusted to 100% for protein levels of the SAMP8 Control group. Representative gene expression for *TrkA, Ngf, TrkB*, and *Bdnf* (F). Gene expression levels were determined by real-time PCR. Values represented are mean ± Standard error of the mean (SEM); (*n* = 3–5 per group); *p < 0.05; **p < 0.01.

**12d** was able to reduce the protein content in the hippocampus of SAMP8, pointing out to be responsible for cognitive improvement shown after **12d** treatment (Figure 10 A, D, E). In fact, PDE1 and CaMKII inhibitors gained interest in the treatment of neurodegenerative diseases.^50^ In addition, **12d** treatment increased the protein levels of hippocalcin (HPCA), which is involved in long-term depression in hippocampal neurons and required for normal spatial learning (Figure 10 B, E).^51^ Of note, HPCA prevented Amyloid-beta toxicity.^52^**12d** also increases protein levels of synaptotagmin 7 (SYT7) (Figure 10 C, E) calcium sensor involved in neuronal membrane trafficking, a transcendental process in neurodegenerative diseases with high synaptic vulnerability, accordingly we found a sparse increase in postsynaptic density 95 (PSD95) and Synaptophysin albeit did not reach significance (data not shown). In this string of evidence, neurotrophic response elements were also modulated by **12d** treatment in SAMP8. Gene expression for Tropomyosin receptor kinase A (*TrkA*) and B (*TrkB*) was significantly increased, and, in parallel, a reduction in Neural growth factor (*Ngf*) and Brain-derived neurotrophic factor (*Bdnf*), its respective ligands, gene expression after 4 weeks of treatment in SAMP8 hippocampus (Figure 10F) demonstrating the neuroprotective effect of **12d** in a mice model of cognitive decline and indicating a putative role as a new therapeutic approach for neurodegenerative conditions.

### Antiallodynic Effects of 12d in Capsaicin-Induced Mechanical Hypersensitivity Model and the Rotarod Test

Besides to its role in neurodegenerative diseases, I_2_–IRs are well-known by their analgesic properties. Indeed, CR4056 (**1**) is an effective analgesic against knee osteoarthritis.^13^ Therefore, in addition to the above-mentioned studies related with neuroprotection/neurodegeneration, we next explored the ability of **12d** for reducing pain in a murine model.

The enhanced pain sensitivity in the area surrounding capsaicin injection is known to result from central sensitization, which is a process that plays a pivotal role in chronic pain development.^53^ In fact, capsaicin-induced mechanical hypersensitivity has been used to study drug effects in central sensitization not only in rodents,^54,55^ but also in humans.^56^

We first tested the effect of CR4056 (**1**), a prototypical I_2_–IRs agonist, in capsaicin-induced mechanical hypersensitivity (allodynia) in mice. Nonsensitized mice showed a response latency to the mechanical stimulation of approximately 45 s. This response latency markedly decreased in mice intraplantarly treated with capsaicin, denoting the development of tactile allodynia (Figure S13A). CR4056 (**1**) (1.25–10 mg/kg, p.o.) induced a dose-dependent and nearly full reversal of capsaicin-induced allodynia (Figure S13A). To our knowledge, this is the first report on the antiallodynic effects of an I_2_–IRs agonist on this behavioral model of central sensitization. Then, we tested the effects of the association of CR4056 (**1**) with idazoxan (**4**) (3 mg/kg, s.c.), a well-known (albeit nonselective) I_2_–IRs antagonist.^57^ The administration of this latter compound fully suppressed the antiallodynic effect of CR4056 (**1**) (Figure S13B). Therefore, these results suggest that the antiallodynic effect of CR4056 (**1**) is mediated through I_2_–IRs agonism.

**Figure 11:**
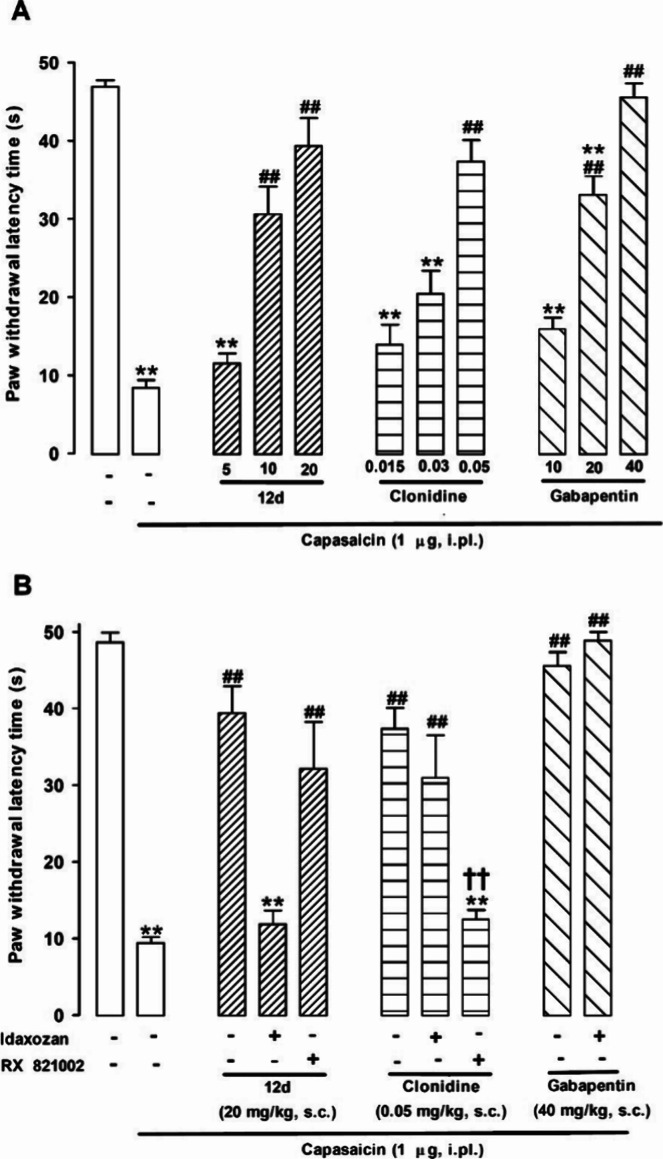
Reduction of capsaicin-induced mechanical hypersensitivity by the administration of **12d**, clonidine and gabapentin in mice. (A) Dose dependency of the antinociceptive effects induced by the subcutaneous (s.c.) administration of **12d**, clonidine and gabapentin. (B) Effects of **12d**, clonidine or gabapentin alone and associated with the administration of the I_2_–IRs antagonist idazoxan (**4**) or its methoxy analog RX 821002 (both at 3 mg/kg, s.c.). Values are the mean ± SEM obtained from 7–9 animals per group: **p < 0.01 vs nonsensitized animals treated with the vehicle of the drugs tested; ## *p* < 0.01 vs capsaicin-injected mice treated with the vehicle of the drugs tested; †† *p* < 0.01 **12d**-treated animals associated with idazoxan (**4**) or its solvent, and clonidine-treated animals associated with RX 821002 or its solvent (one-way ANOVA followed by Bonferroni test).

We then tested the effect of **12d** (5–20 mg/kg, s.c.), clonidine (0.05 mg/kg, s.c.) and gabapentin (10–40 mg/kg, s.c.). Both clonidine and gabapentin have known analgesic effects in human patients,^58,59^ and have been previously reported to decrease capsaicin-induced mechanical hypersensitivity in mice.^55,58^ However, mechanisms of their analgesic effects differ, since whereas clonidine is a prototypical α_2_-ARs agonist, with low affinity for imidazoline receptors,^60^ gabapentin acts through the modulation of α_2_δ auxiliary subunits of calcium channels^61^ (i.e., not related to actions on adrenergic or imidazoline receptors). We show here that not only clonidine and gabapentin, but also **12d**, induced dose-dependent and full antiallodynic effects (Figure 11A). We also evaluated the *in vivo* effects of the association of **12d** and clonidine with idazoxan (**4**) and its methoxy analog RX 821002 (both at 3 mg/kg, s.c.). Whereas idazoxan (**4**) is known to be an antagonist at both I_2_–IRs and α_2_-AR, RX 821002 is a selective α_2_-AR antagonist without affinity for imidazoline receptors.^62^ We selected doses of **12d** and clonidine that induced the maximum antiallodynic effect (i.e., **12d** 20 mg/kg and clonidine 0.05 mg/kg). The administration of idazoxan (**4**) fully reversed the antiallodynic effect of **12d**, without modifying the effect induced by clonidine, suggesting that idazoxan (**4**) (in our experimental conditions) is acting primarily through I_2_–IRs but not α_2_-ARs. Conversely, RX 821002 completely reversed the effect of clonidine (as expected), but without significantly modifying the effect of **12d** (Figure 11B). Altogether, these results suggest that **12d** induces its antiallodynic effect through I_2_–IRs agonism without the participation of α_2_-AR. Finally, we also found that idazoxan (**4**) was unable to modify the antiallodynic effect of gabapentin (40 mg/kg) (Figure 14B). These latest results further validate the specificity of idazoxan in reversing only the effect of I_2_-ARs agonism.

**Figure 12:**
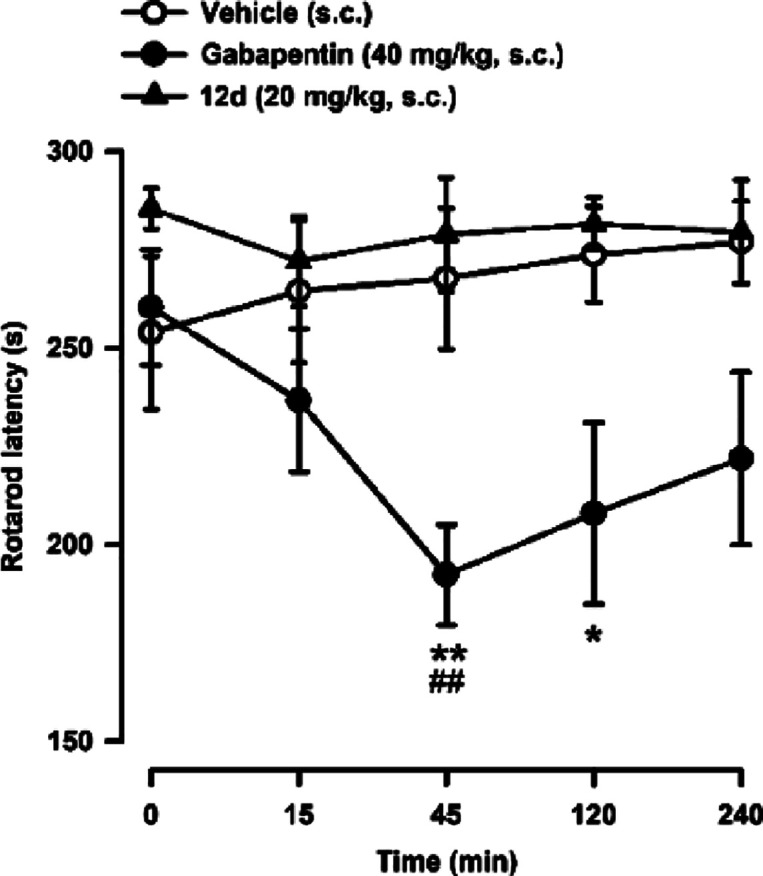
Effect of **12d** and gabapentin on motor coordination. The latency to fall-down from the rotarod was recorded in each mouse immediately before (time 0) and at several times after the subcutaneous (s.c.) administration of **12d** (20 mg/kg), gabapentin (40 mg/kg) or their vehicle. Values are the mean ± SEM from 8 animals: ∗*p* < 0.05, ∗∗*p* < 0.01 between the values at time 0 and after drug administration; ##*p* < 0.01 between gabapentin- and vehicle-treated groups on the same time after treatment (2-way repeated measures ANOVA followed by Bonferroni test).

The rotarod test is a standard test to assess motor coordination in rodents. Drugs that affect rotarod latency are thought to impair motor coordination.^63^ We tested animals treated with **12d** and gabapentin in rotarod performance, at doses able to reverse approximately the same degree of mechanical hypersensitivity (20 and 40 mg/kg, respectively). Administration of the vehicle of the drugs did not alter rotarod latency at any time point tested during the 4 h evaluation period, in comparison to the baseline value (time 0 in Figure 12). Similarly, animals administered with compound **12d** showed no change in the latency to fall in comparison to the baseline value or to vehicle-treated mice, at any time point tested (Figure 12). Assessment of drug-induced motor functioning is relevant for the interpretation of the results from tests of nociception, as nonanalgesic treatments affecting motor coordination might attenuate nociceptive responses (which are motor responses) inducing false analgesic-like effects.^63^ Therefore, our results on the effects of **12d** on capsaicin-induced mechanical hypersensitivity cannot be attributed to motor impairment.

In contrast to the effects of **12d**, gabapentin induced a marked decrease in the rotarod latency which peaked 45 min after its administration but that was still observable even 2 h after its administration (Figure 12). These data agree with previous rodent studies showing that gabapentin and other gabapentinoids have an impact on rotarod performance,^64,65^ but also with the clinical effects of this type of drugs on human patients, since it is well-known that the most frequent side effects of gabapentinoids include fatigue, dizziness, sedation, somnolence, and ataxia. These effects occur in the range of the analgesic effects and have a clinical relevance limiting the use of the gabapentinoids.^58^

## Conclusions

Following with our interest in the description of new I_2_–IRs ligands, we have accessed to a family of (3-phenylcarbamoyl-3,4-dihydro-2*H*-pyrrol-2-yl)phosphonates from previously reported bicyclic iminophosphonates. The evaluation of the affinity for I_2_–IRs in human tissues by a competition binding assay using radiolabeled compounds, led to the selection of representative **12d** to continue with its pharmacological characterization. Compound **12d** was endowed with optimal ADME parameters and showed a neuroprotective role on the human cell line SH-SY5Y and an anti-inflammatory activity in primary cultures of astrocytes and microglia treated with LPS. After the determination of its pharmacokinetic profile, the treatment of mice with **12d** confirmed a hypothermic effect and a decrease in the content of FADD protein, a key signaling mediator of neuroprotective actions. The I_2_–IRs also fostered an improvement in the condition, spatial memory and working memory, of the murine SAMP8 model by a modification in the Ca2+/calmodulin-dependent protein kinase II. Finally, compound **12d** induced antiallodynic effects attributable to I_2_–IRs agonism in a behavioral model of central sensitization, and at doses not affecting motor coordination, unlike gabapentin, which induced a prominent motor impairment at therapeutic doses.

To sum up, we have delivered a new pharmacological tool for characterizing the physiopatological implications of I_2_–IRs. This new I_2_–IRs ligand, with an unprecedent structure and endowed with optimal *in vivo* properties may be progressed toward early preclinical phases, either in neurodegenerative diseases or as analgesic.

## Experimental Section

### Chemistry

#### General Information

Reagents, solvents and starting products were acquired from commercial sources. The term “concentration” refers to the vacuum evaporation using a Büchi rotavapor. When indicated, the reaction products were purified by “flash” chromatography on silica gel (35–70 μm) with the indicated solvent system. The melting points were measured in a MFB 59510 M Gallenkamp instruments. IR spectra were performed in a spectrophotometer Nicolet Avantar 320 FTR-IR or in a Spectrum Two FT-IR Spectrometer, and only noteworthy IR absorptions (cm^–1^) are listed. NMR spectra were recorded in CDCl_3_ at 400 MHz (^1^H) and 100.6 MHz (^13^C), and chemical shifts are reported in δ values downfield from TMS or relative to residual chloroform (7.26 ppm, 77.0 ppm) as an internal standard. Data are reported in the following manner: chemical shift, multiplicity, coupling constant (*J*) in hertz (Hz), integrated intensity and assignment (when possible). The coupling constants (*J*) described in the ^13^C spectra referred to the carbon-phosphor couplings (J_CP_). Multiplicities are reported using the following abbreviations: s, singlet; d, doublet; dd, doublet of doublets; ddd, double double of doublets; dq, doble quadruplet; t, triplet; quin, quintet; m, multiplet; br s, broad signal, app, apparent. Assignments and stereochemical determinations are given only when they are derived from definitive two-dimensional NMR experiments (g-HSQC–COSY). Mass Spectrometry was performed using LC/MSD TOF Agilent Technologies G1969A spectrometer by the Mass Spectrometry for Molecular Characterization Unit (Biomolecular Analysis Unit), from Scientific and Technological Centers (CCiTUB), Universitat de Barcelona. The elemental analyses were carried out in a Flash 1112 series Thermofinnigan elemental microanalyzator (A5) to determine C, H, and N. HPLC-MS (Agilent 1260 Infinity II) analysis was conducted on an Poroshell 120 EC-C15 (4.6 mm x50 mm, 2.7 μm) at 40 °C. Mobile phase (A: H_2_0 + 0.05% formic acid and B: ACN + 0.05% formic acid) using a gradient elution. Flow rate 0.6 mL/min. The DAD detector was set at 254 nm and the injection volume was 5 μL and oven temperature 40 °C. All compounds are >95% pure by HPLC.

#### General Procedure for the Opening of the Imide Ring in Basic Media

A solution of bicyclic α-iminophosphonates (**11a**–**11n**) in 0.05 M NaOH in THF/H_2_O 2:1 was stirred at room temperature for 2.5 h. The reaction mixture was concentrated *in vacuo*, water (2 mL) was added, and the pH of the mixture was acidified (pH = 3) with a 1 M solution of HCl. The reaction mixture was extracted with EtOAc, the organic phases were combined, dried over Na_2_SO_4_, filtered, and concentrated to give a residue that was purified by column chromatography to afford pure product **12a**–**12o**.

#### Diethyl [(2*RS*,3*RS*)-3-(Phenylcarbamoyl)-3,4-dihydro-2*H*-pyrrol-2-yl]phosphonate (12a)

Following the general procedure, **11a** (100 mg, 0.28 mmol) and a solution of 0.05 M NaOH in THF/H_2_O 2:1 (7 mL) gave **12a** (47 mg, 51%) as a white solid, after column chromatography (CH_2_Cl_2_/MeOH 97.5:2.5). An analytical sample was recrystallized in EtOAc. Mp 123–125 °C (EtOAc). IR (NaCl) 3265, 2983, 1686, 1601, 1444, 1232, 1028, 755, 693 cm^–1^. ^1^H NMR (400 MHz, CDCl_3_) δ 1.41 (dt, *J* = 15.4, 7.0 Hz, 6H, OCH_2_C*H*_3_), 2.90 (ddd, *J* = 18.4, 10.4, 3.5 Hz, 1H, H-4), 3.31–3.39 (m, 1H, H-4), 3.41–3.54 (ddt, *J* = 20.9, 10.4, 8.0 Hz, 1H, H-3), 4.18–4.34 (m, 4H, OC*H*_2_CH_3_), 4.45–4.53 (ddt, *J* = 17.4, 8.0, 2.7 Hz, 1H, H-2), 7.06–7.10 (tt, *J* = 7.3, 1.2 Hz, 1H, ArH), 7.27–7.33 (tt, 2H, *J* = 7.4 Hz, 2.0 Hz, ArH), 7.60 (d, *J* = 7.1 Hz, 2H, ArH), 7.65 (t, *J* = 2.6 Hz, 1H, H-5), 9.53 (br s, 1H, NH). ^13^C NMR (101 MHz, CDCl_3_) δ 16.4 (t, *J* = 4.9 Hz, OCH_2_*C*H_3_), 40.1 (C-4), 43.3 (C-3), 63.4 (dd, *J* = 7.4, 3.5 Hz, O*C*H_2_CH_3_), 72.3 (d, *J* = 162.0 Hz, C-2), 119.5 (2CHAr), 123.9 (CHAr), 128.8 (2CHAr), 138.5 (C*-ipso*), 169.0 (d, *J* = 16.5 Hz, C-5), 169.1 (CO). HRMS C_15_H_22_N_2_O_4_P [M + H]^+^ 325.1308; found, 325.1312. Anal. Cald. for C_15_H_21_N_2_O_4_P·1/4H_2_O: C, 54.79; H, 6.59; N, 8.52; found, C,54.60; H, 6.54; N, 8.36%.

#### Diethyl (2*RS*,3*RS*)- 3-[(3-Chloro-4-fluorophenylcarbamoyl)-3,4-dihydro-2*H*-pyrrol-2-yl]phosphonate (12b)

Following the general procedure, **11b** (210 mg, 0.52 mmol) and 0.05 M NaOH in THF/H_2_O 2:1 (13 mL) gave **12b** (86.8 mg, 44%) as a white solid after column chromatography (CH_2_Cl_2_/MeOH, 97.5:2.5). An analytical sample was recrystallized in EtOAc. Mp 120–123 °C (EtOAc). IR (NaCl) 3268, 2986, 1689, 1501, 1220, 1054, 1028, 973 cm^–1^. ^1^H NMR (400 MHz, CDCl_3_) δ 1.42 (dt, *J* = 23.9, 7.0 Hz, 6H, OCH_2_C*H*_3_), 2.93 (dd, *J* = 18.0, 9.5 Hz, 1H, H-4), 3.32–3.39 (m, 2H, H-4), 3.40–3.48 (ddt, *J* = 20.9, 10.4, 8.2 Hz, 1H, H-3), 4.13–4.35 (m, 4H, OC*H*_2_CH_3_), 4.46–4.35 (m, 1H, H-2), 7.07 (t, *J* = 8.8 Hz, 1H, ArH), 7.42 (ddd, *J* = 9.0, 4.3, 2.5 Hz, 1H, ArH), 7.66 (t, *J* = 2.9 Hz, 1H, H-5), 7.77 (dd, *J* = 6.6, 2.6 Hz, 1H, ArH), 9.77 (br s, 1H, NH). ^13^C NMR (101 MHz, CDCl_3_) δ 16.4 (t, *J* = 4.6 Hz, OCH_2_*C*H_3_), 39.6 (d, *J* = 5.9 Hz, C-4), 43.3 (C-3), 63.4 (dd, *J* = 13.4, 6.6 Hz, O*C*H_2_CH_3_), 72.3 (d, *J* = 162.6 Hz, C-2), 116.4 (d, *J* = 22.0 Hz, CHAr), 119.0 (d, *J* = 7.0 Hz, CHAr), 121.0 (d, *J* = 18.0 Hz, C-*ipso*), 121.5 (CHAr), 135.2 (d, *J* = 3.0 Hz, C-*ipso*), 154.5 (d, *J* = 245.0 Hz, C-*ipso*), 168.0 (CO), 169.0 (d, *J* = 12.5 Hz, C-5). HRMS C_15_H_20_ClFN_2_O_4_P [M + H]^+^ 377.0836; found, 377.0828. Purity 100% (t_R_ = 4.153 min).

#### Diethyl [(2*RS*,3*RS*)-2-Phenyl-3-(phenylcarbamoyl)-3,4-dihydro-2*H*-pyrrol-2-yl]phosphonate (12c)

Following the general procedure, **11c** (144 mg, 0.34 mmol) and 0.05 M NaOH in THF/H_2_O 2:1 (8 mL) gave **12c** (94 mg, 70%) as a white solid after column chromatography (EtOAc/MeOH, 95:5). An analytical sample was recrystallized in EtOAc. Mp 117–122 °C (EtOAc). IR (NaCl) 3267, 2981, 1692, 1600, 1548, 1443, 1225, 1028, 968, 755 cm^–1^. ^1^H NMR (400 MHz, CDCl_3_) δ 1.08 (t, *J* = 7.1 Hz, 3H, CH_2_C*H*_3_), 1.36 (t, *J* = 6.9 Hz, 3H, CH_2_C*H*_3_), 2.87 (ddt, *J* = 18.4, 9.5, 1.2 Hz, 1H, H-4), 3.32 (dddd, *J* = 18.4, 8.2, 6.3, 1.0 Hz, 1H, H-4), 3.79–3.94 (complex signal, 2H, H-3 and C*H*_2_CH_3_), 4.06–4.15 (m, 1H, C*H*_2_CH_3_), 4.16–4.30 (m, 2H, C*H*_2_CH_3_), 7.04 (tt, *J* = 7.4, 1.3 Hz, 1H, ArH), 7.17–7.24 (m, 5H, ArH), 7.28–7.31 (m, 2H, ArH), 7.51–7.54 (m, 2H, ArH), 8.03 (dt, *J* = 3.8, 1.2 Hz, H-5), 9.24(br s, 1H, NH). ^13^C NMR (101 MHz, CDCl_3_) δ 16.0 (d, *J* = 5.8 Hz, CH_2_*C*H_3_), 16.4 (d, *J* = 5.8 Hz, CH_2_*C*H_3_), 39.5 (d, *J* = 5.0 Hz, C-4), 50.0 (d, *J* = 3.0 Hz, C-3), 63.5 (d, *J* = 8.0 Hz, *C*H_2_CH_3_), 64.8 (d, *J* = 7.5 Hz, *C*H_2_CH_3_), 83.9 (d, *J* = 162.2 Hz, C-2), 119.8 (2CHAr), 123.9 (CHAr), 127.2 (d, *J* = 7.2 Hz, 2 CHAr), 128.0 (d, *J* = 0.8 Hz, 2CHAr), 128.1 (d, *J* = 1.6 Hz, CHAr) 128.7 (2CHAr), 133.6 (C-*ipso*), 138.0 (C-*ipso*), 166.8 (d, *J* = 4.1 Hz, CO), 169.2 (d, *J* = 15.0 Hz, C-5). HRMS C_21_H_26_N_2_O_4_P [M + H]^+^ 401.1626; found, 401.1625. Anal. Cald. for C_21_H_25_N_2_O_4_P: C, 62.99; H, 6.29; N, 7.00; found, C, 63.76; H, 6.31; N, 6.55%. Purity 98.2% (t_R_ = 4.176 min).

#### Diethyl [(2*RS*,3*RS*)-3-((3-Chloro-4-fluorophenyl)carbamoyl)-2-phenyl-3,4-dihydro-2*H*-pyrrol-2-yl]phosphonate (12d)

Following the general procedure, **9** (99.3 mg, 0.21 mmol) and 0.05 M NaOH in THF/H_2_O 2:1 (5 mL) gave **12d** (71 mg, 76%) as a white solid after column chromatography (EtOAc/MeOH, 95:5). An analytical sample was recrystallized in EtOAc. Mp 136–138 °C (EtOAc). IR (NaCl) 3266, 2923, 2853, 1694, 1500, 1220, 1056, 1029, 971 cm^–1^. ^1^H NMR (400 MHz, CDCl_3_) δ 1.07 (td, *J* = 7.1, 0.8 Hz, 3H, CH_2_C*H*_3_), 1.36 (t, *J* = 7.0 Hz, 3H, CH_2_C*H*_3_), 2.84–2.92 (ddt, *J* = 18.5, 9.4, 1.6 Hz, 1H, H-4), 3.30 (dddd, *J* = 18.5, 7.5, 6.5, 1.0 Hz, 1H, H-4), 3.81–3.93 (complex signal, 2H, H-3 and C*H*_2_CH_3_), 4.03–4.11 (m, 1H, C*H*_2_CH_3_), 4.13–4.28 (m, 2H, C*H*_2_CH_3_), 6.97 (t, *J* = 8.8 Hz, 1H, ArH), 7.13 (ddd, *J* = 8.5, 4.0, 2.5 Hz, 1H, ArH), 7.18–7.21 (m, 3H, ArH), 7.40 (dd, *J* = 6.6, 2.6 Hz, 1H ArH), 7.51–7.54 (m, 2H, ArH), 8.03 (dt, *J* = 4.0, 1.2 Hz, 1H, H-5), 9.57 (br s, 1H, NH). ^13^C NMR (101 MHz, CDCl_3_) δ 16.0 (d, *J* = 5.8 Hz, CH_2_*C*H_3_), 16.4 (d, *J* = 5.7 Hz, CH_2_*C*H_3_), 39.9 (d, *J* = 4.1 Hz, C-4), 49.7 (d, *J* = 3.0 Hz, C-3), 63.6 (d, *J* = 8.1 Hz, *C*H_2_CH_3_), 65.0 (d, *J* = 7.6 Hz, *C*H_2_CH_3_), 84.3 (d, *J* = 160.9 Hz, C-2), 116.3 (d, *J* = 22.1 Hz, CHAr), 119.4 (d, *J* = 6.8 Hz, CHAr), 120.7 (d, *J* = 18.5 Hz, C-*ipso*), 121.8 (CHAr), 127.2 (d, *J* = 7.0 Hz, 2CHAr), 128.2 (2CHAr), 128.3 (CHAr), 133.6 (C-*ipso*), 134.7 (d, *J* = 3.2 Hz, C-*ipso*), 155.7(d, *J* = 246.3 Hz, C-*ipso*), 167.4 (d, *J* = 5.1 Hz, CO), 169.3 (d, *J* = 14.7 Hz, C-5). HRMS C_21_H_24_ClFN_2_O_4_P [M + H]^+^ 453.1143; found, 453.1141. Anal. Cald. for C_21_H_23_ClN_2_O_4_P: C, 55.70; H, 5.12; N, 6.19; found, C, 55.82; H, 5.09; N, 5.93%. Purity 99.3% (t_R_ = 4.496 min).

#### Diethyl [(2*RS*,3*RS*)-3-((4-Chlorophenyl)carbamoyl)-2-phenyl-3,4-dihydro-2*H*-pyrrol-2-yl]phosphonate (12e)

Following the general procedure, **11e** (100 mg, 0.22 mmol) and a solution of 0.05 M NaOH in THF/H_2_O 2:1 (7 mL) gave **12e** (47 mg, 50%) as a white solid, after column chromatography (CH_2_Cl_2_/MeOH, 97.5:2.5). An analytical sample was recrystallized in EtOAc. Mp 130–132 °C (EtOAc). IR (ATR) 3300, 3263, 2984, 1662, 1540, 1491, 1251, 1036, 977, 752, 704 cm^–1^. ^1^H NMR (400 MHz, CDCl_3_) δ 1.09 (td, *J* = 7.1, 0.7 Hz, 3H, OCH_2_C*H*_3_), 1.38 (td, *J* = 7.1, 0.6 Hz, 3H, OCH_2_C*H*_3_), 2.82–2.90 (dddd, *J* = 18.5, 9.6, 1.4, 0.7 Hz 1H, H-4), 3,27–3.36 (dddd, *J* = 18.4, 8.7, 6.2 0.9 Hz, 1H, H-4), 3.75–3.84 (quin, *J* = 9.0 Hz, 1H, H-3), 3.88–3.98 (complex signal, 1H, OC*H*_2_CH_3_), 4.08–4.16 (m, 1H, OC*H*_2_CH_3_), 4.18–4.29 (m, 2H, OC*H*_2_CH_3_), 7.17–7.20 (m, 5H, ArH), 7.26–7.29 (m, 2H, ArH), 7.45–7.48 (m, 2H, ArH), 8.04 (d, *J* = 3.7 Hz, 1H, H-5), 9.37 (s, 1H, NH). ^13^C NMR (101 MHz, CDCl_3_) δ 16.2 (d, *J* = 5.8 Hz, OCH_2_*C*H_3_), 16.6 (d, *J* = 5.7 Hz, OCH_2_*C*H_3_), 39.3 (d, *J* = 5.5 Hz, C-4), 50.4 (d, *J* = 2.6 Hz, C-3), 63.8 (d, *J* = 8.0 Hz, O*C*H_2_CH_3_), 65.1 (d, *J* = 7.7 Hz, O*C*H_2_CH_3_), 83.9 (d, *J* = 163.2 Hz, C-2), 121.0 (2CHAr), 127.2 (d, *J* = 7.2 Hz, 2CHAr), 128.3 (d, *J* = 0.6 Hz, 2CHAr), 128.4 (d, *J* = 1.5 Hz, CHAr), 128.8 (2CHAr), 128.9 (C*-ipso*), 133.4 (C*-ipso*), 136,8 (C*-ipso*), 166.9 (d, *J* = 3.2 Hz, CO), 169.3 (d, *J* = 15.3 Hz, C-5). HRMS C_21_H_25_ClN_2_O_4_P [M + H]^+^ 435.1235; found, 435.1237. Anal. Cald. for C_21_H_24_ClN_2_O_4_P: C, 58.00; H, 5.56; N, 6.44; found, C, 58.45; H, 5.34; N, 6.35%. Purity 98.1% (t_R_ = 4.455 min).

#### Diethyl [(2*RS*,3*RS*)-3-((1,1′-Biphenyl)-4-ylcarbamoyl)-2-phenyl-3,4-dihydro-2*H*-pyrrol-2-yl]phosphonate (12f)

Following the general procedure, **11f** (100 mg, 0.20 mmol) and a solution of 0.05 M NaOH in THF/H_2_O 2:1 (7 mL) gave **12f** (47 mg, 50%) as a white solid, after column chromatography (EtOAc/hexane, 70:30). An analytical sample was recrystallized in EtOAc. Mp 194–195 °C (EtOAc). IR (ATR) 3248, 3058, 2923, 1686, 1537, 1485, 1222, 1027, 968, 764, 697 cm^–1^. ^1^H NMR (400 MHz, CDCl_3_) δ 1.09 (td, *J* = 7.1, 0.7 Hz, 3H, OCH_2_C*H*_3_), 1.39 (td, *J* = 7.0, 0.6 Hz, 3H, OCH_2_C*H*_3_), 2.85–2.92 (ddd, *J* = 18.5, 9.6, 0.8 Hz, 1H, H-4), 3.31–3.39 (dddd, *J* = 18.4, 8.8, 6.1, 0.8 Hz, 1H, H-4), 3.79–3.88 (quin, *J* = 9.3 Hz, 1H, H-3), 3.89–3.99 (m, 1H, OC*H*_2_CH_3_), 4.10–4.18 (m, 1H, OC*H*_2_CH_3_), 4.19–4.33 (m, 2H, OC*H*_2_CH_3_), 7.19–7.22 (m, 3H, ArH), 7.31 (tt, *J* = 7.4, 1.2 Hz, 1H, ArH), 7.38–7.43 (m, 4H, ArH), 7.48 (t, *J* = 2.1 Hz, 1H, ArH), 7.49–7.53 (m, 3H, ArH), 7.54–7.57 (m, 2H, ArH), 8.06 (dt, *J* = 3.6, 1.3 Hz, 1H, H-5), 9.30 (s, 1H, NH). ^13^C NMR (101 MHz, CDCl_3_) δ 16.2 (d, *J =* 5.7 Hz, OCH_2_*C*H_3_), 16.6 (d, *J =* 5.7 Hz, OCH_2_*C*H_3_), 39.3 (d, *J =* 5.5 Hz, C-4), 50.5 (d, *J =* 2.7 Hz, C-3), 63.8 (d, *J =* 7.9 Hz, O*C*H_2_CH_3_), 65.0 (d, *J =* 7.6 Hz, O*C*H_2_CH_3_), 83.8 (d, *J* = 163.3 Hz, C-2), 120.2 (2CHAr), 126.9 (2CHAr), 127.1 (CHAr), 127.3 (d, *J* = 7.1 Hz, CHAr), 127.5 (2CHAr), 128.3 (2CHAr), 128.4 (2CHAr), 128.8 (2CHAr), 133.5 (C*-ipso*), 136.8 (C*-ipso*), 137.5 (C*-ipso*), 140.8 (C*-ipso*), 166.8 (d, *J* = 3.0 Hz, CO), 169.4 (d, *J* = 15.4 Hz, C-5). HRMS C_27_H_30_N_2_O_4_P [M + H]^+^ 477.1938; found, 477.1940. Anal. Cald. for C_27_H_29_N_2_O_4_P: C, 68.06; H, 6.13; N, 5.88; found: C, 67.94; H, 6.28; N, 5.73%. Purity 97.2% (t_R_ = 4.680 min).

#### Diethyl [(2*RS*,3*RS*)-2-Phenyl-3-((4-(trifluoromethyl)phenyl)carbamoyl)-3,4-dihydro-2*H*-pyrrol-2-yl]phosphonate (12g)

Following the general procedure, **11g** (100 mg, 0.20 mmol) and a solution of 0.05 M NaOH in THF/H_2_O 2:1 (7 mL) gave **12g** (47 mg, 50%) as a white solid, after column chromatography (EtOAc/hexane, 70:30). An analytical sample was recrystallized in EtOAc. Mp 137–139 °C (EtOAc). IR (ATR) 3269, 3087, 2990, 1698, 1560, 1493, 1335, 1222, 1163, 1023, 978, 898, 796 cm^–1^. ^1^H NMR (400 MHz, CDCl_3_) δ 1.09 (td, *J* = 7.0, 0.8 Hz, 3H, OCH_2_C*H*_3_), 1.37 (td, *J* = 7.1, 0.6 Hz, 3H, OCH_2_C*H*_3_), 2.84–2.92 (ddt, *J* = 18.4, 9.6, 1.3 Hz, 1H, H-4), 3.28–3.37(dddd, *J* = 18.4, 8.6, 6.2, 1.0 Hz, 1H, H-4), 3.90 (quin, *J* = 9.2, 1H, H-3), 3.92–3.99 (m, 1H, O*C*H_2_CH_3_), 4.11–4.17 (m, 1H, OC*H*_2_CH_3_), 4.19–4.29 (m, 2H, OC*H*_2_CH_3_), 7.17–7.20 (m, 3H, ArH), 7.27–7.35 (dt, *J* = 15.8, 7.8 Hz, 2H, ArH), 7.48–7.51 (m, 3H, ArH), 7.60 (s, 1H, ArH), 8.06 (dt, *J* = 3.7, 1.2 Hz, 1H, H-5), 9.60 (s, 1H, NH). ^13^C NMR (101 MHz, CDCl_3_) δ 16.2 (d, *J* = 5.8 Hz, OCH_2_*C*H_3_), 16.6 (d, *J* = 5.7 Hz, OCH_2_*C*H_3_), 39.3 (d, *J* = 5.3 Hz, C-4), 50.4 (d, *J* = 2.9 Hz, C-3), 63.8 (d, *J* = 7.9 Hz, O*C*H_2_CH_3_), 65.1 (d, *J* = 7.6 Hz, O*C*H_2_CH_3_), 83.9 (d, *J* = 162.9 Hz, C-2), 116.7 (q, *J* = 4.0 Hz, CHAr), 120.6 (q, *J* = 3.8 Hz, CHAr), 123.0 (d, *J* = 1.2 Hz, CHAr), 125.9 (d, *J* = 273.5 Hz, CF_3_), 127.2 (d, *J* = 7.1 Hz, 2CHAr), 128.3 (d, *J* = 0.6 Hz, 2CHAr), 128.5 (d, *J* = 1.5 Hz, CHAr), 129.3 (CHAr), 131.1 (q, *J* = 32.0 Hz, *C*CF_3_), 133.4 (C*-ipso*), 138.6 (C*-ipso*), 167.3 (d, *J* = 3.5 Hz, CO), 169.3 (d, *J* = 15.3 Hz, C-5). HRMS C_22_H_25_F_3_N_2_O_4_P [M + H]^+^ 469.1499; found, 469.1502. Anal. Cald. for C_22_H_25_ClFN_2_O_5_P: C, 56.41; H, 5.16; N, 5.98; found, C, 56.65; H, 5.28; N, 5.82%.

#### Diethyl [(2*RS*,3*RS*)-3-(Cyclohexylcarbamoyl)-2-phenyl-3,4-dihydro-2*H*-pyrrol-2-yl]phosphonate (12h)

Following the general procedure, **11h** (116 mg, 0.27 mmol) and 0.05 M NaOH in THF/H_2_O 2:1 (6 mL) gave **12h** (80 mg, 73%) as a white solid after column chromatography (EtOAc/MeOH, 95:5). An analytical sample was recrystallized in EtOAc. Mp 143–148 °C (EtOAc). IR (NaCl) 3269, 2934, 2854, 1640, 1560, 1240 cm^–1^. ^1^H NMR (400 MHz, CDCl_3_) δ 0.90–1.25 (m, 5H, CH_2_cycl), 1.08 (td, *J* = 7.2 0.6 Hz, 3H, OCH_2_C*H*_3_), 1.31 (td, *J* = 7.1, 0.5 Hz, 3H, OCH_2_C*H*_3_), 1.50–1.73 (m, 5H, CH_2_cycl), 2.83–2.91 (ddq, *J* = 18.4, 9.6, 1.0 Hz, 1H, H-4), 3.28 (dtd, *J* = 18.4, 7.0, 1.0 Hz, 1H, H-4), 3.34–3.42 (m, 1H, CHcycl), 3.56–3.65 (ddd, *J* = 18.6, 9.6, 7.1 Hz, 1H, H-3), 3.82–3.91 (m, 1H, OC*H*_2_CH_3_), 4.07 (m, 1H, OC*H*_2_CH_3_), 4.10–4.24 (m, 2H, OC*H*_2_CH_3_), 6.54 (d, *J* = 7.6 Hz, 1H, NH), 7.23–7.31 (m, 3H, ArH), 7.54–7–57 (m, 2H, ArH), 7.98 (d, *J* = 4.2 Hz, H-5). ^13^C NMR (101 MHz, CDCl_3_) δ 16.0 (d, *J* = 5.6 Hz, OCH_2_*C*H_3_), 16.3 (d, *J* = 5.8 Hz, OCH_2_*C*H_3_), 24.7 (2CH_2_cycl), 25.5 (CH_2_cycl), 32.3 (CH_2_cycl), 32.9 (CH_2_cycl), 40.8 (d, *J* = 3.7 Hz, C-4), 48.2 (CHcycl), 48.7 (d, *J* = 2.9 Hz, C-3), 63.2 (d, *J* = 7.9 Hz, O*C*H_2_CH_3_), 64.4 (d, *J* = 7.5 Hz, O*C*H_2_CH_3_), 84.4 (d, *J* = 159.8 Hz, C-2), 127.6 (d, *J* = 7.1 Hz, 2CHAr), 127.8 (d, *J* = 1.0 Hz, 2CHAr), 127.9 (d, *J* = 1.8 Hz, CHAr), 134.4 (d, *J* = 1.0 Hz, C-*ipso*), 167.8 (d, *J* = 6.3 Hz, CO), 169.2 (d, *J* = 14.2 Hz, C-5). HRMS C_21_H_32_N_2_O_4_P [M + H]^+^ 407.2096; found, 407.2094. Purity 99.7% (t_R_ = 4.487 min).

#### Diethyl [(2*RS*,3*RS*)-2-(4-Fluorophenyl)-3-(phenylcarbamoyl)-3,4-dihydro-2*H*-pyrrol-2-yl]phosphonate (12i)

Following the general procedure, **11i** (100 mg, 0.23 mmol) and a solution of 0.05 M NaOH in THF/H_2_O 2:1 (7 mL) gave **12i** (47 mg, 50%) as a white solid, after column chromatography (EtOAc/hexane, 70:30). An analytical sample was recrystallized in EtOAc. Mp 188–190 °C (EtOAc). IR (ATR) 3262, 3145, 2988, 1659, 1550, 1443, 1227, 1049, 971, 753, 691 cm^–1^. ^1^H NMR (400 MHz, CDCl_3_) δ 1.10 (t, *J* = 7.0 Hz, 3H, OCH_2_C*H*_3_), 1.35 (t, *J* = 7.1 Hz, 3H, OCH_2_C*H*_3_), 2.84–2.92 (ddt, *J* = 18.5, 9.6, 1.2 Hz, 1H, H-4), 3.32 (dt, *J* = 18.4, 6.7 Hz, 1H, H-4), 3.75–3.84 (quin, *J* = 8.4 Hz, 1H, H-3), 3.87–3.96 (m, 1H, OC*H*_2_CH_3_), 4.07–4.16 (m, 1H, OC*H*_2_CH_3_), 4.17–4.30 (m, 2H, OC*H*_2_CH_3_), 6.89 (t, *J* = 8.6 Hz, 2H, ArH), 7.07 (tt, *J* = 7.3, 1.3 Hz, 1H, ArH), 7.24 (d, *J* = 8.3 Hz, 2H, ArH), 7.32 (d, *J* = 7.6 Hz, 2H, ArH), 7.48–7.52 (ddd, *J* = 8.9, 5.2, 1.7 Hz, 2H, ArH), 8.03 (d, *J* = 3.8 Hz, 1H, H-5), 9.12 (s, 1H, NH). ^13^C NMR (101 MHz, CDCl_3_) δ 16.3 (d, *J* = 5.7 Hz, OCH_2_*C*H_3_), 16.6 (d, *J* = 5.6 Hz, OCH_2_*C*H_3_), 39.7 (d, *J* = 5.0 Hz, C-4), 50.2 (d, *J* = 3.3 Hz, C-3), 63.8 (d, *J* = 8.0 Hz, O*C*H_2_CH_3_), 65.0 (d, *J* = 7.6 Hz, O*C*H_2_CH_3_), 83.5 (d, *J* = 163.0 Hz, C-2), 115.3 (d, *J* = 21.5 Hz, 2 CHAr), 119.8 (2CHAr), 124.2 (CHAr), 129.0 (2CHAr), 129.2 (d, *J* = 7.0 Hz, CHAr), 129.3 (d, *J* = 7.1 Hz, CHAr), 129.6 (d, *J* = 2.8 Hz, C*-ipso*), 137.9 (C*-ipso*), 163.8 (d, *J* = 248.9 Hz, C*-ipso*), 166.8 (d, *J* = 3.8 Hz, CO), 169.6 (d, *J* = 15.1 Hz, C-5). HRMS C_21_H_25_FN_2_O_4_P [M + H]^+^ 419.1530; found, 419.1526. Anal. Cald. for C_21_H_24_FN_2_O_4_P: C, 60.28; H, 5.78; N, 6.70; found, C, 60.29; H, 6.00; N, 6.26%. Purity 97.0% (t_R_ = 4.234 min).

#### Diethyl [(2*RS*,3*RS*)-3-((3-Chloro-4-fluorophenyl)carbamoyl)-2-(4-fluorophenyl)-3,4-dihydro-2*H*-pyrrol-2-yl]phosphonate (12j)

Following the general procedure, **11j** (100 mg, 0.20 mmol) and a solution of 0.05 M NaOH in THF/H_2_O 2:1 (7 mL) gave **12j** (47 mg, 50%) as a white solid, after column chromatography (EtOAc/hexane, 70:30). An analytical sample was recrystallized in EtOAc. Mp 176–178 °C (EtOAc). IR (ATR) 3264, 3069, 2986, 1684, 1499, 1397, 1226, 1187, 1024, 972, 805 cm^–1^. ^1^H NMR (400 MHz, CDCl_3_) δ 1.12 (td, *J* = 7.0, 0.7 Hz, 3H, OCH_2_C*H*_3_), 1.38 (td, *J* = 7.0, 0.6 Hz, 3H, OCH_2_C*H*_3_), 2.83–2.91 (ddt, *J* = 18.2, 9.6, 1.2 Hz, 1H, H-4), 3.25–3.33 (dddd, *J* = 18.5, 8.5, 6.2, 1.0 Hz, 1H, H-4), 3.83 (quin, *J* = 9.4 Hz, 1H, H-3), 3.90–3.99 (m, 1H, OC*H*_2_CH_3_), 4.08–4.16 (m, 1H, OC*H*_2_CH_3_), 4.18–4.29 (m, 2H, OC*H*_2_CH_3_), 6.90 (t, *J* = 8.4 Hz, 2H, ArH), 7.00 (t, *J* = 8.8 Hz, 1H, ArH), 7.17–7.21 (ddd, *J* = 8.9, 4.2, 2.6 Hz, 1H, ArH), 7.45–7.49 (m, 3H, ArH), 8.03 (dt, *J* = 3.7, 1.2 Hz, 1H, H-5), 9.45 (s, 1H, NH). ^13^C NMR (101 MHz, CDCl_3_) δ 16.1 (d, *J* = 5.8 Hz, OCH_2_*C*H_3_), 16.4 (d, *J* = 5.7 Hz, OCH_2_*C*H_3_), 39.2 (d, *J* = 5.2 Hz, C-4), 50.0 (d, *J* = 3.2 Hz, C-3), 63.8 (d, *J* = 8.1 Hz, O*C*H_2_CH_3_), 65.0 (d, *J* = 7.8 Hz, O*C*H_2_CH_3_), 83.2 (d, *J* = 163.3 Hz, C-2), 115.2 (d, *J* = 21.4 Hz, 2CHAr), 116.5 (d, *J* = 22.0 Hz, CHAr), 119.1 (d, *J* = 6.9 Hz, CHAr), 121.0 (d, *J* = 18.7 Hz, C*-ipso*), 121.6 (CHAr), 128.9 (d, *J* = 7.3 Hz, CHAr), 129.0 (d, *J* = 7.3 Hz, CHAr), 129.2 (d, *J* = 3.2 Hz, C*-ipso*), 134.6 (d, *J* = 3.4 Hz, C*-ipso*), 155.7 (d, *J* = 246.8 Hz, C*-ipso*), 162.7 (d, *J* = 249.7 Hz, C*-ipso*), 166.7 (d, *J* = 3.5 Hz, CO), 169.4 (d, *J* = 15.3 Hz, C-5). HRMS C_21_H_23_ClF_2_N_2_O_4_P [M + H]^+^ 471.1047; found, 471.1059. Anal. Cald. for C_21_H_22_ClF_2_N_2_O_4_P: C, 53.57; H, 4.71; N, 5.95; found, C, 54.06; H, 4.95; N, 5.81%.

#### Diethyl [(2*RS*,3*RS*)-2-(4-Methoxyphenyl)-3-(phenylcarbamoyl)-3,4-dihydro-2*H*-pyrrol-2-yl]phosphonate (12k)

Following the general procedure, **11k** (100 mg, 0.22 mmol) and a solution of 0.05 M NaOH in THF/H_2_O 2:1 (7 mL) gave **12k** (47 mg, 50%) as a white solid, after column chromatography (EtOAc/hexane, 70:30). An analytical sample was recrystallized in EtOAc. Mp 105–107 °C (EtOAc). IR (ATR) 3299, 3144, 2987, 1659, 1550, 1443, 1240, 1032, 970, 832, 764 cm^–1^. ^1^H NMR (400 MHz, CDCl_3_) δ 1.10 (t, *J* = 7.0 Hz, 3H, OCH_2_C*H*_3_), 1.37 (t, *J* = 7.0 Hz, 3H, OCH_2_C*H*_3_), 2.87 (dd, *J* = 18.3, 9.6 Hz, 1H, H-4), 3.35 (ddd, *J* = 18.0, 8.2, 6.4 Hz, 1H, H-4), 3.68 (s, 3H, OC*H*_3_), 3.73–382 (quin, *J* = 9.2 Hz, 1H, H-3), 3.86–3.96 (m, 1H, OC*H*_2_CH_3_), 4.07–4.15 (m, 1H, OC*H*_2_CH_3_), 4.17–4.28 (m, 2H, OC*H*_2_CH_3_), 6.71 (d, *J* = 8.8 Hz, 2H, ArH), 7.05 (t, *J* = 7.4 Hz, 1H, ArH), 7.25 (t, *J* = 7.6 Hz, 2H, ArH), 7.36 (d, *J* = 7.6 Hz, 2H, ArH), 7.42 (dd, *J* = 8.8, 1.6 Hz, 2H, ArH), 8.01 (d, *J* = 3.6 Hz, 1H, H-5), 9.26 (s, 1H, NH). ^13^C NMR (101 MHz, CDCl_3_) δ 16.3 (d, *J* = 5.8 Hz, OCH_2_*C*H_3_), 16.6 (d, *J* = 5.7 Hz, OCH_2_*C*H_3_), 39.2 (d, *J* = 5.7 Hz, C-4), 50.3 (d, *J* = 3.3 Hz, C-3), 55.2 (O*C*H_3_), 63.6 (d, *J* = 8.0 Hz, O*C*H_2_CH_3_), 64.9 (d, *J* = 7.6 Hz, O*C*H_2_CH_3_), 83.3 (d, *J* = 163.2 Hz, C-2), 113.7 (2CHAr), 119.9 (2CHAr), 124.0 (CHAr), 125.4 (C*-ipso*), 128.6 (d, *J* = 7.1 Hz, 2CHAr), 128.9 (2CHAr), 138.2 (C*-ipso*), 159.4 (d, *J* = 1.4 Hz, C*-ipso*), 166.9 (d, *J* = 3.0 Hz, CO), 169.1 (d, *J* = 15.6 Hz, C-5). HRMS C_22_H_28_N_2_O_5_P [M + H]^+^ 431.1730; found, 431.1727. Anal. Cald. for C_22_H_27_N_2_O_5_P: C, 61.39; H, 6.32; N, 6.51; found, C, 61.14; H, 6.41; N, 6.34%. Purity 98.0% (t_R_ = 4.156 min).

#### Diethyl [(2*RS*,3*RS*)-3-((3-Chloro-4-fluorophenyl)carbamoyl)-2-(4-methoxyphenyl)-3,4-dihydro-2*H*-pyrrol-2-yl]phosphonate (12l)

Following the general procedure, **11l** (100 mg, 0.20 mmol) and a solution of 0.05 M NaOH in THF/H_2_O 2:1 (7 mL) gave **2l** (47 mg, 50%) as a white solid, after column chromatography (EtOAc/hexane, 70:30). An analytical sample was recrystallized in EtOAc. Mp 121–123 °C (EtOAc). IR (ATR) 3257, 3073, 2981, 1689, 1499, 1218, 1027, 975, 828, 789 cm^–1^. ^1^H NMR (400 MHz, CDCl_3_) δ 1.12 (t, *J* = 7.2, 0.9 Hz, 3H, OCH_2_C*H*_3_), 1.37 (td, *J* = 7.0, 0.6 Hz 3H, OCH_2_C*H*_3_), 2.79–2.87 (ddd, *J* = 18.4, 9.5, 1.4 Hz, 1H, H-4), 3.25–3.33 (dddd, *J* = 18.4, 8.9, 6.2, 1.0 Hz, 1H, H-4), 3.71 (s, 3H, OC*H*_3_), 3.81 (quin, *J* = 9.0 Hz, 1H, H-3), 3.89–3.99 (m, 1H, OC*H*_2_CH_3_), 4.08–4.16 (m, 1H, OC*H*_2_CH_3_), 4.18–4.28 (m, 2H, OC*H*_2_CH_3_), 6.73 (d, *J* = 8.2 Hz, 2H, ArH), 7.02 (t, *J* = 8.7 Hz, 1H, ArH), 7.21 (ddd, *J* = 8.9, 4.2 2.6 Hz, 1H, ArH), 7.38 (dd, *J* = 9.1, 1.7 Hz, 2H, ArH), 7.49 (dd, *J* = 6.6, 2.6 Hz, 1H, ArH), 8.01 (dt, *J* = 3.6, 1.1 Hz, 1H, H-5), 9.52 (s, 1H, NH). ^13^C NMR (101 MHz, CDCl_3_) δ 16.3 (d, *J* = 5.8 Hz, OCH_2_*C*H_3_), 16.6 (d, *J* = 5.6 Hz, OCH_2_*C*H_3_), 38.9 (d, *J* = 5.7 Hz, C-4), 50.3 (d, *J* = 3.4 Hz, C-3), 55.3 (OCH_3_), 63.8 (d, *J* = 7.9 Hz, O*C*H_2_CH_3_), 65.1 (d, *J* = 7.6 Hz, O*C*H_2_CH_3_), 83.2 (d, *J* = 163.5 Hz, C-2), 113.7 (2CHAr), 116.4 (d, *J* = 22.0 Hz, CHAr), 119.4 (d, *J* = 6.8 Hz, CHAr), 121.0 (d, *J* = 18.7 Hz, C*-ipso*), 122.0 (CHAr), 125.1 (C*-ipso*), 128.5 (d, *J* = 7.2 Hz, 2CHAr), 134.9 (d, *J* = 3.2 Hz, C*-ipso*), 155.8 (d, *J* = 246.3 Hz, C*-ipso*), 159.5 (d, *J* = 1.4 Hz, C*-ipso*), 167.1 (d, *J* = 2.9 Hz, CO), 169.1 (d, *J* = 15.6 Hz, C-5). HRMS C_22_H_26_ClFN_2_O_5_P [M + H]^+^ 483.1246; found, 483.1249. Anal. Cald. for C_22_H_25_ClFN_2_O_5_P: C, 54.72; H, 5.22; N, 5.80; found: C, 54.91; H, 5.50; N, 5.65%.

#### Diethyl [(2*RS*,3*RS*)-2-Methyl-3-(phenylcarbamoyl)-3,4-dihydro-2*H*-pyrrol-2-yl]phosphonate (12m)

Following the general procedure, **11m** (62 mg, 0.17 mmol) and 0.05 M NaOH in THF/H_2_O 2:1 (4 mL) gave **12m** (18 mg, 31%) as a white solid after column chromatography (AcOEt:MeOH, 95:5). An analytical sample was recrystallized in EtOAc. Mp 129–132 °C (EtOAc). IR (ATR) 3339, 2982, 2911, 1665, 1599, 1543, 1443, 1228, 1047, 1016, 955, 755, 691 cm^–1^. ^1^H NMR (400 MHz, CDCl_3_) δ 1.36 (t, *J* = 9.2 Hz, 3H, OCH_2_C*H*_3_), 1.38 (d, *J* = 14.9 Hz, 3H, CH_3_), 1.44 (t, *J* = 7.0 Hz, 3H, OCH_2_C*H*_3_), 2.75–2.85 (m, 1H, H-4), 3.43–3.57 (complex signal, 2H, H-3 and H-4), 4.16–4.26 (m, 2H, OC*H*_2_CH_3_), 4.29–4.39 (m, 2H, OC*H*_2_CH_3_), 7.06–7.10 (tt, *J* = 7.4, 1.2 Hz, 1H, ArH), 7.29–7.33 (t, *J* = 7.4 Hz, 2H, ArH), 7.58 (complex signal, 1H, ArH), 7.61 (m, 2H, H-5 and ArH) 9.65 (br s, 1H, NH).^13^C NMR (101 MHz, CDCl_3_) δ 16.4 (d, *J* = 5.7 Hz, OCH_2_*C*H_3_), 16.5 (d, *J* = 5.9 Hz, OCH_2_*C*H_3_), 17.3 (*C*H_3_), 38.2 (d, *J* = 6.7 Hz, C-4), 47.3 (d, *J* = 3.8 Hz, C-3), 63.5 (d, *J* = 7.7 Hz, O*C*H_2_CH_3_), 64.2 (d, *J* = 7.4 Hz, O*C*H_2_CH_3_), 76.7 (d, *J* = 168.6 Hz, C-2), 119.6 (2CHAr), 123.9 (CHAr), 128.9 (2CHAr), 138.4 (C-*ipso*), 166.7 (d, *J* = 16.6 Hz, C-5), 167.3 (d, *J* = 1.6 Hz, CO). HRMS C_16_H_24_N_2_O_4_P [M + H]^+^ 339.1475; found, 339.1468.

#### Diethyl [(2*RS*,3RS)-2-Benzyl-3-(phenylcarbamoyl)-3,4-dihydro-2*H*-pyrrol-2-yl]phosphonate (12n)

Following the general procedure, **11n** (45 mg, 0.10 mmol) and a solution of 0.05 M NaOH in THF/H_2_O 2:1 (4 mL) gave **12n** (8 mg, 19%) as a yellowish oil after column chromatography (EtOAc/hexane, 60:40). IR (ATR) 3263, 3196, 3032, 2925, 2853, 1688, 1621, 1601, 1555, 1496, 1443, 1301, 1253, 1218, 1050, 1023, 971, 792, 755, 729, 695 cm^–1^. ^1^H NMR (400 MHz, CDCl_3_) δ 1.32–1.38 (dtd, *J* = 10.7, 7.1, 0.6 Hz, 6H, OCH_2_C*H*_3_), 2.50–2.57 (dddd, *J* = 18.6, 10.3, 5.7, 1.0 Hz, 1H, H-4), 2.60 (dd, *J* = 9.9, 1.4 Hz, 1H, H-4), 3.22–3.34 (m, 2H, C*H*_2_-benzyl), 3.53–3.63 (quin, *J* = 9.8 Hz, 1H, H-3), 4.16–4.31 (m, 4H, OC*H*_2_CH_3_), 7.10 (m, 3H, ArH), 7.18 (m, 3H, ArH), 7.36 (m, 2H, ArH), 7.70 (complex signal, 2H, ArH), 7.72 (complex signal, 1H, H-5), 10.28 (br s, 1H, NH). ^13^C NMR (101 MHz, CDCl_3_) δ 16.4 (d, *J* = 1.6 Hz, CH_2_*C*H_3_), 16.5 (d, *J* = 1.7 Hz, CH_2_*C*H_3_), 36.6 (*C*H_2_-benzyl), 39.3 (d, *J* = 7.6 Hz, C-4), 47.8 (d, *J* = 3.1 Hz, C-3), 63.6 (d, *J* = 7.8 Hz, *C*H_2_CH_3_), 64.4 (d, *J* = 7.8 Hz, *C*H_2_CH_3_), 79.7 (d, *J* = 164.5 Hz, C-2), 119.4 (2CHAr), 123.8 (CHAr), 127.0 (CHAr), 127.6 (2CHAr), 129.0 (2CHAr), 131.4 (2CHAr), 134.0 (d, *J* = 13.5 Hz, C-*ipso*), 138.8 (C-*ipso*), 166.7 (CO), 168.9 (d, *J* = 17.1 Hz, C-5). HRMS C_22_H_28_N_2_O_4_P [M + H]^+^ 415.1781; found, 415.1763. Purity: 95.2% (t_R_ = 4.703 min).

#### Diethyl [(2*RS*,3*RS*)-2-Benzyl-3-(cyclohexylcarbamoyl)-3,4-dihydro-2*H*-pyrrol-2-yl]phosphonate (12o)

Following the general procedure, **11o** (76 mg, 0.17 mmol) and a solution of 0.05 M NaOH in THF/H_2_O 2:1 (4 mL) gave **12o** (41 mg, 57%) as a white solid, after column chromatography (EtOAc/hexane, 70:30). An analytical sample was recrystallized in EtOAc. Mp 119–121 °C (EtOAc). IR (ATR) 3522, 3294, 2928, 2856, 1638, 1541, 1238, 1216, 1025, 974, 959, 758, 697 cm^–1^. ^1^H NMR (400 MHz, CDCl_3_) δ 1.28 (tdd, *J* = 7.1, 1.6 0.6 Hz, 6H, OCH_2_C*H*_3_), 1.33–1.48 (m, 5H, CH_2_cycl), 1.58–1.64 (m, 1H, CH_2_cycl), 1.71–1.81 (m, 2H, CH_2_cycl), 1.89–1.95 (m, 1H, CH_2_cycl), 2.00–2.08 (m, 1H, CH_2_cycl), 2.53–2.62 (ddd, *J* = 18.6, 10.2, 1.5 Hz, 1H, H-4), 2.66–2.77 (dddd, *J* = 16.1, 9.9, 6.2, 1.0 Hz, 1H, H-4), 3.09–3.26 (complex signal, 2H, C*H*_2_-benzyl), 3.44 (dt, *J* = 19.3, 10.1 Hz, 1H, H-3), 3.81–3.90 (m, 1H, CHcycl), 3.98- 4.17 (m, 4H, OC*H*_2_CH_3_), 7.15–7.23 (m, 5H, ArH), 7.63 (br d, *J* = 3.2 Hz, 1H, NH), 7.82 (d, *J* = 7.8 Hz, 1H, H-5). ^13^C NMR (101 MHz, CDCl_3_) δ 16.4 (d, *J* = 5.7, 2OCH_2_*C*H_3_), 24.8 (d, *J* = 3.0 Hz, 2CH_2_cycl), 25.6 (CH_2_cycl), 32.9 (2CH_2_cycl), 36.8 (*C*H_2_-benzyl), 39.6 (d, *J* = 7.1 Hz, C-4), 47.1 (d, *J* = 3.0 Hz, C-3), 48.5 (CHcycl), 63.4 (d, *J* = 7.7 Hz, O*C*H_2_CH_3_), 63.8 (d, *J* = 7.7 Hz, O*C*H_2_CH_3_), 79.9 (d, *J* = 164.9 Hz, C-2), 126.7 (CHAr), 127.5 (2CHAr), 131.5 (2CHAr), 135.0 (d, *J* = 10.9 Hz, C-*ipso*), 167.9 (d, *J* = 1.9 Hz, CO), 168.9 (d, *J* = 16.8 Hz, C-5). HRMS C_22_H_34_N_2_O_4_P [M + H]^+^ 421.2251; found, 421.2266. Purity 97.0% (t_R_ = 4.639 min).

### X-Ray Crystallographic Analysis of 12b, 12d, and 12h

Crystals of **12b**, **12d**, and **12h** were obtained from slow evaporation of methanol solutions. Single crystal X-ray diffraction data sets for **12d** and **12h** were collected at 295 K up to a max 2ϑ of ca. 57° on a Bruker Smart APEX II diffractometer, using monochromatic MoKα radiation λ = 0.71073 Å and 0.3° separation between frames. Data integration was performed using SAINT V6.45A and SORTAV^66^ in the diffractometer package.^67^ The single crystal experiment for **12b** was made on an Enraf Nonius CAD4 diffractometer using also MoKα radiation. The crystal and collection data and structural refinement parameters are given in the Tables S3–S17 in the Supporting Information. The structures were solved by direct methods using SHELXT-2014 and Fourier’s difference methods and refined by least-squares on F2 using SHELXL-2014/7 inside the WinGX program environment.^68,69^ Anisotropic displacement parameters were used for non-H atoms and the H atoms were positioned in calculated positions and refined riding on their parent atoms. Atom coordinates are given and bond distances and hydrogen bonds. All this information is detailed in the Tables S3–S17 in the Supporting Information.

**12b** crystallized in the triclinic P-1 space group (Ortep in Figure 3). The 1-pyrroline ring presented envelope conformation on C3 (E-form). The phenyl ring appears as flat. The stereocenters C2 and C3 exhibit identical quiralities, either R, R or S, S. Molecules are packed in dimers through the mutual N7–H7···O1 hydrogen bond.

**12d** crystallized in the triclinic P-1 space group (Ortep in Figure 3). The 1-pyrroline ring presented envelope conformation on C3. The phenyl rings appear as flat. The stereocenters C2 and C3 exhibit opposed quiralities either R, S or S, R, as the space group is centrosymmetric. Molecules are packed in dimers through the mutual N7–H7···O1 hydrogen bond.

**12h** crystallized in the monoclinic P21/a space group space group with four molecules per asymmetric unit (Ortep in Figure 3). All trifluoromethyl groups have been modeled as having two possible rotational disordered orientations (for two molecules, the disorder was about 50:50, and for the other two, 80:20 and 64:36, approximately). Also, one terminal ethyl group exhibited two different orientations (67:33). The five-membered rings present envelope conformations on C3, C23, C43 and C63. The six-membered rings appeared all as planar. The stereocenters C2 and C3 showed opposed quirality, as well as C22 and C23, C42 and C43 and C62 and C63 (S and R, respectively, for the asymmetric unit provided). Molecules are packed as two dimers through the N7–H7···O10 and N67–H67···O1 hydrogen bonds, for one of them, and through the N27–H27···O7 and N47–H47···O5 hydrogen bonds, for the other. Dimers are further linked by other hydrogen bonds of different types (C–H···F, C–H···O, C–H···N), amounting for a total of 23 identified formal HBs (see Table S17 for **12h**)

Crystallographic data for the reported structures has been deposited in the Cambridge Crystallographic Data Centre as supplementary publication, CCDC No. 2369628, 2369629, and 2369630. Copies of this information may be obtained free of charge from The Director, CCDC, 12 Union Road, Cambridge CB2 1EZ, UK. Fax: +44 1223 336 033. Email: data_request@ccdc.cam.ac.uk. Web page: http://www.ccdc. cam.ac.uk.

### Binding Studies

#### Preparation of Cellular Membranes

Human brain samples were obtained at autopsy in the Basque Institute of Legal Medicine, Bilbao, Spain. Samples from the prefrontal cortex (Brodmann’s area 9) were dissected at the time of autopsy and immediately stored at −70 °C until assay. The study was developed in compliance with policies of research and ethical review boards for post-mortem brain studies. For I1-IR experiments kidneys were obtained from male Sprague–Dawley rats (250–280 g).

To obtain cellular membranes (P2 fraction) the different samples were homogenized using an ultraturrax in 10 volumes of homogenization buffer (0.25 M sucrose, 5 mM Tris–HCl, pH 7.4). The crude homogenate was centrifuged for 5 min at 1000 g (4 °C) and the supernatant was centrifuged again for 10 min at 40,000 g (4 °C). The resultant pellet was washed twice in 5 volumes of homogenization buffer and recentrifuged in similar conditions. Protein content was measured according to the method of Bradford using BSA as standard.

#### Competition Binding Assays

The pharmacological activity of the compounds was evaluated through competition binding studies against the I_2_–IRs selective radioligand [^3^H]2-BFI, the I_1_–IRs selective radioligand [^3^H]clonidine, or the α_2_-adrenergic receptor selective radioligand [^3^H]RX821002. Specific binding was measured in 0.25 mL aliquots (50 mM Tris-HCl, pH 7.5) containing 100 μg of membranes, which were incubated in 96-well plates either with [^3^H]2-BFI (2 nM) for 45 min at 25 °C, [^3^H]clonidine (5 nM) for 45 min at 22 °C or [^3^H]RX821002 (1 nM) for 30 min at 25 °C, in the absence or presence of the competing compounds (10^–12^ to 10^–3^ M, 10 concentrations). [^3^H]clonidine experiments were performed in the presence of 10 μM adrenaline to preclude binding to α2-adrenoceptors.

Incubations were terminated by separating free ligand from bound ligand by rapid filtration under vacuum (1450 Filter Mate Harvester, PerkinElmer) through GF/C glass fiber filters. The filters were then rinsed three times with 300 μL of binding buffer, air-dried (120 min), and counted for radioactivity by liquid scintillation spectrometry using a MicroBeta TriLux counter (PerkinElmer). Specific binding was determined and plotted as a function of the compound concentration. Nonspecific binding was determined in the presence of idazoxan (10^–5^ M), a compound with well stablished affinity for I_2_–IRs and α_2_-ARs, in [^3^H]2-BFI and [^3^H]RX821002 assays. To obtain the inhibition constant (*K*_i_) analyses of competition experiments were performed by nonlinear regression using the GraphPad Prism program. *K*_i_ values were normalized to p*K*_i_ values. I_2_–IRs/α_2_ selectivity index was calculated as the antilogarithm of the difference between p*K*_i_ values for I_2_–IRs and p*K*_i_ values for α_2_-AR. For [^3^H]clonidine experiments logIC_50_ values were calculated (the concentration of tested ligand that displaces 50% of specifically bound [^3^H]clonidine).

### 3D-QSAR study. Data Set Preparation

The three-dimensional Quantitative Structure Activity Relationship (3D-QSAR) study was performed on a data set consisting of 17 I_2_–IRs and 16 α_2_-ARs ligands (4 previously reported compounds: Idazoxan, CR40506, BU99008.^25^ and B06,^26^ and 15 new compounds, Table 1 and 2).

The compounds reported here (Table 2) with p*K*_i_ values between 3.07 and 9.98 for I_2_–IRs and 2.65–9.43 for α_2_-ARs were used for 3D-QSAR modeling.

For all compounds studied, the dominant forms were determined at a phisiological pH of 7.4 using the program Marvin Sketch 5.5.1.0.^70^ The semiempirical/PM3 method (Parameterized Model revision 3)^71,72^ was applied in the first step of geometry optimization on selected dominant forms, followed by the ab initio Hartree–Fock/3-21G method,^73^ using the Gaussian 09 software,^74^ which is part of the ChemBio3D Ultra 13 program.^75^

The Pentacle program^76^ was used for the calculation of alignment-independent three-dimensional molecular descriptors (GRIND) and 3D-QSAR models building. The computation of GRIND descriptors is based on Molecular Interaction Fields (MIF) by using four different probes: O probe (hydrogen bond acceptor groups), N1 probe (hydrogen bond donor groups), TIP probe (the shape of molecule), and DRY probe (hydrophobic interactions). In the next step the ALMOND algorithm was applied for the extraction of the most relevant regions representing favorable interaction positions between ligand and probe. Finaly, the CLACC (Consistently Large Auto and Cross Correlation) algorithm with a smoothing window of 0.8 Å was applied to display node–node energies, between the same or a different probe, into auto- and cross-correlograms.^77^

The analyzed data set was divided into a training set for the creation of the 3D-QSAR model and a test set for the external validation of the model. The 3D-QSAR (I_2_–IRs) model was built with 17 compounds, 12 ligands in the training set and 5 ligands in the test set, while the 3D-QSAR (α_2_-ARs) model was built with 11 compounds in the training set and 5 compounds in the test set. Ligands with p*K*_i_ values less than 3 were not used for 3D-QSAR analysis. The PCA (Principal Component Analysis) plot was used to select a test set of compounds, taking into account that the p*K*_i_ values were homogeneously distributed over the entire range of activities studied. The most significant variables were selected using a Fractional Factorial Design (FFD), and the final 3D-QSAR models were created by Partial Least Square (PLS) regression.

### *In Vitro* Effects of 12b in a Preclinical Model of Neurodegeneration

#### SH-SY5Y Cell Culture

SH-SY5Y human neuroblastoma-derived cells (ATCC, Rockville, MD, USA, ref.CRL-2266) were cultured and maintained in DMEM medium (Sigma) supplemented with 2 mM glutamine (Sigma) and 10% fetal bovine serum (FBS, Gibco) and 1% penicillin/streptomycin under standard conditions. For cell viability assays, cells were seeded onto 96-well plates at a density of 3 × 10^4 cells per well. Upon reaching semiconfluence, cells were treated with 35 μM 6-OHDA (Sigma) for 18 h.

#### Primary Glial Cultures

Primary glial cultures were isolated from the cerebral cortex of 3-day-old mice as described previously.^78^ In brief, the cerebral cortex was dissected, dissociated, and incubated with trypsin/EDTA (0.25%) at 37 °C for 1 h. Following centrifugation, the pellet was washed with HBSS (Gibco) and the cells were plated in poly-d-lysine (20 μg/mL) pretreated flasks (75 cm^2^). After 7–10 days, the flasks were agitated in an orbital shaker at 230 rpm for 4 h at 37 °C to isolate nonadherent microglial cells, which were then plated onto 24-well plates at a density of 3 × 10^5 cells per well. The flasks were subsequently replenished with DMEM and agitated in a horizontal shaker at 260 rpm at 37 °C. After overnight agitation, the supernatant, containing oligodendrocytes and some residual microglial cells, was removed. Astrocytes, which remained adherent, were collected and plated onto 96-well plates at a density of 3 × 10^5 cells per well for the neuronal survival studies, and onto 24-well plates (10 × 10^5 cells/well) for the analysis of inflammation by immunofluorescence. The purity of the cultures exceeded 95%, as determined by immunofluorescence analysis using an antiglial fibrillary acidic protein (GFAP; clone G-A-5; Sigma-Aldrich) antibody to identify astrocytes, an anti-Iba1 (Wako) antibody to identify microglial cells, and an anti-O4 (Millipore) antibody as an oligodendrocyte marker.

#### Treatments

SH-SY5Y, astrocytes and microglial cells seeded on 96-well plates were treated with compound **12d** at different doses (0.5 μM to 20 μM). Some SH-SY5Y cultures were treated with the neurotoxic agent 6-hydroxydopamine (6-OHDA, 35 μM, Merck-Sigma) as a control for cellular damage, and glial cells with bacterial lipopolysaccharide (LPS, 10 μg/mL, Merck-Sigma) as an inducer of inflammation. Then cellular viability and nitrite production (indicator of inflammation) was assessed using the MTT and the Griess assays, respectively. None of the concentrations of **12d** tested were neurotoxic or pro-inflammatory at those doses. Then, cultures were treated for 1h with compound **12d** at 1, 5, and 10 μM. Some cultures were treated with the well-known anti-inflammatory compound CR4056 (1 μM). Doses were chosen based on previous studies.^27,28^ Subsequently, SH-SY5Y cultures were exposed to 6-OHDA (35 μM) for 18 h. To determine the inflammatory state, glial cells subcultured in 96-well plates were exposed to LPS (10 μg/mL). Finally, the cellular viability and nitrite production of the cultures were assessed.

#### Cell Viability Assay

Cell viability was determined using the 3-(4,5-dimethylthiazol-2-yl)-5-(3-carboxymethoxyphenyl)-2-(4-sulfophenyl)-2H-tetrazolium (MTT) assay kit (Roche Diagnostic, GmbH) on SH-SY5Y cultures, based on the ability of viable cells to reduce yellow MTT to blue formazan. The extent of reduction of MTT was quantified by absorbance measurement at 595 nm according to the manufacturer’s protocol. Each data point represents the mean ± SD of 6 replications in 3 different experiments and the results were expressed as a percentage of the control.

#### Nitrite Measurement

After treatments, 100 μL of the cell culture supernatant (SH-SY5Y and glial cultures) was mixed with an equal volume of Griess reagent (consisting of 1% sulfanylamide and 0.1% naphthyl ethylene diamine in 5% phosphoric acid, sourced from Sigma-Aldrich, Madrid, Spain) at room temperature for 15 min. To determine the nitrite concentrations, a standard solution of sodium nitrite was employed. The absorbance of the mixture was measured at 492/540 nm using a microplate reader from Thermofisher (Madrid, Spain). The experiments were repeated a minimum of three times.

#### Immunocytochemical Analysi

For immunofluorescence analysis, primary glial cultures, grown on glass coverslips in 24-well cell culture plates, were fixed in phosphate-buffered saline (PBS) containing 4% paraformaldehyde (Sigma), blocked in PBS containing 0.1% Triton X-100 for 30 min at 37 °C, and incubated overnight at 4 °C with primary antibodies. The following antibodies were used: rabbit polyclonal anti-COX2 (Cayman) and mouse monoclonal anti-TNFα (Abcam). Cells were then incubated for 45 min at 37 °C with appropriate Alexa Fluor 488- and 546-conjugated secondary antibodies (Jackson Immuno Research). Nuclei were stained with DAPI. Coverslips were mounted with Vectashield Mounting Medium (Vector Laboratories). Images were captured using a Zeiss LSM710 laser scanning spectral confocal microscope equipped with Plan-Apochromat 63*X*/1.4. Confocal microscope settings were adjusted to produce the optimum signal-to-noise ratio. To compare fluorescence signals from different preparations, settings were fixed for all samples within the same analysis. Representative images of at least three independent experiments are shown.

#### Statistical Analysis

*In vitro* models of neurodegeneration and neuroinflammation, Figures 4 and 5, are expressed as the mean ± standard deviation (SD) of six replicates determinations. The experiments were repeated at least three times, and consistent outcomes were obtained. Data analysis was initially performed using one-way analysis of variance (ANOVA). Subsequently, post hoc statistical analyses were conducted using the Tukey test, with a significance level set at *p* < 0.05. The SPSS statistical software package, version 20.0, for Windows (Chicago, IL, USA), was utilized for these analyses.

### *In Vivo* Pharmacokinetics

The pharmacokinetic study was carried on in male CD1 mice (Envigo Laboratories) with a body weight between 40 to 50 g (n = 3–4 per group). Animals were randomized to be included in the treated or control groups. A single intraperitoneal dose of **12d** (30 mg/kg, 10 mL/kg) was administered early in the morning (between 8 and 11 a.m.) without anesthesia. The compound was dissolved in 10% of 2-hydroxypropyl-β-cyclodextrin in physiological saline. Mice were monitored for signs of pain or distress during the time between injection and euthanasia. Mice were sacrificed by cervical dislocation and blood (0.6 mL) was collected at different time points (0 min, 5 min, 10 min, 15 min, 30 min, 45 min, 60 min, 2 h, 3 h, 4 h, 6 h, 8 and 24 h after injection) in tubes with serum gel and clotting activator (Sarstedt Micro tube 1.1 mL Z-Gel). Samples were centrifugated at 10.000 rpm for 10 min to obtain plasma and stored at −80 °C up to analysis of compound concentration by UPLC-MS/MS. Experimental procedures were in line with the Directive 2010/63/EU and approved by the Institutional Animal Care and Generalitat de Catalunya (#10291, 1/28/2018).

### Hypothermic Effects Evaluation: Changes in Core Body Temperature

Twenty-five adult CD-1 mice (30–40 g), bred in the animal facility of the University of the Balearic Islands, were used in this study. All experimental procedures were performed following the ARRIVE (McGrath and Lilley, 2015) and standard ethical guidelines (European Communities Council Directive 86/609/EEC; Guidelines for the Care and Use of Mammals in Neuroscience and Behavioral Research, National Research Council 2003), and were approved by the Local Bioethics Committee (UIB CAIB). All efforts were made to minimize the number of mice used and their suffering. Mice had continuous access to a regular diet and tap water while being housed in standard cages under specific conditions (22 °C, 70% humidity, and 12 h light/dark cycle, lights on at 8:00 h). Prior to drug treatment, mice were habituated to the experimenter by being handled and weighed for 2 days. For the acute treatment, mice received (i.p.) either a single dose of **12b** (20 mg/kg, n = 6) or vehicle (1 mL/kg of DMSO, n = 7). For the repeated treatment, mice received a daily injection (i.p.) of **12b** (20 mg/kg, n = 6) or vehicle (n = 6) for 5 consecutive days. Changes on rectal temperature were calculated by subtracting the values obtained with a rectal probe connected to a digital thermometer (Compact LCD display thermometer, SA880–1M, RS, Corby, UK) 1 h post-treatment minus before drug treatment (baseline values). Mice were sacrificed right after the last temperature recording, and the hippocampus was freshly dissected and kept at −80 °C until further neurochemical analyses were performed.

### *In Vivo* Treatment in SAMP8Mice

Male and female SAMP8 (n = 16, 10-month-old) were used to perform behavioral and molecular analyses. Animals were randomly divided into control (n = 8), and **12d** (5 mg·kg–1·day–1) (n = 8). The animals had free access to food and water and were kept under standard temperature conditions (22 ± 2 °C) and 12-h light/dark cycles (300/0 lx). Compounds were dissolved in 1.8% (2-hydroxypropyl)-β-cyclodextrin and administered through drinking water. Control groups received water plus 1.8% (2-hydroxypropyl)-β-cyclodextrin during the treatment period. For the drug administration, dosages were calculated based on average daily water consumption recorded in each cage, and they were confirmed by recalculations once a week. Each animal’s weight was also recorded once a week during the treatment period and the drug dosages were recalculated, when necessary, based on the results obtained. After 4 weeks of treatment, Novel Object Recognition and Novel Object Location tests were performed to study the effects of treatment on working and spatial memory (Figure 9). Mice were also treated during this period and up to their euthanasia. All studies and procedures for the mouse behavior test of brain dissection and extractions followed the standard ethical guidelines (European Communities Council Directive 2010/63/EU and Guidelines for the Care and Use of Mammals in Neuroscience and Behavioral Research, National Research Council 2003). Animal studies are reported in compliance with the ARRIVE guidelines,^79^ and were approved by Bioethical Committees from the University of Barcelona and the Government of Catalonia (#222/18).

#### Novel Object Recognition Test

A modification of the novel object recognition test protocol was performed (Ennaceur & Delacour, 1988). In brief, mice were placed in a 90° two-arm (25 × 20 × 5 cm) black maze, with removable walls for easy cleaning and light intensity in midfield was 30 lx. Before the memory, trial mice were habituated to the apparatus for 10 min for 3 days. On day 4, the animals were submitted to a 10 min acquisition trial, in which they were allowed to freely explore two identical objects located at the end of each arm (first trial—familiarization). After 2 h (for short-term memory evaluation) and 24 h (for long-term memory evaluation) from the first trial, the mice were submitted to a 10 min retention trial, in which one of the two identical objects had been replaced by a novel one. The behavior was recorded, and the time that the mice spent exploring the new object (TN) and the old one (TO) was measured manually. Exploration was defined as sniffing or touching objects with the nose and/or forepaws. The discrimination index (DI) was calculated as (TN – TO)/(TN + TO). To avoid object preference biases, objects were alternated; 70% EtOH was used to clean the arms and objects after each trial to eliminate olfactory cues.

#### Object Location Test

The test was performed in a cage (50 × 50 × 25 cm), in which three walls were white except one that was black and lasted 3 days. On day 1, mice were familiarized to the arena for 10 min. On day 2, two identical objects (A+A) were in front of the black wall, and the mice were freely allowed to explore both objects for 10 min (Trial 1–training phase). On day 3, after a retention period of 24 h mice were returned to the testing arena for another 10 min (Trial 2–testing phase) with one object moved to a different position (opposite direction toward the black wall) and were allowed to explore (Figure 6). The trials were recorded, and the object exploration time was measured manually. The time sniffing the object in the old position (PO) and the time exploring the object in the new position (TN) were evaluated. The DI defined as (PN-TO)/(PN+PO) was determined as an indicator of cognitive performance. For the elimination of olfactory cues, 70% EtOH was used to clean the testing arena after each trial.

#### Western Blot Analysis for Neurochemical Markers

Following standardized Western blot procedures,^19^ total homogenates from each hippocampal sample were prepared and loaded (40 μg) into 10–12% SDS-PAGE polyacrilamide gels (Bio-Rad), and transferred onto nitrocellulose membranes to later label the content of key target proteins using primary antibodies. In particular, membranes were incubated overnight with anti-FADD (H-181, sc-5559, Santa Cruz Biotechnology, Santa Cruz, CA), anti-Cdk5 (Ab-2, DC17, Lab Vision, NeoMarkers, USA), anti-pERK1/2 (#9101L, Cell Signaling, MA, USA), anti-ERK (CEMI0112011, Clone 631122, R&D Systems, Biotechne, USA) anti-PDE1B (Santa Cruz/sc-393112); SYT7 (Santa Cruz/sc-293343); HPCA (Santa Cruz/sc393125); CAMKII (Abcam/ab52476). Membranes were stripped and reprobed with β-actin antibody (clone AC-15, no. A1978, Sigma) or GAPDH (Millipore/MAB374), since its content was used as a loading control (no differences between treatment groups, data not shown). Then, with the appropriate secondary antibody incubation (Goat-antimouse HRP conjugated, Biorad/170–5047, or Goat-antirabbit HRP conjugated, Biorad/170–6515), and posterior ECL detection system (Amersham, Buckinghamshire, UK), target proteins were visualized on autoradiographic films (Amersham ECL Hyperfilm), and immunoreactive bands were quantified (integrated optical density, IOD) by densitometric scanning (GS-800 Imaging Densitometer, Bio-Rad). The amount of target protein (e.g., FADD, Cdk5, pERK/ERK, CaMKII, SYT7) from each mouse treated with **12b** was compared in the same gel with that of control mice (% change vs acute or repeated controls). Each brain sample (and target protein) was quantified at least in 2–4 gels, and the mean value was used as a final estimate.

#### RNA Extraction and Gene Expression Determination

Total RNA isolation from hippocampal samples was performed using the TRIzol reagent according to the manufacturer’s instructions (Bioline Reagents, London, UK). The yield, purity, and quality of RNA were determined spectrophotometrically with a NanoDrop ND-1000 apparatus (Thermo Fisher, Waltham, MA, USA) and an Agilent 2100B Bioanalyzer (Agilent Technologies, Santa Clara, CA, USA). RNA samples with 260/280 ratios and RINs higher than 7.5, respectively, were selected. RT-PCR was performed. Briefly, 2 μg of mRNA was reverse transcribed using a high-capacity cDNA reverse transcription kit (Applied Biosystems, Foster City, CA, USA). SYBR Green real-time PCR was performed using a Step One Plus Detection System (Applied Biosystems) with SYBR Green PCR Master Mix (Applied Biosystems). Each reaction mixture contained 6.75 μL of cDNA (with a concentration of 2 μg), 0.75 μL of each primer (with a concentration of 100 nM), and 6.75 μL of SYBR Green PCR Master Mix (2 × ) (Applied Biosystems). Data were analyzed utilizing the comparative cycle threshold (Ct) method (ΔΔCt), where the housekeeping gene level was used to normalize differences in sample loading and preparation. Normalization of expression levels was performed with β-actin for SYBR Green-based real-time PCR results. Each sample was analyzed in triplicate, and the results represented the n-fold difference of the transcript levels among different groups. The data were analyzed utilizing the comparative cycle threshold (Ct) (ΔΔCt) method, in which the levels of a housekeeping gene are used to normalize differences in sample loading and preparation. The normalization of expression levels was performed with β-actin. The primer sequences and TaqMan probes used in this study are presented in Tables S40–S41. Each sample was analyzed in duplicate and the results represent the n-fold difference in the transcript levels among different groups.

#### Data Analysis

Data analysis, Figures 9 and 10, was performed using GraphPad Prism ver.9.2 software (GraphPad Software, San Diego, CA, USA, www.graphpad.com). Data were expressed as the mean ± standard error of the mean (SEM) from at minimum eight samples per group for behavioral tests and four samples for molecular techniques. Means were compared with one-way analysis of variance followed by Tukey’s post hoc analysis. Comparison between groups was also performed by two-tail Student’s *t* test for independent samples. Statistical significance was considered when P-values were <0.05.

### *In Vivo* Analgesia Studies

#### Experimental Animals

Female CD-1 mice (Charles River, Barcelona, Spain) weighing 25–30 g (8–11 weeks old) were used to perform the pain studies. The mice were housed in colony cages (10 animals per cage) in a temperature-controlled room (22 ± 2 °C) with an automatic 12-h light/dark cycle (08:00–20:00). The mice were fed a standard laboratory diet and had free access to tap water until the beginning of the experiments. Envionmental enrichment (igloo and plastic tunnel) were placed in each cage to improve animal welfare. The behavioral tests were performed during the light phase (9:00–15:00). The mice were randomized to treatment groups, testing a balanced number of animals from several groups each day. Random testing was also conducted throughout the estrous cycle. The mice were handled in accordance with international standards (European Communities Council directive 2010/63), and the experimental protocols were approved by regional (Junta de Andalucía) and institutional (Research Ethics Committee of the University of Granada) authorities.

#### Drugs and Drugs Administration

The effects of the drugs tested on nociception were studied in a model of mechanical hypersensitiviy induced by capsaicin. The algogenic agent, capsaicin (Sigma-Aldrich Química S.A.), was dissolved in 1% DMSO in physiological sterile saline to a concentration of 0.05 μg/μL (i.e., 1 μg per mouse). Capsaicin solution was injected intraplantarly (i.pl.) into the right hind paw proximate to the heel, in a volume of 20 μL using a 1710 TLL Hamilton microsyringe (Teknokroma, Barcelona, Spain) with a 30^1/2^-gauge needle. Control animals were injected with the same volume of the vehicle of capsaicin.

Drug solutions were prepared immediately before the start of the experiments. In addition to our compound **12d**, we used CR4056 (**1**), synthesized following described procedures,^31^ as a control prototypical I_2_–IRs agonist.^13^ Clonidine (Sigma-Aldrich Química S.A.) was used as a prototypical α_2_ agonist, with low affinity for imidazoline receptors.^60^ Gabapentin (Abcam, UK), was used as a control drug active on central sensitization with known antinociceptive effects.^55^ In some experiments, the I_2_/α_2_ antagonist idazoxan (**4**)^57^ (Sigma-Aldrich Química S.A.) or ist methoxy analog, the α_2-_AR agonist RX 821002 (Biogen Científica S.L., Madrid, Spain) were associated with other treatments to determine the mechanism involved in the antiallodynic effects.^80^**12d**, CR4056 (**1**), and gabapentin were dissolved in DMSO 10% and cyclodextrin 40% in saline. Clonidine, idazoxan (**4**) and RX 821002 were dissolved in physiological sterile saline. CR4056 (**1**) was orally (p.o.) administrated in a volumen of 10 mL/kg by oral gavage. The other drugs were administered subcutaneously (s.c.) in a volume of 5 mL/kg into the interscapular area. When the effect of the association of several drugs was assessed, each s.c. administration was performed in different areas of the interscapular zone to avoid the mixture of the drug solutions and any physicochemical interaction between them. The researchers who performed the experiments were blinded to the treatment received by each animal.

#### Evaluation of Capsaicin-Induced Secondary Mechanical Hypersensitivity

Animals were placed into individual test compartments for 2 h before the behavioral evaluation to habituate them to the test conditions. The compounds tested, or their vehicles, were administered as described above, and 30 min after drug administration, capsaicin or DMSO 1%, was intraplantarly (i.pl.) administered. Punctate mechanical stimulation was applied with a dynamic plantar aesthesiometer (Ugo Basile, Varese, Italy) at 15 min after the administration of capsaicin or its solvent (i.e., drug effects were evaluated 45 min after drug administration). Briefly, a nonflexible filament (0.5 mm diameter) was electronically driven into the ventral side of the paw previously injected with capsaicin or solvent (i.e., the right hind paw), at least 5 mm away from the site of the injection toward the fingers. The intensity of the stimulation was fixed at 0.5 g force, as described previously.^54^ When a paw withdrawal response occurred, the stimulus was automatically terminated, and the latency of the behavioral response was automatically recorded. The filament was applied three times, separated by intervals of 0.5 min, and the mean value of the three trials was considered the withdrawal latency of the animal. A cutoff time of 50 s was used.

#### Rotarod Test

Motor coordination was assessed with an accelerating rotarod (Cibertec, Madrid, Spain), as previously described.^81^ Briefly, mice were required to walk against the motion of an elevated rotating drum at increasing speed (4–80 rpm over 5 min), and the latency to fall down was recorded using a cutoff time of 300 s. Mice were given three training sessions 24 h before drug testing. On the day of the test, rotarod latencies were measured immediately before administration of the drug or their solvent (time 0) and then several times (45, 90, 120, and 240 min) after the s.c. injection. As a comparison drug we used gabapentin (40 mg/kg, s.c.).

## Supporting Information Available

^1^H and ^13^C NMR spectra, tables of representative data and discussion on the assignation of the signals; X-ray crystallography data of compounds **12b**, **12d**, and **12h**; 3D-QSAR study and theoretical ADMET parameters; PAMPA-BBB studies; experimental details on physicochemical properties, solubility, chemical stability at different pHs of **12d**; complete details of *in vitro* biological methods of **12d**: cytotoxicity assays, Caco-2 permeability, microsomal stability, plasma stability, protein binding, cytochrome inhibition, and hERG ion channel inhibition; complete details of experimental procedures/tables for *in vivo* experiments: pharmacokinetics, neurodegenerative preclinical model, SAMP8 studies, and capsaicin-induced mechanical hypersensitivity; molecular formula string and data (PDF)

## Abbreviations

I_2_–IRs: imidazoline I_2_ receptors
5xFAD: murine familial AD model
α_2_-AR: α_2_-adrenergic receptor
FADD: Fas-associated protein with death domain
NORT: Novel Object Recognition
OLT: Novel Object Location Test
SAMP8: mouse model of accelerated aging and AD
*K*_i_: inhibition constant
MoA: mechanism of action
