# Supplementary material for: Discovery of (3-Phenylcarbamoyl-3,4-dihydro-2H-pyrrol-2-yl)phosphonates as Imidazoline I2 Receptor Ligands with Anti-Alzheimer and Analgesic Properties
Source: J Med Chem. 2025 Jan 17;68(3):2551–73. doi: 10.1021/acs.jmedchem.4c01644 (PMC11831594; doi:10.1021/acs.jmedchem.4c01644)
Supplement: Supplementary file 1 — jm4c01644_si_001.pdf [file jm4c01644_si_001.pdf]

Discovery of (3-phenylcarbamoyl-3,4-dihydro-2*H*-pyrrol-2-yl)phosphonates as imidazoline I_2 receptor ligands with anti-Alzheimer and analgesic properties.

Andrea Bagán[1,2], Alba López-Ruiz[1,2], Sònia Abás[1], M. Carmen Ruiz-Cantero[1], Foteini Vasilopoulou[3], Teresa Taboada-Jara[3], Christian Griñán-Ferré[3,4], Mercè Pallàs[3,4], Carolina Muguruza[5], Rebeca Diez-Alarcia[5,6], Luis F. Callado[5,6], José M. Entrena[7], Enrique J. Cobos[8], Belén Pérez[9], José A. Morales-García[10], Elies Molins[11], Steven De Jonghe[12], Dirk Daelemans[13], José Brea[14], Cristina Val[14], M. Isabel Loza[14], Elena Hernández-Hernández[15,†], Jesús A. García-Sevilla[15], M. Julia García-Fuster[15], Caridad Díaz[16], Rosario Fernández-Godino[16], Olga Genilloud[16], Milan Beljkaš[17], Slavica Oljačić[17], Katarina Nikolic[17], Carmen Escolano[1, 2*].

[1]Laboratory of Medicinal Chemistry (Associated Unit to CSIC), Department of Pharmacology, Toxicology and Medicinal Chemistry, Faculty of Pharmacy and Food Sciences, University of Barcelona, Av. Joan XXIII, 27-31, 08028 Barcelona, Spain.

[2]Institute of Biomedicine of the University of Barcelona (IBUB), University of Barcelona, 08028 Barcelona, Spain.

[3]Pharmacology Section, Toxicology and Medicinal Chemistry, Faculty of Pharmacy and Food Sciences, and Institut de Neurociències, University of Barcelona, Av. Joan XXIII, 27-31. 08028 Barcelona, Spain.

[4]Centro de Investigación Biomédica en Red Enfermedades Neurodegenerativas (CiberNed), National Institute of Health Carlos III, 28029 Madrid, Spain.

[5]Department of Pharmacology, University of the Basque Country, UPV/EHU, 48940 Leioa, Bizkaia, and Centro de Investigación Biomédica en Red de Salud Mental, CIBERSAM, Spain.

[6] BioBizkaia Health Research Institute, Barakaldo, Bizkaia, Spain.

[7]Animal Behavior Research Unit, Scientific Instrumentation Center, University of Granada, Parque Tecnológico de Ciencias de la Salud, 18100 Armilla, Granada, Spain.

[8]Department of Pharmacology, Faculty of Medicine and Biomedical Research Center (Neurosciences Institute), Biosanitary Research Institute ibs. Granada, University of Granada, 18016 Granada, Spain.

[9]Department of Pharmacology, Therapeutic and Toxicology. Autonomous University of Barcelona, 08193 Cerdanyola. Spain.

[10]Department of Cell Biology. Faculty of Medicine, Computense University of Madrid. (UCM), 28040 Madrid, Spain.

[11]Institut de Ciència de Materials de Barcelona (CSIC), Campus UAB, 08193 Cerdanyola, Spain.

[12]Molecular, Structural and Translational Virology Research Group, Rega Institute for Medical Research, Department of Microbiology, Immunology and Transplantation, Katholieke Universiteit Leuven, 3000 Leuven, Belgium.

[13]Molecular Genetics and Therapeutics in Virology and Oncology Research Group, Rega Institute for Medical Research, Department of Microbiology, Immunology and Transplantation, Katholieke Universiteit Leuven, 3000 Leuven, Belgium.

[14]Drug Screening Platform/Biofarma Research Group, CIMUS Research Center, University of Santiago de Compostela (USC), 15782 Santiago de Compostela, Spain.

[15] IUNICS, University of the Balearic Islands (UIB), and IdISBa, Cra. Valldemossa km 7.5, 07122 Palma de Mallorca, Spain.

[16]Fundación MEDINA Centro de Excelencia en Investigación de Medicamentos Innovadores de Andalucía, Avda. Del Conocimiento 34, 10016 Ganada, Spain.

[17] Department of Pharmaceutical Chemistry, Faculty of Pharmacy, University of Belgrade, Vojvode Stepe 450, 11000 Belgrade, Serbia.

*Corresponding author.

E-mail address: cescolano@ub.edu (Carmen Escolano).

CONTENTS

^{1}H-NMR and ^{13}C-NMR spectra for compounds 12a-12o S3
Representative data of ^{1}H-NMR spectra of new compounds S18
Representative data of ^{13}C-NMR spectra of new compounds S19
Discussion on the spectra data S20
HPLC-MS reports S21
X-ray crystallographic data for 12b, 12d and 12h S31
I_1-IR Binding Activity S68
3D-QSAR study and physicochemical parameters S69
In vitro Blood-Brain Barrier Permeation Assay S74
Cytotoxicity assays S76
Solubility S76
Chemical stability assay at different pHs S76
Caco-2 permeability assay S79
Microsomal stability at human and mice microsomes S81
Plasma stability S82
Human and mouse plasma protein binding S83
Cytochromes inhibition S84
hERG ion channel inhibition S86
In vitro effects of 12d in a preclinical model of neurodegeneration and neuroinflammation S86
Pharmacokinetics S87
MoA Alzheimer's panel (Eurofins) S94
Western Blot Analysis for Neurochemical Markers S96
RNA extraction and gene expression determination S96
Reduction of capsaicin-induced mechanical hypersensitivity by CR4056 in mice, and contribution of imidazoline receptor-2 (I_2R) to its effects S97
Molecular Formula Strings (SMILES) S98
References S103

^{1}H-NMR and ^{13}C-NMR spectra for compounds 12a-12o

Diethyl [(2*RS*,3*RS*)-3-(phenylcarbamoyl)-3,4-dihydro-2*H*-pyrrol-2-yl]phosphonate, 12a.

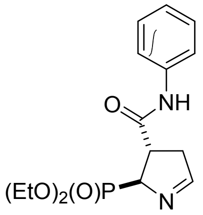

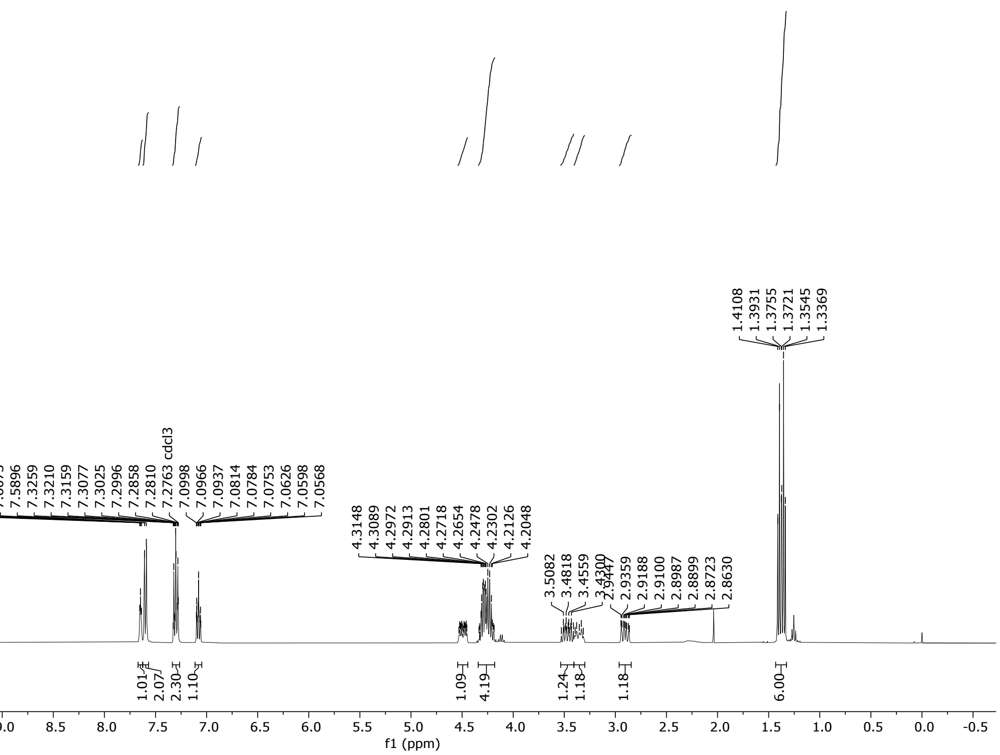

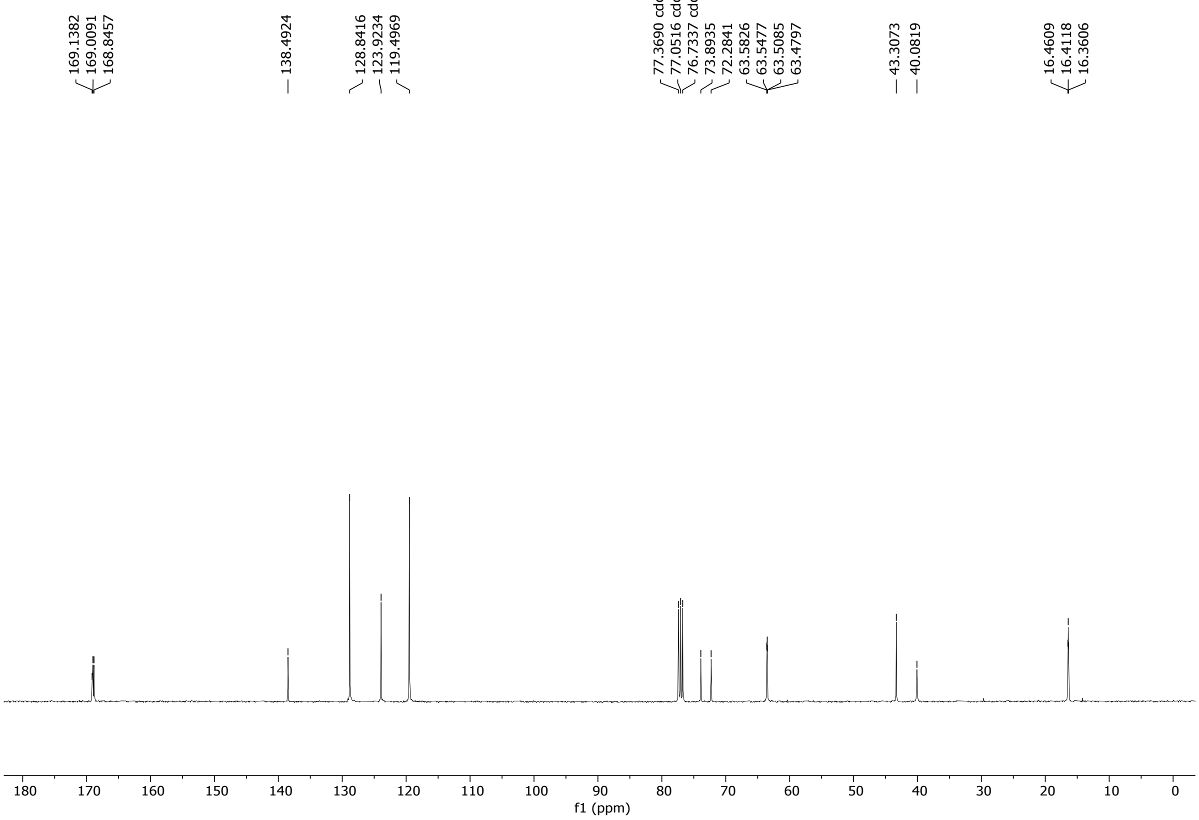

Diethyl [(2*RS*,3*RS*)-3-[(3-chloro-4-fluorophenylcarbamoyl)-3,4-dihydro-2*H*-pyrrol-2-yl]phosphonate, 12b.

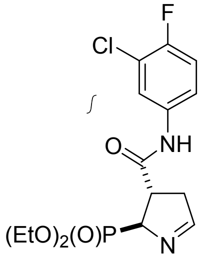

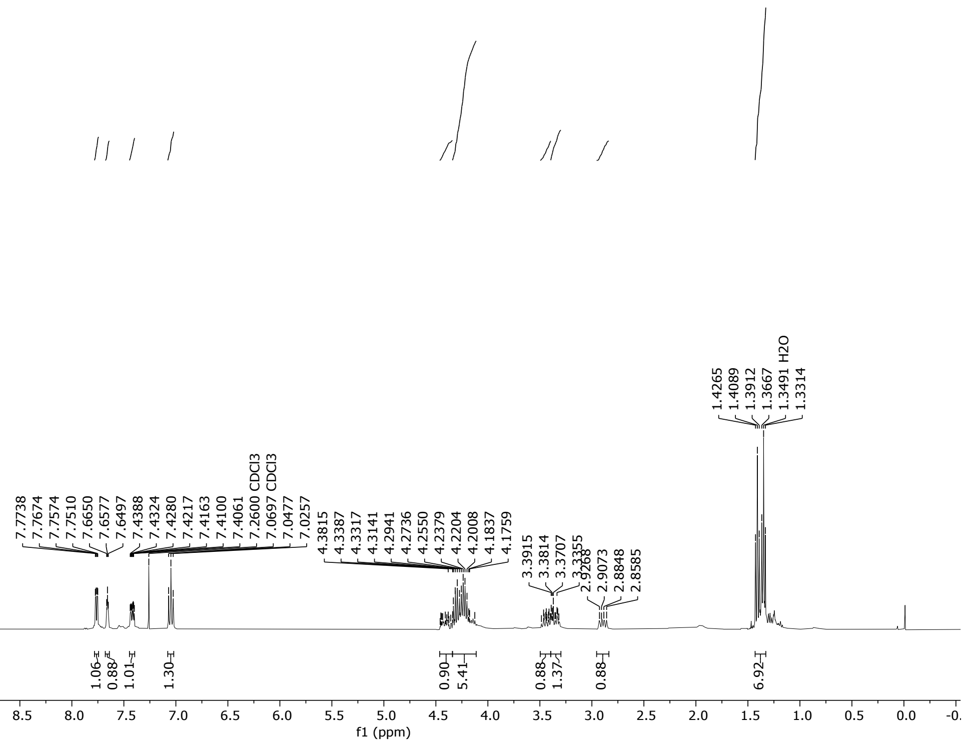

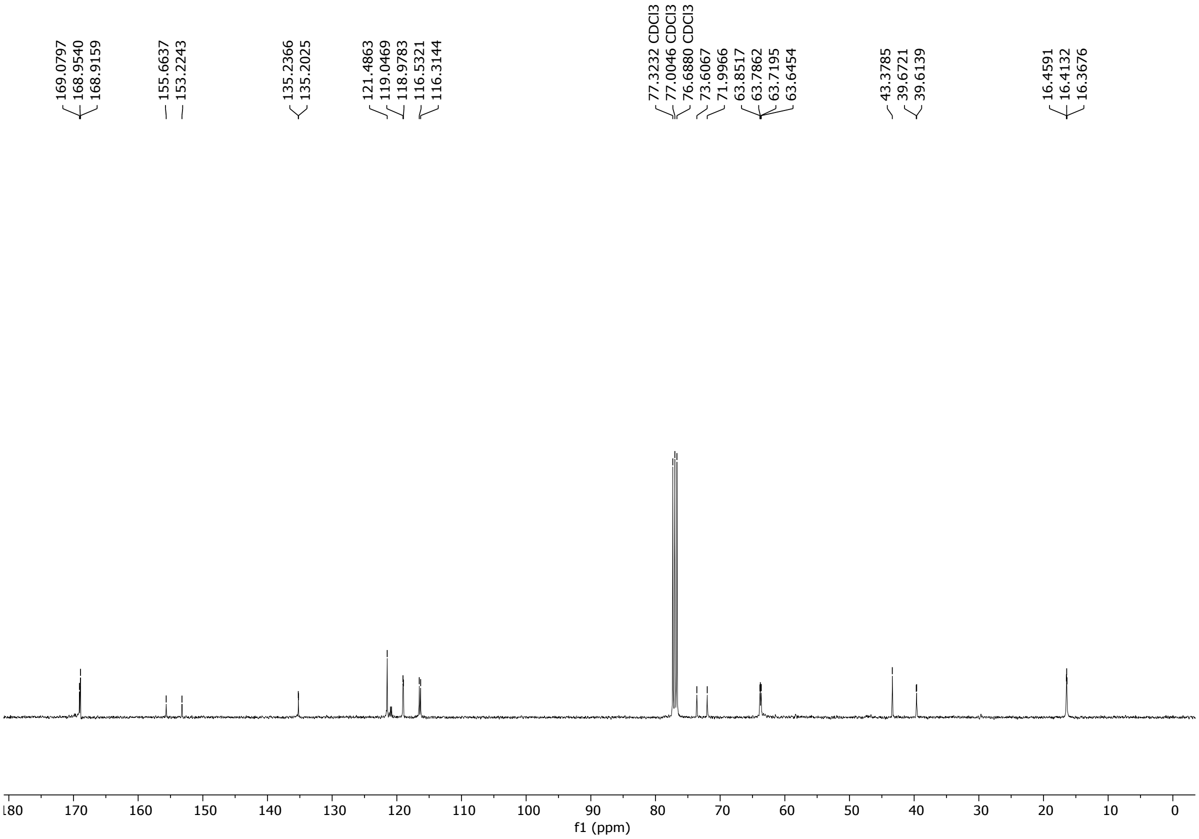

Diethyl [(2*RS*,3*RS*)-2-phenyl-3-(phenylcarbamoyl)-3,4-dihydro-2*H*-pyrrol-2-yl]phosphonate, 12c.

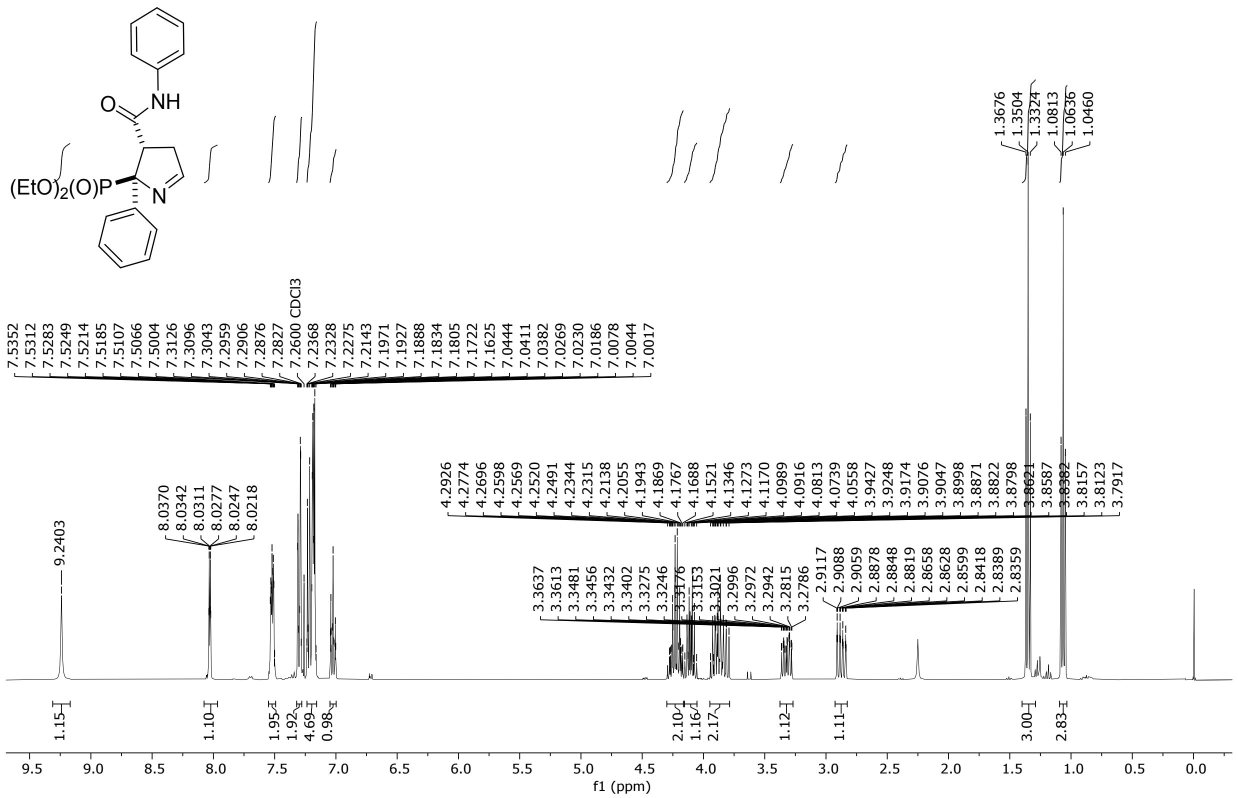

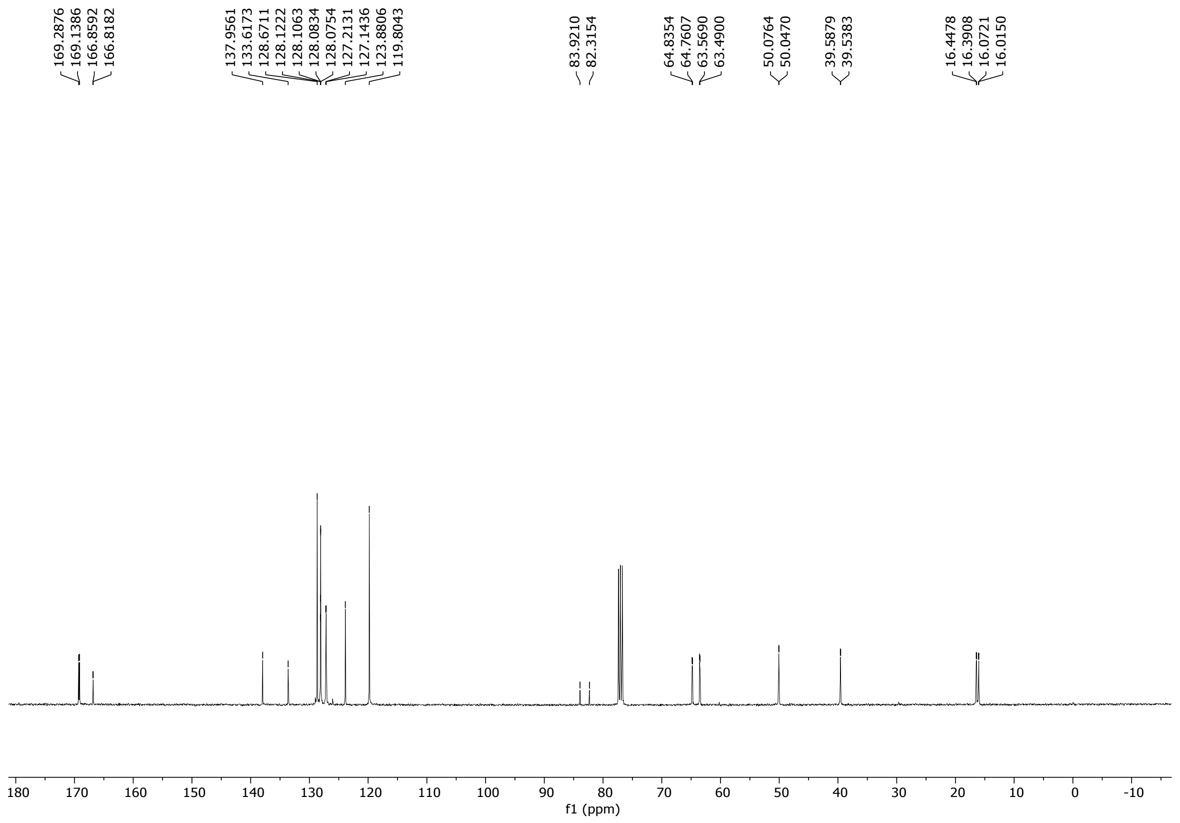

Diethyl [(2*RS*,3*RS*)-3-((3-chloro-4-fluorophenyl)carbamoyl)-2-phenyl-3,4-dihydro-2*H*-pyrrol-2-yl]phosphonate, 12d.

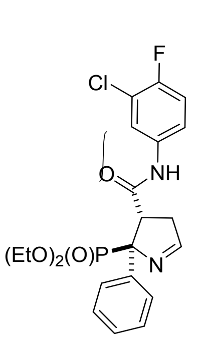

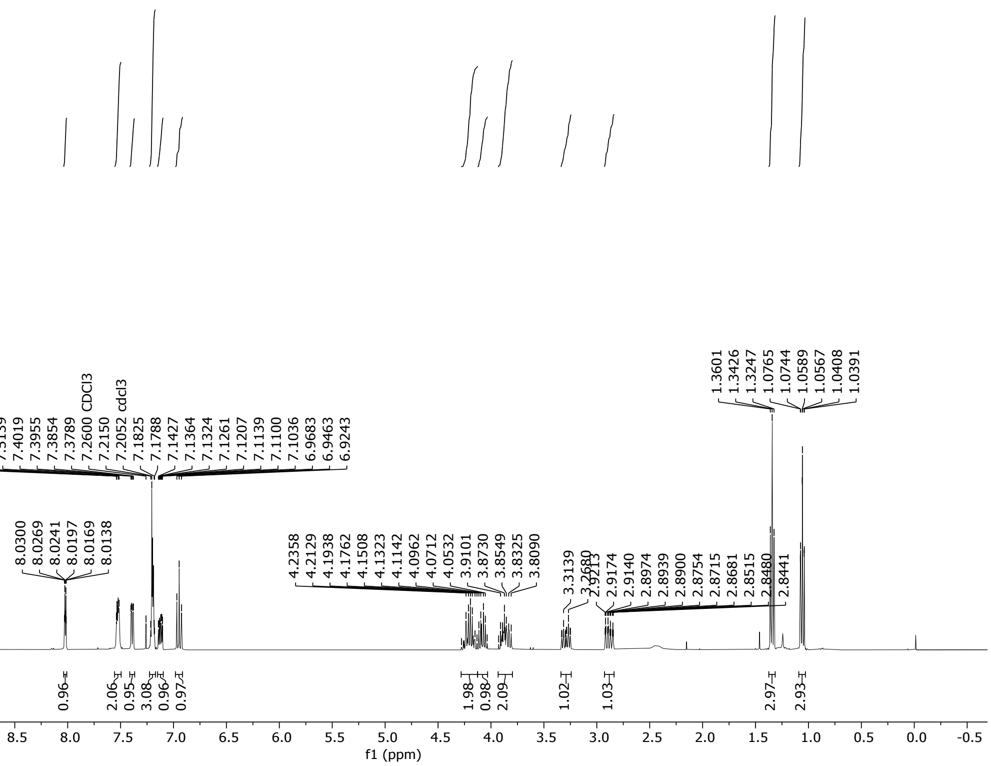

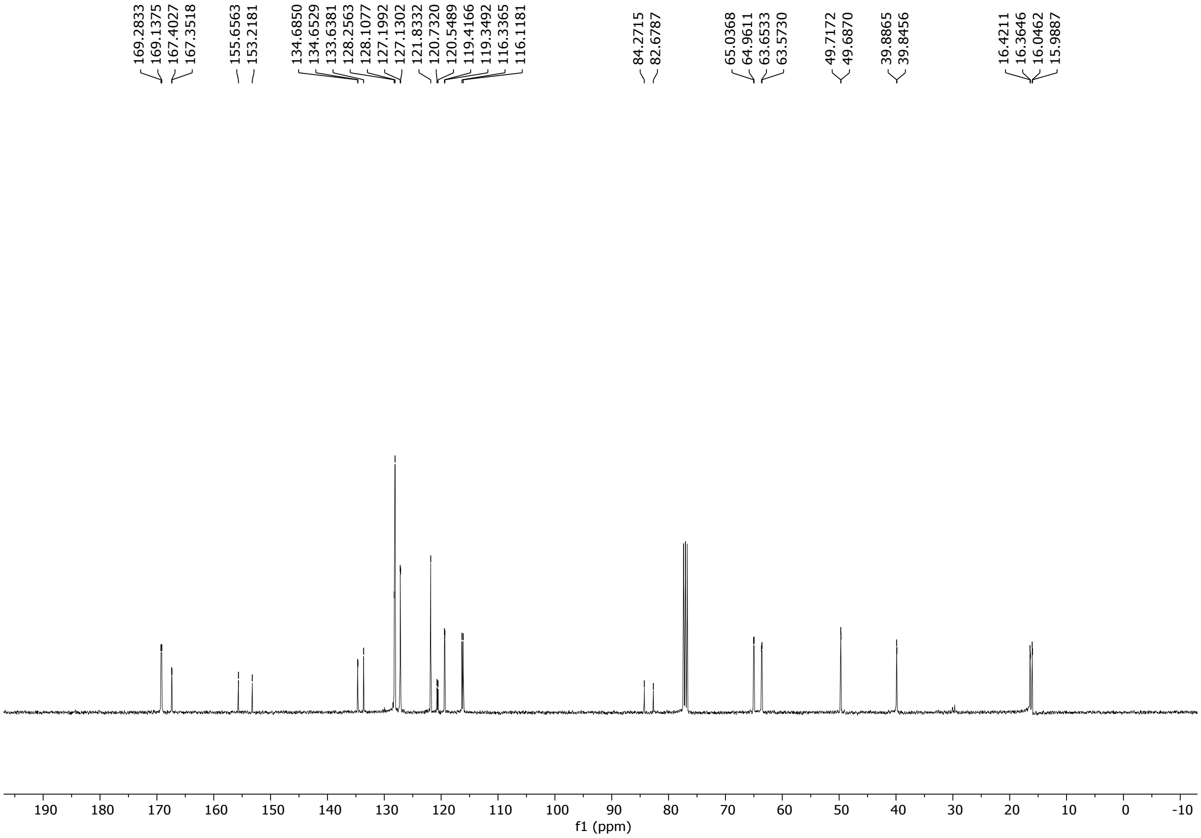

Diethyl [(2*RS*,3*RS*)-3-((4-chlorophenyl)carbamoyl)-2-phenyl-3,4-dihydro-2*H*-pyrrol-2-yl]phosphonate, 12e.

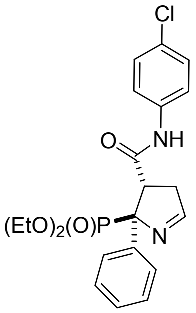

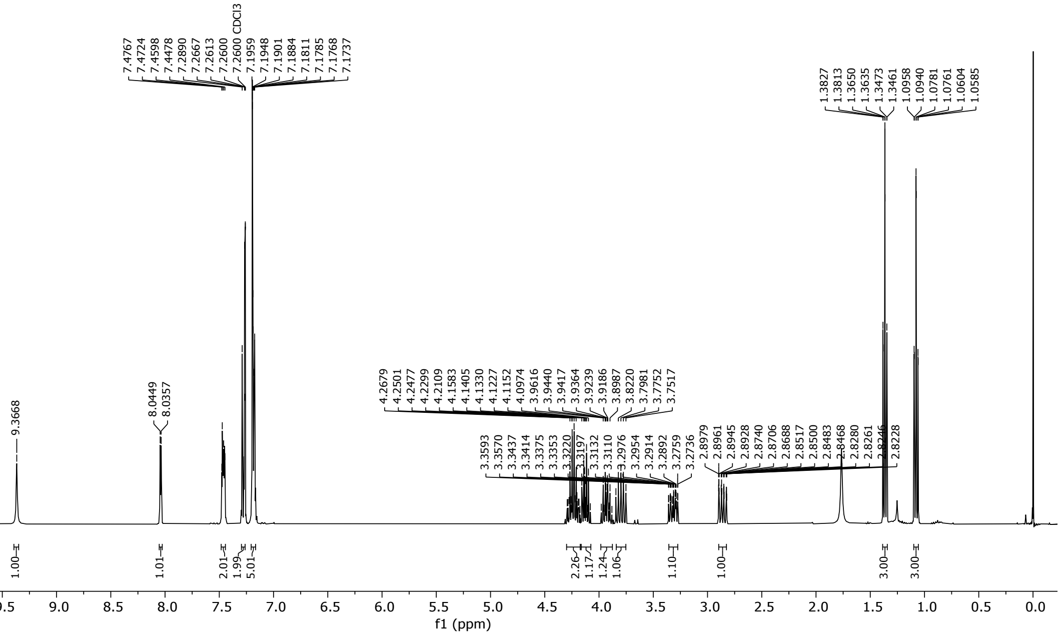

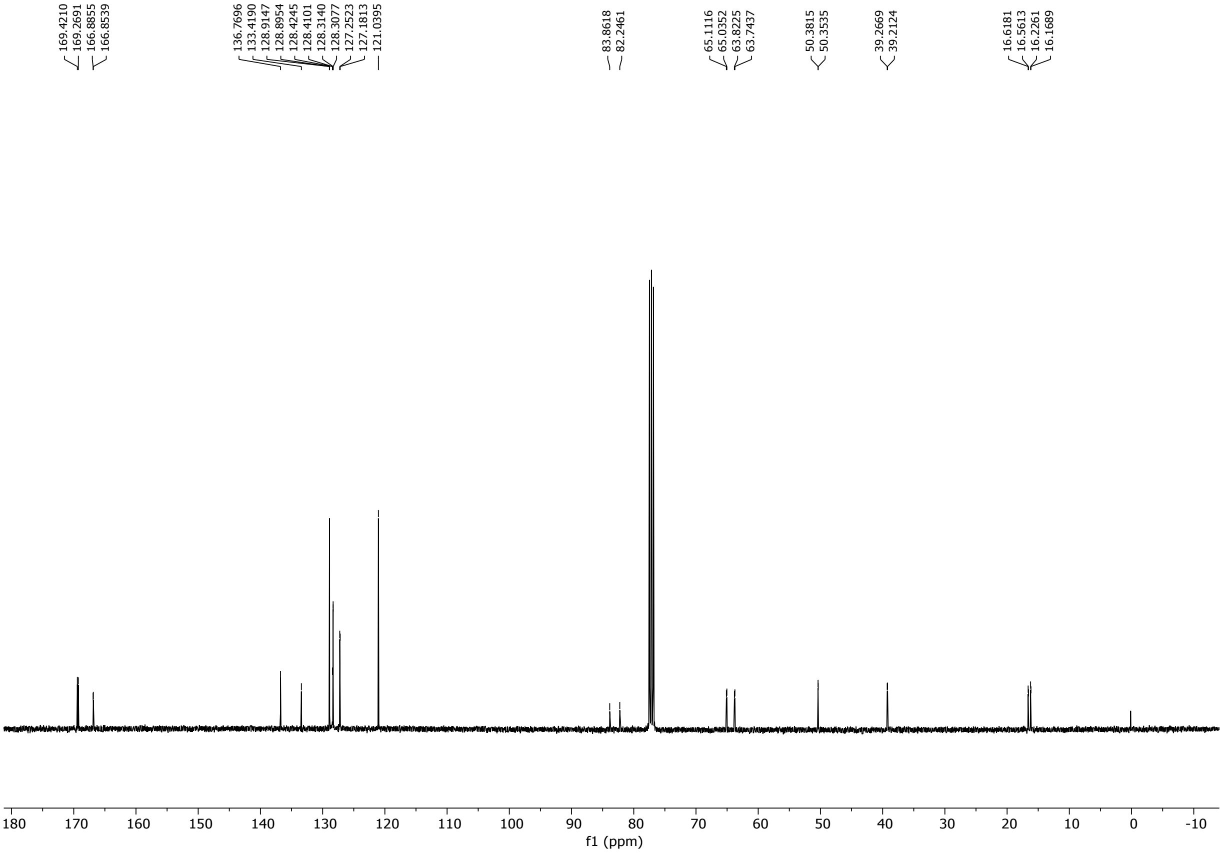

Diethyl [(2*RS*,3*RS*)-3-([1,1'-biphenyl]-4-ylcarbamoyl)-2-phenyl-3,4-dihydro-2*H*-pyrrol-2-yl]phosphonate, 12f.

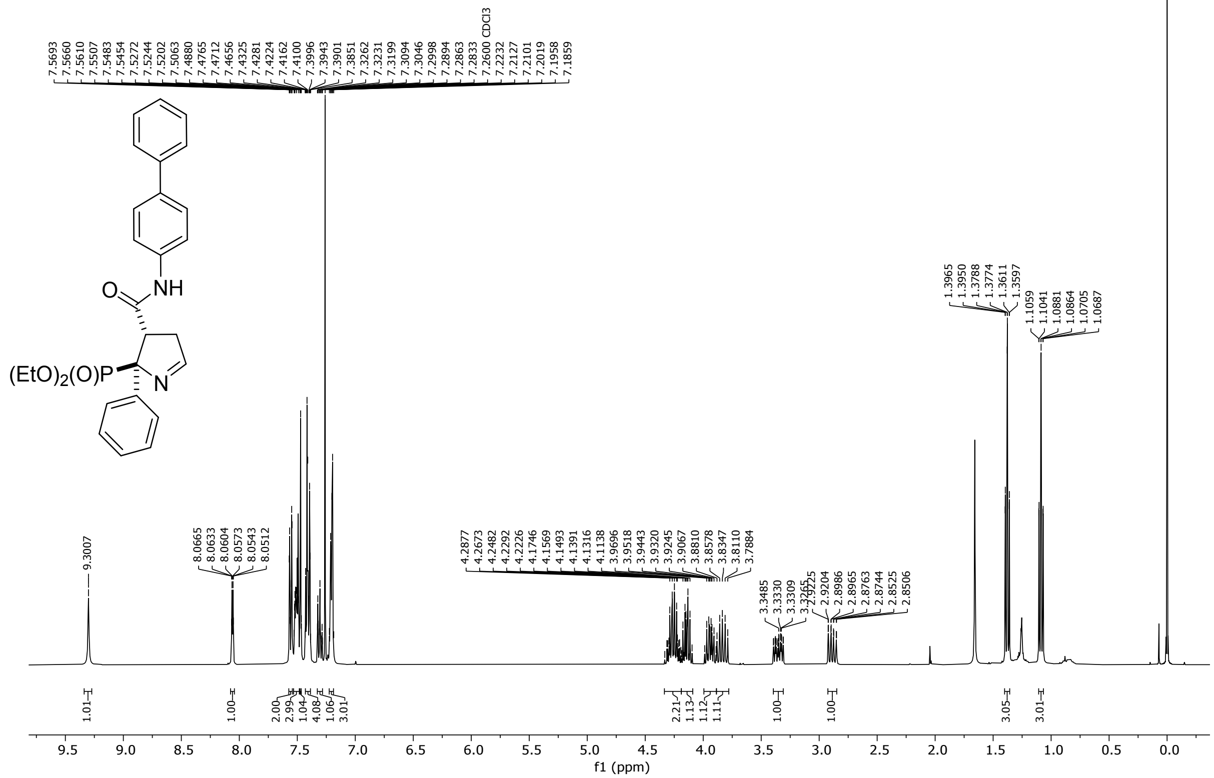

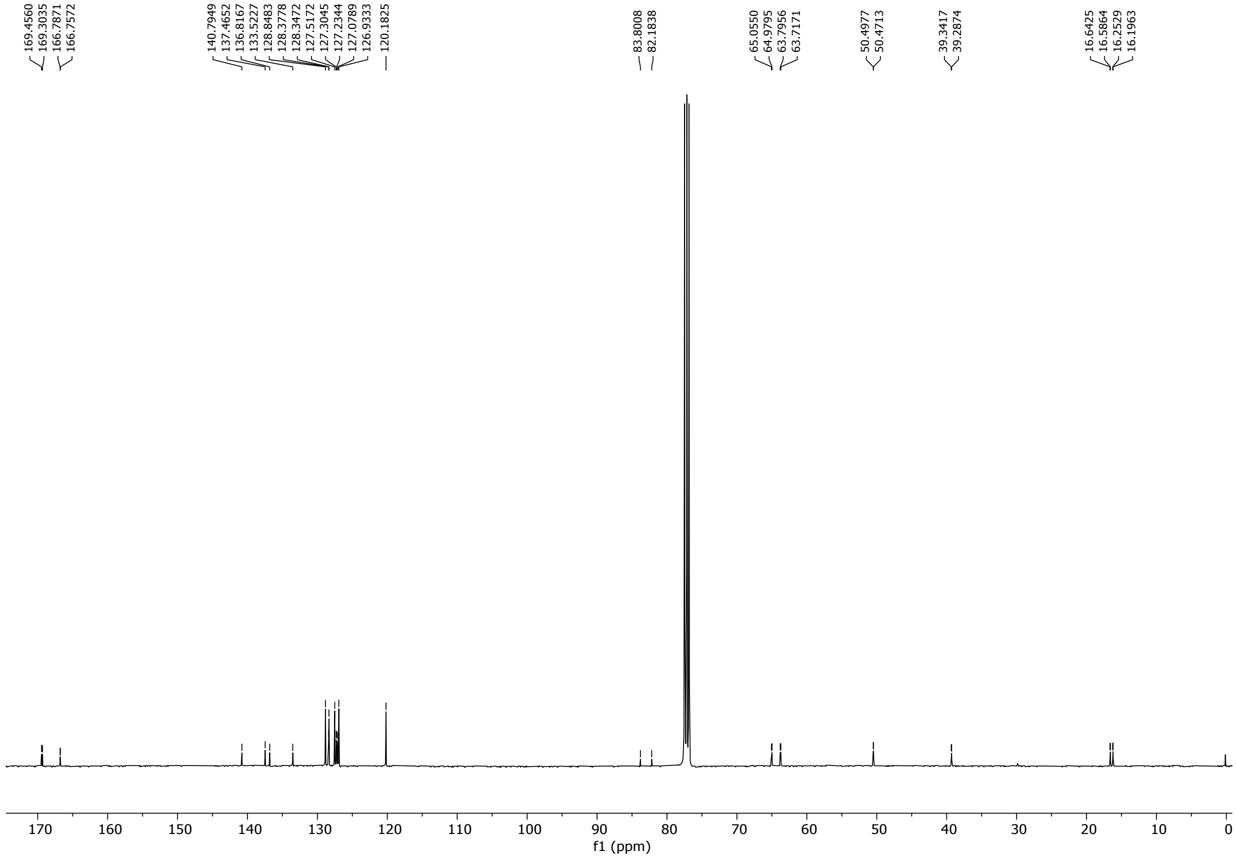

Diethyl [(2*RS*,3*RS*)-2-phenyl-3-((4-(trifluoromethyl)phenyl)carbamoyl)-3,4-dihydro-2*H*-pyrrol-2-yl]phosphonate, 12g.

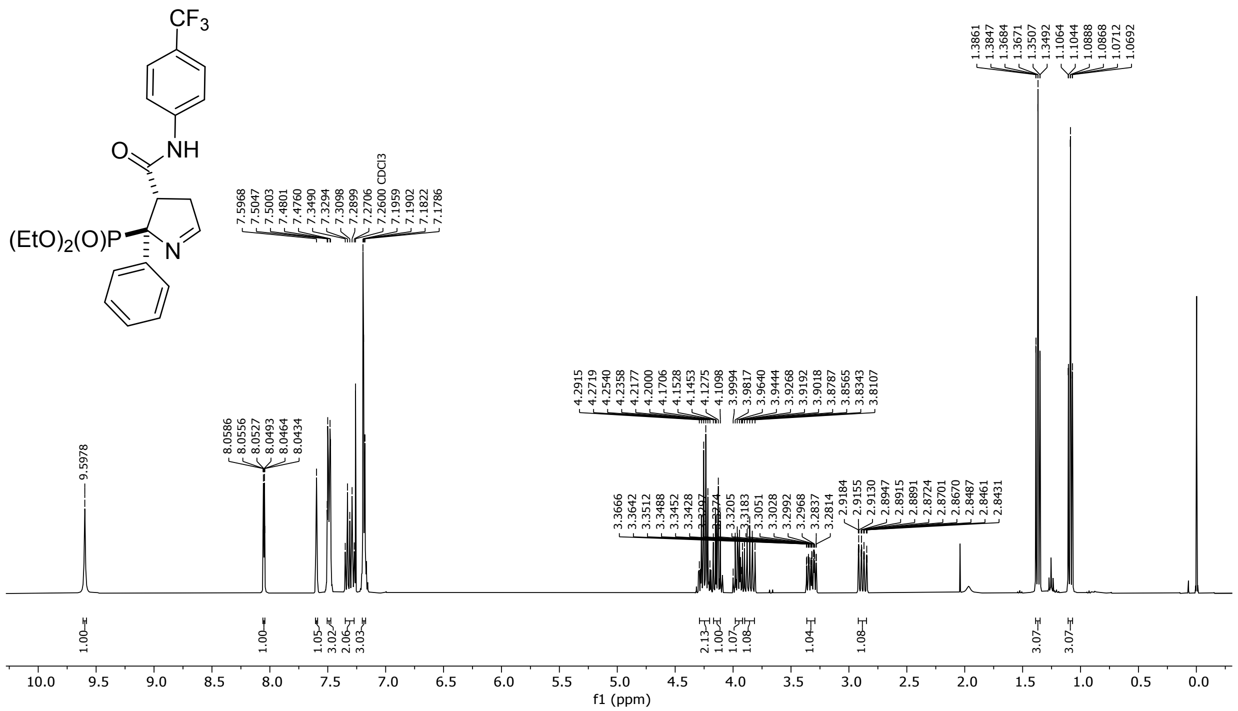

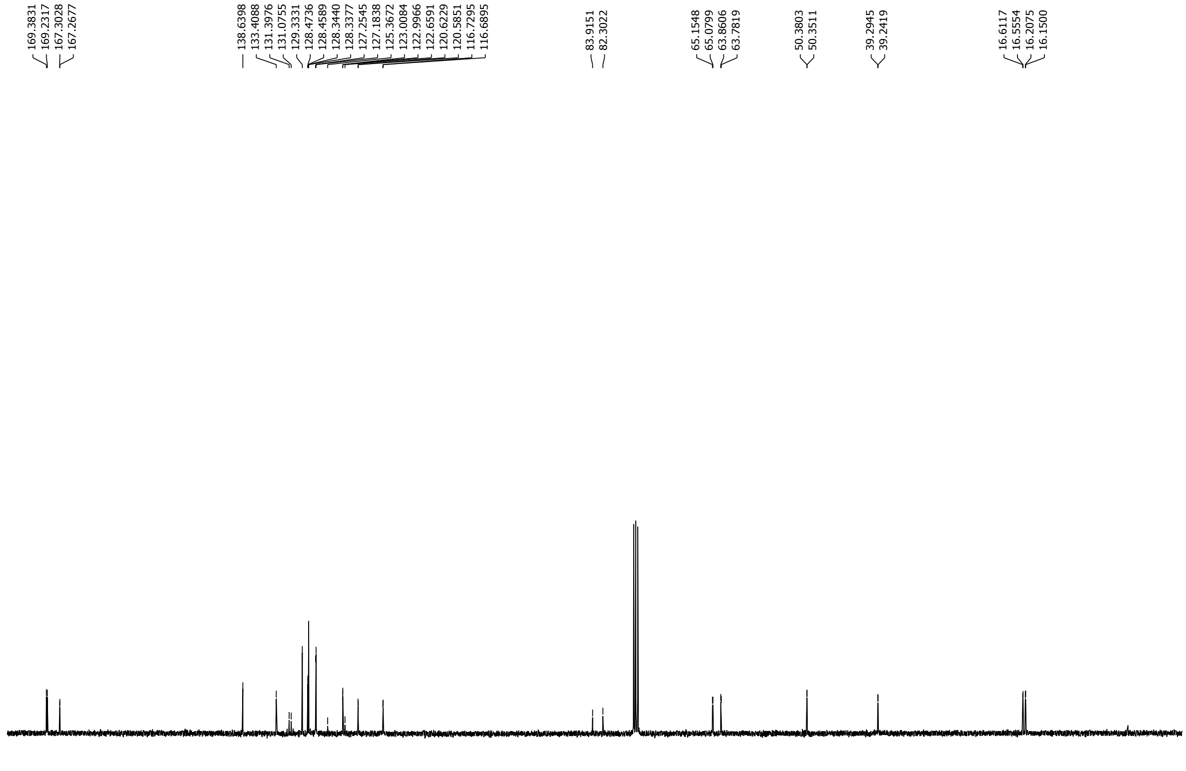

Diethyl [(2*RS*,3*RS*)- 3-(cyclohexylcarbamoyl)-2-phenyl-3,4-dihydro-2*H*-pyrrol-2-yl]phosphonate, 12h.

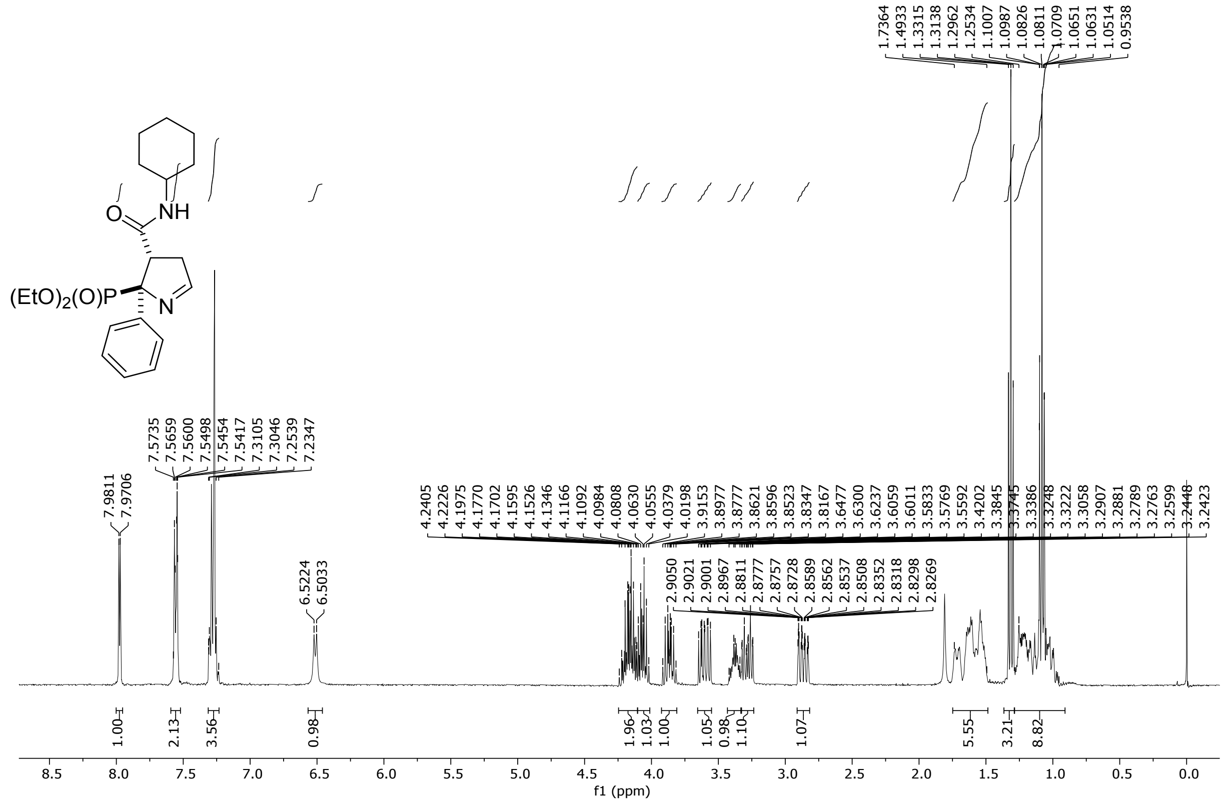

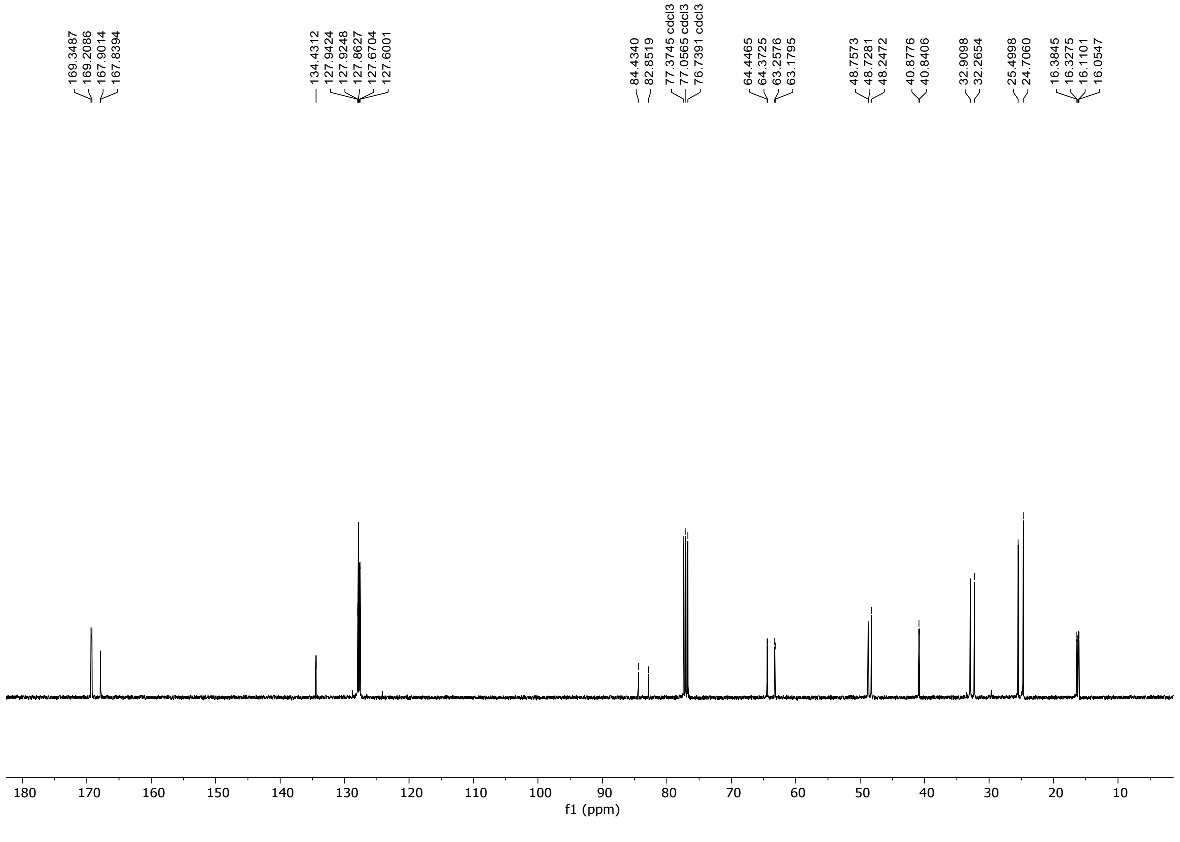

Diethyl [(2*RS*,3*RS*)-2-(4-fluorophenyl)-3-(phenylcarbamoyl)-3,4-dihydro-2*H*-pyrrol-2-yl]phosphonate, 12i.

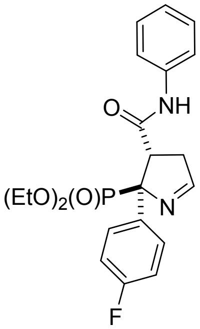

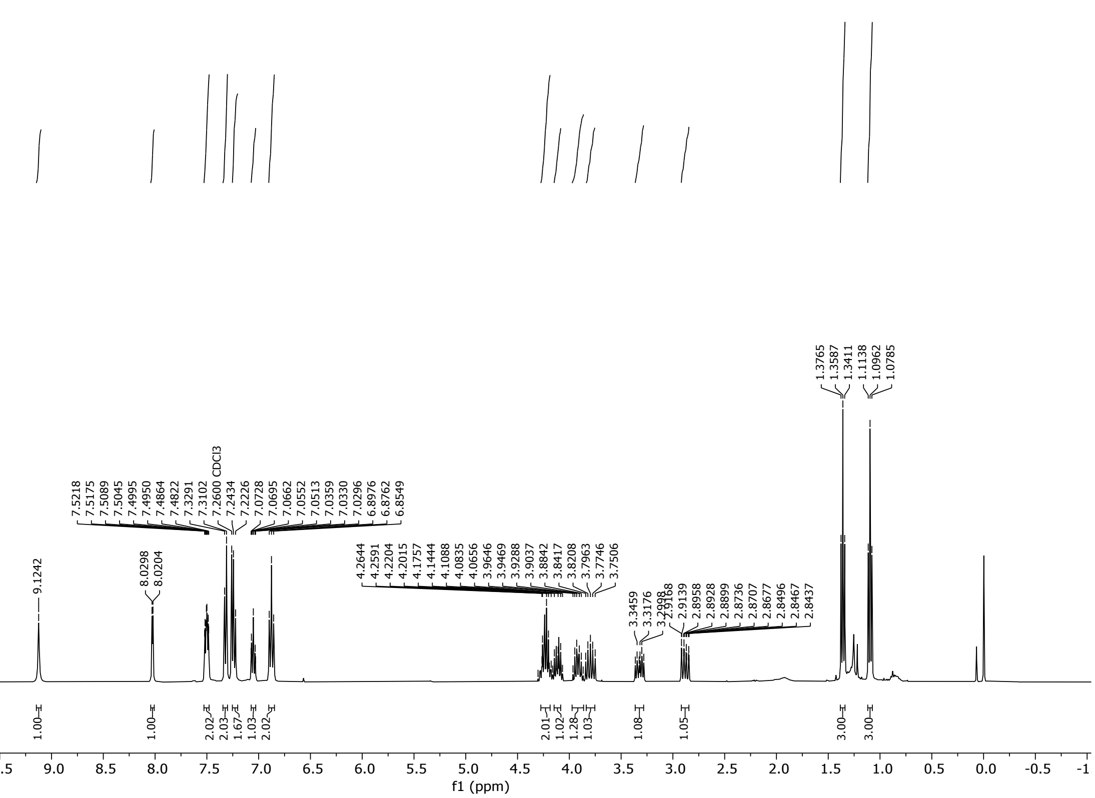

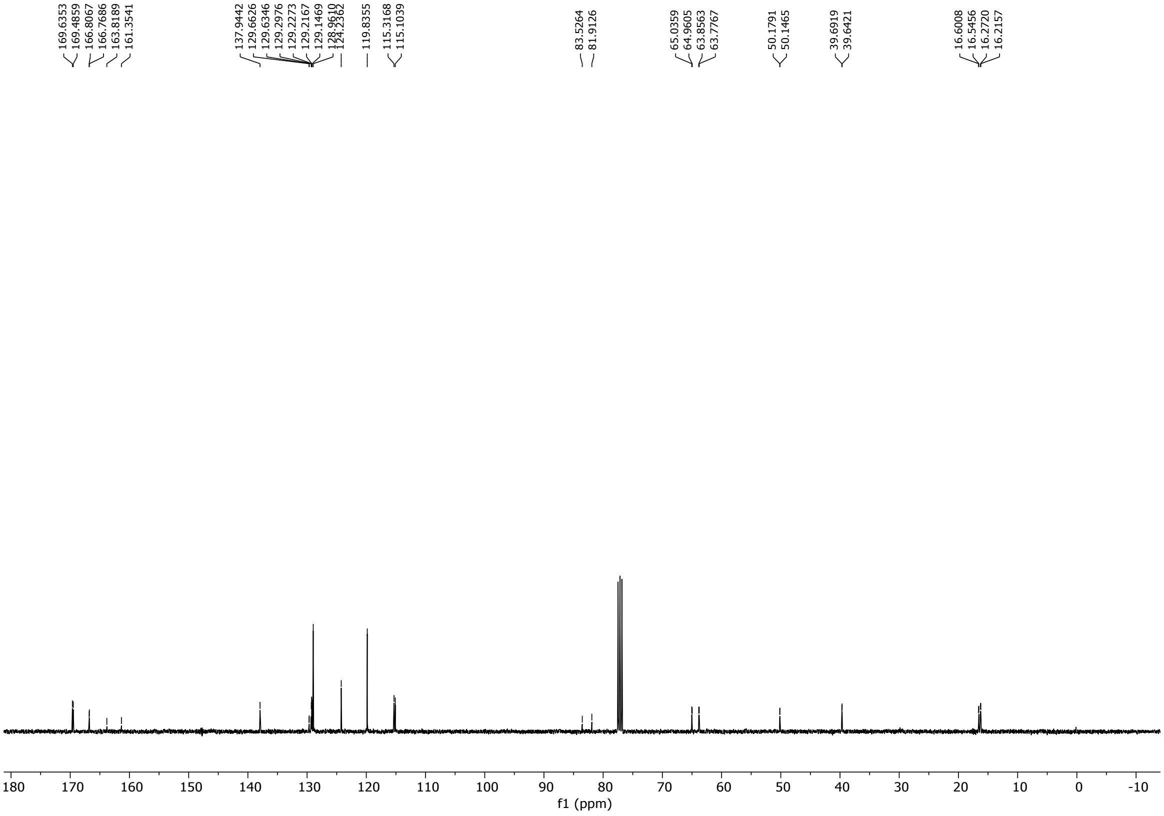

Diethyl [(2*RS*,3*RS*)-3-((3-chloro-4-fluorophenyl)carbamoyl)-2-(4-fluorophenyl)-3,4-dihydro-2*H*-pyrrol-2-yl]phosphonate, 12j.

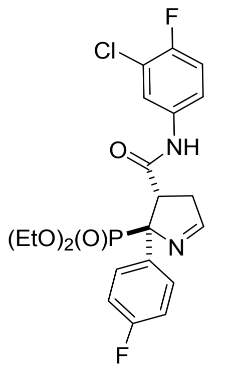

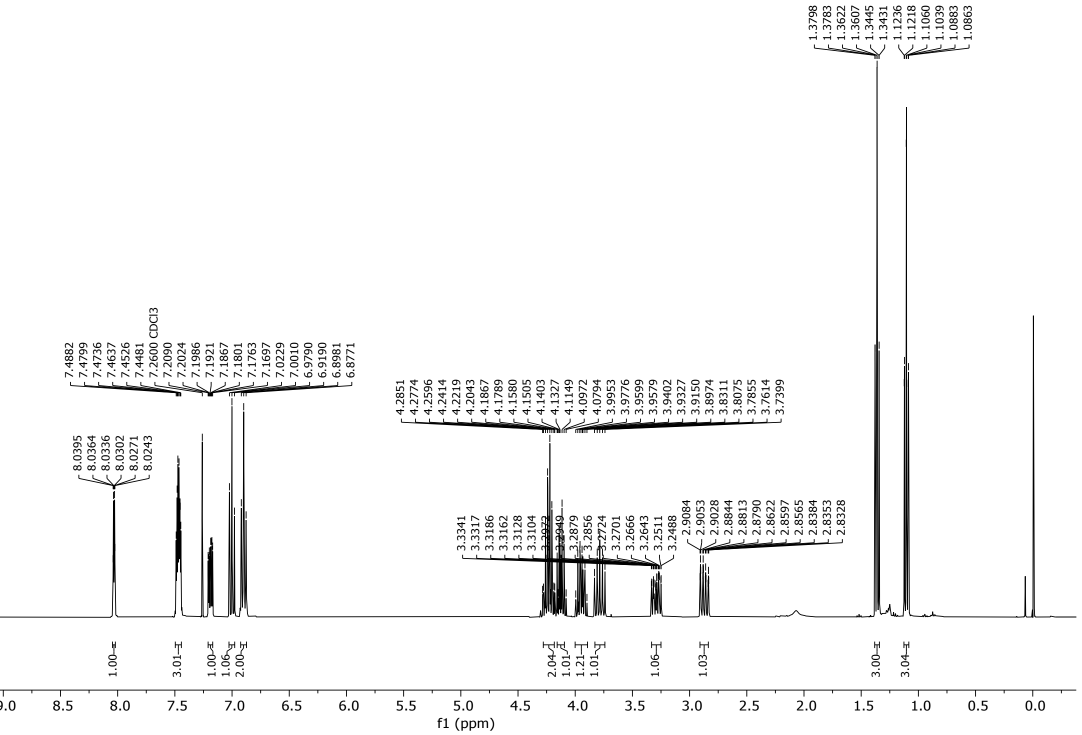

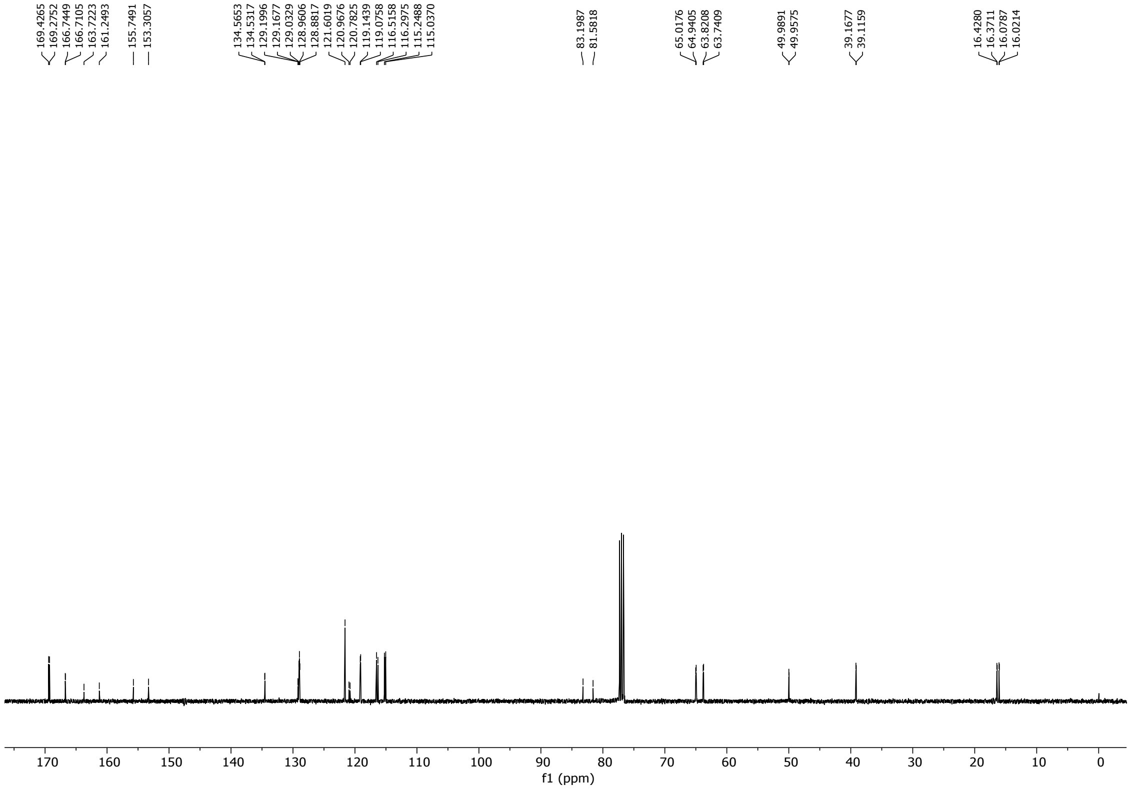

Diethyl [(2*RS*,3*RS*)-2-(4-methoxyphenyl)-3-(phenylcarbamoyl)-3,4-dihydro-2*H*-pyrrol-2-yl]phosphonate, 12k.

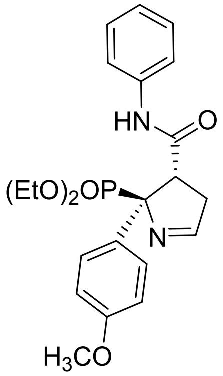

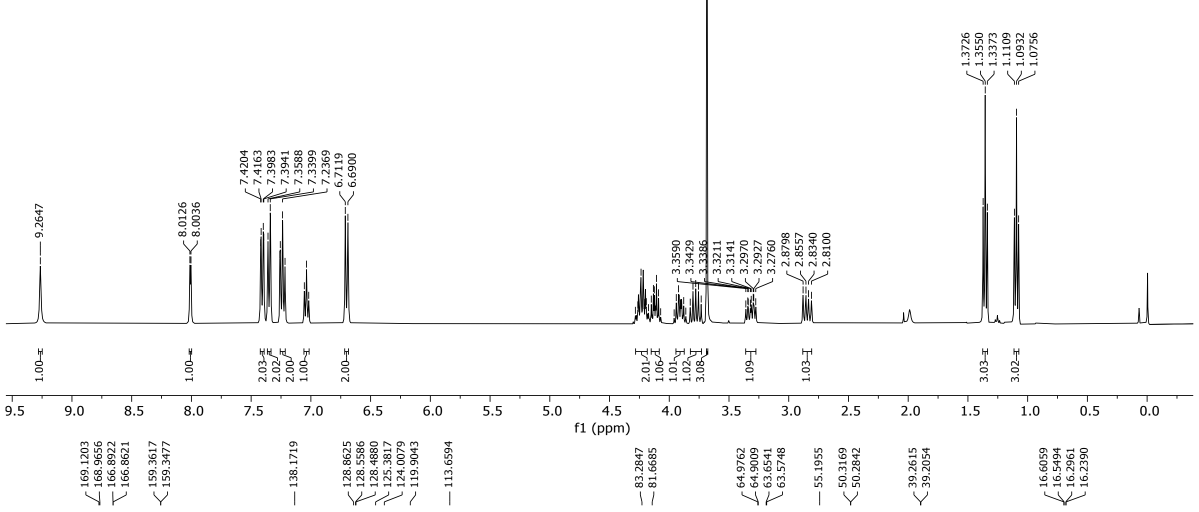

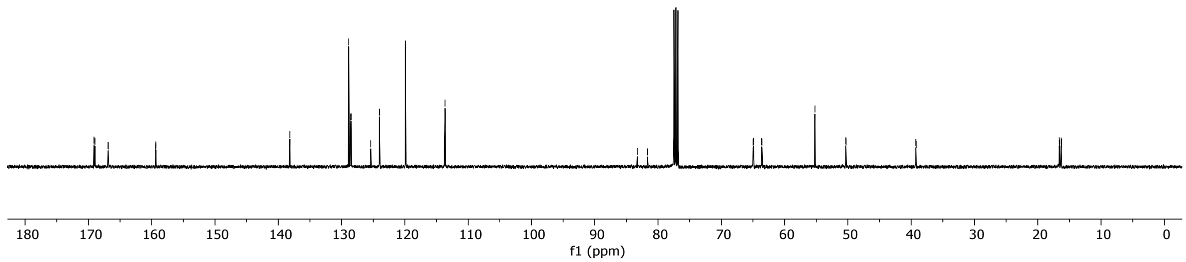

Diethyl [(2*RS*,3*RS*)-3-((3-chloro-4-fluorophenyl)carbamoyl)-2-(4-methoxyphenyl)-3,4-dihydro-2*H*-pyrrol-2-yl]phosphonate, 12l.

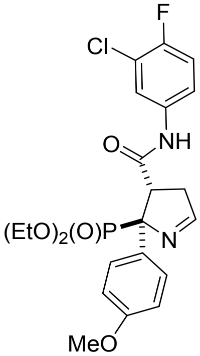

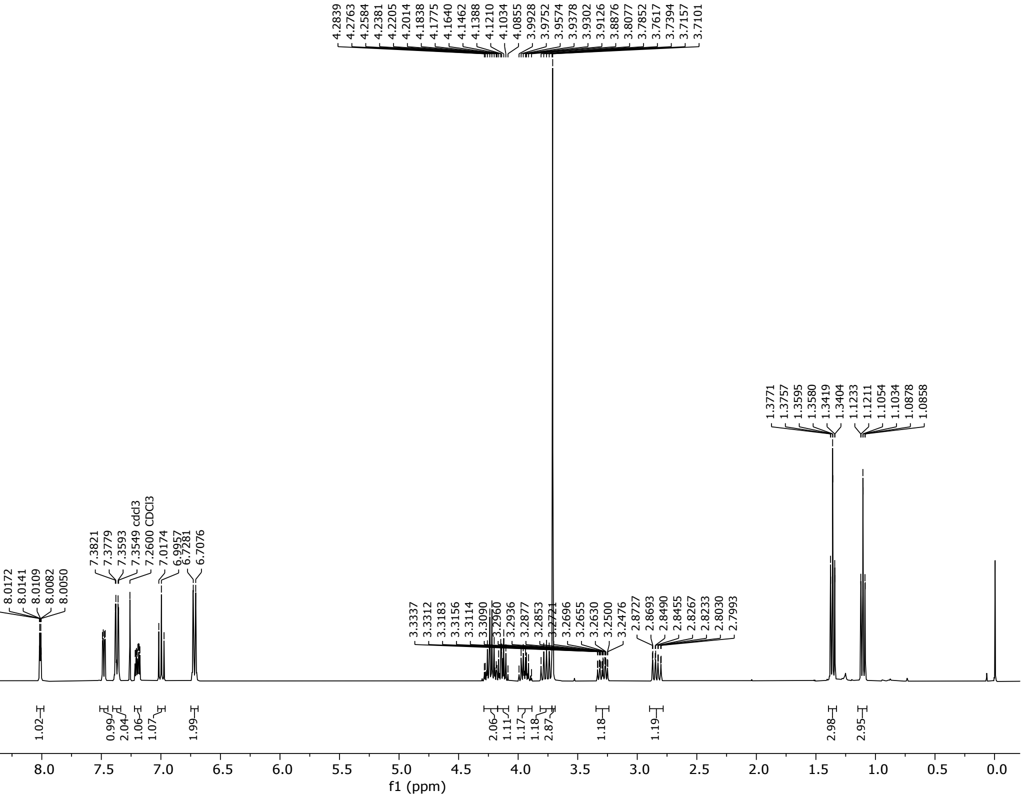

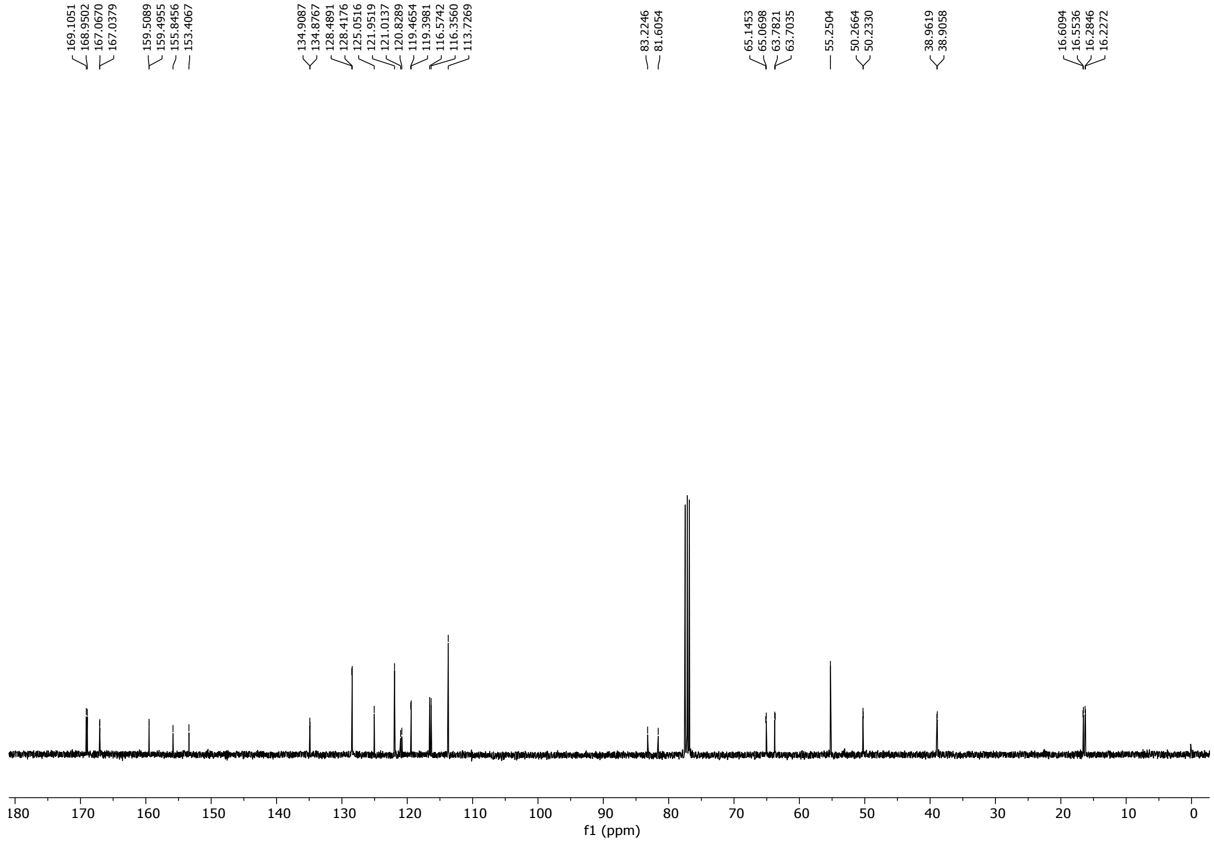

Diethyl [(2*RS*,3*RS*)-2-methyl-3-(phenylcarbamoyl)-3,4-dihydro-2*H*-pyrrol-2-yl]phosphonate, 12m.

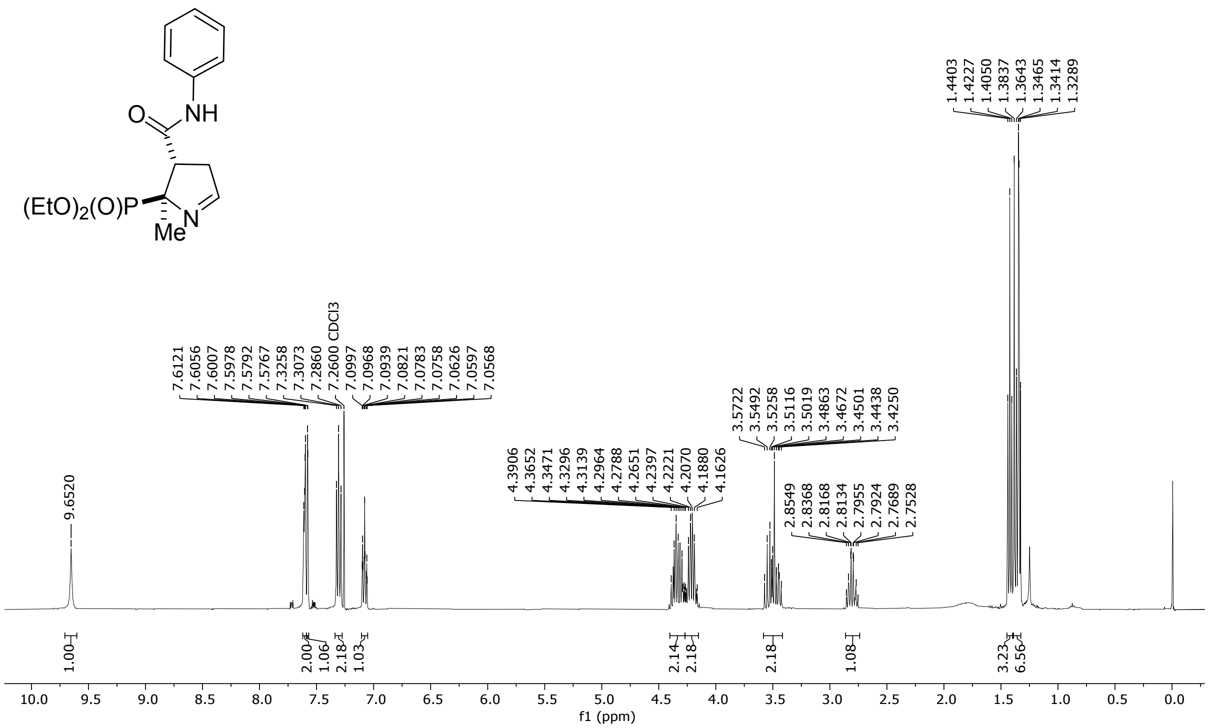

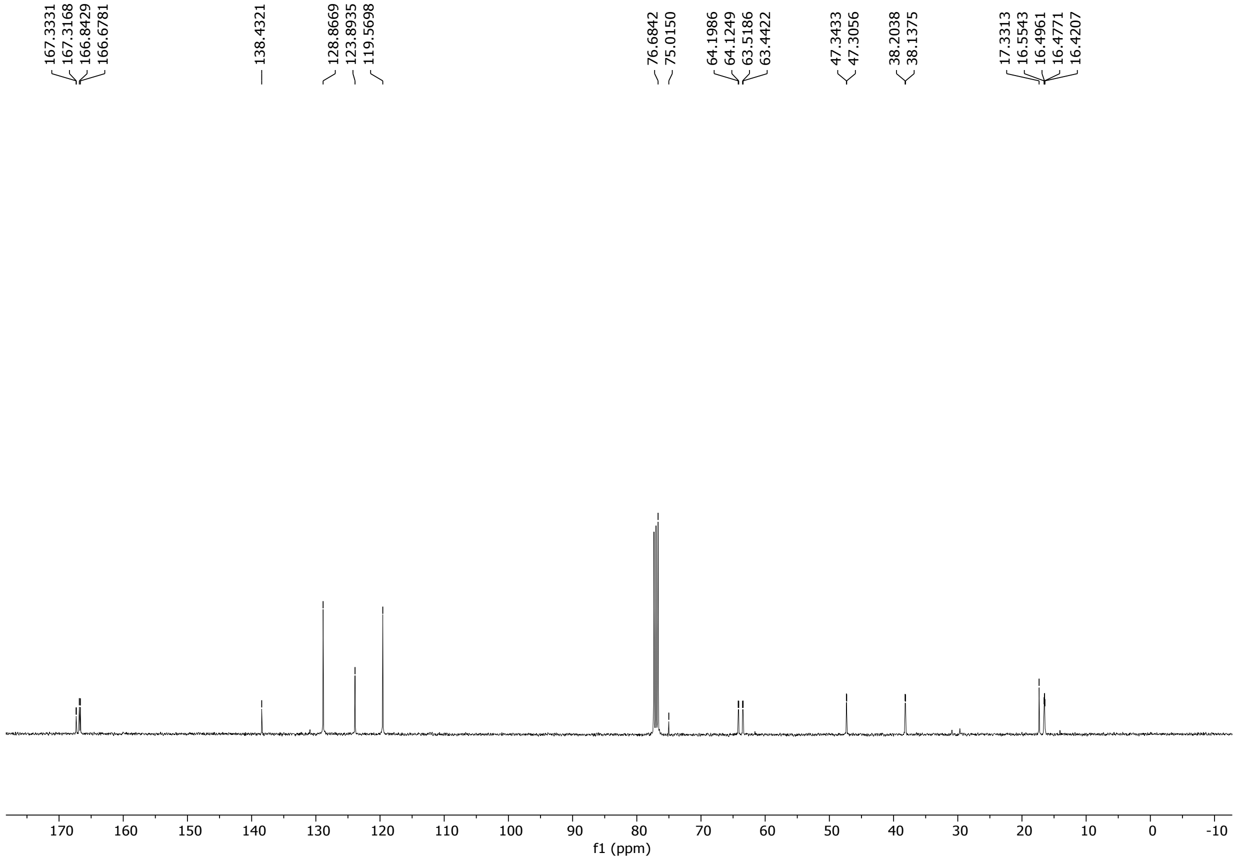

Diethyl [(2RS,3RS)-2-benzyl-3-(phenylcarbamoyl)-3,4-dihydro-2H-pyrrol-2-yl]phosphonate, 12n.

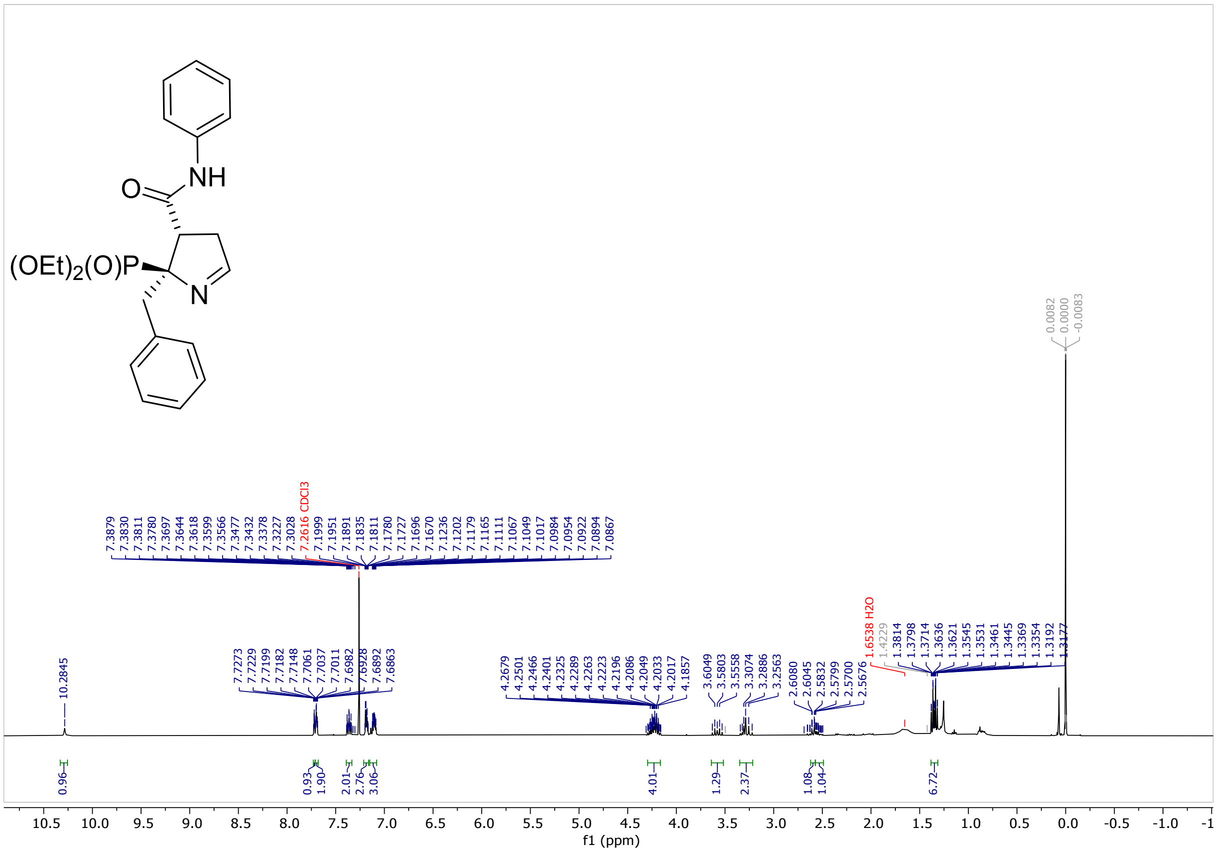

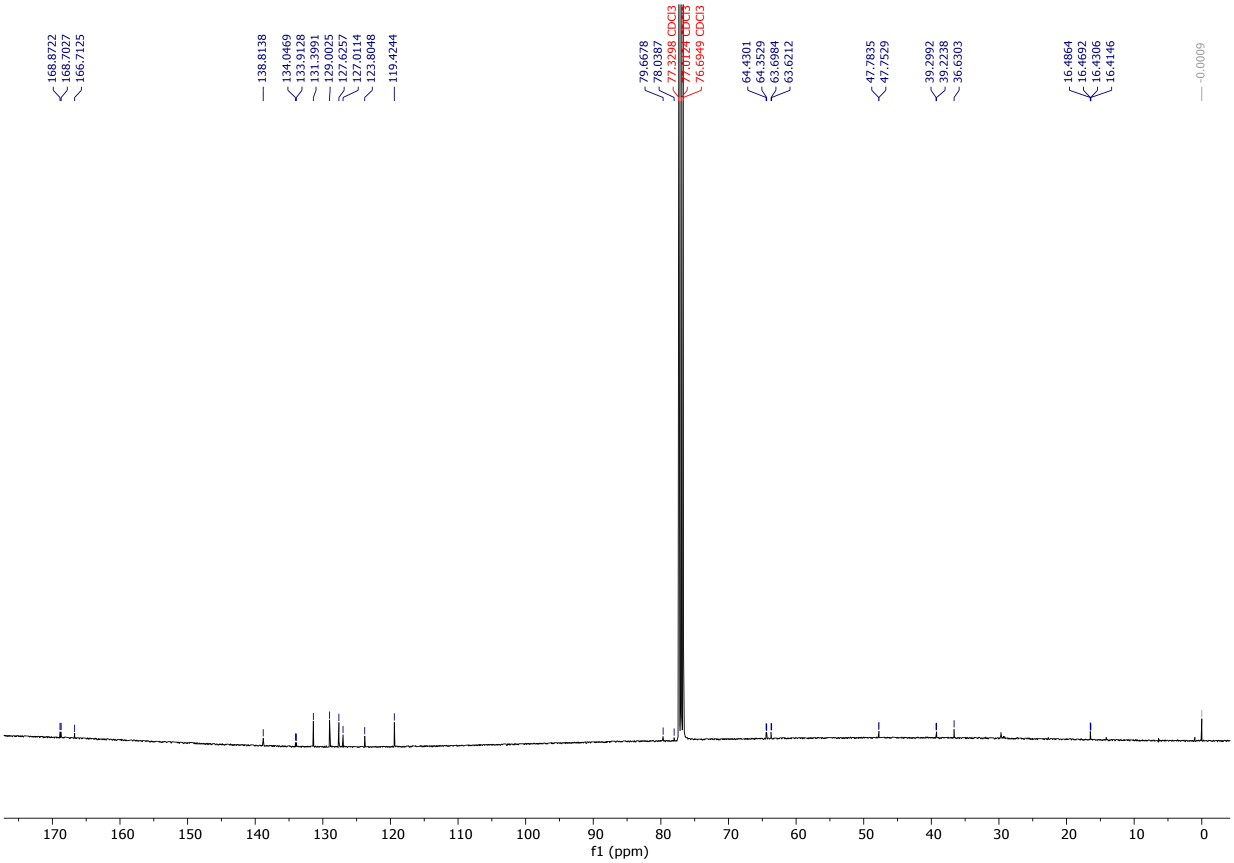

Diethyl [(2*RS*,3*RS*)-2-benzyl-3-(cyclohexylcarbamoyl)-3,4-dihydro-2*H*-pyrrol-2-yl]phosphonate, 12o.

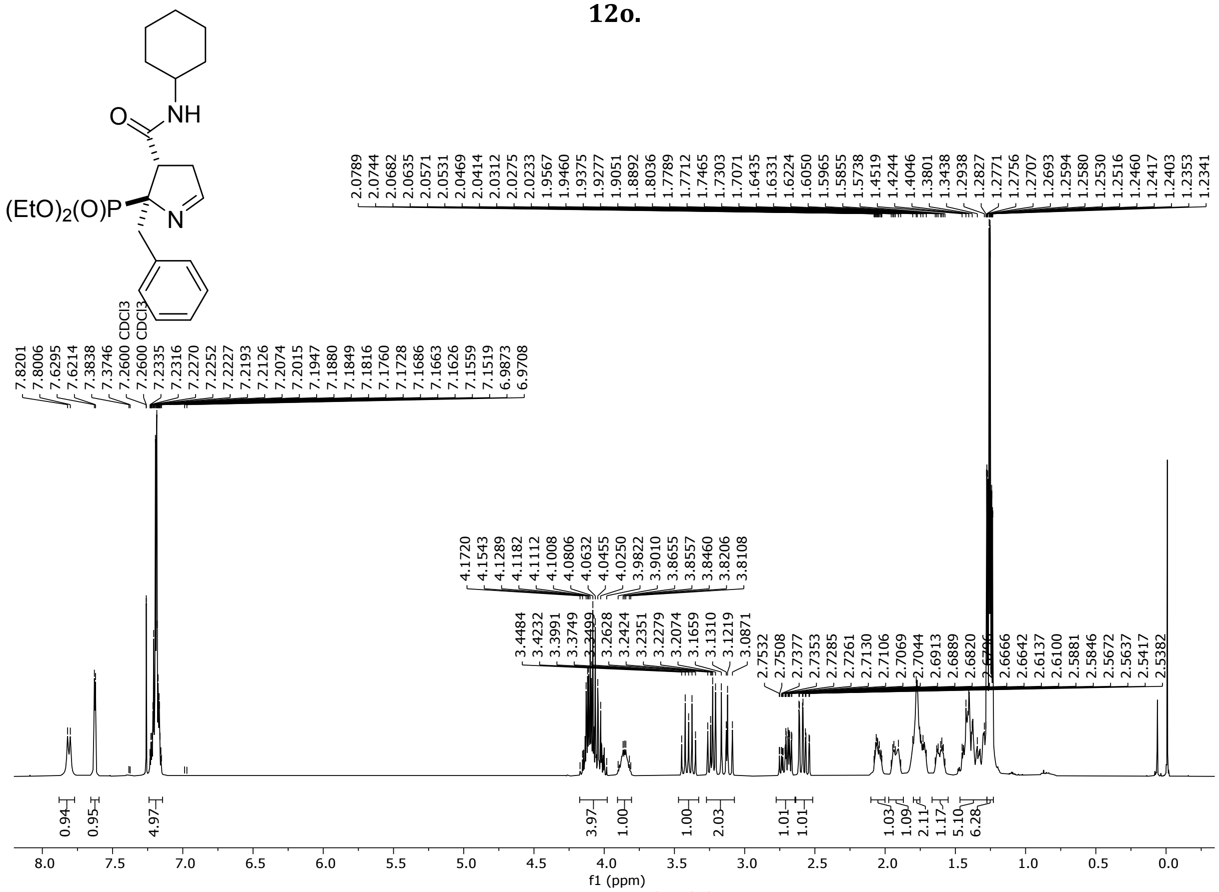

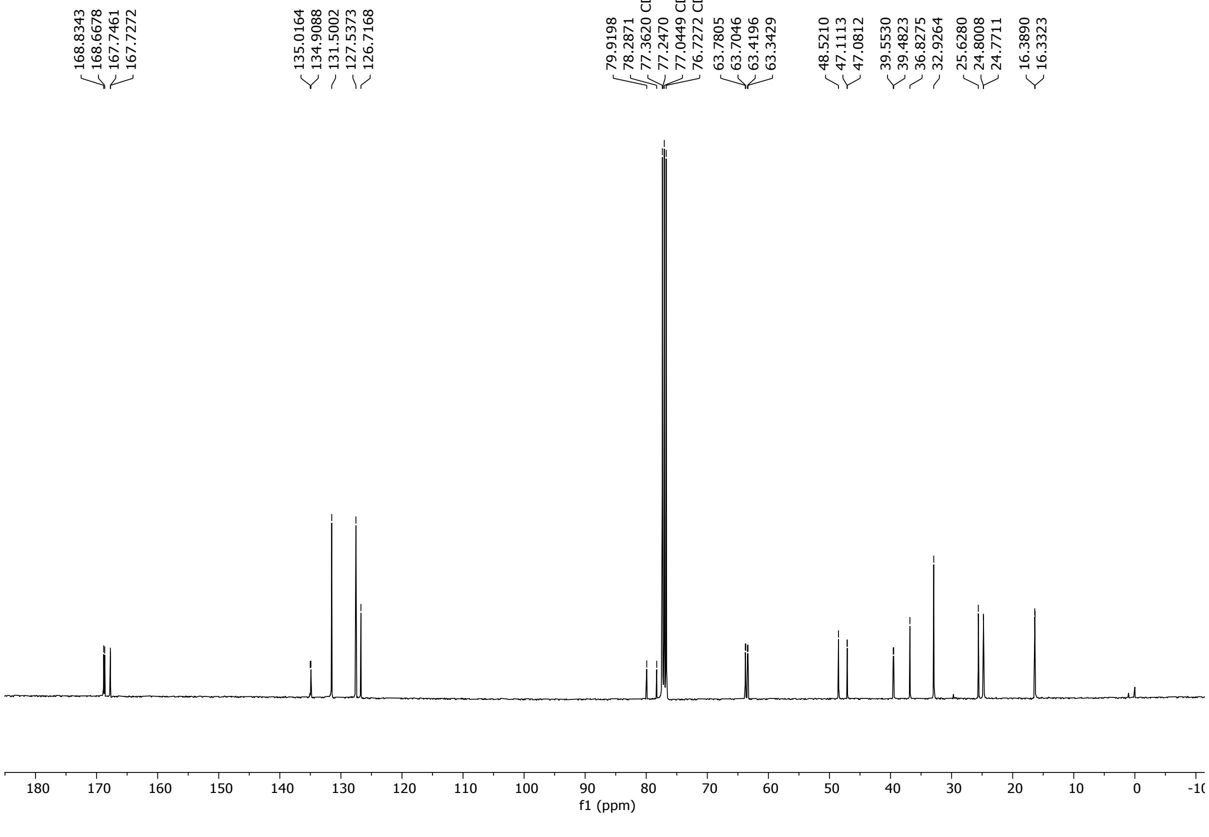

Representative data of ¹H-NMR spectra of new compounds

Table S1. ¹H chemical shifts (ppm) for new compounds including both the multiplicity and the coupling constants (Hz).

Compound	H2	H3	H4	H4	H5
**12a**	4.45-4.53<br>ddt<br>17.4, 8.0, 2.7	3.41-3.54<br>ddt<br>20.9, 10.4, 8.0	2.90<br>ddd<br>18.4, 10.4, 3.5	3.31-3.39<br>m	7.65<br>t<br>2.6
**12b**	4.46-4.35<br>m	3.40-3.48<br>ddt<br>20.9, 10.4, 8.2	2.93<br>dd<br>18.0, 9.5	3.32-3.39<br>m	7.66<br>t<br>2.9
**12c**	-	3.79-3.94<br>c.s.	2.87<br>ddt<br>18.4, 9.5, 1.2	3.32<br>dddd<br>18.4, 8.2, 6.3, 1.0	8.03<br>dt<br>3.8
**12d**	-	3.81-3.93<br>c.s.	2.90<br>ddt<br>18.5, 9.4, 1.6	3.30<br>dddd<br>18.5, 7.5, 6.5, 1.0	8.03<br>dt<br>4.0, 1.2
**12e**	-	3.61<br>quin<br>9.0	2.82-2.90<br>dddd<br>18.5, 9.6, 1.4, 0.7	3.27-3.36<br>dddd<br>18.4, 8.7, 6.0, 0.9	8.04<br>d<br>4.5
**12f**	-	3.80<br>quin<br>9.3	2.85-2.92<br>ddd<br>18.5, 9.6, 0.8	3.31-3.39<br>dddd<br>18.4, 8.8, 6.1, 0.8	8.06<br>dt<br>3.6, 1.3
**12g**	-	3.83<br>quin<br>9.2	2.89<br>ddt<br>18.4, 9.6,1.3	3.28-3.37<br>dddd<br>18.4, 8.6, 6.2, 1.0	8.06<br>dt<br>3.7,1.2
**12h**	-	3.56-3.65<br>ddd<br>18.6, 9.6, 7.1	2.83-2.91<br>ddq<br>18.4, 9.6, 1.0	3.28<br>dtd<br>18.4, 7.0, 1.0	7.98<br>d<br>4.2
**12i**	-	3.75-3.84<br>quin<br>8.4	2.84-2.92<br>ddt<br>18.5, 9.6, 1.2	3.32<br>dt<br>18.4, 6.7	8.03<br>d<br>3.8
**12j**	-	3.83<br>quin<br>9.4	2.83-2.91<br>ddt<br>18.2, 9.6, 1.2	3.25-3.33<br>dddd<br>18.5, 8.5, 6.2, 1.0	8.03<br>dt<br>3.7
**12k**	-	3.73-3.82<br>quin<br>9.2	2.87<br>dd<br>18.3, 9.6	3.35<br>ddd<br>18.0, 8.2, 6.4	8.01<br>d<br>3.6
**12l**	-	3.77<br>quin<br>9.0	2.79-3.87<br>ddd<br>18.4, 9.5, 1.4	3.25-3.33<br>dddd<br>18.4, 8.9, 6.2, 1.0	8.01<br>dt<br>3.6, 1.1
**12m**	-	3.43-3.57<br>c.s.	2.75-2.85<br>m	3.43-3.57<br>c.s.	7.61<br>m
**12n**	-	3.53-3.63<br>quin<br>9.8	2.50-2.57<br>dddd<br>18.6, 10.3, 5.7, 1.0	2.60<br>dd<br>9.9, 1.4	7.72<br>c.s.
**12o**	-	3.44<br>dt<br>19.3, 10.1	3.53-2.62<br>ddd<br>18.6, 10.2, 1.5	3.66-2.77<br>dddd<br>16.1, 9.9, 6.2, 1.0	7.82<br>d<br>7.8

Representative data of ^{13}C-NMR spectra of new compounds

Table S2. ^{13}C chemical shifts (ppm) for new compounds including both the multiplicity and the coupling constants (J_{CP}, Hz).

Compound	C2	C3	C4	C5
**12a**	72.3<br>d<br>162.0	43.3	40.1	169.0<br>d<br>16.5
**12b**	72.3<br>d<br>162.6	43.3	39.6<br>d<br>5.9	169.0<br>d<br>12.5
**12c**	83.9<br>d<br>162.2	50.0<br>d<br>3.0	39.5<br>d<br>5.0	169.2<br>d<br>15.0
**12d**	84.33<br>d<br>160.9	49.7<br>d<br>3.0	39.9<br>d<br>4.1	169.3<br>d<br>14.7
**12e**	83.9<br>d<br>163.2	50.4<br>d<br>2.6	39.3<br>d<br>5.5	169.3<br>d<br>15.3
**12f**	83.8<br>d<br>163.3	50.5<br>d<br>2.7	39.3<br>d<br>5.5	169.4<br>d<br>15.4
**12g**	83.9<br>d<br>162.9	50.4<br>d<br>2.9	39.3<br>d<br>5.3	169.3<br>d<br>15.3
**12h**	84.4<br>d<br>159.8	48.7<br>d<br>2.9	40.8<br>d<br>3.7	169.2<br>d<br>14.2
**12i**	83.5<br>d<br>163.0	50.2<br>d<br>3.3	39.7<br>d<br>5.0	169.6<br>d<br>15.1
**12j**	83.2<br>d<br>163.3	50.0<br>d<br>3.2	39.2<br>d<br>5.2	169.4<br>d<br>15.3
**12k**	83.3<br>d<br>163.2	50.3<br>d<br>3.3	39.2<br>d<br>5.7	169.1<br>d<br>15.6
**12l**	83.2<br>d<br>163.5	50.3<br>d<br>3.4	38.9<br>d<br>5.7	169.1<br>d<br>15.6
**12m**	76.7<br>d<br>168.6	47.3<br>d<br>3.8	38.2<br>d<br>6.7	166.7<br>d<br>16.6
**12n**	79.7<br>d<br>164.5	47.8<br>d<br>3.1	39.3<br>d<br>7.6	168.9<br>d<br>17.1
**12o**	79.9<br>d<br>164.9	47.1<br>d<br>3.0	39.6<br>d<br>7.1	168.9<br>d<br>16.8

Discussion on the spectra data

To unequivocally disclose the relative stereochemistry of the two stereocenters of the final compounds with general structure **12** (Scheme 1 in the manuscript), we prepared monocrystals of compounds **12b**, **12d** and **12h** (Figure 3 in the manuscript). To confirm the relative stereochemistry in the rest of the new compounds we compared their ^{1}H and ^{13}C NMR spectra with the data of compounds **12b**, **12d** and **12h**, with an X-ray crystallographic structure.

The chemical shift, multiplicity, and coupling constants of the different protons and carbons were in concordance with the stereochemistry confirmed in the compounds **12b**, **12d** and **12h** (Tables S1 and S2). Taking a closer look to the ^{1}H NMR in compounds **12a** and **12b**, the H2 is the most deshielded of the aliphatic protons, the H3 and the two protons in position 4 showed complex multiplicities and coupling constants of ~ 18 Hz due to the presence of the phosphorous atom of the phosphonic ester in the 2-position. In the compounds **12a** and **12b** (Figure S1), C2 appears at 72.3 ppm with a multiplicity of doublet and a coupling constant of 162 Hz, and C3 appears at 43.3 ppm, both. Therefore, compound **12a** have the same relative configuration than compound **12b**. For the compound **12m**, with R_1= methyl, C2/C3 have values of 76.7 and 47.3 ppm, respectively. For the rest of the compounds (including **12d** and **12h**), C2 appears at 79.7/84.4 ppm and C3 at 47.0/51.5 ppm. In all the cases, C2 is a doublet due to the interaction with the phosphor atom, showing a coupling constant in the range of 160.0-168.5 Hz, and C3 is a doublet with a coupling constant of 2.5-3.5 Hz. The C4 appears at ~39.0-40.0 ppm as a doublet (^{13}C-P) with a coupling constant of 4.5-7.0 Hz. In the case of C5 at 166.7-169.6 ppm, the doublet has a coupling constant of 15.0-17.0 ppm. These data confirm the relative stereochemistry of the new family with a *cis* relationship between the H3 and the phosphonic ester substituent at the 2-position.

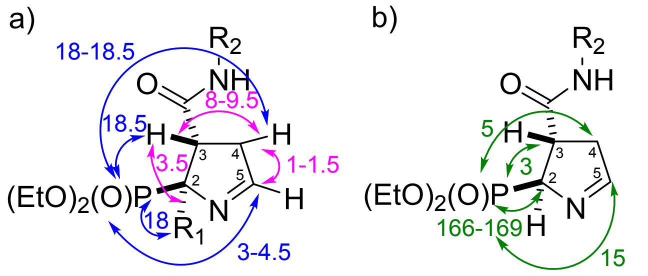

Figure S1. a) Representative coupling constants (Hz) of ^{1}H; in blue ^{1}H-P, in pink, ^{1}H-^{1}H; b) Representative coupling constants (J_{CP} Hz) of ^{13}C-P, in green.

HPLC-MS reports

Diethyl (2*RS*,3*RS*)- 3-[(3-chloro-4-fluorophenylcarbamoyl)-3,4-dihydro-2*H*-pyrrol-2-yl]phosphonate, 12b (PIP04).

```
=====================================================================
Acq. Operator   : SYSTEM                        Seq. Line :    1
Sample Operator : SYSTEM
Acq. Instrument : HPLCMS                         Location :   P1-C-07
Injection Date  : 6/20/2024 8:21:01 AM                Inj :    1
                                               Inj Volume : 5.000 µl
Method          : D:\Chemstation\1\Data\CEM\2024-06-20_CEM_PIP04-VOL2 2024-06-20 08-19-04
                  \Pepito 220-254nm.M (Sequence Method)
Last changed    : 11/28/2023 1:59:46 PM by SYSTEM
Additional Info : Peak(s) manually integrated
```

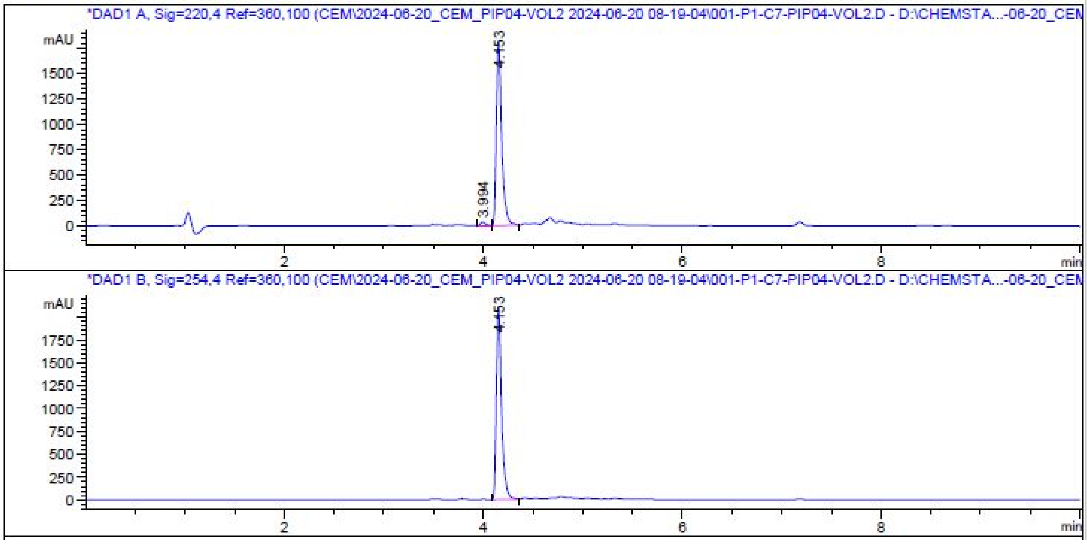


```
=====================================================================
                         Area Percent Report
=====================================================================

Sorted By             :      Signal
Multiplier            :      1.0000
Dilution              :      1.0000
Use Multiplier & Dilution Factor with ISTDs


Signal 1: DAD1 A, Sig=220,4 Ref=360,100
 Signal has been modified after loading from rawdata file!

Peak RetTime Type  Width     Area      Height     Area
  #   [min]        [min]   [mAU*s]     [mAU]        %
----|-------|----|-------|----------|----------|--------|
   1   3.994 BB    0.0545  120.97809   34.86164   1.7572
   2   4.153 BB    0.0572 6763.64014 1826.39600  98.2428

Totals :                  6884.61823 1861.25763



Signal 2: DAD1 B, Sig=254,4 Ref=360,100
 Signal has been modified after loading from rawdata file!

Peak RetTime Type  Width     Area      Height     Area
  #   [min]        [min]   [mAU*s]     [mAU]        %
----|-------|----|-------|----------|----------|--------|
   1   4.153 BB    0.0562 7692.03271 2126.21948 100.0000

Totals :                  7692.03271 2126.21948
```

Diethyl [(2*RS*,3*RS*)-2-phenyl-3-(phenylcarbamoyl)-3,4-dihydro-2*H*-pyrrol-2-yl]phosphonate, 12c (PIP02).

```
=====================================================================
Acq. Operator   : SYSTEM                        Seq. Line :    1
Sample Operator : SYSTEM
Acq. Instrument : HPLCMS                         Location :   P1-A-03
Injection Date  : 6/25/2024 9:35:30 AM                Inj :    1
                                               Inj Volume : 5.000 µl
Different Inj Volume from Sample Entry!  Actual Inj Volume : 10.000 µl
Method          : D:\Chemstation\1\Data\CEM\2024-06-25_CEM_PIP02-003 2024-06-25 09-33-35
                  \Pepito 220-254nm.M (Sequence Method)
Last changed    : 11/28/2023 1:59:46 PM by SYSTEM
Additional Info : Peak(s) manually integrated
```

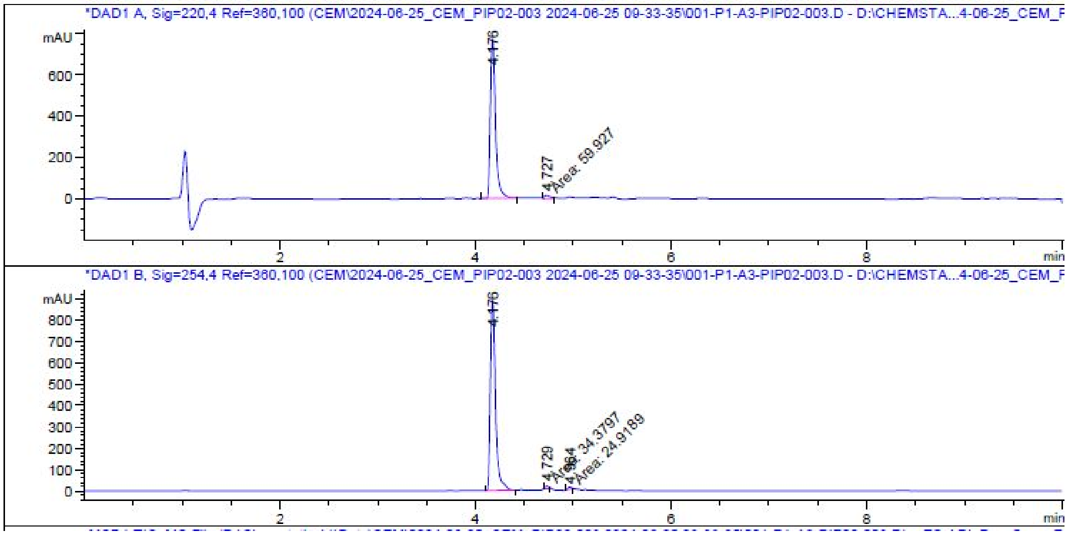


```
=====================================================================
                         Area Percent Report
=====================================================================

Sorted By             :      Signal
Multiplier            :      1.0000
Dilution              :      1.0000
Use Multiplier & Dilution Factor with ISTDs


Signal 1: DAD1 A, Sig=220,4 Ref=360,100
 Signal has been modified after loading from rawdata file!
```

Peak #	RetTime [min]	Type	Width [min]	Area [mAU*s]	Height [mAU]	Area %
1	4.176	BB	0.0534	2737.16846	772.26251	97.8575
2	4.727	MM	0.0683	59.92701	14.62909	2.1425

Totals : 2797.09547 786.89160

```
Signal 2: DAD1 B, Sig=254,4 Ref=360,100
 Signal has been modified after loading from rawdata file!
```

Peak #	RetTime [min]	Type	Width [min]	Area [mAU*s]	Height [mAU]	Area %
1	4.176	BB	0.0537	3188.69409	892.58362	98.1743
2	4.729	MM	0.0406	34.37968	14.10844	1.0585
3	4.964	MM	0.0418	24.91891	9.94543	0.7672

Totals : 3247.99269 916.63749

Diethyl [(2*RS*,3*RS*)-3-((3-chloro-4-fluorophenyl)carbamoyl)-2-phenyl-3,4-dihydro-2*H*-pyrrol-2-yl]phosphonate, 12d (PIP01).

===

Acq. Operator : SYSTEM Seq. Line : 4
Sample Operator : SYSTEM
Acq. Instrument : HPLCMS Location : P1-F-04
Injection Date : 5/15/2024 7:05:36 PM Inj : 1
Inj Volume : 5.000 µl
Method : D:\Chemstation\1\Data\CEM\2024-05-15_CEM_SEQ1 2024-05-15 18-29-03\Pepito 220-254nm.M (Sequence Method)
Last changed : 11/28/2023 1:59:46 PM by SYSTEM
Additional Info : Peak(s) manually integrated

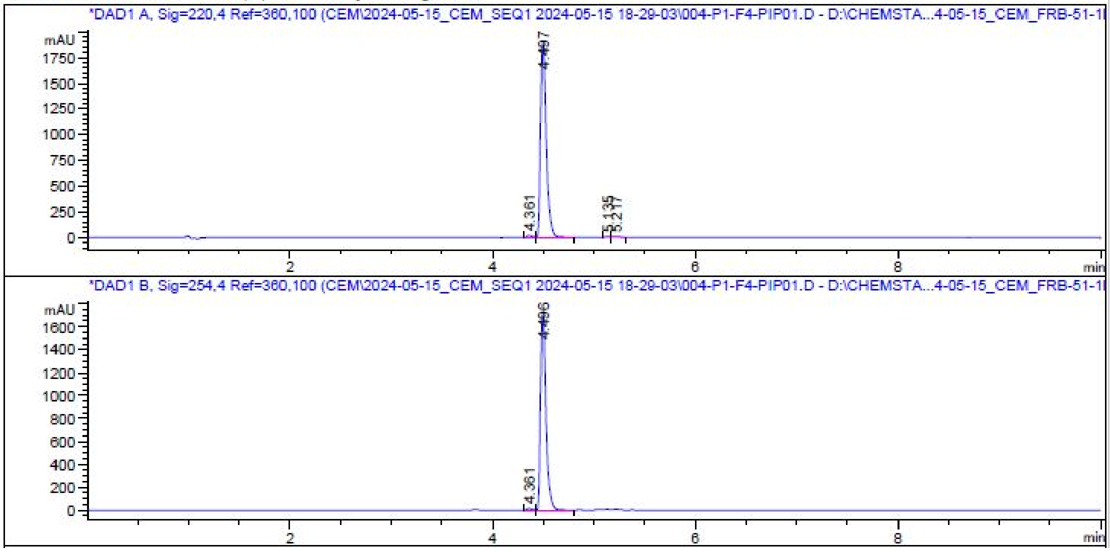

===

Area Percent Report

===

Sorted By : Signal
Multiplier : 1.0000
Dilution : 1.0000
Use Multiplier & Dilution Factor with ISTDs

Signal 1: DAD1 A, Sig=220,4 Ref=360,100
Signal has been modified after loading from rawdata file!

Peak #	RetTime [min]	Type	Width [min]	Area [mAU*s]	Height [mAU]	Area %
1	4.361	BB	0.0496	61.39600	20.13391	0.8619
2	4.497	BB	0.0550	7045.10791	1912.61682	98.9070
3	5.135	BB	0.0407	5.17061	2.10415	0.0726
4	5.217	BB	0.0576	11.29065	3.02082	0.1585

Totals : 7122.96517 1937.87570

Signal 2: DAD1 B, Sig=254,4 Ref=360,100
Signal has been modified after loading from rawdata file!

Peak #	RetTime [min]	Type	Width [min]	Area [mAU*s]	Height [mAU]	Area %
1	4.361	BB	0.0494	43.43524	14.32920	0.7107
2	4.496	BB	0.0527	6068.09717	1738.70654	99.2893

Totals : 6111.53241 1753.03574

Diethyl [(2*RS*,3*RS*)-3-((4-chlorophenyl)carbamoyl)-2-phenyl-3,4-dihydro-2*H*-pyrrol-2-yl]phosphonate, 12e (PIP08).

```
=====================================================================
Acq. Operator   : SYSTEM                        Seq. Line :   7
Sample Operator : SYSTEM
Acq. Instrument : HPLCMS                         Location :   P1-F-07
Injection Date  : 5/15/2024 7:40:23 PM                Inj :   1
                                               Inj Volume : 5.000 µl
Method          : D:\Chemstation\1\Data\CEM\2024-05-15_CEM_SEQ1 2024-05-15 18-29-03\Pepito
                  220-254nm.M (Sequence Method)
Last changed    : 11/28/2023 1:59:46 PM by SYSTEM
Additional Info : Peak(s) manually integrated
```

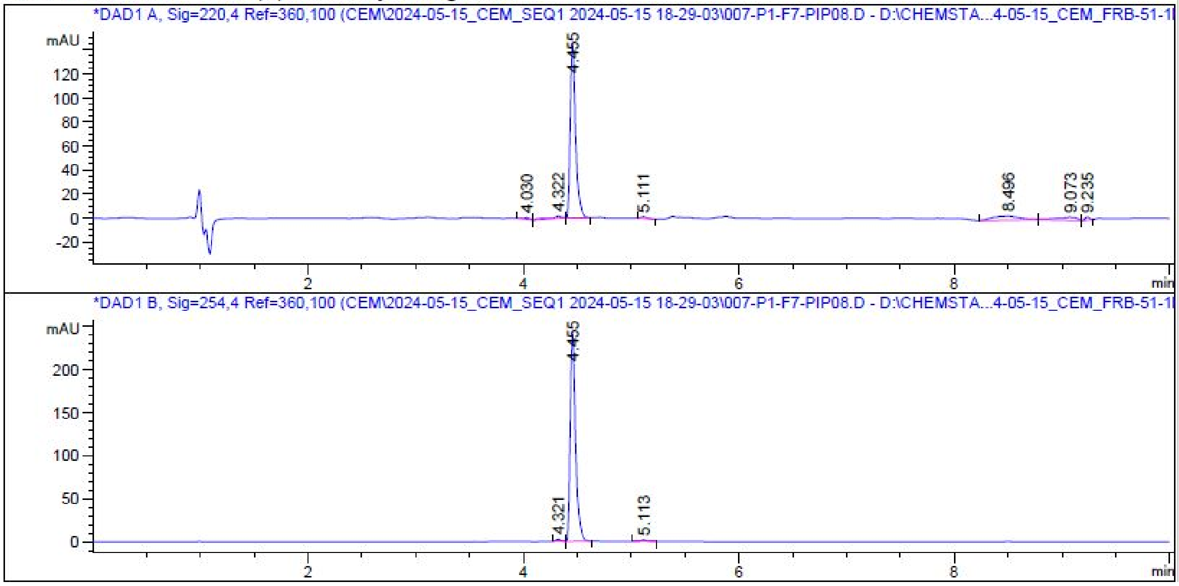

Signal 2: DAD1 B, Sig=254,4 Ref=360,100
Signal has been modified after loading from rawdata file!

Peak #	RetTime [min]	Type	Width [min]	Area [mAU*s]	Height [mAU]	Area %
1	4.321	BB	0.0478	7.98357	2.60166	0.9371
2	4.455	BB	0.0520	836.08142	243.59386	98.1355
3	5.113	BB	0.0567	7.90096	2.15954	0.9274

Totals : 851.96595 248.35505

Diethyl [(2*RS*,3*RS*)-3-((1,1'-biphenyl)-4-ylcarbamoyl)-2-phenyl-3,4-dihydro-2*H*-pyrrol-2-yl]phosphonate, 12f (PIP09).

```
=====================================================================
Acq. Operator   : SYSTEM                        Seq. Line :  10
Sample Operator : SYSTEM
Acq. Instrument : HPLCMS                         Location :   P1-F-10
Injection Date  : 5/15/2024 8:15:09 PM                Inj :   1
                                               Inj Volume : 5.000 µl
Method          : D:\Chemstation\1\Data\CEM\2024-05-15_CEM_SEQ1 2024-05-15 18-29-03\Pepito
                  220-254nm.M (Sequence Method)
Last changed    : 11/28/2023 1:59:46 PM by SYSTEM
Additional Info : Peak(s) manually integrated
```

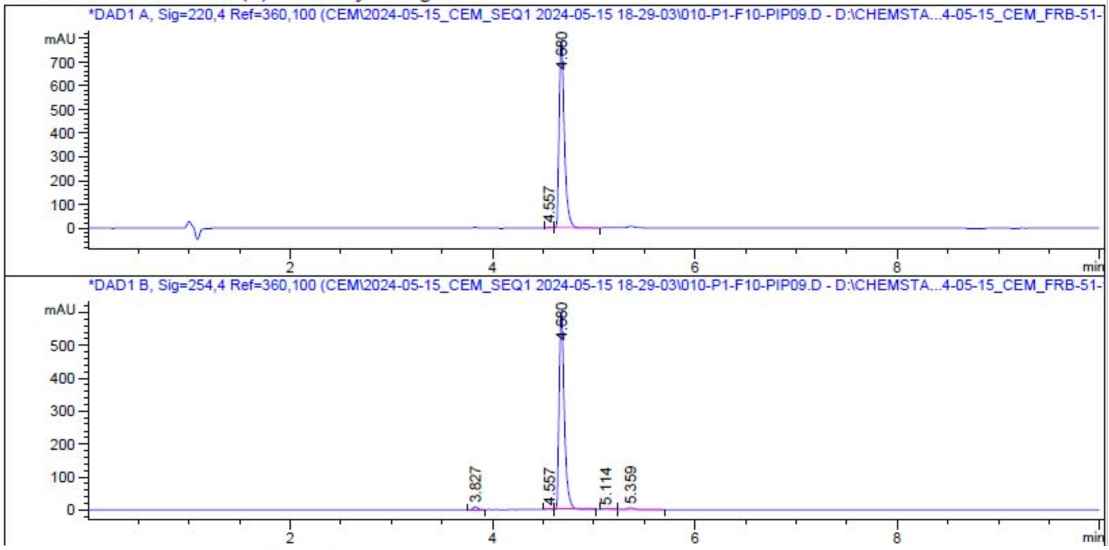


```
=====================================================================
                        Area Percent Report
=====================================================================

Sorted By             :      Signal
Multiplier            :      1.0000
Dilution              :      1.0000
Use Multiplier & Dilution Factor with ISTDs


Signal 1: DAD1 A, Sig=220,4 Ref=360,100
 Signal has been modified after loading from rawdata file!

Peak RetTime Type  Width     Area      Height     Area
  #   [min]        [min]   [mAU*s]     [mAU]       %
----|-------|----|-------|----------|----------|--------|
   1   4.557 BB    0.0466    6.83486    2.44951   0.2432
   2   4.680 BB    0.0534 2803.34180  790.46179  99.7568

Totals :                  2810.17666  792.91131



Signal 2: DAD1 B, Sig=254,4 Ref=360,100
 Signal has been modified after loading from rawdata file!

Peak RetTime Type  Width     Area      Height     Area
  #   [min]        [min]   [mAU*s]     [mAU]       %
----|-------|----|-------|----------|----------|--------|
   1   3.827 BB    0.0498   26.62081    8.22513   1.2205
   2   4.557 BB    0.0481    5.51260    1.88515   0.2527
   3   4.680 BB    0.0529 2119.91016  604.27051  97.1925
   4   5.114 BB    0.0533    5.94016    1.67731   0.2723
   5   5.359 BB    0.0746   23.16307    4.77073   1.0620

Totals :                  2181.14679  620.82883
```

Diethyl [(2*RS*,3*RS*) -3-(cyclohexylcarbamoyl)-2-phenyl-3,4-dihydro-2*H*-pyrrol-2-yl]phosphonate, 12h (PIP03).

```
=====================================================================
Acq. Operator   : SYSTEM                        Seq. Line :   1
Sample Operator : SYSTEM
Acq. Instrument : HPLCMS                         Location :   P1-A-05
Injection Date  : 6/13/2024 10:27:52 AM               Inj :   1
                                               Inj Volume : 5.000 µl
Different Inj Volume from Sample Entry!  Actual Inj Volume : 10.000 µl
Method          : D:\Chemstation\1\Data\CEM\2024-06-13_CEM_PIP03_PIP02 2024-06-13 10-26-12
                  \Pepito 220-254nm.M (Sequence Method)
Last changed    : 11/28/2023 1:59:46 PM by SYSTEM
Additional Info : Peak(s) manually integrated
```

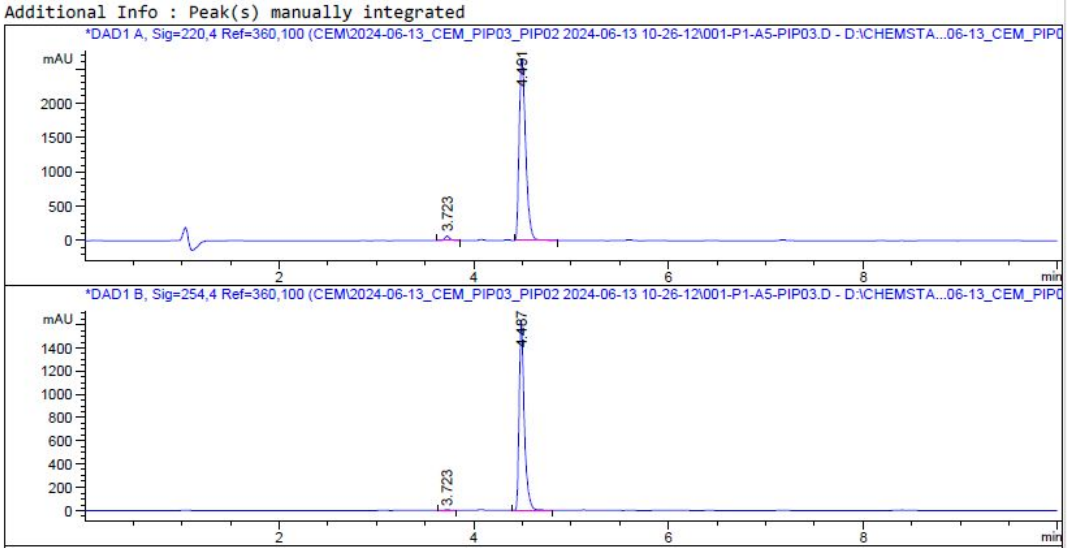

===

Area Percent Report

===

Sorted By : Signal
Multiplier : 1.0000
Dilution : 1.0000
Use Multiplier & Dilution Factor with ISTDs

Signal 1: DAD1 A, Sig=220,4 Ref=360,100
Signal has been modified after loading from rawdata file!

Peak #	RetTime [min]	Type	Width [min]	Area [mAU*s]	Height [mAU]	Area %
1	3.723	BB	0.0574	240.51233	64.71175	1.9084
2	4.491	BB	0.0746	1.23620e4	2643.77100	98.0916

Totals : 1.26025e4 2708.48274

Signal 2: DAD1 B, Sig=254,4 Ref=360,100
Signal has been modified after loading from rawdata file!

Peak #	RetTime [min]	Type	Width [min]	Area [mAU*s]	Height [mAU]	Area %
1	3.723	BB	0.0541	19.92884	5.79551	0.3277
2	4.487	BB	0.0552	6061.64209	1638.53796	99.6723

Totals : 6081.57093 1644.33347

Diethyl [(2*RS*,3*RS*)-2-(4-fluorophenyl)-3-(phenylcarbamoyl)-3,4-dihydro-2*H*-pyrrol-2-yl]phosphonate, 12i (PIP11).

===

Acq. Operator : SYSTEM Seq. Line : 1
Sample Operator : SYSTEM
Acq. Instrument : HPLCMS Location : P1-C-09
Injection Date : 6/27/2024 9:00:50 AM Inj : 1
Inj Volume : 5.000 µl
Method : D:\Chemstation\1\Data\CEM\2024-06-27_CEM_PIP011-VOL2 2024-06-27 08-59-10
\Pepito 220-254nm.M (Sequence Method)
Last changed : 11/28/2023 1:59:46 PM by SYSTEM
Additional Info : Peak(s) manually integrated

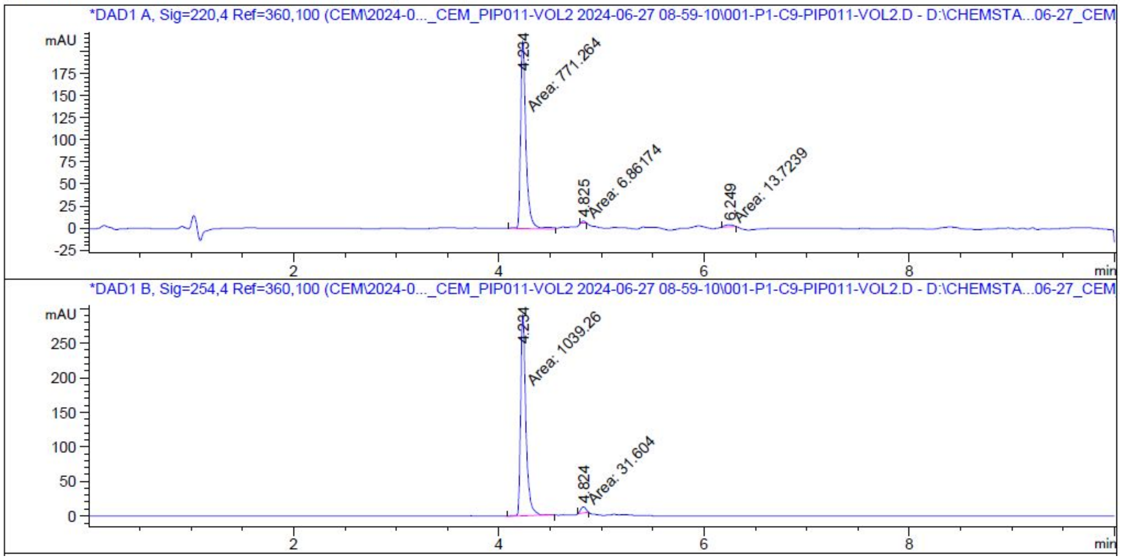

===

Area Percent Report

===

Sorted By : Signal
Multiplier : 1.0000
Dilution : 1.0000
Use Multiplier & Dilution Factor with ISTDs

Signal 1: DAD1 A, Sig=220,4 Ref=360,100
Signal has been modified after loading from rawdata file!

Peak #	RetTime [min]	Type	Width [min]	Area [mAU*s]	Height [mAU]	Area %
1	4.234	MM	0.0602	771.26398	213.44968	97.4003
2	4.825	MM	0.0407	6.86174	2.80827	0.8665
3	6.249	MM	0.0979	13.72390	2.33734	1.7331

Totals : 791.84961 218.59528

Signal 2: DAD1 B, Sig=254,4 Ref=360,100
Signal has been modified after loading from rawdata file!

Peak #	RetTime [min]	Type	Width [min]	Area [mAU*s]	Height [mAU]	Area %
1	4.234	MM	0.0592	1039.25671	292.83072	97.0487
2	4.824	MM	0.0595	31.60399	8.85122	2.9513

Totals : 1070.86071 301.68194

Diethyl [(2*RS*,3*RS*)-2-(4-methoxyphenyl)-3-(phenylcarbamoyl)-3,4-dihydro-2*H*-pyrrol-2-yl]phosphonate, 12k (PIP12).

```
=====================================================================
Acq. Operator   : SYSTEM                        Seq. Line :   5
Sample Operator : SYSTEM
Acq. Instrument : HPLCMS                         Location :   P1-F-05
Injection Date  : 5/15/2024 7:17:12 PM                Inj :   1
                                               Inj Volume : 5.000 µl
Method          : D:\Chemstation\1\Data\CEM\2024-05-15_CEM_SEQ1 2024-05-15 18-29-03\Pepito
                  220-254nm.M (Sequence Method)
Last changed    : 11/28/2023 1:59:46 PM by SYSTEM
Additional Info : Peak(s) manually integrated
```

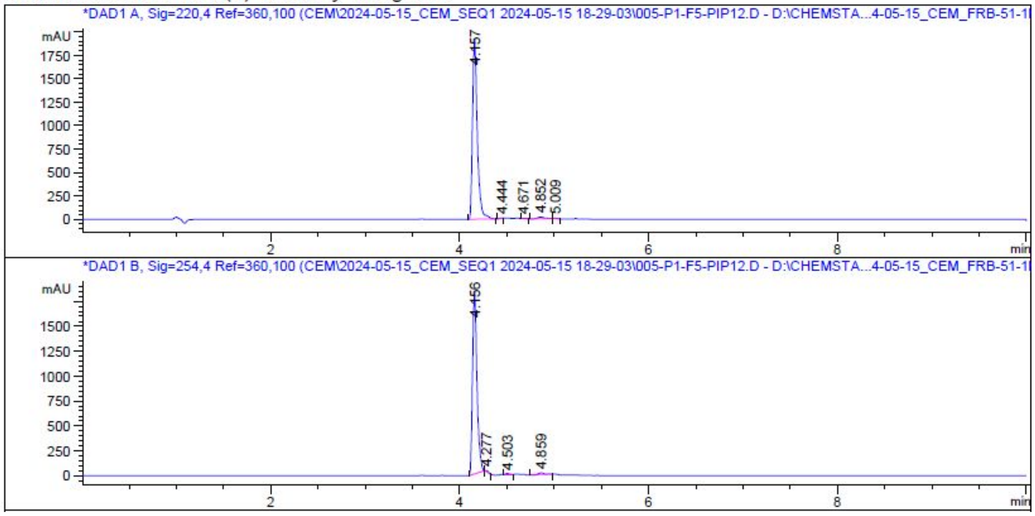


```
=====================================================================
                         Area Percent Report
=====================================================================

Sorted By             :      Signal
Multiplier            :      1.0000
Dilution              :      1.0000
Use Multiplier & Dilution Factor with ISTDs
```

Signal 1: DAD1 A, Sig=220,4 Ref=360,100
Signal has been modified after loading from rawdata file!

Peak #	RetTime [min]	Type	Width [min]	Area [mAU*s]	Height [mAU]	Area %
1	4.157	BB	0.0531	6822.16895	1934.46265	98.6817
2	4.444	BB	0.0357	8.85336	4.01289	0.1281
3	4.671	BB	0.0487	5.28859	1.67976	0.0765
4	4.852	BB	0.0608	68.75660	17.12285	0.9946
5	5.009	BB	0.0500	8.23708	2.66756	0.1191

Totals : 6913.30457 1959.94571

Signal 2: DAD1 B, Sig=254,4 Ref=360,100
Signal has been modified after loading from rawdata file!

Peak #	RetTime [min]	Type	Width [min]	Area [mAU*s]	Height [mAU]	Area %
1	4.156	BB	0.0502	6035.15820	1842.42444	97.9551
2	4.277	BB	0.0400	19.82941	7.70707	0.3218
3	4.503	BB	0.0495	30.67878	9.54586	0.4979
4	4.859	BB	0.0644	75.48027	17.46374	1.2251

Totals : 6161.14666 1877.14111

Diethyl [(2RS,3RS)-2-benzyl-3-(phenylcarbamoyl)-3,4-dihydro-2H-pyrrol-2-yl]phosphonate, 12n (PIP17).

```
Data File D:\Chemsta...9_SVC_ALR-PIP17-F1_F2 2024-10-29 14-06-19\001-P1-F10-ALR-PIP17-F1.D
Sample Name:

    =====================================================================
    Acq. Operator   : SYSTEM                        Seq. Line :   1
    Sample Operator : SYSTEM
    Acq. Instrument : HPLCMS                         Location :   P1-F-10
    Injection Date  : 10/29/2024 2:08:07 PM               Inj :   1
                                                   Inj Volume : 5.000 µl
    Method          : D:\Chemstation\1\Data\SVC\2024-10-29_SVC_ALR-PIP17-F1_F2 2024-10-29 14-06-
                      19\Pepito 220-254nm.M (Sequence Method)
    Last changed    : 7/15/2024 2:25:35 PM by SYSTEM
    Additional Info : Peak(s) manually integrated
```

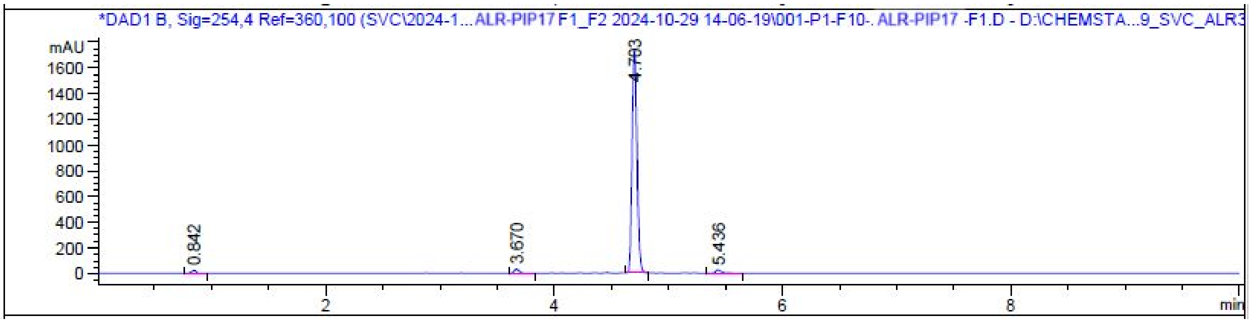

Signal 2: DAD1 B, Sig=254,4 Ref=360,100
Signal has been modified after loading from rawdata file!

Peak #	RetTime [min]	Type	Width [min]	Area [mAU*s]	Height [mAU]	Area %
1	0.842	BB	0.0460	67.14371	23.02960	1.2370
2	3.670	BB	0.0464	93.36327	31.68293	1.7201
3	4.703	BB	0.0464	5164.68457	1753.65051	95.1506
4	5.436	BB	0.0580	102.71493	26.04151	1.8923

Totals : 5427.90648 1834.40455

Diethyl [(2*RS*,3*RS*)-2-benzyl-3-(cyclohexylcarbamoyl)-3,4-dihydro-2*H*-pyrrol-2-yl]phosphonate, 12o (PIP16).

===

Acq. Operator : SYSTEM Seq. Line : 3
Sample Operator : SYSTEM
Acq. Instrument : HPLCMS Location : P1-F-02
Injection Date : 7/1/2024 10:41:45 AM Inj : 1
Inj Volume : 5.000 µl
Method : D:\Chemstation\1\Data\CEM\2024-07-01_CEM_PIP015 TOP_PIP015 BOTTOM_PIP016 2024-07-01 10-15-35\Pepito 220-254nm.M (Sequence Method)
Last changed : 11/28/2023 1:59:46 PM by SYSTEM
Additional Info : Peak(s) manually integrated

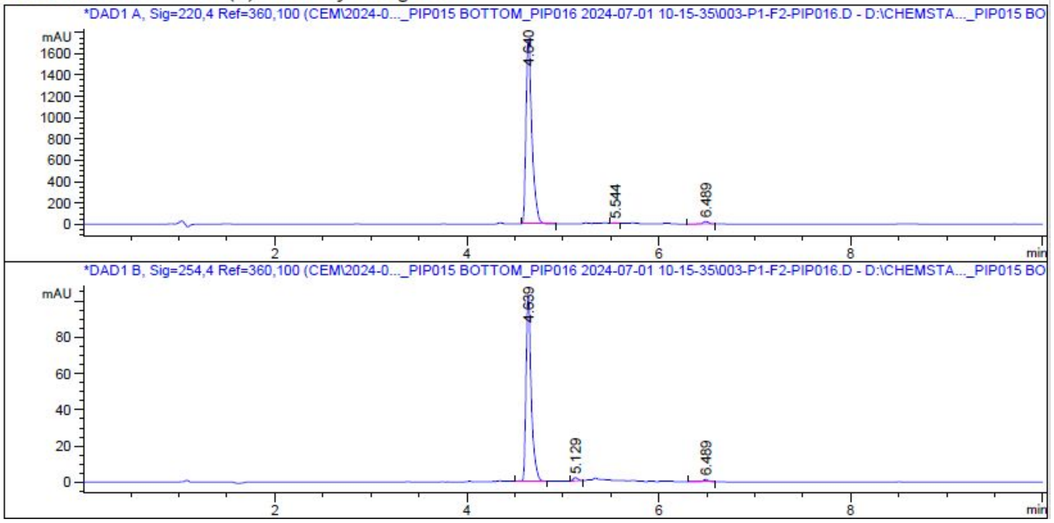

===

Area Percent Report

===

Sorted By : Signal
Multiplier : 1.0000
Dilution : 1.0000
Use Multiplier & Dilution Factor with ISTDs

Signal 1: DAD1 A, Sig=220,4 Ref=360,100
Signal has been modified after loading from rawdata file!

Peak #	RetTime [min]	Type	Width [min]	Area [mAU*s]	Height [mAU]	Area %
1	4.640	BB	0.0635	7394.00342	1744.16626	98.4126
2	5.544	BB	0.0486	9.83796	3.31660	0.1309
3	6.489	BB	0.0652	109.42482	24.94288	1.4564
Totals :				7513.26620	1772.42574	

Signal 2: DAD1 B, Sig=254,4 Ref=360,100
Signal has been modified after loading from rawdata file!

Peak #	RetTime [min]	Type	Width [min]	Area [mAU*s]	Height [mAU]	Area %
1	4.639	BB	0.0580	388.26193	102.95723	97.0611
2	5.129	BB	0.0539	6.42494	1.87720	1.6062
3	6.489	BB	0.0600	5.33130	1.35230	1.3328
Totals :				400.01817	106.18672	

X-ray crystallographic data for 12b, 12d and 12h

(2*RS*,3*RS*)-3-[(3-chloro-4-fluorophenylcarbamoyl)-3,4-dihydro-2*H*-pyrrol-2-yl]phosphonate, 12b.

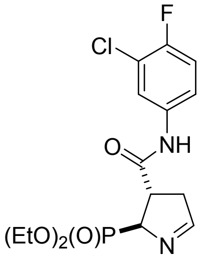

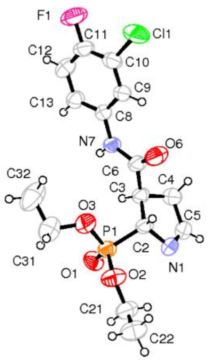

Table S3. Crystal data and structure refinement for **12b**.

Identification code	**12b**	
Empirical formula	C15 H19 Cl F N2 O4 P	
Formula weight	376.74	
Temperature	293(2) K	
Wavelength	0.71073 Å	
Crystal system	Triclinic	
Space group	P -1	
Unit cell dimensions	a = 8.893(3) Å	α= 99.21(4)°.
	b = 10.725(5) Å	β= 111.60(3)°.
	c = 10.981(4) Å	γ = 106.55(3)°.
Volume	891.3(7) Å^3	
Z	2	
Density (calculated)	1.404 Mg/m^3	
Absorption coefficient	0.335 mm^{-1}	
F(000)	392	
Crystal size	0.39 x 0.24 x 0.18 mm^3	
Theta range for data collection	2.075 to 24.965°.	
Index ranges	-10<=h<=9, -12<=k<=12, 0<=l<=13	
Reflections collected	3313	
Independent reflections	3135 [R(int) = 0.0209]	
Completeness to theta = 25.242°	97.4 %	
Refinement method	Full-matrix least-squares on F^2	
Data / restraints / parameters	3135 / 0 / 219	
Goodness-of-fit on F^2	1.039	
Final R indices [I>2sigma(I)]	R1 = 0.0576, wR2 = 0.1362	
R indices (all data)	R1 = 0.1055, wR2 = 0.1563	
Largest diff. peak and hole	0.291 and -0.214 e.Å^{-3}	

Table S4. Atomic coordinates (x 10^4) and equivalent isotropic displacement parameters ($Å^2$x 10^3)

For **12b**. U(eq) is defined as one third of the trace of the orthogonalized U^{ij} tensor.

	x	y	z	U(eq)
Cl(1)	-4473(2)	-1295(1)	-5853(1)	99(1)
P(1)	3276(1)	6647(1)	-53(1)	54(1)
F(1)	-2673(4)	-2820(2)	-4366(3)	98(1)
O(1)	5157(3)	7030(3)	633(3)	65(1)
O(2)	2608(3)	7734(3)	451(3)	72(1)
O(3)	2141(4)	5326(3)	104(3)	77(1)
O(6)	-323(4)	3597(3)	-3873(3)	95(1)
N(1)	3090(5)	7391(3)	-2304(4)	69(1)
N(7)	1505(4)	2614(3)	-2779(3)	52(1)
C(2)	2357(5)	6209(3)	-1889(4)	54(1)
C(3)	2684(5)	5012(3)	-2573(4)	55(1)
C(4)	3074(6)	5503(4)	-3717(4)	70(1)
C(5)	3449(6)	6982(4)	-3258(5)	71(1)
C(6)	1130(5)	3680(4)	-3138(4)	61(1)
C(8)	359(4)	1238(3)	-3208(3)	48(1)
C(9)	-1346(5)	740(4)	-4204(4)	55(1)
C(10)	-2348(5)	-630(4)	-4588(4)	59(1)
C(11)	-1654(6)	-1480(4)	-3982(4)	64(1)
C(12)	22(6)	-1004(4)	-3004(5)	71(1)
C(13)	1035(5)	357(4)	-2617(4)	59(1)
C(21)	3487(5)	9175(4)	757(5)	73(1)
C(22)	2440(6)	9889(4)	1060(5)	90(2)
C(31)	2453(6)	5097(5)	1450(5)	89(2)
C(32)	2093(7)	3633(6)	1243(7)	122(2)

Table S5. Bond lengths [Å] and angles [°] for **12b**.

Cl(1)-C(10)	1.730(4)
P(1)-O(1)	1.455(3)
P(1)-O(2)	1.565(3)
P(1)-O(3)	1.565(3)
P(1)-C(2)	1.793(4)
F(1)-C(11)	1.359(4)
O(2)-C(21)	1.437(4)
O(3)-C(31)	1.475(5)
O(6)-C(6)	1.213(4)
N(1)-C(5)	1.253(5)
N(1)-C(2)	1.473(5)
N(7)-C(6)	1.356(4)
N(7)-C(8)	1.416(4)
C(2)-C(3)	1.538(5)
C(3)-C(6)	1.515(5)
C(3)-C(4)	1.549(6)
C(4)-C(5)	1.484(6)
C(8)-C(13)	1.382(5)
C(8)-C(9)	1.382(5)
C(9)-C(10)	1.381(5)
C(10)-C(11)	1.366(6)
C(11)-C(12)	1.359(6)
C(12)-C(13)	1.376(5)
C(21)-C(22)	1.455(6)
C(31)-C(32)	1.471(7)
O(1)-P(1)-O(2)	114.33(16)
O(1)-P(1)-O(3)	116.42(17)
O(2)-P(1)-O(3)	102.37(17)
O(1)-P(1)-C(2)	114.98(18)
O(2)-P(1)-C(2)	107.50(17)
O(3)-P(1)-C(2)	99.51(17)
C(21)-O(2)-P(1)	123.4(3)

C(31)-O(3)-P(1)	122.5(3)
C(5)-N(1)-C(2)	108.4(3)
C(6)-N(7)-C(8)	128.0(3)
N(1)-C(2)-C(3)	107.3(3)
N(1)-C(2)-P(1)	109.8(2)
C(3)-C(2)-P(1)	114.4(2)
C(6)-C(3)-C(2)	112.6(3)
C(6)-C(3)-C(4)	112.5(3)
C(2)-C(3)-C(4)	101.8(3)
C(5)-C(4)-C(3)	102.0(3)
N(1)-C(5)-C(4)	117.2(4)
O(6)-C(6)-N(7)	123.8(3)
O(6)-C(6)-C(3)	121.3(3)
N(7)-C(6)-C(3)	114.9(3)
C(13)-C(8)-C(9)	119.4(3)
C(13)-C(8)-N(7)	116.8(3)
C(9)-C(8)-N(7)	123.7(3)
C(10)-C(9)-C(8)	119.5(4)
C(11)-C(10)-C(9)	120.0(4)
C(11)-C(10)-Cl(1)	119.2(3)
C(9)-C(10)-Cl(1)	120.8(3)
F(1)-C(11)-C(12)	120.0(4)
F(1)-C(11)-C(10)	118.9(4)
C(12)-C(11)-C(10)	121.1(4)
C(11)-C(12)-C(13)	119.5(4)
C(12)-C(13)-C(8)	120.5(4)
O(2)-C(21)-C(22)	109.6(4)
C(32)-C(31)-O(3)	106.7(4)

Table S6. Anisotropic displacement parameters ($Å^2 x 10^3$) for **12b**. The anisotropic displacement factor exponent takes the form: $-2\pi^2[h^2a^{*2}U^{11} + ... + 2 h k a^* b^* U^{12}]$.

	U^{11}	U^{22}	U^{33}	U^{23}	U^{13}	U^{12}
Cl(1)	76(1)	72(1)	86(1)	-4(1)	1(1)	-1(1)
P(1)	51(1)	45(1)	58(1)	11(1)	16(1)	20(1)
F(1)	119(2)	42(1)	116(2)	14(1)	55(2)	10(1)
O(1)	50(2)	74(2)	57(2)	10(1)	8(1)	27(1)
O(2)	63(2)	50(2)	98(2)	10(2)	34(2)	21(1)
O(3)	81(2)	59(2)	77(2)	23(2)	26(2)	15(2)
O(6)	57(2)	51(2)	117(3)	22(2)	-20(2)	15(1)
N(1)	89(3)	50(2)	63(2)	22(2)	20(2)	31(2)
N(7)	45(2)	41(2)	54(2)	9(1)	6(1)	16(1)
C(2)	50(2)	39(2)	60(2)	11(2)	10(2)	19(2)
C(3)	50(2)	41(2)	59(2)	11(2)	9(2)	20(2)
C(4)	74(3)	56(2)	64(3)	11(2)	18(2)	22(2)
C(5)	81(3)	56(3)	59(3)	24(2)	17(2)	16(2)
C(6)	57(2)	43(2)	62(3)	11(2)	5(2)	19(2)
C(8)	52(2)	42(2)	48(2)	12(2)	19(2)	19(2)
C(9)	58(2)	45(2)	49(2)	8(2)	14(2)	17(2)
C(10)	62(2)	51(2)	50(2)	2(2)	23(2)	12(2)
C(11)	86(3)	37(2)	69(3)	7(2)	45(2)	14(2)
C(12)	83(3)	54(3)	90(3)	30(2)	41(3)	36(2)
C(13)	57(2)	52(2)	64(2)	19(2)	20(2)	24(2)
C(21)	71(3)	48(2)	88(3)	15(2)	26(2)	20(2)
C(22)	103(4)	65(3)	110(4)	15(3)	52(3)	43(3)
C(31)	84(3)	90(4)	105(4)	48(3)	45(3)	34(3)
C(32)	86(4)	102(4)	166(6)	77(4)	38(4)	20(3)

Table S7. Hydrogen bonds for **12b** [Å and °].

D-H...A	d(D-H)	d(H...A)	d(D...A)	<(DHA)
N(7)-H(7)...O(1)#1	0.86	2.06	2.903(4)	166.0
C(9)-H(9)...O(6)	0.93	2.26	2.857(5)	121.5
C(13)-H(13)...O(1)#1	0.93	2.59	3.358(5)	139.7
C(32)-H(32B)...F(1)#2	0.96	2.60	3.563(7)	176.8

Symmetry transformations used to generate equivalent atoms:

#1 -x+1,-y+1,-z   #2 -x,-y,-z

Diethyl [(2*RS*,3*RS*)-3-((3-chloro-4-fluorophenyl)carbamoyl)-2-phenyl-3,4-dihydro-2*H*-pyrrol-2-yl]phosphonate, 12d.

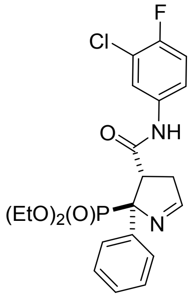

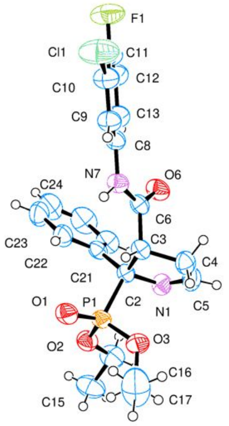

Table S8. Crystal data and structure refinement for **12d**.

Identification code	**12d**	
Empirical formula	C21 H23 Cl F N2 O4 P	
Formula weight	452.83	
Temperature	294(2) K	
Wavelength	0.71073 Å	
Crystal system	Triclinic	
Space group	P -1	
Unit cell dimensions	a = 7.500(3) Å	α= 71.707(7)°.
	b = 12.451(5) Å	β= 74.367(7)°.
	c = 12.973(5) Å	γ = 82.293(8)°.
Volume	1106.0(8) Å^3	
Z	2	
Density (calculated)	1.360 Mg/m^3	
Absorption coefficient	0.283 mm^{-1}	
F(000)	472	
Crystal size	0.270 x 0.180 x 0.140 mm^3	
Theta range for data collection	1.704 to 28.287°.	
Index ranges	-9<=h<=9, -16<=k<=16, -17<=l<=17	
Reflections collected	32053	
Independent reflections	5468 [R(int) = 0.0229]	
Completeness to theta = 25.242°	100.0 %	
Absorption correction	Semi-empirical from equivalents	
Max. and min. transmission	1 and 0.9	
Refinement method	Full-matrix least-squares on F^2	
Data / restraints / parameters	5468 / 0 / 273	
Goodness-of-fit on F^2	1.035	
Final R indices [I>2sigma(I)]	R1 = 0.0476, wR2 = 0.1341	
R indices (all data)	R1 = 0.0561, wR2 = 0.1427	
Largest diff. peak and hole	0.753 and -0.327 e.Å^{-3}	

Table S9. Atomic coordinates (x 10^4) and equivalent isotropic displacement parameters (Å^{2}x 10^3) For **12d**. U(eq) is defined as one third of the trace of the orthogonalized U^{ij} tensor.

	x	y	z	U(eq)
Cl(1)	7683(1)	9275(1)	167(1)	100(1)
P(1)	8488(1)	4280(1)	7427(1)	42(1)
F(1)	6517(3)	11236(1)	997(1)	93(1)
O(1)	10165(2)	4284(1)	6526(1)	56(1)
O(2)	9073(2)	4160(1)	8530(1)	55(1)
O(3)	7143(2)	3320(1)	7676(1)	60(1)
O(6)	4904(2)	7546(1)	5785(1)	58(1)
N(1)	5202(2)	5335(1)	8026(1)	49(1)
N(7)	7075(2)	7216(1)	4300(1)	45(1)
C(2)	6931(2)	5551(1)	7125(1)	38(1)
C(3)	6357(2)	5676(1)	6008(1)	41(1)
C(4)	4478(3)	5120(2)	6442(2)	55(1)
C(5)	3968(3)	5109(2)	7641(2)	57(1)
C(6)	6047(2)	6913(1)	5367(1)	42(1)
C(8)	6914(2)	8270(1)	3485(2)	45(1)
C(9)	7316(3)	8270(2)	2380(2)	51(1)
C(10)	7199(3)	9270(2)	1547(2)	59(1)
C(11)	6658(3)	10267(2)	1822(2)	63(1)
C(12)	6272(3)	10284(2)	2903(2)	65(1)
C(13)	6403(3)	9288(2)	3748(2)	56(1)
C(14)	7829(3)	3940(2)	9654(2)	68(1)
C(15)	8968(4)	3395(2)	10471(2)	80(1)
C(16)	7637(4)	2122(2)	7995(3)	90(1)
C(17)	7926(5)	1662(3)	7070(4)	119(1)
C(21)	7889(2)	6566(1)	7097(2)	42(1)
C(22)	9502(3)	6913(2)	6284(2)	57(1)
C(23)	10330(3)	7867(2)	6222(3)	77(1)
C(24)	9538(4)	8488(2)	6964(3)	86(1)
C(25)	7955(4)	8148(2)	7763(3)	81(1)
C(26)	7125(3)	7190(2)	7844(2)	59(1)

Table S10. Bond lengths [Å] and angles [°] for **12d**.

Cl(1)-C(10)	1.726(2)
P(1)-O(1)	1.4673(14)
P(1)-O(2)	1.5655(15)
P(1)-O(3)	1.5668(15)
P(1)-C(2)	1.8390(17)
F(1)-C(11)	1.352(2)
O(2)-C(14)	1.468(2)
O(3)-C(16)	1.442(3)
O(6)-C(6)	1.220(2)
N(1)-C(5)	1.262(3)
N(1)-C(2)	1.483(2)
N(7)-C(6)	1.351(2)
N(7)-C(8)	1.417(2)
C(2)-C(21)	1.522(2)
C(2)-C(3)	1.578(2)
C(3)-C(6)	1.523(2)
C(3)-C(4)	1.540(3)
C(4)-C(5)	1.494(3)
C(8)-C(9)	1.383(3)
C(8)-C(13)	1.393(2)
C(9)-C(10)	1.380(3)
C(10)-C(11)	1.377(3)
C(11)-C(12)	1.360(3)
C(12)-C(13)	1.387(3)
C(14)-C(15)	1.481(3)
C(16)-C(17)	1.439(5)
C(21)-C(26)	1.387(2)
C(21)-C(22)	1.389(3)
C(22)-C(23)	1.384(3)
C(23)-C(24)	1.382(4)
C(24)-C(25)	1.364(4)
C(25)-C(26)	1.381(3)
O(1)-P(1)-O(2)	108.88(9)
O(1)-P(1)-O(3)	115.48(9)
O(2)-P(1)-O(3)	107.87(8)

O(1)-P(1)-C(2)	113.67(8)
O(2)-P(1)-C(2)	109.45(8)
O(3)-P(1)-C(2)	101.11(8)
C(14)-O(2)-P(1)	125.77(14)
C(16)-O(3)-P(1)	125.08(16)
C(5)-N(1)-C(2)	108.59(15)
C(6)-N(7)-C(8)	126.00(14)
N(1)-C(2)-C(21)	112.24(13)
N(1)-C(2)-C(3)	105.82(13)
C(21)-C(2)-C(3)	113.83(13)
N(1)-C(2)-P(1)	106.67(11)
C(21)-C(2)-P(1)	108.63(11)
C(3)-C(2)-P(1)	109.40(10)
C(6)-C(3)-C(4)	108.50(14)
C(6)-C(3)-C(2)	111.95(12)
C(4)-C(3)-C(2)	102.38(14)
C(5)-C(4)-C(3)	101.85(15)
N(1)-C(5)-C(4)	117.66(17)
O(6)-C(6)-N(7)	123.99(16)
O(6)-C(6)-C(3)	121.62(16)
N(7)-C(6)-C(3)	114.33(13)
C(9)-C(8)-C(13)	119.47(17)
C(9)-C(8)-N(7)	117.45(15)
C(13)-C(8)-N(7)	123.07(17)
C(10)-C(9)-C(8)	120.20(17)
C(11)-C(10)-C(9)	119.7(2)
C(11)-C(10)-Cl(1)	119.88(17)
C(9)-C(10)-Cl(1)	120.43(16)
F(1)-C(11)-C(12)	120.14(19)
F(1)-C(11)-C(10)	118.9(2)
C(12)-C(11)-C(10)	120.92(19)
C(11)-C(12)-C(13)	120.05(18)
C(12)-C(13)-C(8)	119.66(19)
O(2)-C(14)-C(15)	107.5(2)
C(17)-C(16)-O(3)	111.4(3)
C(26)-C(21)-C(22)	119.00(18)
C(26)-C(21)-C(2)	120.81(17)

C(22)-C(21)-C(2)	120.13(15)
C(23)-C(22)-C(21)	120.3(2)
C(24)-C(23)-C(22)	120.0(2)
C(25)-C(24)-C(23)	119.8(2)
C(24)-C(25)-C(26)	120.9(2)
C(25)-C(26)-C(21)	120.0(2)

Table S11. Anisotropic displacement parameters ($Å^2$x 10^3) for **12d**. The anisotropic displacement factor exponent takes the form: $-2\pi^2[h^2a^{*2}U^{11} + ... + 2 h k a^* b^* U^{12}]$.

	U^{11}	U^{22}	U^{33}	U^{23}	U^{13}	U^{12}
Cl(1)	158(1)	79(1)	50(1)	-15(1)	-20(1)	17(1)
P(1)	43(1)	37(1)	45(1)	-13(1)	-7(1)	4(1)
F(1)	126(1)	49(1)	78(1)	1(1)	-14(1)	11(1)
O(1)	52(1)	49(1)	58(1)	-20(1)	-1(1)	13(1)
O(2)	53(1)	60(1)	48(1)	-10(1)	-14(1)	1(1)
O(3)	63(1)	40(1)	77(1)	-15(1)	-19(1)	-2(1)
O(6)	55(1)	55(1)	57(1)	-23(1)	-6(1)	19(1)
N(1)	43(1)	53(1)	47(1)	-22(1)	2(1)	1(1)
N(7)	48(1)	39(1)	46(1)	-18(1)	-8(1)	8(1)
C(2)	36(1)	39(1)	40(1)	-17(1)	-6(1)	4(1)
C(3)	42(1)	42(1)	43(1)	-21(1)	-10(1)	4(1)
C(4)	50(1)	56(1)	67(1)	-24(1)	-18(1)	-6(1)
C(5)	41(1)	59(1)	68(1)	-25(1)	1(1)	-6(1)
C(6)	39(1)	45(1)	45(1)	-20(1)	-13(1)	6(1)
C(8)	43(1)	40(1)	51(1)	-16(1)	-10(1)	3(1)
C(9)	58(1)	43(1)	52(1)	-19(1)	-11(1)	6(1)
C(10)	70(1)	52(1)	50(1)	-12(1)	-12(1)	3(1)
C(11)	71(1)	42(1)	65(1)	-6(1)	-11(1)	2(1)
C(12)	77(1)	38(1)	76(1)	-21(1)	-13(1)	4(1)
C(13)	65(1)	45(1)	59(1)	-24(1)	-11(1)	2(1)
C(14)	72(1)	79(2)	45(1)	-16(1)	-11(1)	8(1)
C(15)	100(2)	82(2)	57(1)	-20(1)	-29(1)	21(1)
C(16)	86(2)	48(1)	126(2)	-15(1)	-19(2)	-3(1)
C(17)	86(2)	112(3)	192(4)	-104(3)	-17(2)	1(2)
C(21)	42(1)	39(1)	52(1)	-19(1)	-19(1)	7(1)
C(22)	45(1)	48(1)	80(1)	-25(1)	-14(1)	2(1)
C(23)	51(1)	57(1)	123(2)	-26(1)	-22(1)	-7(1)
C(24)	77(2)	57(1)	152(3)	-48(2)	-52(2)	0(1)
C(25)	91(2)	69(2)	110(2)	-58(2)	-41(2)	10(1)
C(26)	65(1)	58(1)	66(1)	-35(1)	-21(1)	7(1)

Table S12. Hydrogen bonds for **12d** [Å and °].

D-H...A	d(D-H)	d(H...A)	d(D...A)	<(DHA)
N(7)-H(7)...O(1)#1	0.86	1.96	2.8189(19)	173.9
C(12)-H(12)...O(6)#2	0.93	2.63	3.523(3)	160.7
C(13)-H(13)...O(6)	0.93	2.42	2.899(3)	111.8

Symmetry transformations used to generate equivalent atoms:

#1 -x+2,-y+1,-z+1 #2 -x+1,-y+2,-z+1

Diethyl [(2*RS*,3*RS*) -3-(cyclohexylcarbamoyl)-2-phenyl-3,4-dihydro-2*H*-pyrrol-2-yl]phosphonate, 12h.

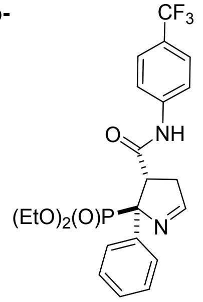

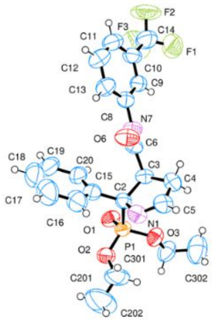

Table S13. Crystal data and structure refinement for **12h**.

Identification code	**12h**	
Empirical formula	C88 H96 F12 N8 O16 P4	
Formula weight	1873.60	
Temperature	294(2) K	
Wavelength	0.71073 Å	
Crystal system	Monoclinic	
Space group	P 21/a	
Unit cell dimensions	a = 22.488(2) Å	α= 90°.
	b = 16.7520(18) Å	β= 114.281(2)°.
	c = 27.123(3) Å	γ = 90°.
Volume	9313.9(17) Å^3	
Z	16	
Density (calculated)	1.336 Mg/m^3	
Absorption coefficient	0.172 mm^{-1}	
F(000)	3904	
Crystal size	0.180 x 0.180 x 0.110 mm^3	
Theta range for data collection	1.468 to 28.324°.	
Index ranges	-29<=h<=30, -22<=k<=22, -36<=l<=36	
Reflections collected	202790	
Independent reflections	23123 [R(int) = 0.1381]	
Completeness to theta = 25.242°	100.0 %	
Absorption correction	Semi-empirical from equivalents	
Max. and min. transmission	1 and 0.89	
Refinement method	Full-matrix least-squares on F^2	
Data / restraints / parameters	23123 / 56 / 1280	
Goodness-of-fit on F^2	1.033	
Final R indices [I>2sigma(I)]	R1 = 0.0984, wR2 = 0.2769	
R indices (all data)	R1 = 0.1995, wR2 = 0.3334	
Extinction coefficient	0.0012(2)	
Largest diff. peak and hole	0.429 and -0.363 e.Å^{-3}	

Table S14. Atomic coordinates (x 10^4) and equivalent isotropic displacement parameters ($Å^2$x 10^3) for **12h**. U(eq) is defined as one third of the trace of the orthogonalized U^{ij} tensor.

	x	y	z	U(eq)
P(1)	6776(1)	1159(1)	10230(1)	57(1)
P(2)	6129(1)	3135(1)	6606(1)	63(1)
P(3)	6538(1)	3735(1)	4760(1)	60(1)
P(4)	4513(1)	1868(1)	8365(1)	63(1)
F(1)	4507(6)	4862(9)	7522(3)	162(7)
F(2)	4288(8)	5994(4)	7721(4)	190(9)
F(3)	3849(5)	4976(11)	7868(5)	176(7)
F(1A)*	4600(17)	5570(40)	7539(16)	200(30)
F(2A)*	3960(20)	5750(30)	7906(14)	200(30)
F(3A)*	4090(30)	4615(16)	7666(15)	180(30)
F(4)	3939(15)	3630(20)	2424(9)	209(16)
F(5)	4892(8)	3380(20)	3005(10)	154(11)
F(6)	4259(14)	2466(10)	2626(11)	161(10)
F(4A)*	3977(18)	2870(30)	2371(11)	270(20)
F(5A)*	4279(17)	3986(11)	2700(13)	154(11)
F(6A)*	4895(10)	3020(20)	2996(15)	176(16)
F(7)	7094(9)	-70(30)	7560(12)	251(18)
F(8)	6218(10)	-648(12)	7383(7)	137(8)
F(9)	6250(30)	607(12)	7386(14)	232(18)
F(7A)*	6950(11)	184(16)	7648(8)	175(12)
F(8A)*	6380(20)	-806(12)	7333(12)	260(20)
F(9A)*	5934(12)	310(20)	7176(10)	190(13)
F(10)	6709(16)	2511(10)	12348(17)	240(20)
F(11)	6886(14)	1420(30)	12074(14)	220(20)
F(12)	6415(16)	1460(30)	12590(9)	260(30)
F(10A)*	6598(8)	2475(8)	12443(5)	188(9)
F(11A)*	6867(5)	1676(11)	11984(5)	122(6)
F(12A)*	6513(8)	1246(9)	12521(7)	154(7)
O(1)	6081(2)	1174(2)	10120(2)	69(1)
O(2)	7168(2)	781(2)	10796(2)	72(1)
O(3)	6952(2)	693(2)	9811(2)	69(1)
O(4)	6336(2)	4010(3)	6792(2)	93(1)

O(5)	6492(2)	2755(2)	6336(2)	72(1)
O(6)	7024(2)	3853(2)	9795(2)	83(1)
O(6A)	6207(2)	2653(3)	7116(2)	100(2)
O(7)	5971(2)	3759(2)	4895(2)	72(1)
O(8)	6350(2)	4077(3)	4184(2)	84(1)
O(9)	7149(2)	4202(2)	5160(2)	75(1)
O(10)	5121(2)	2306(3)	8609(2)	81(1)
O(11)	4022(3)	2204(4)	7821(2)	131(2)
O(12)	4598(3)	991(3)	8222(2)	100(2)
O(26)	4023(2)	3515(3)	5183(2)	77(1)
O(46)	7202(3)	1048(3)	5244(2)	92(1)
O(66)	3844(2)	1583(3)	9795(2)	74(1)
N(1)	7799(2)	2045(3)	10324(2)	68(1)
N(7)	5979(2)	3522(3)	9241(2)	63(1)
N(21)	4955(3)	3664(4)	6499(2)	87(2)
N(27)	4669(2)	3474(3)	4727(2)	62(1)
N(41)	7442(2)	2807(3)	4654(2)	73(1)
N(47)	6749(2)	1433(3)	5815(2)	64(1)
N(61)	3494(2)	1264(4)	8505(2)	81(2)
N(67)	4948(2)	1576(3)	10271(2)	59(1)
C(2)	7102(2)	2150(3)	10204(2)	55(1)
C(3)	6771(3)	2460(3)	9605(2)	61(1)
C(4)	7299(3)	2317(4)	9394(3)	77(2)
C(5)	7884(3)	2130(3)	9893(3)	72(2)
C(6)	6610(3)	3356(3)	9566(2)	63(1)
C(8)	5720(3)	4303(3)	9104(2)	61(1)
C(9)	5215(3)	4414(3)	8601(2)	67(2)
C(10)	4934(3)	5144(4)	8446(3)	77(2)
C(11)	5151(4)	5799(4)	8797(3)	89(2)
C(12)	5632(4)	5666(4)	9294(3)	92(2)
C(13)	5917(3)	4939(4)	9449(3)	80(2)
C(14)	4419(5)	5258(5)	7902(4)	114(3)
C(15)	7018(3)	2664(3)	10632(3)	69(2)
C(16)	7543(4)	2842(4)	11114(3)	97(2)
C(17)	7446(6)	3310(6)	11490(4)	129(3)
C(18)	6851(9)	3604(6)	11405(5)	147(5)
C(19)	6335(6)	3442(5)	10946(5)	125(4)

C(20)	6403(4)	2951(4)	10546(3)	85(2)
C(22)	5247(3)	3232(3)	6171(2)	58(1)
C(23)	5155(3)	3791(3)	5683(2)	59(1)
C(24)	5035(4)	4605(4)	5890(3)	88(2)
C(25)	4864(4)	4381(5)	6328(3)	100(2)
C(26)	4556(3)	3566(3)	5181(2)	58(1)
C(28)	4221(3)	3309(3)	4196(2)	62(1)
C(29)	4451(3)	3332(3)	3802(2)	66(1)
C(30)	4035(3)	3169(4)	3265(3)	76(2)
C(31)	3393(4)	2980(4)	3129(3)	91(2)
C(32)	3165(3)	2960(5)	3521(3)	93(2)
C(33)	3565(3)	3126(4)	4049(3)	78(2)
C(34)	4286(4)	3204(6)	2839(4)	115(3)
C(35)	4980(3)	2381(3)	6033(2)	62(1)
C(36)	5118(3)	1940(3)	5660(3)	75(2)
C(37)	4896(4)	1165(4)	5535(4)	108(3)
C(38)	4528(5)	818(5)	5779(5)	133(4)
C(39)	4419(4)	1258(6)	6162(4)	126(4)
C(40)	4629(3)	2037(4)	6281(3)	90(2)
C(42)	6870(2)	2731(3)	4795(2)	58(1)
C(43)	7155(3)	2446(3)	5404(2)	64(1)
C(44)	7894(3)	2590(4)	5593(3)	80(2)
C(45)	7962(3)	2743(4)	5085(3)	75(2)
C(46)	7030(3)	1572(3)	5472(2)	66(2)
C(48)	6628(3)	682(3)	5993(3)	67(2)
C(49)	6596(3)	668(4)	6492(3)	75(2)
C(50)	6483(3)	-45(4)	6690(3)	83(2)
C(51)	6405(4)	-751(5)	6398(4)	106(3)
C(52)	6425(4)	-716(5)	5907(4)	105(2)
C(53)	6539(3)	-6(4)	5702(3)	83(2)
C(54)	6470(6)	-48(6)	7227(4)	138(4)
C(55)	6345(3)	2211(3)	4393(2)	64(1)
C(56)	5815(3)	1976(3)	4495(3)	72(2)
C(57)	5322(4)	1505(4)	4134(4)	96(2)
C(58)	5370(5)	1260(5)	3667(4)	111(3)
C(59)	5870(5)	1496(6)	3557(3)	111(3)
C(60)	6361(4)	1970(4)	3912(3)	85(2)

C(62)	4074(2)	1764(3)	8802(2)	58(1)
C(63)	4509(3)	1267(3)	9324(2)	57(1)
C(64)	4242(4)	425(4)	9162(3)	82(2)
C(65)	3622(4)	564(5)	8709(4)	101(3)
C(66)	4398(3)	1507(3)	9816(2)	59(1)
C(68)	5017(3)	1727(3)	10801(2)	56(1)
C(69)	5638(3)	1694(3)	11205(2)	61(1)
C(70)	5757(3)	1851(3)	11739(2)	67(2)
C(71)	5234(3)	2050(4)	11866(3)	78(2)
C(72)	4626(3)	2088(4)	11470(2)	75(2)
C(73)	4500(3)	1928(3)	10931(2)	67(2)
C(74)	6416(4)	1810(5)	12164(3)	105(2)
C(75)	3898(3)	2603(3)	8906(2)	64(1)
C(76)	4363(4)	3099(3)	9254(3)	79(2)
C(77)	4212(5)	3897(5)	9318(4)	117(3)
C(78)	3598(7)	4180(6)	9031(6)	142(5)
C(79)	3132(6)	3691(7)	8684(5)	140(4)
C(80)	3271(4)	2912(5)	8610(3)	99(2)
C(111)	3853(8)	2815(12)	7575(5)	263(10)
C(112)	3283(6)	2990(8)	7113(4)	194(6)
C(120)	4949(7)	773(7)	7902(6)	191(6)
C(121)	4803(10)	43(8)	7674(8)	286(11)
C(201)	7844(4)	478(5)	10980(4)	119(3)
C(202)	8043(5)	57(9)	11441(5)	206(7)
C(301)	6637(4)	-59(4)	9561(3)	90(2)
C(302)	6781(5)	-224(6)	9098(4)	143(4)
C(401)	7025(8)	4211(9)	6976(9)	288(12)
C(402)	7191(8)	4934(9)	7150(7)	264(9)
C(601)	6082(9)	2950(10)	7549(4)	245(9)
C(602)	6251(8)	2490(12)	7978(5)	273(10)
C(801)	6805(5)	4382(7)	3984(5)	154(5)
C(802)	6752(8)	4133(13)	3491(6)	171(10)
C(803)*	6668(12)	5053(15)	3657(10)	99(9)
C(901)	7087(3)	4978(4)	5385(3)	94(2)
C(902)	7707(4)	5168(6)	5844(4)	134(3)

* disordered position

Table S15. Bond lengths [Å] and angles [°] for **12h**.

P(1)-O(1)	1.464(4)
P(1)-O(2)	1.556(4)
P(1)-O(3)	1.559(4)
P(1)-C(2)	1.828(5)
P(2)-O(5)	1.450(4)
P(2)-O(6A)	1.548(5)
P(2)-O(4)	1.559(4)
P(2)-C(22)	1.851(5)
P(3)-O(7)	1.465(4)
P(3)-O(8)	1.551(4)
P(3)-O(9)	1.566(4)
P(3)-C(42)	1.828(5)
P(4)-O(10)	1.451(4)
P(4)-O(11)	1.539(6)
P(4)-O(12)	1.552(5)
P(4)-C(62)	1.836(5)
F(1)-F(1A)	1.21(5)
F(1)-F(3A)	1.23(6)
F(1)-C(14)	1.307(10)
F(2)-F(2A)	1.13(4)
F(2)-F(1A)	1.23(4)
F(2)-C(14)	1.315(10)
F(3)-F(3A)	1.10(6)
F(3)-F(2A)	1.32(5)
F(3)-C(14)	1.334(12)
F(1A)-C(14)	1.321(15)
F(2A)-C(14)	1.327(16)
F(3A)-C(14)	1.315(18)
F(4)-F(5A)	1.02(2)
F(4)-C(34)	1.284(13)
F(4)-F(4A)	1.29(3)
F(5)-F(6A)	0.62(4)
F(5)-C(34)	1.283(14)
F(5)-F(5A)	1.63(3)
F(6)-F(4A)	0.98(4)

F(6)-C(34)	1.356(14)
F(6)-F(6A)	1.65(3)
F(4A)-C(34)	1.301(15)
F(5A)-C(34)	1.361(14)
F(6A)-C(34)	1.296(16)
F(7)-F(7A)	0.64(5)
F(7)-C(54)	1.321(16)
F(8)-F(8A)	0.52(5)
F(8)-C(54)	1.308(14)
F(8)-F(9A)	1.73(3)
F(9)-F(9A)	0.87(4)
F(9)-C(54)	1.340(15)
F(9)-F(7A)	1.59(4)
F(7A)-C(54)	1.267(15)
F(8A)-C(54)	1.334(15)
F(9A)-C(54)	1.302(17)
F(10)-C(74)	1.338(15)
F(11)-C(74)	1.347(16)
F(11)-F(12A)	1.76(4)
F(12)-F(12A)	0.50(6)
F(12)-C(74)	1.294(15)
F(10A)-C(74)	1.313(12)
F(11A)-C(74)	1.314(11)
F(12A)-C(74)	1.305(11)
O(2)-C(201)	1.482(7)
O(3)-C(301)	1.467(7)
O(4)-C(401)	1.458(14)
O(6)-C(6)	1.212(6)
O(6A)-C(601)	1.409(12)
O(8)-C(801)	1.434(9)
O(9)-C(901)	1.467(7)
O(11)-C(111)	1.197(14)
O(12)-C(120)	1.437(11)
O(26)-C(26)	1.203(6)
O(46)-C(46)	1.224(6)
O(66)-C(66)	1.230(6)
N(1)-C(5)	1.269(7)

N(1)-C(2)	1.475(6)
N(7)-C(6)	1.356(7)
N(7)-C(8)	1.418(7)
N(21)-C(25)	1.272(9)
N(21)-C(22)	1.493(7)
N(27)-C(26)	1.364(7)
N(27)-C(28)	1.405(7)
N(41)-C(45)	1.274(7)
N(41)-C(42)	1.490(7)
N(47)-C(46)	1.345(7)
N(47)-C(48)	1.412(7)
N(61)-C(65)	1.277(9)
N(61)-C(62)	1.481(7)
N(67)-C(66)	1.345(7)
N(67)-C(68)	1.404(7)
C(2)-C(15)	1.519(8)
C(2)-C(3)	1.573(7)
C(3)-C(4)	1.536(8)
C(3)-C(6)	1.536(7)
C(4)-C(5)	1.481(8)
C(8)-C(13)	1.366(8)
C(8)-C(9)	1.384(8)
C(9)-C(10)	1.362(8)
C(10)-C(11)	1.402(9)
C(10)-C(14)	1.465(10)
C(11)-C(12)	1.356(10)
C(12)-C(13)	1.360(9)
C(15)-C(16)	1.387(9)
C(15)-C(20)	1.389(9)
C(16)-C(17)	1.372(12)
C(17)-C(18)	1.354(15)
C(18)-C(19)	1.336(15)
C(19)-C(20)	1.419(11)
C(22)-C(35)	1.532(8)
C(22)-C(23)	1.562(7)
C(23)-C(26)	1.518(8)
C(23)-C(24)	1.540(8)

C(24)-C(25)	1.441(10)
C(28)-C(29)	1.366(8)
C(28)-C(33)	1.394(8)
C(29)-C(30)	1.395(8)
C(30)-C(31)	1.372(9)
C(30)-C(34)	1.481(10)
C(31)-C(32)	1.356(10)
C(32)-C(33)	1.371(9)
C(35)-C(40)	1.358(8)
C(35)-C(36)	1.387(8)
C(36)-C(37)	1.383(9)
C(37)-C(38)	1.381(13)
C(38)-C(39)	1.376(14)
C(39)-C(40)	1.380(12)
C(42)-C(55)	1.511(8)
C(42)-C(43)	1.579(7)
C(43)-C(46)	1.516(8)
C(43)-C(44)	1.541(8)
C(44)-C(45)	1.471(9)
C(48)-C(53)	1.364(8)
C(48)-C(49)	1.385(8)
C(49)-C(50)	1.375(9)
C(50)-C(51)	1.393(11)
C(50)-C(54)	1.471(12)
C(51)-C(52)	1.352(11)
C(52)-C(53)	1.381(10)
C(55)-C(60)	1.381(8)
C(55)-C(56)	1.386(8)
C(56)-C(57)	1.385(9)
C(57)-C(58)	1.376(11)
C(58)-C(59)	1.335(12)
C(59)-C(60)	1.379(11)
C(62)-C(75)	1.517(8)
C(62)-C(63)	1.585(7)
C(63)-C(66)	1.509(7)
C(63)-C(64)	1.526(8)
C(64)-C(65)	1.448(10)

C(68)-C(69)	1.377(7)
C(68)-C(73)	1.388(7)
C(69)-C(70)	1.386(8)
C(70)-C(71)	1.394(8)
C(70)-C(74)	1.458(9)
C(71)-C(72)	1.350(8)
C(72)-C(73)	1.395(8)
C(75)-C(76)	1.366(8)
C(75)-C(80)	1.402(8)
C(76)-C(77)	1.408(9)
C(77)-C(78)	1.361(14)
C(78)-C(79)	1.358(15)
C(79)-C(80)	1.376(13)
C(111)-C(112)	1.406(13)
C(120)-C(121)	1.349(12)
C(201)-C(202)	1.341(10)
C(301)-C(302)	1.447(9)
C(401)-C(402)	1.299(13)
C(601)-C(602)	1.313(13)
C(801)-C(802)	1.358(13)
C(801)-C(803)	1.38(2)
C(901)-C(902)	1.472(9)
O(1)-P(1)-O(2)	109.5(2)
O(1)-P(1)-O(3)	115.4(2)
O(2)-P(1)-O(3)	107.7(2)
O(1)-P(1)-C(2)	112.8(2)
O(2)-P(1)-C(2)	109.5(2)
O(3)-P(1)-C(2)	101.5(2)
O(5)-P(2)-O(6A)	110.0(3)
O(5)-P(2)-O(4)	114.9(3)
O(6A)-P(2)-O(4)	107.0(3)
O(5)-P(2)-C(22)	113.3(2)
O(6A)-P(2)-C(22)	108.3(3)
O(4)-P(2)-C(22)	102.9(2)
O(7)-P(3)-O(8)	109.8(2)
O(7)-P(3)-O(9)	114.7(2)
O(8)-P(3)-O(9)	108.2(2)

O(7)-P(3)-C(42)	113.0(2)
O(8)-P(3)-C(42)	109.2(3)
O(9)-P(3)-C(42)	101.5(2)
O(10)-P(4)-O(11)	115.0(3)
O(10)-P(4)-O(12)	113.8(3)
O(11)-P(4)-O(12)	102.8(3)
O(10)-P(4)-C(62)	114.2(2)
O(11)-P(4)-C(62)	106.8(3)
O(12)-P(4)-C(62)	103.1(2)
F(1A)-F(1)-F(3A)	118(2)
F(1A)-F(1)-C(14)	63.3(15)
F(3A)-F(1)-C(14)	62.4(16)
F(2A)-F(2)-F(1A)	124.0(19)
F(2A)-F(2)-C(14)	65.4(11)
F(1A)-F(2)-C(14)	62.4(13)
F(3A)-F(3)-F(2A)	118(2)
F(3A)-F(3)-C(14)	64.6(18)
F(2A)-F(3)-C(14)	60.0(14)
F(1)-F(1A)-F(2)	117.5(18)
F(1)-F(1A)-C(14)	62.1(13)
F(2)-F(1A)-C(14)	61.8(11)
F(2)-F(2A)-F(3)	117(2)
F(2)-F(2A)-C(14)	64.2(13)
F(3)-F(2A)-C(14)	60.5(15)
F(3)-F(3A)-F(1)	124.9(19)
F(3)-F(3A)-C(14)	66.4(18)
F(1)-F(3A)-C(14)	61.7(17)
F(5A)-F(4)-C(34)	71.4(11)
F(5A)-F(4)-F(4A)	127(2)
C(34)-F(4)-F(4A)	60.8(12)
F(6A)-F(5)-C(34)	77(2)
F(6A)-F(5)-F(5A)	128(3)
C(34)-F(5)-F(5A)	54.1(9)
F(4A)-F(6)-C(34)	65.3(13)
F(4A)-F(6)-F(6A)	101(2)
C(34)-F(6)-F(6A)	49.8(10)
F(6)-F(4A)-F(4)	130(2)

F(6)-F(4A)-C(34)	71.3(15)
F(4)-F(4A)-C(34)	59.5(9)
F(4)-F(5A)-C(34)	63.4(12)
F(4)-F(5A)-F(5)	103.1(17)
C(34)-F(5A)-F(5)	49.7(9)
F(5)-F(6A)-C(34)	75(2)
F(5)-F(6A)-F(6)	124(3)
C(34)-F(6A)-F(6)	53.1(11)
F(7A)-F(7)-C(54)	71(2)
F(8A)-F(8)-C(54)	82(3)
F(8A)-F(8)-F(9A)	126(4)
C(54)-F(8)-F(9A)	48.2(9)
F(9A)-F(9)-C(54)	68.5(15)
F(9A)-F(9)-F(7A)	116(2)
C(54)-F(9)-F(7A)	50.3(11)
F(7)-F(7A)-C(54)	80(2)
F(7)-F(7A)-F(9)	133(3)
C(54)-F(7A)-F(9)	54.4(11)
F(8)-F(8A)-C(54)	76(2)
F(9)-F(9A)-C(54)	73.2(18)
F(9)-F(9A)-F(8)	103(2)
C(54)-F(9A)-F(8)	48.5(11)
C(74)-F(11)-F(12A)	47.4(12)
F(12A)-F(12)-C(74)	80(3)
F(12)-F(12A)-C(74)	78(2)
F(12)-F(12A)-F(11)	123(3)
C(74)-F(12A)-F(11)	49.4(12)
C(201)-O(2)-P(1)	124.0(4)
C(301)-O(3)-P(1)	123.2(3)
C(401)-O(4)-P(2)	117.5(6)
C(601)-O(6A)-P(2)	125.0(7)
C(801)-O(8)-P(3)	124.9(5)
C(901)-O(9)-P(3)	121.7(4)
C(111)-O(11)-P(4)	141.8(11)
C(120)-O(12)-P(4)	123.0(6)
C(5)-N(1)-C(2)	109.4(5)
C(6)-N(7)-C(8)	124.6(5)

C(25)-N(21)-C(22)	106.6(6)
C(26)-N(27)-C(28)	128.8(5)
C(45)-N(41)-C(42)	108.7(5)
C(46)-N(47)-C(48)	127.0(5)
C(65)-N(61)-C(62)	107.0(6)
C(66)-N(67)-C(68)	128.9(4)
N(1)-C(2)-C(15)	110.7(5)
N(1)-C(2)-C(3)	106.0(4)
C(15)-C(2)-C(3)	116.6(4)
N(1)-C(2)-P(1)	106.9(3)
C(15)-C(2)-P(1)	107.9(3)
C(3)-C(2)-P(1)	108.3(4)
C(4)-C(3)-C(6)	108.8(5)
C(4)-C(3)-C(2)	102.9(4)
C(6)-C(3)-C(2)	113.1(5)
C(5)-C(4)-C(3)	103.0(5)
N(1)-C(5)-C(4)	116.9(5)
O(6)-C(6)-N(7)	124.7(5)
O(6)-C(6)-C(3)	121.8(5)
N(7)-C(6)-C(3)	113.5(5)
C(13)-C(8)-C(9)	118.4(6)
C(13)-C(8)-N(7)	123.9(5)
C(9)-C(8)-N(7)	117.7(5)
C(10)-C(9)-C(8)	120.9(6)
C(9)-C(10)-C(11)	120.3(6)
C(9)-C(10)-C(14)	120.1(7)
C(11)-C(10)-C(14)	119.5(6)
C(12)-C(11)-C(10)	117.4(6)
C(11)-C(12)-C(13)	122.5(7)
C(12)-C(13)-C(8)	120.5(7)
F(1)-C(14)-F(2)	105.4(9)
F(1)-C(14)-F(3A)	56(3)
F(2)-C(14)-F(3A)	126.2(17)
F(1)-C(14)-F(1A)	55(2)
F(2)-C(14)-F(1A)	55.8(19)
F(3A)-C(14)-F(1A)	104.4(17)
F(1)-C(14)-F(2A)	133.9(19)

F(2)-C(14)-F(2A)	50.4(18)
F(3A)-C(14)-F(2A)	103.2(17)
F(1A)-C(14)-F(2A)	103.7(14)
F(1)-C(14)-F(3)	102.7(11)
F(2)-C(14)-F(3)	104.2(9)
F(3A)-C(14)-F(3)	49(3)
F(1A)-C(14)-F(3)	132(2)
F(2A)-C(14)-F(3)	59(2)
F(1)-C(14)-C(10)	114.5(7)
F(2)-C(14)-C(10)	117.4(9)
F(3A)-C(14)-C(10)	116.0(16)
F(1A)-C(14)-C(10)	116.3(19)
F(2A)-C(14)-C(10)	111.6(19)
F(3)-C(14)-C(10)	111.3(8)
C(16)-C(15)-C(20)	119.2(7)
C(16)-C(15)-C(2)	121.1(6)
C(20)-C(15)-C(2)	119.7(6)
C(17)-C(16)-C(15)	119.3(9)
C(18)-C(17)-C(16)	121.9(10)
C(19)-C(18)-C(17)	120.2(11)
C(18)-C(19)-C(20)	120.4(11)
C(15)-C(20)-C(19)	118.9(9)
N(21)-C(22)-C(35)	111.9(5)
N(21)-C(22)-C(23)	106.0(4)
C(35)-C(22)-C(23)	116.7(4)
N(21)-C(22)-P(2)	106.3(4)
C(35)-C(22)-P(2)	106.5(3)
C(23)-C(22)-P(2)	109.0(4)
C(26)-C(23)-C(24)	108.8(5)
C(26)-C(23)-C(22)	111.8(4)
C(24)-C(23)-C(22)	101.8(5)
C(25)-C(24)-C(23)	102.4(5)
N(21)-C(25)-C(24)	119.2(6)
O(26)-C(26)-N(27)	123.4(5)
O(26)-C(26)-C(23)	122.1(5)
N(27)-C(26)-C(23)	114.4(5)
C(29)-C(28)-C(33)	118.6(6)

C(29)-C(28)-N(27)	117.0(5)
C(33)-C(28)-N(27)	124.4(6)
C(28)-C(29)-C(30)	120.2(6)
C(31)-C(30)-C(29)	120.4(6)
C(31)-C(30)-C(34)	119.7(7)
C(29)-C(30)-C(34)	119.9(6)
C(32)-C(31)-C(30)	119.4(7)
C(31)-C(32)-C(33)	121.0(7)
C(32)-C(33)-C(28)	120.5(7)
F(5)-C(34)-F(4)	110.7(14)
F(5)-C(34)-F(6A)	27.6(18)
F(4)-C(34)-F(6A)	127(2)
F(5)-C(34)-F(4A)	121(2)
F(4)-C(34)-F(4A)	59.8(14)
F(6A)-C(34)-F(4A)	106.5(16)
F(5)-C(34)-F(6)	102.7(13)
F(4)-C(34)-F(6)	102.9(12)
F(6A)-C(34)-F(6)	77.1(18)
F(4A)-C(34)-F(6)	43.4(17)
F(5)-C(34)-F(5A)	76.2(15)
F(4)-C(34)-F(5A)	45.2(10)
F(6A)-C(34)-F(5A)	102.3(14)
F(4A)-C(34)-F(5A)	102.1(14)
F(6)-C(34)-F(5A)	140.1(12)
F(5)-C(34)-C(30)	115.2(14)
F(4)-C(34)-C(30)	114.6(11)
F(6A)-C(34)-C(30)	115.1(18)
F(4A)-C(34)-C(30)	121.5(16)
F(6)-C(34)-C(30)	109.3(11)
F(5A)-C(34)-C(30)	106.8(11)
C(40)-C(35)-C(36)	119.1(6)
C(40)-C(35)-C(22)	121.6(6)
C(36)-C(35)-C(22)	119.3(5)
C(37)-C(36)-C(35)	120.7(7)
C(38)-C(37)-C(36)	120.4(9)
C(39)-C(38)-C(37)	117.6(8)
C(38)-C(39)-C(40)	122.2(9)

C(35)-C(40)-C(39)	119.9(8)
N(41)-C(42)-C(55)	111.3(4)
N(41)-C(42)-C(43)	105.9(4)
C(55)-C(42)-C(43)	116.2(4)
N(41)-C(42)-P(3)	106.6(4)
C(55)-C(42)-P(3)	108.1(4)
C(43)-C(42)-P(3)	108.3(4)
C(46)-C(43)-C(44)	109.8(5)
C(46)-C(43)-C(42)	113.7(5)
C(44)-C(43)-C(42)	102.7(4)
C(45)-C(44)-C(43)	103.2(5)
N(41)-C(45)-C(44)	117.6(5)
O(46)-C(46)-N(47)	124.1(5)
O(46)-C(46)-C(43)	121.3(5)
N(47)-C(46)-C(43)	114.6(5)
C(53)-C(48)-C(49)	119.8(6)
C(53)-C(48)-N(47)	124.0(6)
C(49)-C(48)-N(47)	116.3(5)
C(50)-C(49)-C(48)	119.2(6)
C(49)-C(50)-C(51)	121.1(7)
C(49)-C(50)-C(54)	118.2(7)
C(51)-C(50)-C(54)	120.7(7)
C(52)-C(51)-C(50)	118.3(7)
C(51)-C(52)-C(53)	121.4(8)
C(48)-C(53)-C(52)	120.1(7)
F(7A)-C(54)-F(9A)	111.2(17)
F(7A)-C(54)-F(8)	105.5(16)
F(9A)-C(54)-F(8)	83.3(17)
F(7A)-C(54)-F(7)	29(2)
F(9A)-C(54)-F(7)	139.8(19)
F(8)-C(54)-F(7)	103.7(14)
F(7A)-C(54)-F(8A)	104.1(15)
F(9A)-C(54)-F(8A)	104.6(16)
F(8)-C(54)-F(8A)	23(2)
F(7)-C(54)-F(8A)	92(2)
F(7A)-C(54)-F(9)	75(2)
F(9A)-C(54)-F(9)	38.3(16)

F(8)-C(54)-F(9)	105.3(15)
F(7)-C(54)-F(9)	103.1(14)
F(8A)-C(54)-F(9)	127(2)
F(7A)-C(54)-C(50)	122.4(14)
F(9A)-C(54)-C(50)	106.7(12)
F(8)-C(54)-C(50)	120.8(13)
F(7)-C(54)-C(50)	103.3(18)
F(8A)-C(54)-C(50)	106.3(18)
F(9)-C(54)-C(50)	118.2(11)
C(60)-C(55)-C(56)	117.5(6)
C(60)-C(55)-C(42)	122.4(6)
C(56)-C(55)-C(42)	120.0(5)
C(57)-C(56)-C(55)	121.7(7)
C(58)-C(57)-C(56)	118.4(8)
C(59)-C(58)-C(57)	120.6(8)
C(58)-C(59)-C(60)	121.4(8)
C(59)-C(60)-C(55)	120.2(7)
N(61)-C(62)-C(75)	112.7(5)
N(61)-C(62)-C(63)	105.6(4)
C(75)-C(62)-C(63)	115.6(4)
N(61)-C(62)-P(4)	106.9(4)
C(75)-C(62)-P(4)	106.5(4)
C(63)-C(62)-P(4)	109.2(3)
C(66)-C(63)-C(64)	108.2(5)
C(66)-C(63)-C(62)	112.6(4)
C(64)-C(63)-C(62)	101.8(5)
C(65)-C(64)-C(63)	102.8(5)
N(61)-C(65)-C(64)	118.9(6)
O(66)-C(66)-N(67)	124.4(5)
O(66)-C(66)-C(63)	121.2(5)
N(67)-C(66)-C(63)	114.3(5)
C(69)-C(68)-C(73)	119.4(5)
C(69)-C(68)-N(67)	117.1(5)
C(73)-C(68)-N(67)	123.6(5)
C(68)-C(69)-C(70)	121.3(5)
C(69)-C(70)-C(71)	118.9(5)
C(69)-C(70)-C(74)	121.0(6)

C(71)-C(70)-C(74)	120.1(6)
C(72)-C(71)-C(70)	119.8(6)
C(71)-C(72)-C(73)	121.8(6)
C(68)-C(73)-C(72)	118.8(6)
F(12)-C(74)-F(12A)	22(3)
F(12)-C(74)-F(10A)	89(2)
F(12A)-C(74)-F(10A)	105.6(11)
F(12)-C(74)-F(11A)	123(2)
F(12A)-C(74)-F(11A)	103.5(10)
F(10A)-C(74)-F(11A)	104.8(11)
F(12)-C(74)-F(10)	103.9(15)
F(12A)-C(74)-F(10)	116.9(19)
F(10A)-C(74)-F(10)	19(2)
F(11A)-C(74)-F(10)	87(2)
F(12)-C(74)-F(11)	103.7(15)
F(12A)-C(74)-F(11)	83(2)
F(10A)-C(74)-F(11)	116(2)
F(11A)-C(74)-F(11)	21(2)
F(10)-C(74)-F(11)	101.0(15)
F(12)-C(74)-C(70)	110.6(14)
F(12A)-C(74)-C(70)	115.2(10)
F(10A)-C(74)-C(70)	112.9(9)
F(11A)-C(74)-C(70)	113.7(9)
F(10)-C(74)-C(70)	116.0(14)
F(11)-C(74)-C(70)	119.8(15)
C(76)-C(75)-C(80)	118.3(6)
C(76)-C(75)-C(62)	120.5(5)
C(80)-C(75)-C(62)	120.9(6)
C(75)-C(76)-C(77)	120.6(8)
C(78)-C(77)-C(76)	119.9(10)
C(79)-C(78)-C(77)	119.9(10)
C(78)-C(79)-C(80)	121.1(10)
C(79)-C(80)-C(75)	120.1(9)
O(11)-C(111)-C(112)	129.7(18)
C(121)-C(120)-O(12)	114.6(11)
C(202)-C(201)-O(2)	113.4(7)
C(302)-C(301)-O(3)	109.1(6)

C(402)-C(401)-O(4)	116.5(14)
C(602)-C(601)-O(6A)	116.6(12)
C(802)-C(801)-O(8)	118.4(13)
C(803)-C(801)-O(8)	121.7(13)
O(9)-C(901)-C(902)	108.9(6)

Table S16. Anisotropic displacement parameters ($Å^2$x 10^3) for **12h**. The anisotropic displacement factor exponent takes the form: $-2\pi^2[h^2a^{*2}U^{11} + ... + 2 h k a^* b^* U^{12}]$.

	U^{11}	U^{22}	U^{33}	U^{23}	U^{13}	U^{12}
P(1)	54(1)	51(1)	75(1)	7(1)	36(1)	3(1)
P(2)	66(1)	66(1)	56(1)	-9(1)	23(1)	3(1)
P(3)	48(1)	58(1)	78(1)	9(1)	29(1)	3(1)
P(4)	72(1)	64(1)	58(1)	-5(1)	30(1)	-7(1)
F(1)	203(12)	190(15)	72(5)	13(6)	35(5)	82(11)
F(2)	291(17)	92(5)	121(8)	49(5)	19(9)	61(7)
F(3)	92(6)	256(18)	127(9)	24(9)	-10(5)	13(7)
F(1A)	230(40)	240(70)	190(40)	160(40)	140(30)	60(40)
F(2A)	210(40)	220(50)	190(30)	170(40)	110(30)	150(40)
F(3A)	210(60)	160(30)	100(30)	70(30)	-10(30)	-30(40)
F(4)	190(20)	370(40)	71(14)	70(20)	51(14)	120(20)
F(5)	123(18)	260(30)	107(14)	-18(15)	71(14)	-54(14)
F(6)	210(20)	178(15)	132(16)	-66(12)	110(16)	-24(13)
F(4A)	250(40)	450(70)	101(19)	-120(30)	70(20)	-120(40)
F(5A)	190(20)	183(16)	105(16)	67(12)	78(16)	41(14)
F(6A)	160(30)	250(30)	140(20)	50(20)	90(20)	100(20)
F(7)	210(20)	430(50)	90(14)	21(19)	40(15)	-60(20)
F(8)	141(13)	167(16)	92(9)	24(9)	38(8)	-55(9)
F(9)	510(50)	110(12)	131(19)	32(11)	190(30)	61(19)
F(7A)	210(20)	240(20)	86(11)	-60(13)	66(13)	-123(19)
F(8A)	410(50)	180(20)	220(30)	76(18)	140(30)	-90(20)
F(9A)	220(20)	280(30)	101(14)	26(15)	99(14)	-7(19)
F(10)	114(18)	84(17)	410(60)	40(20)	-10(20)	-33(14)
F(11)	190(30)	290(30)	110(20)	-10(19)	-3(16)	170(30)
F(12)	85(15)	600(80)	66(12)	140(20)	5(10)	10(30)
F(10A)	118(10)	230(20)	123(10)	-98(11)	-39(8)	32(10)
F(11A)	47(5)	228(16)	76(6)	16(7)	10(4)	7(7)
F(12A)	101(8)	186(13)	165(14)	109(9)	45(8)	38(7)
O(1)	56(2)	60(2)	99(3)	5(2)	40(2)	3(2)
O(2)	71(2)	71(2)	84(3)	22(2)	42(2)	16(2)
O(3)	71(2)	58(2)	94(3)	-10(2)	51(2)	-9(2)
O(4)	85(3)	80(3)	100(3)	-41(3)	25(3)	-12(2)

O(5)	65(2)	79(3)	72(2)	-17(2)	29(2)	2(2)
O(6)	66(3)	60(2)	102(3)	12(2)	15(2)	-17(2)
O(6A)	112(4)	123(4)	65(3)	10(3)	37(3)	6(3)
O(7)	55(2)	69(2)	102(3)	5(2)	43(2)	5(2)
O(8)	74(3)	91(3)	93(3)	35(2)	39(2)	8(2)
O(9)	54(2)	62(2)	106(3)	-9(2)	30(2)	-4(2)
O(10)	81(3)	85(3)	89(3)	-22(2)	48(2)	-28(2)
O(11)	143(5)	180(6)	76(3)	57(4)	50(3)	45(4)
O(12)	142(4)	74(3)	117(4)	-35(3)	88(4)	-24(3)
O(26)	61(2)	92(3)	84(3)	9(2)	34(2)	-4(2)
O(46)	130(4)	72(3)	98(3)	1(2)	71(3)	31(3)
O(66)	54(2)	100(3)	75(3)	5(2)	34(2)	7(2)
N(1)	47(3)	81(3)	78(3)	15(3)	27(2)	-2(2)
N(7)	55(3)	52(3)	70(3)	5(2)	14(2)	-5(2)
N(21)	92(4)	100(4)	85(4)	-12(3)	51(3)	20(3)
N(27)	47(2)	69(3)	67(3)	7(2)	21(2)	-2(2)
N(41)	56(3)	97(4)	74(3)	2(3)	33(3)	15(3)
N(47)	79(3)	56(3)	64(3)	2(2)	37(3)	12(2)
N(61)	60(3)	96(4)	77(3)	-11(3)	19(3)	-25(3)
N(67)	49(2)	74(3)	57(3)	5(2)	24(2)	6(2)
C(2)	52(3)	57(3)	58(3)	5(2)	25(3)	0(2)
C(3)	55(3)	54(3)	69(3)	8(3)	20(3)	4(2)
C(4)	87(4)	81(4)	73(4)	10(3)	42(4)	0(3)
C(5)	62(4)	72(4)	90(4)	7(3)	38(3)	-9(3)
C(6)	60(3)	61(3)	66(3)	10(3)	25(3)	-10(3)
C(8)	62(3)	55(3)	69(4)	-1(3)	29(3)	-11(3)
C(9)	75(4)	61(3)	63(4)	2(3)	26(3)	2(3)
C(10)	79(4)	71(4)	79(4)	20(3)	31(4)	14(3)
C(11)	97(5)	62(4)	113(6)	9(4)	47(5)	13(4)
C(12)	90(5)	64(4)	116(6)	-15(4)	39(5)	0(4)
C(13)	76(4)	69(4)	89(5)	-10(3)	28(4)	-2(3)
C(14)	129(8)	100(7)	101(7)	11(6)	34(6)	39(6)
C(15)	85(4)	55(3)	77(4)	7(3)	42(4)	-2(3)
C(16)	122(6)	81(5)	77(5)	-12(4)	31(5)	-17(4)
C(17)	170(10)	117(8)	89(6)	-28(5)	43(7)	-32(7)
C(18)	267(17)	89(7)	127(9)	-18(6)	124(11)	-16(9)
C(19)	201(11)	64(5)	169(9)	14(6)	135(9)	24(6)

C(20)	118(6)	60(4)	103(5)	8(3)	73(5)	9(4)
C(22)	56(3)	63(3)	62(3)	-5(3)	30(3)	7(3)
C(23)	59(3)	52(3)	67(3)	5(3)	28(3)	-1(2)
C(24)	89(5)	61(4)	101(5)	-5(4)	25(4)	7(3)
C(25)	110(6)	75(5)	121(6)	-21(5)	54(5)	27(4)
C(26)	51(3)	51(3)	76(4)	9(3)	29(3)	1(2)
C(28)	52(3)	54(3)	74(4)	7(3)	20(3)	3(2)
C(29)	60(3)	61(3)	76(4)	4(3)	27(3)	2(3)
C(30)	76(4)	80(4)	70(4)	11(3)	28(3)	11(3)
C(31)	75(5)	91(5)	84(5)	-2(4)	9(4)	6(4)
C(32)	68(4)	115(6)	82(5)	1(4)	15(4)	-12(4)
C(33)	63(4)	85(4)	79(4)	11(3)	22(3)	-12(3)
C(34)	101(7)	143(9)	100(7)	20(7)	41(6)	14(7)
C(35)	53(3)	59(3)	68(4)	11(3)	18(3)	5(3)
C(36)	75(4)	59(4)	83(4)	-3(3)	25(3)	4(3)
C(37)	90(5)	65(5)	140(7)	-16(5)	19(5)	0(4)
C(38)	118(7)	68(5)	158(10)	22(6)	1(7)	-21(5)
C(39)	102(6)	114(7)	130(8)	49(6)	13(6)	-41(6)
C(40)	80(5)	91(5)	98(5)	20(4)	35(4)	-11(4)
C(42)	53(3)	64(3)	60(3)	6(3)	27(3)	7(2)
C(43)	74(4)	60(3)	63(3)	6(3)	32(3)	20(3)
C(44)	72(4)	82(4)	75(4)	-3(3)	21(3)	13(3)
C(45)	58(4)	83(4)	87(5)	-3(3)	34(3)	17(3)
C(46)	75(4)	61(3)	62(3)	3(3)	30(3)	15(3)
C(48)	57(3)	63(4)	77(4)	6(3)	24(3)	6(3)
C(49)	74(4)	68(4)	73(4)	7(3)	20(3)	0(3)
C(50)	78(4)	83(5)	80(5)	20(4)	23(4)	-3(4)
C(51)	94(5)	68(5)	151(8)	22(5)	45(6)	-9(4)
C(52)	102(6)	80(5)	144(8)	-20(5)	63(6)	-7(4)
C(53)	91(5)	60(4)	104(5)	-14(4)	45(4)	-3(3)
C(54)	123(9)	138(10)	134(11)	59(8)	33(8)	-20(8)
C(55)	64(3)	59(3)	67(4)	5(3)	25(3)	10(3)
C(56)	66(4)	55(3)	90(4)	4(3)	26(3)	-1(3)
C(57)	81(5)	70(4)	118(6)	7(4)	22(5)	-5(4)
C(58)	120(7)	78(5)	97(6)	-17(4)	6(5)	0(5)
C(59)	129(7)	119(7)	78(5)	-12(5)	36(5)	4(6)
C(60)	100(5)	88(5)	66(4)	0(4)	35(4)	6(4)

C(62)	49(3)	64(3)	59(3)	-1(3)	18(2)	-3(2)
C(63)	56(3)	52(3)	65(3)	0(2)	27(3)	1(2)
C(64)	104(5)	57(4)	97(5)	1(3)	53(5)	-5(3)
C(65)	119(7)	87(5)	123(7)	-25(5)	75(6)	-48(5)
C(66)	61(3)	55(3)	67(3)	13(3)	33(3)	10(3)
C(68)	57(3)	51(3)	65(3)	6(2)	30(3)	5(2)
C(69)	54(3)	64(3)	71(4)	8(3)	31(3)	1(3)
C(70)	67(4)	70(4)	56(3)	6(3)	18(3)	1(3)
C(71)	89(5)	86(4)	66(4)	-1(3)	37(4)	0(4)
C(72)	73(4)	91(4)	67(4)	6(3)	34(3)	15(3)
C(73)	64(3)	73(4)	66(4)	2(3)	29(3)	7(3)
C(74)	97(6)	124(8)	76(5)	15(5)	17(5)	16(6)
C(75)	66(4)	67(4)	70(4)	15(3)	40(3)	8(3)
C(76)	96(5)	56(3)	102(5)	-7(3)	59(4)	-4(3)
C(77)	156(8)	66(5)	176(9)	-9(5)	117(8)	-1(5)
C(78)	198(13)	86(7)	219(13)	35(7)	165(12)	40(7)
C(79)	149(10)	127(9)	192(12)	81(8)	117(9)	78(7)
C(80)	88(5)	121(6)	102(5)	38(5)	54(4)	38(5)
C(111)	310(20)	360(20)	145(12)	135(14)	115(14)	142(19)
C(112)	156(10)	239(14)	104(7)	59(8)	-30(7)	-18(9)
C(120)	236(14)	148(10)	267(15)	-70(10)	181(13)	-32(10)
C(121)	430(30)	171(13)	400(30)	-163(16)	320(20)	-113(15)
C(201)	92(5)	136(7)	143(7)	70(6)	61(5)	60(5)
C(202)	125(8)	316(18)	175(11)	124(12)	61(8)	110(10)
C(301)	103(5)	67(4)	120(6)	-20(4)	66(5)	-12(4)
C(302)	173(9)	138(8)	144(8)	-55(7)	90(8)	-35(7)
C(401)	214(15)	182(13)	500(30)	-216(18)	183(19)	-117(12)
C(402)	246(18)	270(20)	253(18)	-32(15)	77(15)	-140(16)
C(601)	360(20)	310(20)	97(8)	72(10)	124(12)	154(17)
C(602)	280(20)	440(30)	129(11)	43(15)	118(13)	70(20)
C(801)	141(8)	175(10)	181(11)	91(9)	102(8)	25(7)
C(802)	140(13)	290(30)	91(11)	22(13)	54(10)	-1(14)
C(901)	86(5)	55(4)	146(7)	-5(4)	53(5)	-1(3)
C(902)	118(7)	124(7)	131(7)	-44(6)	23(6)	-28(6)

Table S17. Hydrogen bonds for **12h** [Å and °].

D-H...A	d(D-H)	d(H...A)	d(D...A)	<(DHA)
N(7)-H(7)...O(10)	0.86	2.01	2.845(6)	164.1
N(27)-H(27)...O(7)	0.86	1.96	2.813(5)	173.7
N(47)-H(47)...O(5)	0.86	1.96	2.811(6)	170.3
N(67)-H(67)...O(1)	0.86	1.98	2.825(5)	169.1
C(5)-H(5)...O(66)#1	0.93	2.63	3.140(7)	115.4
C(13)-H(13)...O(6)	0.93	2.44	2.910(8)	111.2
C(23)-H(23)...O(7)	0.98	2.49	3.342(7)	145.1
C(33)-H(33)...O(26)	0.93	2.30	2.888(8)	120.9
C(43)-H(43)...O(5)	0.98	2.62	3.460(7)	144.2
C(45)-H(45)...O(26)#1	0.93	2.58	3.112(7)	116.5
C(53)-H(53)...O(46)	0.93	2.38	2.898(8)	114.8
C(63)-H(63)...O(1)	0.98	2.47	3.304(7)	142.7
C(73)-H(73)...O(66)	0.93	2.28	2.876(7)	121.2
C(111)-H(11B)...F(3A)	0.97	2.50	3.05(3)	116.1
C(121)-H(12D)...F(9A)	0.96	2.49	3.37(2)	151.5
C(201)-H(20A)...O(6)#2	0.97	2.60	3.526(11)	159.1
C(201)-H(20B)...N(1)	0.97	2.57	3.149(9)	118.8
C(301)-H(30A)...O(6)#2	0.97	2.66	3.335(9)	127.2
C(601)-H(60B)...N(21)	0.97	2.53	3.167(14)	123.1
C(801)-H(80B^a)...N(41)	0.97	2.62	3.189(11)	117.8
C(802^a)-H(80C^a)...F(2)#3	0.96	2.48	3.163(17)	128.2
C(802^a)-H(80C^a)...F(1A)#3	0.96	2.44	3.21(4)	137.5
C(901)-H(90B)...O(46)#4	0.97	2.63	3.306(8)	126.7

Symmetry transformations used to generate equivalent atoms:

#1 x+1/2,-y+1/2,z #2 -x+3/2,y-1/2,-z+2 #3 -x+1,-y+1,-z+1

#4 -x+3/2,y+1/2,-z+1

I_1-IR Binding Activity

Table S18. I_2-IRs and I_1-IRs Binding Affinities (pK_i) and I_2-IRs/I_1-IRs selectivity of the most representative new compounds.

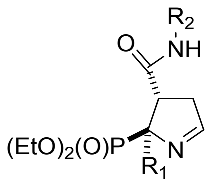

12 (a-o)

Compound	R_1	R_2	[³H]-2-BFI, I_2 one site<br>[b] I_2 one site two sites H/L; (pK_{iH}/pK_{iL})<br>High affinity site %	[³H]-Clonidine I_1 LogIC_{50}	Selectivity I_2/I_1 [a]
**12a**	H	Ph	4.64±0.47<br>8.70±1.29/3.89±0.62<br>25±12	7.84±0.24	0.0014
**12b**	H	3-Cl,4-FPh	5.61±0.21<br>8.66±0.43/5.12±0.24<br>28±5	<3	-
**12c**	Ph	Ph	3.52±0.64	7.58±0.47	0.0001
**12d**	Ph	3-Cl,4-FPh	9.98±0.74	<3	-
**12g**	Ph	4-CF3Ph	7.04±0.28	4.68±0.27	229.1
**12h**	Ph	cyclohexyl	3.57±0.21<br>8.93±0.38/3.18±0.29<br>15±4	4.31±0.05	0.182
**12l**	4-MeOPh	3-Cl,4-FPh	3.99±0.18<br>10.20±0.31/3.75±0.13<br>23±3	5.18±0.18	0.065
**12n**	Bn	Ph	8.385±0.38<br>8.99±0.37/3.04±0.33<br>32±1	<3	-

[a] Selectivity I_2-IRs/I_1-IRs expressed as the antilog (pK_i I_2-IR- pK_i /I_1-IR).). [b] The best fit of the data for **12a**, **12b**, **12h, 12l** and **12n** was to a two-site binding model of binding with high pK_i (pK_{iH}) and low pK_i (pK_{iL}) affinities for both binding sites respectively.

3D-QSAR study and physicochemical parameters

Internal and external validation of 3D-QSAR models

The internal validation of the created 3D-QSAR models was performed on training set compounds calculating parameters such as: Q^2 (Cross-validated Squared Correlation Coefficient) and RMSEE (Root Mean Square Error of Estimation):

$$Q^2 = 1 - \frac{PRESS}{\sum (Y_{obs(training)} - \overline{Y}_{training})^2} \quad (1)$$

In the above equation, $Y_{obs(training)}$ denote the experimental p*K*i values of the compounds in the training set, $\overline{Y}_{training}$ is the mean value of the dependent variable of the compounds in the training set, and PRESS is a parameter calculated using the leave-one-out (LOO) approach, in which each compound was deleted once from the training set and a new model was created with the remaining compounds and used to predict the Y value of the deleted compound. The procedure was repeated until all compounds have been deleted once. For all models created, the squared sum of the differences between the observed and LOO predicted values (e(i)) (PRESS) was calculated according to Eq.(2):

$$PRESS = \sum_{i=1}^{n} e_{(i)}^2 \quad (2)$$

The models with $Q^2 \geq 0.5$ can be considered to have good predictive capability.[1, 2]

The Root Mean Squared Error of Estimation (RMSEE) was calculated to characterize the predictive ability of the models for the compounds in the training set:

$$RMSEE = \sqrt{\frac{\sum_{i=1}^{n} (Y_{obs(training)} - Y_{pred(training)})^2}{n}} \quad (3)$$

where n is the number of compounds in the training set, while $Y_{obs(training)}$ and $Y_{pred(training)}$ denote the experimental and predicted values for the compounds in the training set.

The external validation of 3D-QSAR models is evaluated using test set compounds calculating the parameters R^2_{pred} and RMSEP (Root Mean Square Error of Prediction) according to Eq. 4 and 5:

$$R^2_{pred} = 1 - \frac{\sum (Y_{pred(test)} - Y_{obs(test)})}{\sum (Y_{obs(test)} - \overline{Y}_{training})^2} \quad (4)$$

where $Y_{pred(test)}$ and $Y_{obs(test)}$ are the predicted and observed values of the dependent variables of the compounds in the test set, respectively, and $\overline{Y}_{training}$ indicates the mean value of the dependent

variables of the compounds in the training set. For a predictive QSAR model the R^2_{pred} value should be greater than 0.5.[3]

$$RMSEP = \sqrt{\frac{\sum_{i=1}^{n} (Y_{obs(test)} - Y_{pred(test)})^2}{n}} \quad (5)$$

where n is the number of compounds in the test set, while $Y_{obs(test)}$ and $Y_{pred(test)}$ are the experimental and predicted values for the compounds in the test set.

Table S19. Results of developed 3D-QSAR (I_2-IRs) model.

**Training set**			**Test set**		
	**p*K*$_i$ (exp)**	**p*K*$_i$ (pred)**		**p*K*$_i$ (exp)**	**p*K*$_i$ (pred)**
**12k**	3.070	3.239	**12l**	3.990	4.259
**12c**	3.520	3.824	**12i**	5.030	6.345
**12h**	3.570	3.291	**12b**	5.610	5.656
**12j**	3.710	4.019	**12g**	7.040	7.162
**12a**	4.640	4.669	**BU99008**	7.050	7.742
**12o**	5.220	4.991			
**1, CR4056**	5.950	5.930	**R^2_{pred} = 0.671 (>0.5)**		
**12f**	6.010	5.730	**RMSEP= 0.678**		
**4, Idazoxan**	7.410	7.131			
**12n**	8.385	8.162			
**9, B06**	8.560	8.838			
**12d**	9.980	10.071			
**R^2 = 0.989 (>0.7)**					
**Q^2_{LOO} = 0.66 (>0.5)**					
**RMSEE= 0.231**					

Table S20. Results of developed 3D-QSAR (α_2-ARs) model.

**Training set**			**Test set**		
	**p*K*$_i$ (exp)**	**p*K*$_i$ (pred)**		**p*K*$_i$ (exp)**	**p*K*$_i$ (pred)**
**1, CR4056**	2.650	1.978	**12n**	3.897	5.532
**12l**	3.240	3.011	**12f**	4.670	3.857
**12j**	3.560	3.783	**12c**	6.550	5.452
**12h**	3.600	4.664	**12m**	7.270	6.979
**2, BU99008**	4.370	4.748	**12b**	7.410	7.404
**12g**	5.330	5.681	**R^2_{pred} = 0.542 (>0.5)**		
**12a**	6.000	6.677	**RMSEP=0.962**		
**B06**	6.270	6.707			
**12o**	7.970	7.199			
**4, Idazoxan**	8.350	7.403			
**12d**	9.430	9.209			
**R^2 = 0.921 (>0.7)**					
**Q^2_{LOO} = 0.61 (>0.5)**					
**RMSEE= 0.614**					

The applicability domain (AD). Each 3D-QSAR model has a specific applicability domain within which its activity predictions remain accurate. To determine this range, the leverage approach is applied by creating a William plot using SPSS v.18.0 software for the analysis.[4] The critical leverage threshold (h*), represented by a vertical line, is calculated using the following formula (Eq. 6):

$h^* = 3(p+1)/n$ (**6**)

In this equation, 'n' denotes the number of compounds in the training set and 'p' refers to the GRIND variables used to define the applicability domain (AD). If the absolute standardized residual of a molecule exceeds three standard deviations or if the leverage value exceeds the critical leverage value (h*), the molecule falls outside the AD and makes the prediction of the model unreliable for that compound. As shown in **Figure S2**, all compounds are within the chemical space defined by the AD for both 3D-QSAR models.

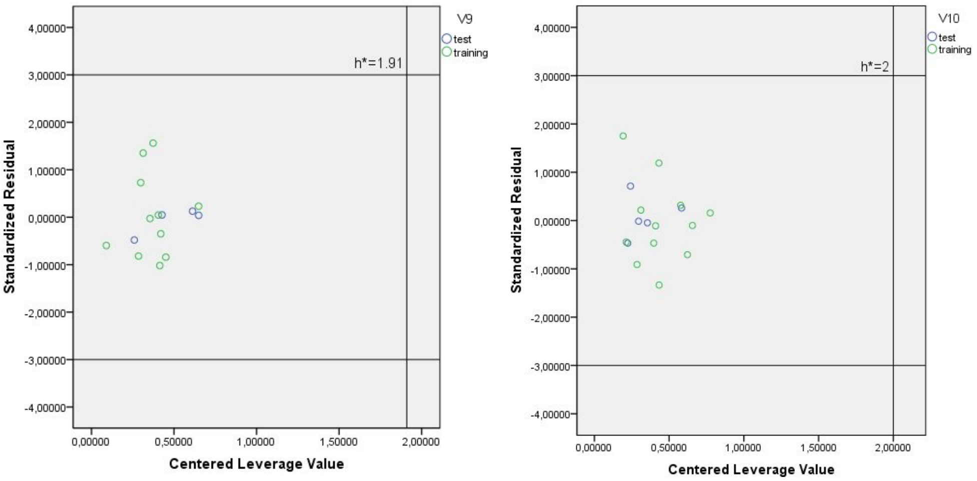

Figure S2. Applicability domain of developed 3D-QSAR (α_2–AR) model (left) and 3D-QSAR (I_2–IR) model (right).

Table S21. Results of physico-chemical parameters of I_2-IRs ligands obtained from SwissADME web tool.[5]

Compound	pKi (I_2)	MW <500 (g/mol)	Log S (ESOL) -10 to 0	Solubility class	Log Po/w (XLOGP3) < 5	Log Po/w (MLOGP) < 4.15	Log Po/w (WLOGP) < 5.6	Consensus Log Po/w	RuleOf5	TPSA 20Å^2<TPSA<130 Å^2
**Idazoxan**	7.41	204.23	-1.78	Very soluble	0.70	0.99	0.07	1.23	Yes; 0 violation	42.85 Å^2
**CR4056**	5.95	272.3	-4.13	Moderately soluble	3.17	2.28	3.48	2.90	Yes; 0 violation	43.60 Å^2
**BU99008**	7.05	199.25	-2.27	Soluble	1.29	1.95	0.77	1.75	Yes; 0 violation	29.32 Å^2
**B06**	8.56	478.84	-4.19	Moderately soluble	2.48	2.99	4.34	3.43	Yes; 0 violation	95.08 Å^2
**12a**	4.64	324.31	-1.71	Very soluble	0.3	0.8	2.74	1.62	Yes; 0 violation	86.80 Å^2
**12b**	5.61	376.75	-2.48	Soluble	1.02	1.7	3.95	2.45	Yes; 0 violation	86.80 Å^2
**12c**	3.52	400.41	-3.17	Soluble	1.78	1.98	4.15	2.94	Yes; 0 violation	86.80 Å^2
**12d**	9.98	452.84	-3.93	Soluble	2.51	2.84	5.37	3.79	Yes; 0 violation	86.80 Å^2
**12e**	<3	434.85	-3.77	Soluble	2.41	2.46	4.81	3.52	Yes; 0 violation	86.80 Å^2
**12f**	6.01	476.5	-4.67	Moderately soluble	3.41	3.02	5.82	4.29	Yes; 0 violation	86.80 Å^2
**12g**	7.04	468.41	-4.04	Moderately soluble	2.66	2.78	6.33	3.99	Yes; 0 violation	86.80 Å^2
**12h**	3.57	406.46	-3.2	Soluble	2.03	1.94	4.16	3.05	Yes; 0 violation	86.80 Å^2
**12i**	5.03	418.4	-3.33	Soluble	1.88	2.35	4.71	3.27	Yes; 0 violation	86.80 Å^2
**12j**	3.71	470.83	-4.10	Moderately soluble	2.61	3.21	5.93	4.09	Yes; 0 violation	86.80 Å^2
**12k**	3.07	430.43	-3.25	Soluble	1.75	1.66	4.16	2.92	Yes; 0 violation	96.03 Å^2
**12l**	3.99	482.87	-4.01	Moderately soluble	2.48	2.52	5.38	3.77	Yes; 0 violation	96.03 Å^2
**12m**	<3	338.34	-1.91	Very soluble	0.48	1.05	3.13	2.01	Yes; 0 violation	86.80 Å^2
**12n**	8.38	414.43	-3.36	Soluble	2.07	2.19	4.35	3.13	Yes; 0 violation	86.80 Å^2
**12o**	5.22	420.48	-3.41	Soluble	2.33	2.16	4.35	3.33	Yes; 0 violation	86.80 Å^2

Legend: MW – molecular weight; **Log S (ESOL)** – water solubility, topological method implemented from Delaney JS 2004 J. Chem. Inf. Model; **Solubility Class** - insoluble<-10 < poorly <-6 <moderately<-4 < soluble<-2 < very < 0 < highly; **XLOGP3** – atomistic and knowledge-based method calculated by XLOGP program; **MLOGP** – topological method implemented from Moriguchi I. et al. 1992 Chem. Pharm. Bull.; **WLOGP** – atomistic method implemented from Wildman SA and Crippen GM. 1999 J. Chem. Inf. Model.; **Consensus Log Po/w** – average logP value of five predictions; **RuleOf5** - Lipinski's "Rule of Five"; **TPSA** – topological polar surface area.

Table S22. Results of pharmacokinetic parameters of I2-IR ligands obtained with ADMET Predictor software.[6]

Compound	pKi(I_2)	S+Peff	BBB filter	hum_puf%	Pgp_Substrat	Pgp_Inh	CYP_risk	Tox_Risk	hERG_ Filter
**Idazoxan**	7.41	3.45234	High (84%)	69.5129	No (79%)	No (93%)	2	2	Yes (45%)
**CR4056**	5.95	5.13308	High (99%)	3.96437	No (94%)	No (76%)	0.651104	2	No (61%)
**BU99008**	7.05	3.14552	High (87%)	58.9229	No (85%)	No (93%)	1.55779	3.15212	No (61%)
**B06**	8.56	3.96564	High (99%)	6.54671	No (94%)	Yes (79%)	1	0.5	Yes (69%)
**12a**	4.64	2.24027	High (99%)	28.6657	No (79%)	Yes (86%)	1	0.5	Yes (57%)
**12b**	5.61	3.28897	High (99%)	15.8575	No (85%)	Yes (77%)	1	1.5	Yes (64%)
**12c**	3.52	3.13241	High (99%)	8.45195	No (79%)	Yes (90%)	1.14514	0	Yes (79%)
**12d**	9.98	3.71647	High (99%)	5.49241	No (94%)	Yes (90%)	1.81859	0	Yes (86%)
**12e**	<3	3.61849	High (96%)	6.472	No (85%)	Yes (90%)	1.92425	0.0175094	Yes (86%)
**12f**	6.01	2.70986	High (96%)	3.15081	No (69%)	Yes (98%)	2	1	Yes (86%)
**12g**	7.04	3.53687	High (96%)	5.89359	No (94%)	Yes (90%)	2.08948	1	Yes (79%)
**12h**	3.57	2.53425	High (99%)	9.15945	No (85%)	Yes (98%)	2.14992	0	Yes (72%)
**12i**	5.03	3.35637	High (99%)	7.70125	No (79%)	Yes (90%)	2.48629	0	Yes (82%)
**12j**	3.71	4.30569	High (96%)	5.33004	No (94%)	Yes (90%)	1.1582	1.50399	Yes (86%)
**12k**	3.07	2.74557	High (99%)	8.79688	No (79%)	Yes (90%)	1.43917	0	Yes (69%)
**12l**	3.99	3.17049	High (93%)	5.62294	No (85%)	Yes (90%)	2.43353	0.716675	Yes (69%)
**12m**	<3	2.41947	High (99%)	21.2166	No (79%)	Yes (86%)	1	0.565654	Yes (59%)
**12n**	8.38	3.11064	High (99%)	7.32456	No (79%)	Yes (90%)	1.9429	0	Yes (82%)
**12o**	5.22	2.59276	High (99%)	7.92465	No (85%)	Yes (98%)	2.72276	0	Yes (75%)

Legend: S+Peff – estimated permeability, BBB filter - qualitative likelihood (High/Low) of crossing the blood brain barrier; hum_fup% - percentage of unbound drug in plasma; Pgp_Substr - likelihood of P-glycoprotein efflux; Pgp_Inh – likelihood of a molecule being an inhibitor of P-glycoprotein; CYP_Risk – cytochrome P450 liability score; Tox_Risk – toxicity liability score; hERG_Filter - qualitative estimation of the likelihood of the hERG potassium channel inhibition in human.

In vitro Blood-Brain Barrier Permeation Assay

To evaluate the brain penetration of the different compounds, a parallel artificial membrane permeation assay for blood-brain barrier was used, following the method described by Di et al.[7] The *in vitro* permeability (P_e) of fourteen commercial drugs through the lipid extract of porcine brain membrane together with the test compounds were determined. Commercial drugs and assayed compounds were tested using a mixture of PBS:EtOH (70:30). Assay validation was made by comparing the experimental permeability with the reported values of the commercial drugs by a bibliography and linear correlation between experimental and reported permeability of the fourteen commercial drugs using the parallel artificial membrane permeation assay was evaluated (Table S23). From this equation and taking into account the limits established by Di et al. for BBB permeation, we established the ranges of permeability as compounds of high BBB permeation (CNS+): Pe (10^{-6} cms^{-1}) > 5.198; compounds of low BBB permeation(CNS-): Pe (10^{-6} cm s^{-1}) < 2.054 and compounds of uncertain BBB permeation (CNS+/-): 5.198 > Pe (10^{-6} cm s^{-1}) > 2.054. Results of the BBB-permeation for new compounds are in Table S24.

Table S23. Permeability (*Pe* 10^{-6} cm s^{-1}) in the PAMPA-BBB assay of the 14 commercial drugs predictive penetration in the CNS used as references.

**Compound**	**Bibliography value[(a)]**	**Experimental value (n=3) ± S.D.**	***CNS Prediction***
Verapamil	16.0	25.4±0.6	CNS+
Testosterone	17.0	27.1±0.5	CNS+
Costicosterone	5.1	6.7±0.1	CNS+
Clonidine	5.3	6.5±0.05	CNS+
Ofloxacin	0.8	0.1±0.08	CNS-
Lomefloxacin	0.0	0.85±0.03	CNS-
Progesterone	9.3	16.8±0.3	CNS+
Promazine	8.8	13.8±0.3	CNS+
Imipramine	13.0	12.5±0.2	CNS+
Hidrocortisone	1.9	1.4±0.05	CNS-
Piroxicam	2.5	1.9±0.07	CNS-
Desipramine	12.0	17.8±0.1	CNS+
Cimetidine	0.0	0.7±0.03	CNS-
Norfloxacine	0.1	8.8±0.5	CNS+

Table S24. Permeability results (Pe 10^{-6} cm s^{-1}) from the PAMPA-BBB assay for new report compounds and their prediction of BBB permeation.

Compound	$^{a}P_{e}$ 10^{-6} cm s^{-1}	Prediction
**12a**	7,95±0,2	CNS+
**12b**	5,3±0,2	CNS+/-
**12c**	12,5±0,3	CNS+
**12d**	20,4±1,2	CNS+
**12e**	12,2±0,3	CNS+
**12f**	11,5±0,3	CNS+
**12g**	10,1±0,6	CNS+
**12h**	19,5±1,25	CNS+
**12i**	10,8±0,6	CNS+
**12j**	17,0±0,7	CNS+
**12k**	4,1,0±0,3	CNS+/-
**12l**	16,3±0,9	CNS+
**12m**	2,9±0,2	CNS+/-
**12o**	>30	CNS+

[a] PBS/EtOH (70:30) was used as solvent. Values are expressed as mean±SD of at least three independent experiments.

Cytotoxicity assays

All tumor cell lines and the normal human embryonal lung fibroblast cell line MRC-5 were acquired from the American Type Culture Collection (ATCC, Manassas, VA, USA), except for the DND-41 cell line, which was purchased from the Deutsche Sammlung von Mikroorganismen und Zellkulturen (DSMZ Leibniz-Institut, Germany), and the Hap-1 cell line which was ordered from Horizon Discovery (Horizon Discovery Group, UK). All cell lines were cultured as recommended by the suppliers. Media were purchased from Gibco Life Technologies, USA, and supplemented with 10 % fetal bovine serum (HyClone, GE Healthcare Life Sciences, USA). Adherent cell lines HCT-116, NCI-H460, LN-229, Hap-1 and Capan-1 cells were seeded at a density between 500 and 1500 cells per well, in 384-well, black walled, clear-bottomed tissue culture plates (Greiner). After overnight incubation, cells were treated with the test compounds at seven different concentrations ranging from 100 to 6.4 x 10^{-3} µM. Suspension cell lines HL-60, K-562, Z-138, and DND-41 were seeded at densities ranging from 2500 to 5500 cells per well in 384-well, black walled, clear-bottomed tissue culture plates containing the test compounds at the same seven concentration points. The plates were incubated and monitored at 37 °C for 72 h in an IncuCyte® (Essen BioScience Inc., Ann Arbor, MI, USA) for real-time imaging. Images were taken every 3 h, with one field imaged per well under 10x magnification.

Solubility

The stock solutions (10^{-2} M) of the assayed compound were diluted to decreased molarity, from 300 µM to 0.1µM, in 384 well transparent plate (*Greiner* 781801) with 1% DMSO: 99% PBS buffer. Incubated at 37ºC and read after 2 hours in a *NEPHELOstar Plus* (*BMG LABTECH*).

The results were adjusted to a segmented regression to obtain the maximum concentration in which compound is soluble. **12d,** solubility >100 µM.

Chemical stability assay at different pHs

Chemical stability assay may be used to identify:

- Highly unstable compounds: Compounds which are highly unstable are not always suitable as drug candidates as it may be difficult to maintain a therapeutically effective formulation.

- Compounds which are not stable at low pHs: For oral administration compounds must be chemically stable at pHs like those found in the stomach.

- Compounds which are unstable at pH 7.4: Subsequent analysis of *in vivo* samples following administration of such compounds may be very difficult. The data from *in vitro* screens may also be unreliable.

Protocol overview:[8]

Test compound solution (1 µM, 0.1% final DMSO concentration) is incubated with buffer pH 2, 5 and 7.4 at 37ºC. Serial samples are taken at 0, 5, 15, 30, 60 and 120 min. All samples are added immediately to 3 volumes of methanol containing internal standard in a microtiter plate cooled to halt chemical degradation. All the samples are analyzed by LC-MS/MS. The percentage of parent compound remaining at each time point relative to the 0 min sample is calculated from peak area ratios.
The chemical stability assay returns the percent parent compound remaining at each time point for **12d**.

LC/MS Conditions:

The detection of **12d** were obtained by LC/MS/MS spectrometry. The autosampler's drawers were kept cooled at 4 °C.

Detection of analytes and internal standards was carried out in multiple reaction monitoring mode (MRM) with electrospray positive ionization. Mass transitions used in the present method are summarized below.

- m/z **12d**:453.4/315.1; 453.4/144.1 Collision energy: 20 V
- m/z Internal standard: diethyl ((1*R*,3a*S*,6a*S*)-4,6-dioxo-1,5-diphenyl-1,3a,4,5,6,6a-hexahydropyrrolo[3,4-*c*]pyrrol-1-yl)phosphonate: 427.2/289.3; Collision energy: 21 V

During analysis, the following mobile phase is used: A is 0.1% formic acid in water/acetonitrile 90/10 and B is 0.1% formic acid in acetonitrile/water. 90/10. The injection volume was 5 µL.

Results

The result obtained are showed below in different graphics and tables for pH 2, 5 and 7.4

pH 2

Table S25. % Compound remaining at pH 2.

Time	12d % Compound remaining
0	100±12.89
5	96±13.56
15	93±12.99
30	102±8.68
60	105±15.42
120	96±13.2

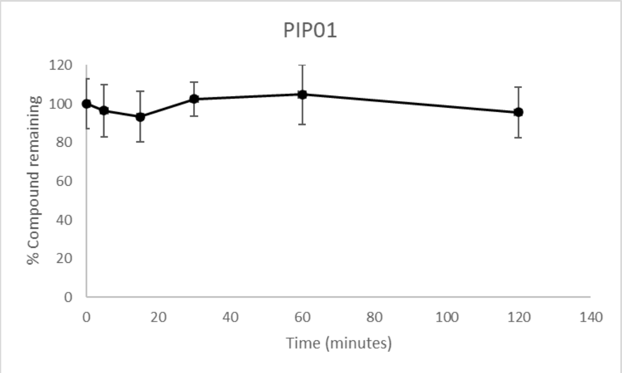

Figure S3. Chemical stability at pH 2 of **12d** (PIP01).

pH 5

Table S26. % Compound remaining at pH 5.

Time	12d % Compound remaining
0	100±8.9
5	85±7.5
15	125±5.3
30	103±19.0
60	98±1.9
120	127±10.5

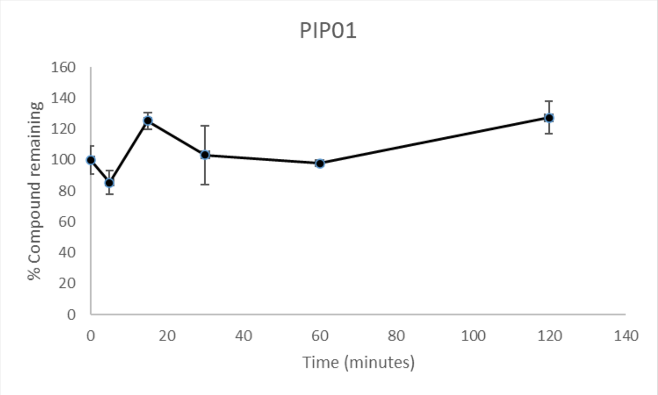

Figure S4. Chemical stability at pH 5 of **12d** (PIP01).

pH 7.4

Table S27. % Compound remaining at pH 7.4

Time	12d % Compound remaining
0	100±15.4
5	109±16.0
15	123±2.9
30	97±5.4
60	118±8.8
120	118±23.5

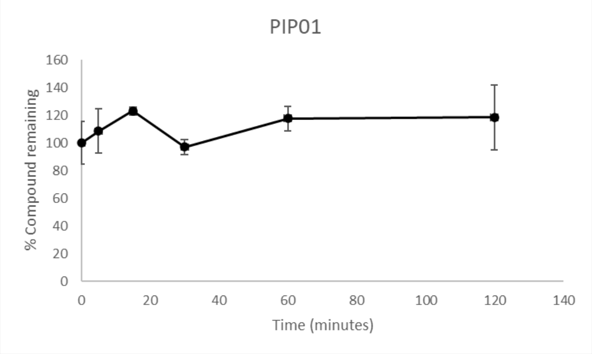

Figure S5. Chemical stability at pH 7.4 of **12d** (PIP01).

Remarks:

The **12d** compound was stable for 2 hours at pH 2, pH 5 and pH 7.

Caco-2 permeability assay

The Caco-2 cells are cultured to confluency, trypsinized, and seeded onto a filter transwell insert at a density of ~10,000 cells/well in DMEM cell culture medium. Confluent Caco-2 cells were subcultured at passages 58-62 and grown in a humidified atmosphere of 5% CO_2 at 37 °C. Following an overnight attachment period (24 h after seeding), the cell medium is replaced with fresh medium in both the apical and basolateral compartments every other day. The cell monolayers are used for transport studies 21 days post-seeding.

The monolayer integrity was checked by measuring the transepithelial electrical resistance (TEER) obtaining values ≥ 500 Ω/cm^2.

On the day of the study, after the TEER measurement, the medium was removed, and the cells were washed twice with pre-warmed (37°C) Hank's Balanced Salt Solution (HBSS) buffer to remove traces of medium.

Stock solutions were made in dimethyl sulfoxide (DMSO), and further diluted in HBSS (final DMSO concentration 1%). Each compound and reference compound (Colchicine, E3S) were all tested at a final concentration of 10µM.

For A → B directional transport, the donor working solution is added to the apical (A) compartment and the transport media as receiver working solution is added to the basolateral (B) compartment. For B → A directional transport, the donor working is added to the basolateral (B) compartment and transport media as receiver working solution is added to the apical (A) compartment.

The cells were incubated at 37 °C for 120 minutes with gentle stirring.

At the end of the incubation, samples were taken from both donor and receiver compartments and transferred into 384- well plates and analyzed by UPLC-MS/MS. The detection was performed using an ACQUITY UPLC /Xevo TQD *System.*

After the assay, Lucifer yellow was used to further validate the cell monolayer integrity, cells were incubated with LY 10µM in HBSS for 1hour at 37ºC, obtaining permeability (Papp) values for LY of ≤ 10 nm/s confirming the well-established Caco-2 monolayer.

Chromatographic conditions:

- Column: Acquity UPLC HSS T3 1.7µm (2.1mm x 50mm)
- Gradient: A (H_2O + 0.1% Formic acid); B (ACN + 0.1% Formic acid)
- Flow: 0.6mL/min

t(min)	A (%)	B (%)
0	95	5
0,1	95	5
1	0	100
2	0	100
2,1	95	5
3	95	5

Data Analysis:

Papp = (dQ/dt) × (1/C_0) × (1/A)

Efflux ratio (ER) = Papp [B → A] / Papp [A → B]

%Recovery = (Total compound mass in donor and receiver compartments at the end of the incubation / Initial compound mass in the donor compartment) x 100.

Where dQ/dt is the permeability rate, C_0 is the initial concentration in the donor compartment, and A is the surface area of the cell monolayer (0.146 cm^2). The Papp value is a rate measured in nm/s.

Results

Table S28. Parameters obtained for **12d** in the Caco-2 permeability assay.

	**Recovery (%)**	**Recovery (%)**	**Papp (nm/s)**	**Papp (nm/s)**	**Efflux ratio**
**12d**	AB 91,6 ± 1,2	BA 105,8 ± 1,5	AB 158,0 ± 0,5	BA 260,2 ± 36,6	1,6 ± 0,6
**Colchicine**	AB 109,3 ± 4,8	BA 92,1 ± 5,6	AB 3,9 ± 0,2	BA 67,9 ± 4,6	17,5 ± 1,3
**E3S**	AB 86,1 ± 4,8	BA 80,5 ± 6,8	AB 4,8 ± 0,4	BA 53,9 ± 8,4	11,2 ± 2,1

Microsomal stability at human and mice microsomes

Human and mice microsomes from Tebu-Xenotech were employed in the assay. They content 20 mg/mL of protein.

The following quantities were added to each well of a 96-well microplate.

	Blank (µl)	Rat (µl)	Mice (µl)	Human (µl)
Phosphate buffer Na/K 50 mM pH 7.4	295	301.3	301.3	301.3
MgCl2 30 mM	50	50	50	50
NADP 10 mM	50	50	50	50
Glucose 6-P 100mM	50	50	50	50
Glucose 6-P DH 20 U/ml	25	25	25	25
Water	25			
Rat microsomes		18.7		
Mice microsomes			18.7	
Human microsomes				18.7
Test compound	5	5	5	5

Plates were incubated at 37 °C and 75 µL samples were taken at 0, 10, 20, 40, and 60 min. Samples were transferred to a microplate, and 75 µL Acetonitrile was added to inactivate the microsomes, and 30 µL of H_2O was added to improve the chromatographic conditions and kept at 4 °C. When all the samples were taken the plate was centrifuged at 46000 g for 30 min at 15 °C. The supernatant was taken and injected in the UPLC-MS/MS. Stationary phase: Reverse phase Acquity UPLC® BEH C18 1.7 µm (2.1 mm x 50 mm) (Waters). Mobile phase: A: H_20 + 0.1 % formic; B: acetonitrile + 0.1 % formic acid.

Gradient:

Time	Water	Acetonitrile
0	95%	5%
0.1	95%	5%
1	0%	100%
2	0%	100%
2.1	95%	5%
2.5	95%	5%

Flow: 0.6 mL/min. The chromatographic equipment employed was an UPLC QSM Waters Acquity. Compound concentrations were calculated from the UV peak areas. The response was linear in the range between 10 ng/mL and 0.3125 ng/mL. Metabolic stability was calculated from the logarithm of the remaining **12d** at each of the times evaluated.

Table S29. Microsomal stability of **12d** at mice, human, and rat microsomes**.**

**Mice**			**Human**			**Rat**		
**% remanent** (sampling time 60 min)	$t_{1/2}$ (min)	**Clint** (µL/min* mg prot)	**% remanent** (sampling time 60 min)	$t_{1/2}$ (min)	**Clint** (µL/min* mg prot)	**% remanent** (sampling time 60 min)	$t_{1/2}$ (min)	**Clint** (µL/min* mg prot)
41.21	40.95	18.93	25.98	31.24	15.20	30.91	38.07	15.17

Plasma stability

Human and mouse plasma pooled from healthy donors extracted in citrate tubes was employed in the assay.

Plates containing 10 µM compounds in plasma (total volume: 50 µL) were incubated at 37 ºC at different times (0, 60, 180 and 360 min). Then 100 µl Acetonitrile was added for precipitating plasma protein, and the plate was centrifuged at 46000 g for 60 min at 5 ºC. The supernatant was taken and analyzed by UPLC/MS/MS for sample quantification. Stationary phase: Reverse phase Acquity UPLC® BEH C18 1.7 µm (2.1 mm x 50 mm) (Waters). Mobile phase: 0.1 % formic acid in H_20/0.1 % formic acid in acetonitrile.

Gradient:

Time	Water	Acetonitrile
0	95%	5%
0.1	95%	5%
1	0%	100%
2	0%	100%
2.1	95%	5%
2.5	95%	5%

Flow: 0.6 mL/min. The chromatographic equipment employed was an UPCL QSM Waters Acquity. Compound concentrations were calculated from the MS peak areas.

Table S30. Remaining percentage for **12d** at rat, human and mouse plasma at the different times.

	**0 min**	**60 min**	**120 min**	**360 min**
**Rat**	100.0	100.0	100.0	100.0
**Human**	100.0	100.0	100.0	100.0
**Mouse**	100.0	100.0	100.0	93.3

Human and mouse plasma protein binding

Human and mouse plasma from Seralab was employed in the assay which was carried out by employing Rapid Equilibrium Dyalisis (RED) from Thermo Scientific. The compounds were dissolved at 5 µM in plasma and added to the corresponding insert of the RED device. Dialysis buffer was added to the corresponding insert of the RED device. The plate was incubated for 4 h at 37 ºC. After the incubation period 50 µL aliquots of each chamber were transferred to empty vials. 50 µL of dialysis buffer was added to the plasma samples and 50 µL of plasma was added to the buffer samples. 300 µL of acetonitrile were added to all the samples and centrifuged at 4000 rpm. 100 µL aliquots of the supernatants were transferred to a LC analysis plate and diluted with 100 µL of water. Samples were analyzed in a UPLC/MS/MS. Stationary phase: Reverse phase Acquity UPLC® BEH C18 1.7 µm (2.1 mm x 100 mm) (Waters). Mobile phase: 125 mM Ammonium hydroxide/acetonitrile.

Gradient:

Time	Ammonium hydroxide	Acetonitrile
0	95%	5%
0.1	95%	5%
1	0%	100%
2	0%	100%
2.1	95%	5%
2.5	95%	5%

Flow: 0.6 mL/min. The chromatographic equipment employed was an UPLC QSM Waters Acquity. Compound concentrations were calculated from the MS peak areas.

Table S31. Rat, human and mouse plasma protein binding of compound **12d**.

**Compound**	**Species**	**Plasma protein binding (%)**	**Unbound fraction (%)**
**12d**	Rat	83.5	16.5
**12d**	Human	74.7	25.3
**12d**	Mouse	82.6	17.4
**phenytoin**	Rat	80.8	19.2
**phenytoin**	Human	79.0	21.0
**phenytoin**	Mouse	73.4	26.6

Cytochromes inhibition

Method:

The objective of this study was to screen the inhibition potential of the compounds using recombinant human cytochrome P450 enzymes (CYP1A2, CYP2C9, CYP2C19, CYP3A4 (BFC) and CYP3A4 (DBF)) and probe substrates with fluorescent detection.

Incubations were conducted in a 200 µl volume in 96 well microtiter plates (COSTAR 3915). Addition of cofactor-buffer mixture (KH2PO4 buffer, 1.3mM NADP+, 3.3mM $MgCl_2$, 3.3 mM Glucose-6-phosphate and 0.4U/mL Glucose-6-phosphate Dehydrogenase), supersomes control, standard inhibitors (Furafyline, Tranylzypromine, Ketoconazole, Sulfaphenazole and Quinidine; from Sigma Aldrich) previously diluted and compounds to plates were carried out by a liquid handling station (Zephyr Caliper). The plate was then pre-incubated at 37ºC for 5 min, and the reaction initiated by the addition of pre-warmed enzyme/substrate (E/S) mix. The E/S mix contained buffer (KH2PO4), c-DNA-expressed P450 in insect cell microsomes, substrate (3-cyano-7-ethoxycoumarin (CEC) for CYP1A2 and CYP2C19, 7-Methoxy-4-(trifluoromethyl)coumarin (7-MFC) for CYP2C9, 7-benzyloxytrifluoromethyl coumarin (7-BFC) and Dibenzylfluorescein (DBF) for CYP3A4) and other components to give the final assay concentrations in a reaction volume of 200 µl. Reactions were terminated after various times (a specific time for each cytochrome) by addition of STOP solution (ACN/TrisHCl 0.5M 80:20, and NaOH 2N for CYP3A4 (DBF).

Fluorescence per well was measured using a fluorescence plate reader (Tecan M1000 pro) and percentage of inhibition was calculated.

Results:

The table shows the data obtained.

Table S32: Results of inhibition of compound **12d**.

CYP1A2	CYP2C9	CYP2C19	CYP2C19	CYP3A4 (BFC)	CYP3A4 (BFC)	CYP3A4 (DBF)
%inhib 10µM	%inhib 10µM	%inhib 10µM	IC_{50} (µM)	%inhib 10µM	IC_{50} (µM)	%inhib 10µM
1 ± 1	35 ± 6	85 ± 1	1.27	64 ± 1	1.25	49 ± 1

Table S33: Inhibition percent values obtained and inhibitor potency values (IC_{50}) described in the literature for the compounds used to validate the assay.

Cytochrome	Compound	% inhibition obtained	IC_{50} (µM) described	Bibliographic Reference
CYP1A2	Furafylline	74 (2µM)	5.2	G. Gudi et al. Int J Pharm Pharm Sci. Vol 5, Issue 2, 303-307. 2013
CYP2C9	Sulfaphenazole	32 (0.366µM)	0.64	G. Gudi et al. Int J Pharm Pharm Sci. Vol 5, Issue 2, 303-307. 2013
CYP2C19	Tranylzypromine	74 (3.3µM)	3.2	MT. Donato et al. Drug Metabolism and Disposition. Vol 32, Nº 7; 699-706, 2004
CYP3A4 (BFC)	Ketoconazole	41 (0.027µM)	0.01	G. Gudi et al. Int J Pharm Pharm Sci. Vol 5, Issue 2, 303-307. 2013
CYP3A4 (DBF)	Ketoconazole	55 (0.008µM)	0.002	Corning Gentest

Method:

The objective of this study was to screen the compounds' inhibition potential using the recombinant human cytochrome P450 enzyme CYP2D6 and probe substrate with fluorescent detection.

Incubations were conducted in a 200 µl volume in 96 well microtiter plates (COSTAR 3915). Addition of cofactor-buffer mixture (KH2PO4 buffer, 8.2µM NADP+, 0.41 mM MgCl2, 0.41mM Glucose-6-phosphate and 0.4U/mL Glucose-6-phosphate Dehydrogenase, supersomes control, standard inhibitor Quinidine from Sigma Aldrich previously diluted and compound to plate were carried out by a liquid handling station (Zephyr Caliper). The plate was then pre-incubated at 37ºC for 5 min, and the reaction initiated by the addition of pre-warmed enzyme/substrate (E/S) mix. The E/S mix contained buffer, c-DNA-expressed P450 in insect cell microsomes, substrate 3-[2-(N,N-Diethyl-N-methylammonium)ethyl]-7-Methoxy-4-Methylcoumarin (AMMC) for CYP2D6 and other components to give the final assay concentrations in a reaction volume of 200 µl. Reaction was terminated after 30 min at 37 ºC by addition of STOP solution (ACN/TrisHCl 0.5M 80:20).

Fluorescence per well was measured using a fluorescence plate reader (Tecan M1000 pro) and percentage of inhibition was calculated.

Results:

% inhibition at 10µM is 10 ± 2.

Table S34: Inhibition percent value obtained, and inhibitor potency value (IC_{50}) described in the literature for the compound used to validate the assay.

Cytochrome	Compund	% inhibition obtained	IC_{50} (µM) described	Bibliographic Reference
CYP2D6	Quinidine	56 (0.0073µM)	0.004	G.Gudi et al. Int J Pharm Pharm Sci. Vol 5, Issue 2, 303-307.2013

hERG ion channel inhibition

The assay was carried out at a CHO cell line transfected with the hERG potassium channel. 72 h before the assay, 2500 cells were seeded on a 384 well black plate (Greiner 781091). Cell line were maintained at 37 ºC in a 5 % CO_2 atmosphere for 24 h and at 30 ºC in a 5 % CO_2 atmosphere for 48 h plus. hERG activity was measured by using the FluxorTM Potassium Ion Chanel Assay Kit (Thermo Fisher F10016). Medium was replaced for 20 µl Loading Buffer and the cells were incubated for 60 min at 25 ^{o}C, protected from direct light. After incubation, Loading Buffer was replaced for Assay buffer and the compounds were incubated for 30 min at 25 ^{o}C. 5µl of Stimulus Buffer was added to each well and the fluorescence was read (λex = 490 nm, λem = 525nm) using imaging plate reader system (FDSS7000EX, Hamamatsu®) every second after the establishment of a baseline line.

Table S35: % inhibition of hERG ion channel of **12d** at 10 µM.

Compound	% inhibition (10 µM)
**12d**	22.5±0.7

In vitro effects of 12d in a preclinical model of neurodegeneration and neuroinflammation

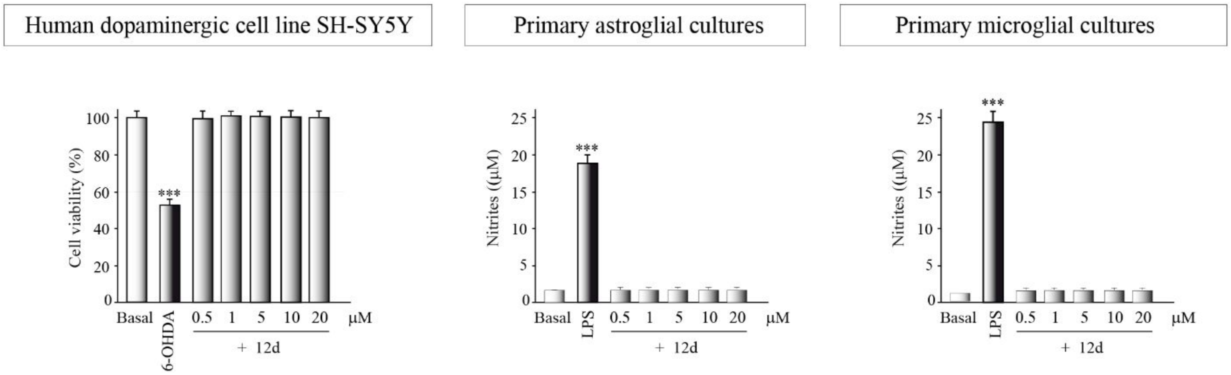

Figure S6: *In vitro* analysis of the cytotoxicity of compound **12d**. SH-SY5Y cell line and astrocytes and microglial cells isolated from murine cortex were exposed to escalating concentrations of the **12d** compound for 18h (SH-SY5Y cultures) and 24 h (glial cells). As a damage control, the neurotoxic agent 6-hydroxydopamine (6-OHDA, 35µM) was used for SH-SY5Y cells, and bacterial lipopolysaccharide (LPS, 10 µg/mL) as an inflammation inducer was used with primary glial cultures. The 3-(4,5-dimethylthiazol-2-yl)-5-(3-carboxymethoxyphenyl)-2-(4-sulfophenyl)-2*H*-tetrazolium (MTT) test was employed to assess the cytotoxic potential of the compound. Griess reaction was used to determine nitrite production. Values represent the mean ± SD obtained from triplicate determinations repeated a minimum of three times. ***p<0.001 (compared to basal).

Pharmacokinetics

Liquid chromatography-tandem mass spectrometry conditions:

The aim of the study was to develop and validate a quantization method for **12d** in mouse brain and plasma using liquid chromatography coupled to mass spectrometry (LC-MS).

Instrumentation: Mass spectrometer: Api4000 SCIEX; HPLC system: Agilent 1290, Analytical HPLC and Column oven: Agilent 1290. Auto sampler: CTC Pal-xt. Mobile Phase A: Water/Acetonitrile (90/10), 0.1% Formic acid. Mobile Phase B: Acetonitrile/Water (90/10), 0.1% Formic acid. Rising solution A: Water. Rising solution B: Acetonitrile/Water (80/20). Chromatographic conditions: Chromatographic column: C18 2.1x 5 mm, 3 µm (Supelco). Flow rate: 0.45 mL. Oven temperature: 20 ºC. Injection volume: 5 µL. Run time: 5.0 min. Auto-sampler temperature: 4 ºC. Gradient.

Mass spectrometry conditions: The analyte and internal standards were detected using triple quadrupole mass spectrometry with electrospray ionization in positive mode. Mass/charge employed in this method are outlined next: m/z analyte: 453.4/144.1; 453.4/315.1 and B16 compound was used as Internal standard their m/z internal standard 427.2/289.3. Parameters, voltage: 5000 V, temperature 500 ºC. Standard stock solutions: independent stock solutions of analyte an internal standard was prepared in 100% DMSO to get a concentration of 0.5 mg/mL.

Sample preparation:

A 50 µL aliquot of plasma and brain were added with 3x organic solvent (acetonitrile) to precipitate proteins, then the samples were centrifugated at 13500 rpm for 15 minutes at 4ºC. The supernatant was collected for LCMS analysis. Previously, brain samples were homogenised using a tissue disruptor and adding phosphate buffer (3x).

Analytical standards were prepared as follows: 1) Standard blank samples were prepared by dispensing 2 µL of 100% DMSO into 50 µL of Plasma. 2) Calibration Curve samples were prepared by dispensing 2 µL of standard calibration curve solution into 50 µL of Plasma. 3) Standard Quality control samples were prepared by dispensing 2 µL of standard QC solution into 50 µL of Plasma.

Calibration curve solutions were prepared and diluted with 100% DMSO, from the previous combined standard stock solution to achieve a concentration range from 2 ng/mL to 512 ng/mL in brain and from 4 ng/mL to 512 ng/mL in plasma. Two independent standard curves in mouse plasma and brain samples were prepared and analyzed covering both ranges. Internal standard combined solution was transferred into a final volume of 10 mL acetonitrile to get a final concentration of 250 ng/mL, which was used to precipitate the proteins.

Quality control samples (QC) were prepared from an independent analyte standard solution preparation (standard quality control solution).

Quantification method in brain and plasma samples

Chromatography: **12d** (PIP01) displayed a retention time of 2.4 minutes. Figures S7 and S8 show the chromatograms for analyte and Internal standard for blank and the lower limit of quantitation samples in brain, respectively.

Specificity was investigated using six blank samples. No significant interferences were observed at the retention time of analyte and internal standards in brain and plasma samples (Table S36 and Figures S7 and S8).

The lower limits of quantification were established at 2 ng/ml and 4 ng/ml in brain and plasma, respectively, and the upper limit at 512 ng/ml in both matrices.

The linearity of the calibration curves was higher than 0.995 over the entire range in plasma and brain. The ratio of PIP01 (**12d**) peak area to internal standard is related to internal standard concentration using a linear fit, with a weighting of 1/x (where x is the analyte concentration). Figures S11 and S12 are the representative standard curves for PIP01 (**12d**) in mouse brain and plasma samples. All standard Calibration curve samples back calculated concentrations met the acceptance criteria for precision (CV ± 20.0 %) and accuracy (± 20.0 % of theoretical concentration).

Standard Calibration curve samples calculated concentrations in mouse brain and plasma samples are displayed in Tables S37 and S38.

Method precision (CV %) and accuracy (%) using 6 replicates of QC samples of PIP01(**12d**) in mouse brain samples are presented in Table S39. All QC samples met the acceptance criteria for Precision (CV ± 20.0 %) and Accuracy (± 20.0 % of theoretical concentration).

The peak area of the analyte at the retention time set in the mouse plasma quality control samples was compared the peak area of the analyte at the retention time set in the non-matrix based samples to calculate the method recovery.

Mean recovery values for PIP01 (**12d**) at 10, 100, 500 ng/ml concentration levels, were 100, 96 and 76% in brain and 89, 100, 89% in plasma, respectively.

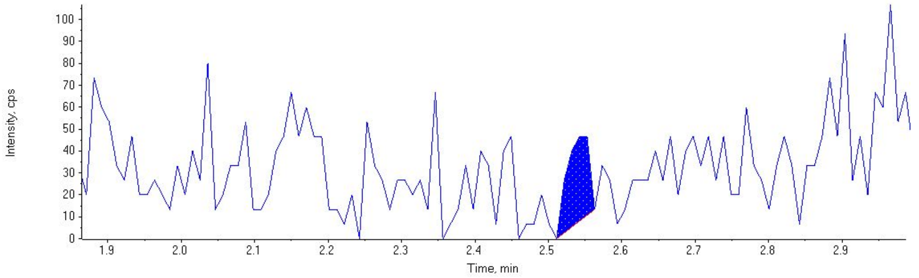

A. Peak of PIP01 (**12d**).

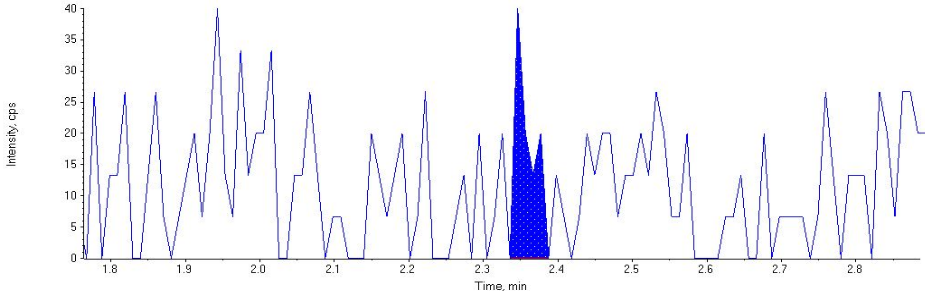

B. Peak of Internal standard.

Figure S7. Double Blank Sample Chromatogram of PIP01 (**12d**) and Internal Standard in mouse brain samples.

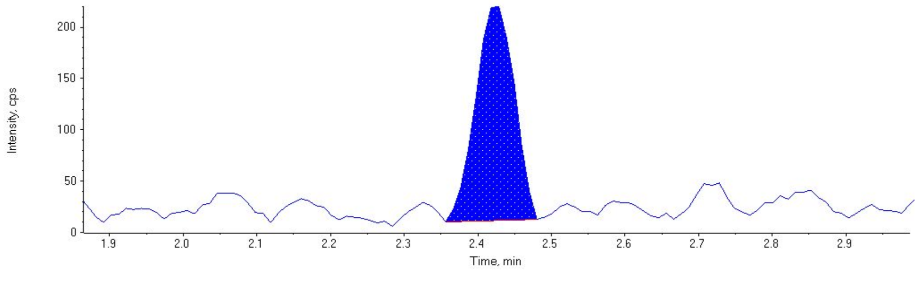

A. Peak of PIP01 (**12d**).

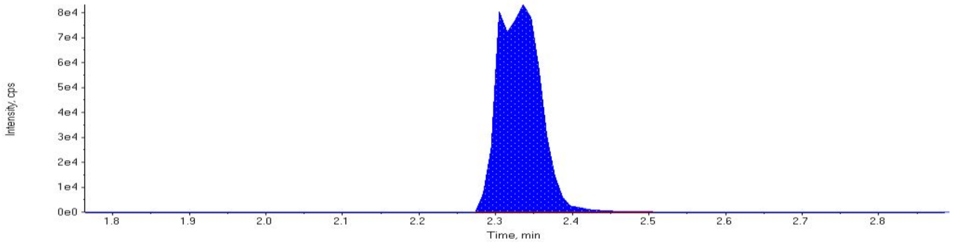

B. Peak of internal standard.

Figure S8. Chromatogram of PIP01 (**12d**) and internal standard in LLOQ sample in mouse brain samples (Concentration 2 ng/mL).

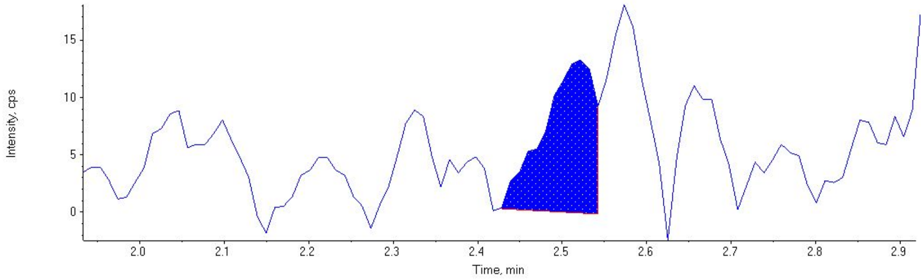

A. _Peak of PIP01 (**12d**).

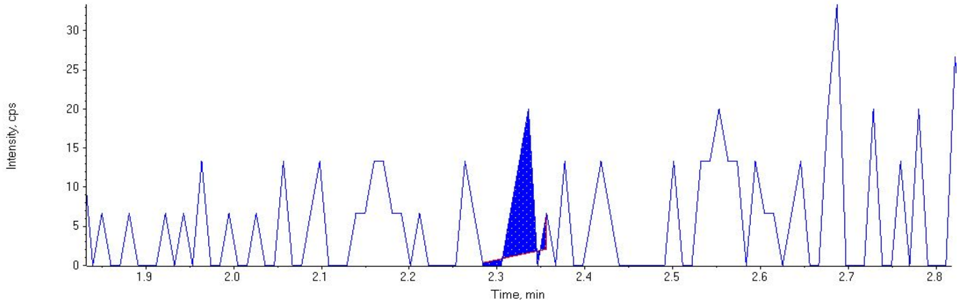

B. Peak of internal standard.

Figure S9. Double Blank Sample Chromatogram of PIP01 (**12d**) and Internal Standard in mouse plasma samples.

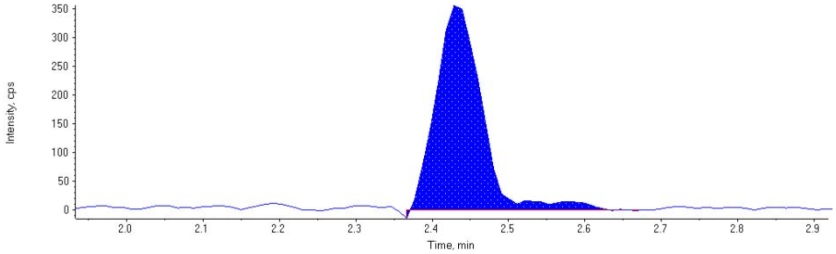

A. Peak of PIP01 (**12d**).

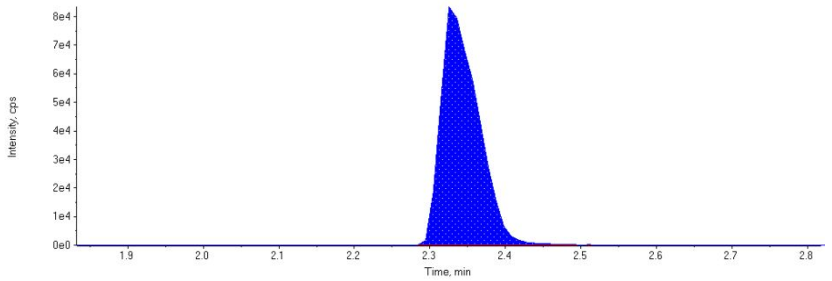

B. Peak of internal standard

Figure S10. Chromatogram of PIP01 (**12d**). and internal standard in LLOQ sample in mouse plasma samples (Concentration 4 ng/mL).

Table S36. Specificity of PIP01 (**12d**) and internal standard in mouse brain samples.

	Peak area at PIP01 RT in Blank	Peak area at PIP01 RT in LLOQ Sample	Blank Peak area at PIP01 RT (% of LLOQ)	Peak area at IS RT in Blank	Peak area at IS RT in LLOQ Sample	Blank Peak area at IS (% of LLOQ)
Blank 1	82.7	1540.0	5.4	57.9	384000.0	0.02
Blank 2	51.7	1730.0	3.0	107.0	418000.0	0.03
Blank 3	45.5	1580.0	2.9	55.8	377000.0	0.01
Blank 4	84.7	1620.0	5.2	68.2	497000.0	0.01
Blank 5	82.7	1780.0	4.6	57.9	442000.0	0.01
Blank 6	57.9	1590.0	3.6	41.3	510000.0	0.01
n	6.0	6.0	6.0	6.0	6.0	6.0
Mean	67.5	1640.0	4.1	64.7	438000.0	0.02

Regression Equation: *y = 0.00169 x + 0.000646 (r = 0.9984) (weighting: 1 / x)*

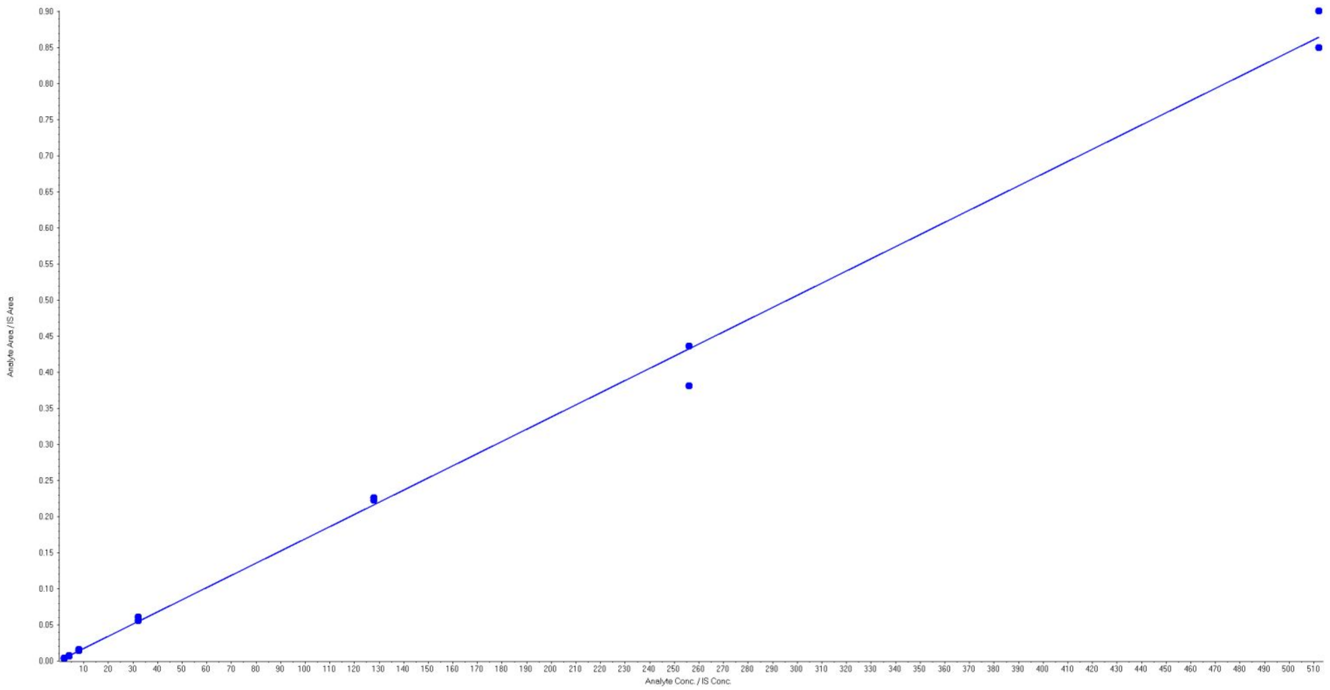

Figure S11. Standard calibration curve of PIP01(**12d**) in mouse brain samples

Table S37. Back calculated values (ng/mL) data of calibration curve standard for PIP01(**12d**) in mouse brain samples.

STD Conc. (ng/ml)	2.00	4.00	8.00	32.00	128.00	256.00	512.00
Run 1	1.98	3.51	8.12	32.56	131.86	258.48	533.59
Run 2	1.71	3.94	9.19	35.89	133.78	225.91	503.50
Mean Calculated Concentration (ng/mL)	1.84	3.72	8.65	34.23	132.82	242.19	518.54
Accuracy (%)	92.2	93	108.2	107	103.8	94.6	101.3
Precision (CV %)	12.2	14.4	5.8	17.6	9.5	2.4	5.7
n	2	2	2	2	2	2	2

Regression Equation: *y = 0.00145 x + -0.000225 (r = 0.9987) (weighting: 1 / x)*

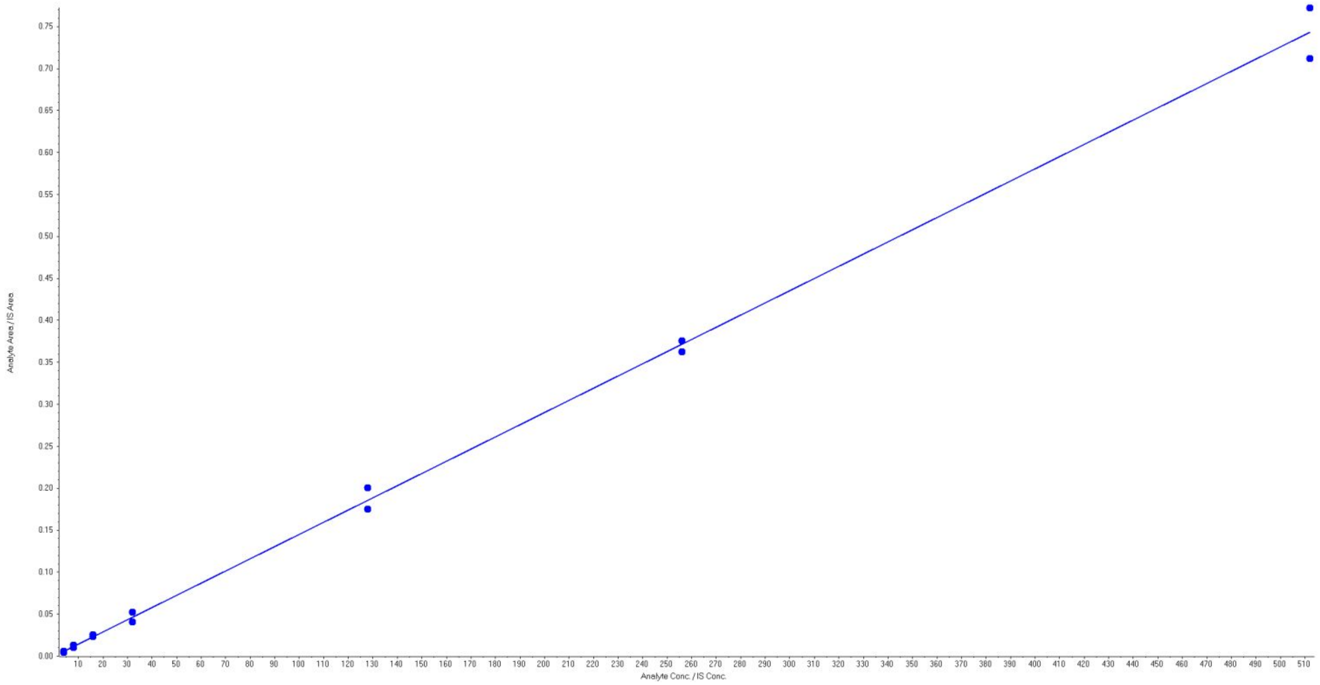

Figure S12. Standard calibration curve of PIP01(**12d**) in mouse plasma samples

Table S38. Back calculated values (ng/mL) data of calibration curve standard for PIP01 (**12d**) in mouse plasma samples.

STD Conc. (ng/ml)	4.00	8.00	16.00	32.00	128.00	256.00	512.00
Run 1	1.98	3.51	8.12	32.56	131.86	258.48	533.59
Run 2	1.71	3.94	9.19	35.89	133.78	225.91	503.50
Mean Calculated Concentration (ng/mL)	3.62	8.21	16.88	32.35	129.29	254.34	511.31
Accuracy (%)	90.6	102.6	105.5	101.1	101.0	99.4	99.9
Precision (CV %)	10.3	8.2	8.7	6.9	1	9.5	4.1
n	2	2	2	2	2	2	2

Table S39. Accuracy and Precision of PIP01 (**12d**) in mouse brain samples.

**Quality Control**	**LQC (2)**	**MQC (57)**	**HQC (512)**
**Concentration (ng/ml)**	1.99	58.93	441.73
	2.07	59.76	433.20
	2.10	60.09	483.54
	1.53	53.90	422.75
	1.78	55.77	471.73
	1.55	52.79	400.79
**Mean**	1.84	56.87	442.29
**Precision (CV %)**	13.93	5.54	6.97
**Accuracy (%)**	91.86	99.78	86.38
n	6	6	6

MoA Alzheimer’s panel (Eurofins)

Alzheimer's MoA Panel (Total # of Assays: 41) (https://www.eurofinsdiscovery.com/catalog/PP284)

Study Objectives

To evaluate, in Alzheimer's MoA Panel, the activity of test compound **12d**, in Enzyme, and Radioligand Binding assays, the activity of the test compound **12d** (UBNA-13, PT#1278609).

Methods:

Methods employed in this study have been adapted from the scientific literature to maximize reliability and reproducibility. Reference standards were run as an integral part of each assay to ensure the validity of the results obtained.

Where presented, IC_{50} values were determined by a non-linear, least squares regression analysis using MathIQTM (ID Business Solutions Ltd., UK). Where inhibition constants (Ki) are presented, the Ki values were calculated using the equation of Cheng and Prusoff (Cheng, Y., Prusoff, W.H., Biochem. Pharmacol. 22:3099-3108, 1973) using the observed IC50 of the tested compound, the concentration of radioligand employed in the assay, and the historical values for the KD of the ligand (obtained experimentally at Eurofins Panlabs, Inc.). Where presented, the Hill coefficient (nH), defining the slope of the competitive binding curve, was calculated using MathIQTM. Hill coefficients significantly different than 1.0, may suggest that the binding displacement does not follow the laws of mass action with a single binding site. Where IC_{50}, Ki, and/or nH data are presented without Standard Error of the Mean (SEM), data are insufficient to be quantitative, and the values presented (Ki, IC_{50}, nH) should be interpreted with caution.

Conclusion:

Significant results are displayed in the following table in rank order of potency for estimated IC_{50} and/or Ki values.

Significant responses (≥ 50% inhibition or stimulation for Biochemical assays) were noted in the primary assays listed below: <u>*No significant results noted.*</u>

Table S40. Biochemical assays, concentration of the compound assessed 12d and IC_{50}.

Experimental Results

Cat #	Assay Name	Batch*	Spec.	Rep.	Conc.	% Inh.	IC_{50}*	K_i	n_H	R
**Compound: PIP01, PT #: 1278609**										
199019	Catechol-O-Methyl Transferase (COMT)	508107	hum	2	10 µM	-3				
104010	Cholinesterase, Acetyl, ACES	508098	hum	2	10 µM	-17				
104050	Cholinesterase, Butyryl, CHLE	508256	hum	2	10 µM	0				
116030	Cyclooxygenase COX-1	508103	hum	2	10 µM	9				
118030	Cyclooxygenase COX-2	508104	hum	2	10 µM	1				
199008	Fatty Acid Amide Hydrolase (FAAH)	508106	hum	2	10 µM	-3				
125810	IDO1	508199	hum	2	10 µM	-6				
199020	Lipoxygenase 15-LOX-2	508258	hum	2	10 µM	3				
140010	Monoamine Oxidase MAO-A	508156	hum	2	10 µM	3				
140120	Monoamine Oxidase MAO-B	508157	hum	2	10 µM	35				
121000	Myeloperoxidase	508153	hum	2	10 µM	3				
199010	Nitric Oxide Synthetase, Inducible (iNOS)	508257	mouse	2	10 µM	6				
164700	Peptidase, BACE1 (β-Secretase)	508155	hum	2	10 µM	1				
112250	Peptidase, CTSB (Cathepsin B)	508097	hum	2	10 µM	-90				1
163950	Peptidase, Endothelin Converting Enzyme-1 (ECE-1)	508200	hum	2	10 µM	7				
164010	Peptidase, Metalloproteinase, Neutral Endopeptidase	508154	hum	2	10 µM	0				
168040	Protein Serine/Threonine Kinase, CAMK2D (KCC2D)	508319	hum	2	10 µM	43				
176500	Protein Serine/Threonine Kinase, GSK3B	508254	hum	2	10 µM	1				
176610	Protein Serine/Threonine Kinase, MAPK14 (p38α)	508159	hum	2	10 µM	-1				
172020	Protein Tyrosine Kinase, Fyn	508152	hum	2	10 µM	11				
189240	Protein Tyrosine Phosphatase, DUSP22	508108	hum	2	10 µM	5				
200610	Adenosine A_{2A}	508206	hum	2	10 µM	-4				
244600	Chemokine CX3CR1	508407	hum	2	10 µM	37				
226630	$GABA_A$, Ro-15-1788, Hippocampus	508202	rat	2	10 µM	11				
232600	Glutamate, AMPA	508287	rat	2	10 µM	7				
237000	Glutamate, Metabotropic, $mGlu_5$	508292	hum	2	10 µM	-4				
233010	Glutamate, NMDA, MK-801	508260	rat	2	10 µM	4				
234000	Glutamate, NMDA, Polyamine	508114	rat	2	10 µM	5				
239820	Histamine H_3	508134	hum	2	10 µM	1				
252610	Muscarinic M_1	508133	hum	2	10 µM	-6				
258660	Nicotinic Acetylcholine α7, Methyllycaconitine	508164	hum	2	10 µM	15				
299037	Platelet Activating Factor (PAF)	508213	hum	2	10 µM	10				
271120	Serotonin (5-Hydroxytryptamine) $5-HT_{1A}$, WAY-100635	508266	hum	2	10 µM	1				
272010	Serotonin (5-Hydroxytryptamine) $5-HT_{4B}$	508167	hum	2	10 µM	19				
272200	Serotonin (5-Hydroxytryptamine) $5-HT_6$	508272	hum	2	10 µM	-1				
299007	Sigma σ_2	508176	hum	2	10 µM	13				
299034	Sigma σ_1	508272	hum	2	10 µM	0				
279450	Sodium Channel, Site 1	508181	rat	2	10 µM	10				
279510	Sodium Channel, Site 2	508182	rat	2	10 µM	-6				
282910	Somatostatin sst4	508179	hum	2	10 µM	10				
226400	Transporter, GABA	508116	rat	2	10 µM	-3				

[illegible]

Western Blot Analysis for Neurochemical Markers

Table S41. Antibodies used in Western blot studies.

Antibodies	Host	Source/Catalog	WB dilution
PDE1B	Mouse	Santa Cruz/sc-393112	1:1000
SYT7	Mouse	Santa Cruz/sc-293343	1:1000
HPCA	Mouse	Santa Cruz/sc393125	1:1000
CAMK2	Rabbit	Abcam/ab52476	1:1000
GAPDH	Mouse	Millipore/MAB374	1:5000
Goat-anti-mouse HRP conjugated		Biorad/170-5047	1:2000
Goat-anti-rabbit HRP conjugated		Biorad/170-6515	1:2000

RNA extraction and gene expression determination

Table S42. Primers and probes used in qPCR studies

Target	Forward Primer	Reverse Primer
TrkA	CTCCTTCTCGCCAGTGGAC	TGCCCTCAGTAGGGGAAAGA
Trkß	CGTCACTTCGCCAGCAGTAG	CTATACGCCAGGCACCACTC
Ngf	GGAGCGCATCGAGTGACTT	CCTCACTGCGGCCAGTATAG
Bdnf	TGCGAGTATTACCTCCGCCAT	TCACGTGCTCAAAAGTGTCAG
ß-actin	CAACGAGCGGTTCCGAT	GCCACAGGTTCCATACCCA

Reduction of capsaicin-induced mechanical hypersensitivity by CR4056 in mice, and contribution of imidazoline receptor-2 (I_2R) to its effects

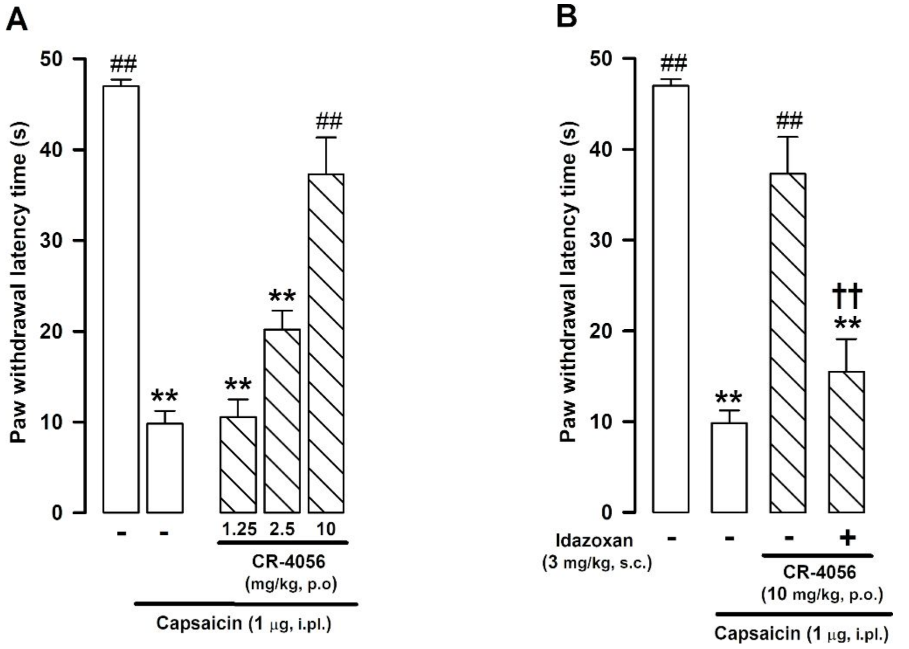

Figure S13. Reduction of capsaicin-induced mechanical hypersensitivity by CR4056 (**1**) in mice, and contribution of I_2Rs to its effects. A) Dose dependency of the antinociceptive effects induced by the oral (p.o.) administration of CR4056. B) Effects of CR4056 (**1**) alone and associated with the subcutaneous (s.c.) administration of the I_2 antagonist idazoxan (**4**) (3 mg/kg). Values are the mean ± SEM obtained from 7–9 animals per group: **p<0.01 vs non-sensitized animals treated with the vehicle of the drug solvent; ## p<0.01 vs capsaicin-injected mice treated with the vehicle of the drug; †† p<0.01 CR4056-treated animals associated with idazoxan or its solvent (one-way ANOVA followed by Bonferroni test).

Molecular Formula Strings (SMILES)

Table S42: Molecular Formula Strings of compounds **12a-o**.

Comp.	Structure	SMILE	I_2-IR Binding Affinities (p*K*i)	α_2-AR Binding Affinities (p*K*i)	I_1-IR Binding Affinities (p*K*i)	Selectivity (I_2/α_2)	Selectivity (I_2/I_1)
**12a**	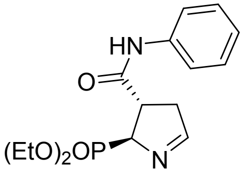	O=C(NC1=CC=CC=C1)[C@H]2[C@H](P(OCC)(OCC)=O)N=CC2	4.64±0.47	6.00±0.51	7.84±0.24	-	0.0014
**12b**	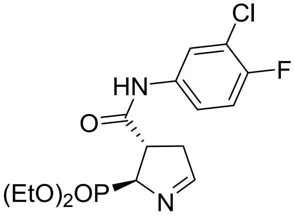	O=C(NC1=CC=C(F)C(Cl)=C1)[C@H]2[C@H](P(OCC)(OCC)=O)N=CC2	5.61±0.21	7.41±0.18	<3	-	-
**12c**	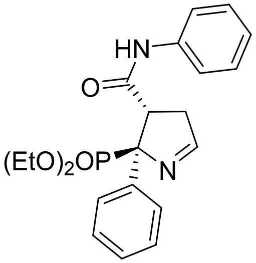	O=C(NC1=CC=CC=C1)[C@H]2[C@](C3=CC=CC=C3)(P(OCC)(OCC)=O)N=CC2	3.52±0.64	6.55±0.32	7.58±0.47	-	0.0001

**12d**	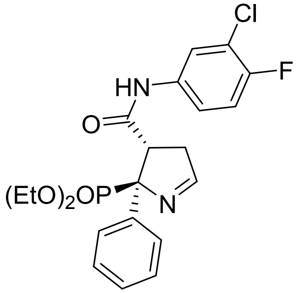	O=C(NC1=CC=C(F)C(Cl)=C1)[C@H]2[C@](C3=CC=CC=C3)(P(OCC)(OCC)=O)N=CC2	9.98±0.74	9.43±0.22	<3	3	-
**12e**	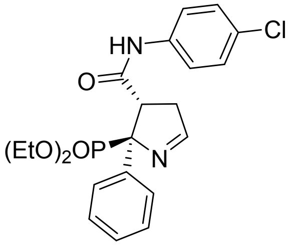	O=C(NC1=CC=C(Cl)C=C1)[C@H]2[C@](C3=CC=CC=C3)(P(OCC)(OCC)=O)N=CC2	<3	<3	ND[a]	-	-
**12f**	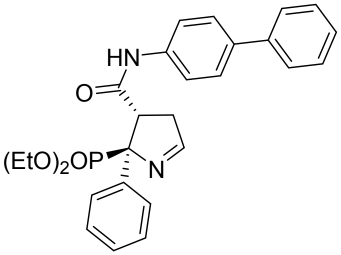	O=C(NC1=CC=C(C2=CC=CC=C2)C=C1)[C@H]3[C@](C4=CC=CC=C4)(P(OCC)(OCC)=O)N=CC3	6.01±0.17	4.67±0.26	ND	22	-
**12g**	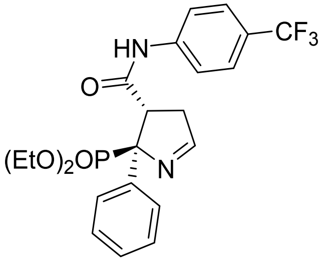	O=C(NC1=CC=C(C(F)(F)F)C=C1)[C@H]2[C@](C3=CC=CC=C3)(P(OCC)(OCC)=O)N=CC2	7.04±0.28	5.33±0.88	4.68±0.27	51	229.1

**12h**	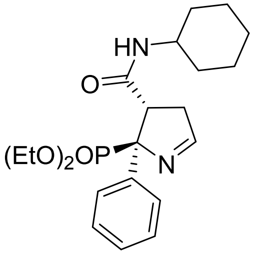	O=C(NC1CCCCC1)[C@H]2[C@](C3=CC=CC=C3)(P(OCC)(OCC)=O)N=CC2	3.57±0.21	3.60±0.24	4.31±0.05	0.9	0.182
**12i**	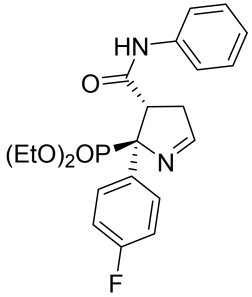	O=C(NC1=CC=CC=C1)[C@H]2[C@](C3=CC=C(F)C=C3)(P(OCC)(OCC)=O)N=CC2	5.03±0.41	<3	ND	-	-
**12j**	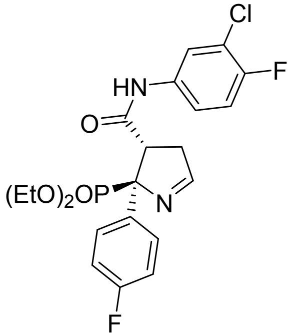	O=C(NC1=CC=C(F)C(Cl)=C1)[C@H]2[C@](C3=CC=C(F)C=C3)(P(OCC)(OCC)=O)N=CC2	3.71±0.27	3.56±0.34	ND	1.4	-

**12k**	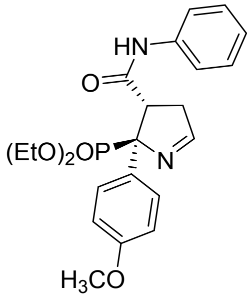	O=C(NC1=CC=CC=C1)[C@H]2[C@](C3=CC=C(OC)C=C3)(P(OCC)(OCC)=O)N=CC2	3.07±0.47	<3	ND	-	-
**12l**	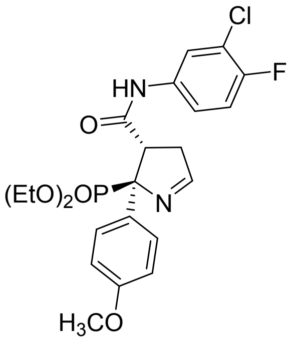	O=C(NC1=CC=C(F)C(Cl)=C1)[C@H]2[C@](C3=CC=C(OC)C=C3)(P(OCC)(OCC)=O)N=CC2	3.99±0.18	3.24±0.72	5.18±0.18	5.6	0.065
**12m**	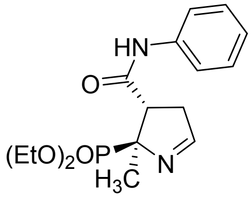	O=C(NC1=CC=CC=C1)[C@H]2[C@](C)(P(OCC)(OCC)=O)N=CC2	<3	7.27±0.46	ND	-	-
**12n**	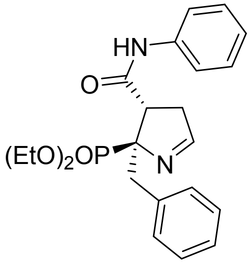	O=C(NC1=CC=CC=C1)[C@H]2[C@](CC3=CC=CC=C3)(P(OCC)(OCC)=O)N=CC2	8.38±0.38	3.90±0.13	<3	6530	-

**12o**	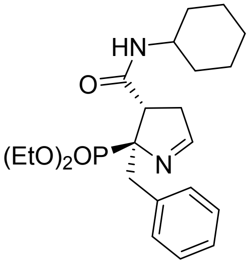	O=C(NC1CCCCC1)[C@H]2[C@](CC3=CC=CC=C3)(P(OCC)(OCC)=O)N=CC2	5.22±0.14	7.97±0.36	ND	-	-

[a] ND = Not determined.

References

(1) Tropsha, A.; Best Practices for QSAR Model Development, Validation, and Exploitation. *Mol. Inform.* **2010**, 29, 476.

(2) Golbraikh, A.; Tropsha, A. Beware of q2. *J. Mol. Graph. Model.* **2002**, 20, 269.

(3) Ojha, P.K.; Roy, K.. Comparative QSARs for antimalarial endochins:importance of descriptor thinning and noise reduction prior to feature selection. *Chemom. Intell. Lab. Syst.* **2011**, 109, 146–161.

(4) SPSS Inc. Released 2009. PASW Statistics for Windows, Version 18.0. Chicago: SPSS Inc.

(5) Antonie, D.; Oliver, M.; Vicent, Z.; SwissADME: a free web tool to evaluate pharmacokinetics, drug-likeness and medicinal chemistry friendliness of small molecules. *Sci. Rep.* **2017,** 7, 1–13.

(6) A.D.M.E.T. PredictorSimulations Plus Inc.v. 9.5, Lancaster, CA, USA. Available in https://www.simulations-plus.com.

(7) Di, L.; Kerns, E. H.; Fan, K.; McConnell, O. J.; and Carter, G. T. High throughput artificial membrane permeability assay for blood-brain barrier. *Eur. J. Med. Chem*. **2003**, *38*, 223-232.

(8) Di, L.; Kerns, E. H.; Chen, H.; Petusky, S. L. Development and application of an automated solution stability assay for drug Discovery. *J. Biomol. Screen.* **2006**, 11, 40-47.
